# Novel Competitive, Nonpeptidic, SARS-CoV‑2 M^pro^ Inhibitors with Improved Solubility

**DOI:** 10.1021/acsmedchemlett.6c00041

**Published:** 2026-05-06

**Authors:** Zafer Sahin, Mario Rivera, Yanli Yang, Ken Liu, Goknil P. C. Sahin, John Bacsa, Stephen C. Pelly, Dennis C. Liotta

**Affiliations:** † Department of Chemistry, 1371 Emory University College of Arts and Sciences, Atlanta, Georgia 30322, United States; § Department of Pharmaceutical Chemistry, Faculty of Pharmacy, Acibadem Mehmet Ali Aydinlar University, 34752 Istan-bul, Türkiye

**Keywords:** SARS-CoV-2, COVID-19, main protease, FEP+

## Abstract

In continuation of our previous work wherein we described novel, nonpeptidic competitive inhibitors of the SARS-CoV-2 main protease, we here describe our efforts to improve upon these compounds by improving water solubility and metabolic stability. As predicted by FEP+ solubility studies, disrupting the coplanar arrangement of the aromatic rings led to significantly improved compound aqueous solubility, unfortunately with an unacceptable loss in potency. Attempts to improve compound metabolic stability by avoiding aromatic moieties occupying the S4 pocket, while retaining compound potency by modification of the pyridyl ring occupying the S1 pocket, were also investigated.

## Graphical abstract

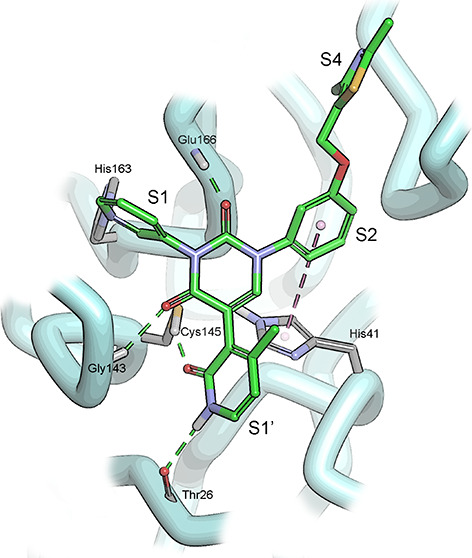

Although the severity of the COVID-19 pandemic has decreased significantly due to widespread vaccination campaigns and the development of natural immunity over time,^1^ several groups remain at risk of developing severe symptoms (requiring hospitalization) upon becoming infected. Older individuals (65 and older) remain at substantially higher risk, as well as immunocompromised individuals and those with certain underlying medical conditions such as COPD (chronic obstructive pulmonary disease), heart disease and diabetes.^2^ Indeed, although Paxlovid (the most widely used antiviral medication to treat COVID-19) has proven to be very effective, its coformulation of nirmatrelvir (a SARS-CoV-2 main protease inhibitor) with ritonavir (a CYP3A4/5 inhibitor) typically complicates its use in individuals currently on other chronic medication.^3^ Nevertheless, the success of the drug highlights the effectiveness of targeting the SARS-CoV-2 M^pro^ (main protease),^4^ which is an essential viral enzyme needed for replication. Only two other antiviral drugs are currently approved by the FDA for the treatment of COVID-19, namely Veklury (remdesivir) and Lagevrio (molnupiravir, emergency use authorization). Unlike nirmatrelvir which is a protease inhibitor, remdesivir and molnupiravir are substrates for the RNA-dependent RNA polymerase (RdRp), although they each have different mechanisms of action after incorporation into the RNA strand. Unfortunately, both of these drugs also have shortcomings. Veklury must be administered intravenously, limiting its broader usage. Lagevrio on the other hand, although orally available, has generally been found to be less effective than Paxlovid,^5^ and there have been concerns regarding molnupiravir’ s mechanism of action and mutagenicity.^6^ Finally, Xocova (ensitrelvir), which similarly to nirmatrelvir is a SARS-CoV-2 M^pro^ inhibitor, is approved in Japan for the treatment of COVID-19. Unfortunately, although ensitrelvir does not need to be coadministered with ritonavir (as is the case with nirmatrelvir), the drug may nevertheless present the same disadvantages as Paxlovid since ensitrelvir is known to be a strong inhibitor of CYP3A4.^7^ Thus, there remains an ongoing need to develop next generation SARS-CoV-2 M^pro^ inhibitors capable of circumventing these problems.

In 2023, we described our efforts in developing novel, competitive, nonpeptidic, SARS-CoV-2 M^pro^ inhibitors.^8^ Having settled on suitable functionality to occupy the S1, S1’ and S2 pockets (Figure 1A), we thoroughly explored the SAR landscape within the S4 pocket, guided by computational structure-based design and in particular, calculation of binding free energy by free energy perturbation methods (FEP+). Emanating from this work, compound **1** for example, displayed low nanomolar potency in a SARS-CoV-2 recombinant protease enzymatic assay (IC_50_) and submicromolar potency in a cellular antiviral assay (EC_50_).

**Figure 1:**
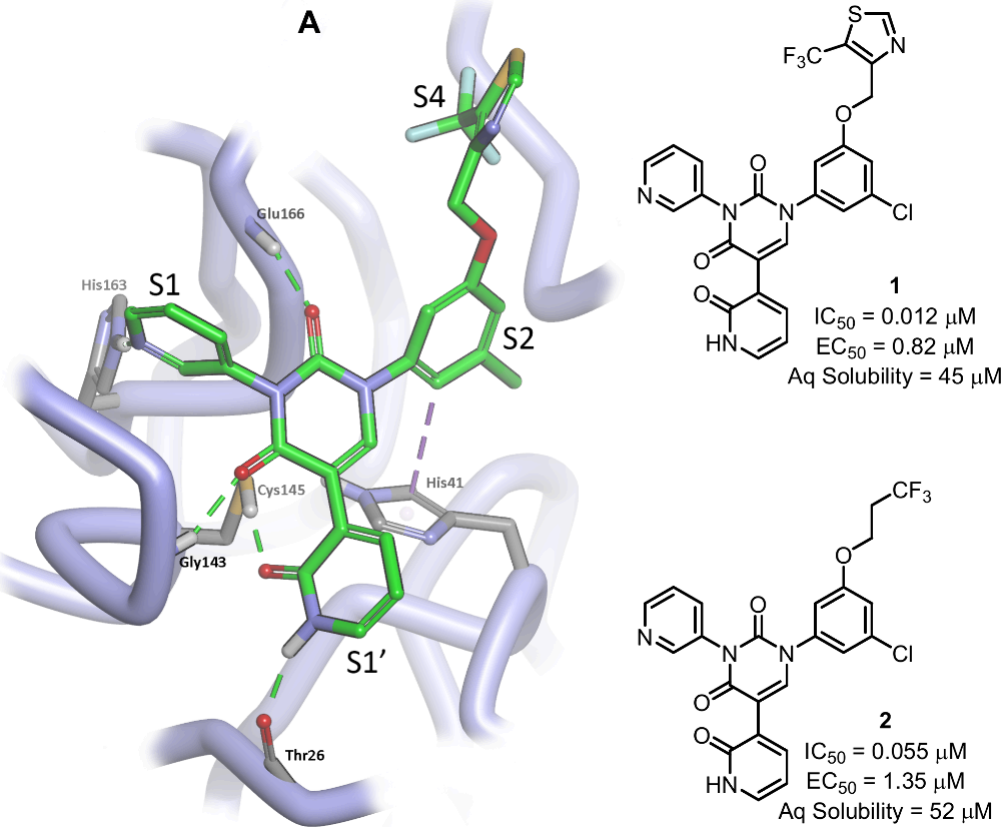
Examples of potent compounds from our previous work (**1** and **2**) and a docked structure of **1** showing efficient occupation of the SARS-CoV-2 M^pro^ active site pocket. A strong correlation between our calculated binding energy values (ΔG, FEP+) and those derived empirically from our IC_50_ values within our compound series provided us with a high level of confidence in the accuracy of our docked poses.

Unfortunately, within this compound series we ran into two major difficulties. First, although compounds containing aromatic or heteroaromatic groups occupying the S4 pocket (E.g. **1**) were more potent than their aliphatic counterparts (E.g. **2**), these compounds typically suffered from poor metabolic stability. Second, most of the compounds displaying good potency had poor solubility (typically <60 μM in PBS buffer). Here we describe our efforts to overcome these problems.

Our initial approach focused on improving solubility to above 100 μM. Unfortunately, outside of the M^pro^ binding pocket, these compounds are not only the epitome of “chemical flatland”, but are also able to very effectively Pi-stack, further hampering solubilization. It is indeed an interesting observation, however, that within the binding pocket, all aromatic rings are, to a greater or lesser extent, orthogonal to each other (Figure 1). Thus, it became our focus to perturb the coplanar nature of at least two of the rings, thereby disrupting the highly stable Pi-stacking interactions of the molecules upon solidification. To this end, we initially chose to introduce functionality at the 4-position of the pyridone ring occupying the S1’ pocket, as this part of the molecule faces out into the solvation region, and adding functionality here would not cause any undesirable steric clashes with the receptor (Figure 2). The choice of substituent became an important decision since the pyridone ring is not symmetrical and therefore although we wished to disrupt the coplanar nature of the two rings, we did not want to go so far as to form atropisomers, as only one of these isomers would be able to bind favorably. We calculated the barrier to rotation about the indicated biaryl axis (Figure 2) for several small groups and eventually settled on a methyl group as our first proof of concept compound, thereby increasing the rotational barrier from about 3 kcal/mol to about 12 kcal/mol. It is also interesting to note just how simple cLogP calculations can be misleading in a setting such as this, as cLogP values suggested that the structural change from **3** to **4** would only worsen the solubility (increase in cLogP). However, more rigorous solubility calculations by free energy perturbation methods (FEP+)^9^ suggested a 17-fold improvement in solubility upon transforming **3** to **4**.

**Figure 2:**
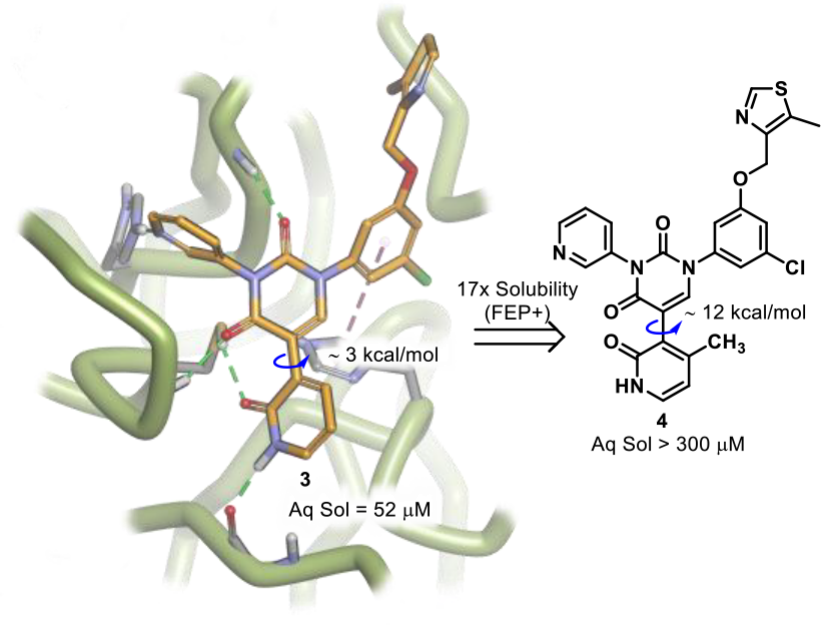
Introduction of a methyl substituent at the 4-position of the pyridone increases the barrier to rotation about the indicated biaryl axis, disrupting Pi-stacking and improving kinetic solubility (Aq Sol), which was predicted by FEP+ Solubility.

Synthesis of **4** (Scheme 1) was carried out in a similar manner to our previously reported synthesis, involving Suzuki coupling of 3-bromo-4-methyl-2-methoxypyridine **5** and boronic acid **6**, thereby furnishing **7** in good yield. Debenzylation under hydrogenation conditions revealed our core uracil moiety **8**, allowing for installation of **9** using a modified version of the Ulmann-Goldberg reaction.^10^ This key reaction allowed for selective coupling at N1 of the uracil, affording **10**, albeit in modest yields. With N1 now functionalized, a Chan-Lam type coupling at N3 with 3-pyridinylboronic acid **11** provided **12** in good yield. Removal of the benzyl protecting group on **12** once again by hydrogenation, allowed for the installation of the S4 pocket functionalities, and here we used this as a point of late-stage diversification and took the opportunity to investigate four different groups (**14–17**), which we had found to be most effective in our previous work. Finally, demethylation of the 2-methoxy pyridyl derivatives using TMSCl and NaI, afforded target compounds **4** and **18–20**. Interestingly, we were able to obtain a crystal structure of intermediate **10** which did reveal an orthogonal orientation of the methoxy-pyridine ring and the central uracil moiety (please see SI).

**Scheme 1:**
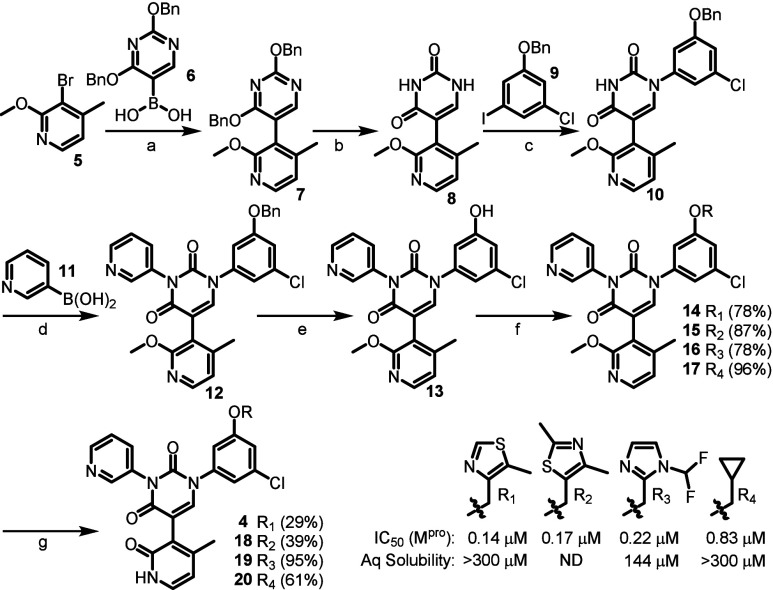
Reagents and conditions: a) Pd­(PPh_3_)_4_, NaHCO_3_, DME/water, 90%; b) 10% Pd/C, H_2_, MeOH/THF (1:1), 90% c) CuI, K_3_PO_4_, N-(2-cyanophenyl)­picolinamide, DMSO, 52%; d) Cu­(OAc)_2_, TMEDA, DMSO, 94%; e) 10% Pd/C, H_2_, MeOH/THF (1:1), mixture, please see SI Scheme S1; f) K_2_CO_3_, R-X, DMF; g) TMS-Cl, NaI, MeCN. (Aq Solubility = kinetic solubility in PBS buffer)

With the conformationally restricted compounds in hand (**4**, **18–20**), we were in a position to test our hypothesis regarding improved solubility. Pleasingly, the compounds were far more soluble in our kinetic solubility assay (Scheme 1), attesting to the utility of free energy perturbation techniques for solubility determination instead of simpler methods such as cLogP calculations. Unfortunately, these compounds performed poorly in a SARS-CoV-2 recombinant protease enzymatic assay. Generally, these methylated derivatives exhibited a 10-fold decrease in potency as compared to the original derivatives,^8^ which did not include the methyl on the pyridone ring. This large decrease in potency cannot be accounted for by restricted rotation alone about the biaryl axis, and therefore clearly there are electronic factors here that need to be considered as well. Indeed, studying these compounds using Schrödinger’s FEP+ relative binding free energy perturbation methods (RBFE) predicted just a 4-fold decrease in potency when comparing for example compounds **3** and **4** (Figure 2). Considering FEP+ cannot take into account binding opportunities lost due to restricted rotation about the biaryl axis, it is evident that the loss in potency is most likely caused by the combination of restricted rotation not allowing for fast enough conformational rearranging at the time of binding and decreased binding efficacy of even the correct conformation. Another point of interest is that during the synthesis of these methylated analogues, while carrying out the debenzylation reactions by hydrogenation (Scheme 1, step e), we inadvertently produced some of the dehalogenated analogues, which allowed us to carry these intermediates to the end of the synthesis (SI Scheme S1), thereby arriving at analogues **21**-**23** (Figure 3), sans the meta-chloro substituent on the phenyl ring. Although it has previously been reported that a chloro substituent occupying this part of the S2 pocket is beneficial for potency,^11,12^ these particular compounds highlight the significance a single change can make to a series, with an approximate 30-fold loss in potency observed for the three analogues. Interestingly, from a structure-based design point of view this phenomenon is not easily explained, since there is no obvious evidence of any halogen bonding occurring with the -Cl in the S2 binding pocket (Figure 3). However, analysis of the binding pocket using Schrödinger’s WaterMap does indeed reveal a high energy water located deep within the S2 pocket (6.9 kcal/mol) which would be favorably displaced by a hydrophobic substituent, such as for example the meta-chloro group on the phenyl ring, as well as another high energy water located in the S4 pocket (4.2 kcal/mol), which further explains our observation that hydrophobic groups in this pocket significantly enhance potency.^8^

**Figure 3:**
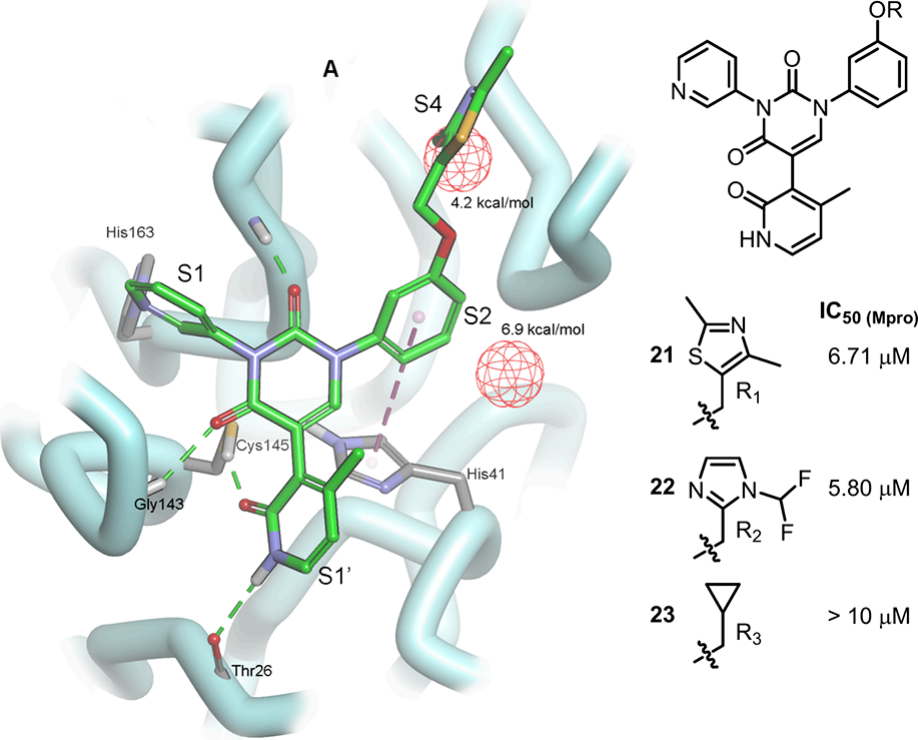
Docked illustration of **21** within the M^pro^ binding pocket, and analogues thereof, all with drastically reduced potency, highlighting the advantage of displacing the high energy water (6.9 kcal/mol) in the S2 pocket with an appropriate hydrophobic substituent located at the meta-position on the phenyl ring. Another high energy water molecule is located in the S4 pocket (4.2 kcal/mol), further rationalizing the observation that hydrophobic functionality in this pocket significantly boosts ligand potency.

With this information in hand, we carried out a small SAR study to investigate whether adjusting the size of the halogen attached to the phenyl group occupying the S2 pocket could optimize potency even further. To this end, in addition to our previously disclosed compound **39** (Scheme 2),^8^ we synthesized the corresponding fluoro **36** and bromo **37** analogues, as well as the para-fluoro analog **38**. Prior analysis of these compounds by FEP+ had indeed predicted that the bromo and chloro derivatives (**37** and **39** respectively) would be significantly more potent than the fluoro derivative (**36**), and this did indeed turn out to be the case after biochemical evaluation of these compounds. Interestingly, FEP+ also accurately predicted the 2-fold improvement in potency when comparing the *para*-fluoro derivative **38** to its closely related analog, **39**. Unfortunately, although interesting in terms of potency, compound **38** (with the additional fluorine) had poor aqueous solubility (23 μM in PBS buffer).

**Scheme 2:**
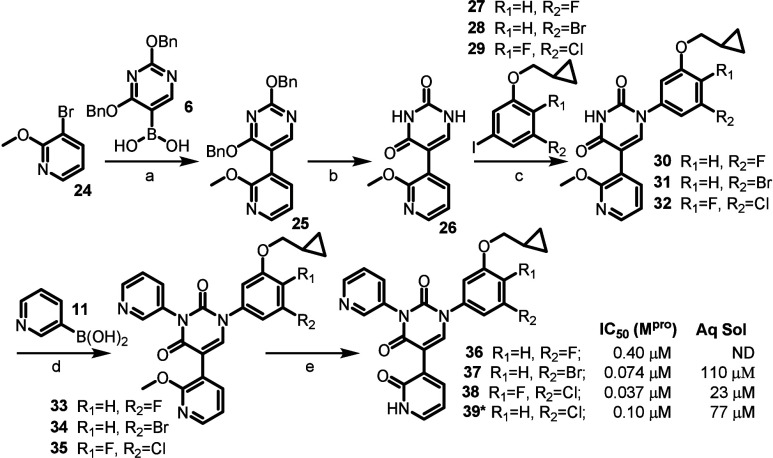
Reagents and conditions: a) Pd­(PPh_3_)_4_, NaHCO_3_, DME/water, 93%; b) 10% Pd/C, H_2_, MeOH/THF (1:1), 99% c) CuI, K_3_PO_4_, N-(2-cyanophenyl)­picolinamide, DMSO, **30** 72%, **31** 80%, **32** 25%; d) Cu­(OAc)_2_, TMEDA, DMSO, **33** 56%, **34** 96%, **35** 68%; e) TMS-Cl, NaI, MeCN, **36** 41%, **37** 56%, **38** 33%. *Note: **39** was not synthesized by way of this scheme. It was synthesized and evaluated as part of our previous work. (Aq Sol = kinetic solubility in PBS buffer).

Having observed only a modest improvement in potency by modifying the phenyl ring occupying the S2 pocket, we now turned our attention to the pyridyl ring in the S1 pocket, which forms a key hydrogen bond to His163 (Figure 3). To this end, we considered both the pyrimidine ring system (Figure 4, **41**), and the pyridazine ring system (Figure 4, **42–44**), but for very different reasons. In the case of the pyrimidine, we hypothesized that symmetry introduced into this part of the molecule would allow for more favorable binding opportunities. Although FEP+ did indeed predict a decrease in potency in switching to the pyrimidine, it cannot of course account for lost binding opportunities due to slow conformational rearrangement. Given the calculated barrier to rotation about the pyridyl-uracil bond is approximately 10 kcal/mol, we hypothesized that even though the pyrimidine ring system is a weaker hydrogen bond acceptor, the increased successful binding opportunities may lead to an improvement in potency. On the other hand, in the case of the pyridazine ring system, we hoped to overcome a slight steric clash observed between the pyridyl ortho-CH and His163, thereby improving the strength of the hydrogen bonding interaction. Furthermore, analysis by FEP+ suggested an order of magnitude improvement in potency for pyridazine **41** when compared to pyridine **3**, which was synthesized in our previous work (as the methyl analog of **1**).^8^ This was an attractive result as the predicted boost in potency would allow us to utilize aliphatic groups in the S4 pocket region, offsetting the previously experienced general loss in potency for these types of compounds, while having the added benefit of metabolically more stable functionality.

**Figure 4:**
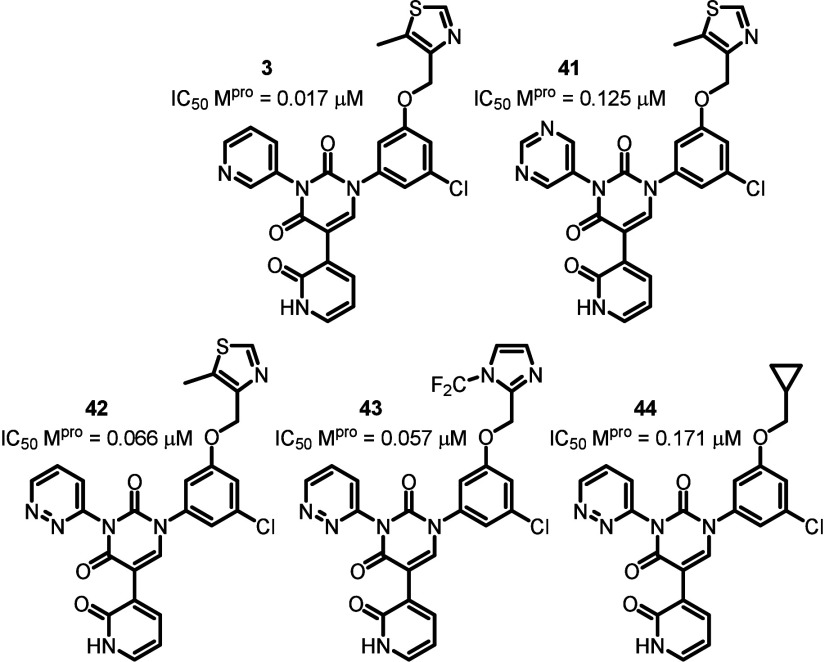
Pyridazine and pyrimidine analogs.

The synthesis of compounds **41–44** followed a very similar route to Scheme 2 (SI Scheme S2), with the only major difference being our methodology for coupling the pyrimidine moiety (for compound **41**) and the pyridazine moiety (for compounds **42–44**). To our astonishment, unlike the coupling of 3-pyridyl boronic acid to the uracil core in a Cham-Lam reaction (E.g. Scheme 2, step d) which had worked so well in our previous syntheses, this same methodology was completely ineffective for both the respective pyrimidine and pyridazine boronic acids. Indeed, to incorporate these two moieties we needed to revert back to the Goldberg methodology, using CuI and 5-iodopyrimidine (for compound **41**) or 3-iodopyridazine (for compounds **42–44**), all at higher temperatures. Even then, yields were poor and the reactions needed to be repeated several times in order to obtain enough material to continue with the syntheses in the usual manner. Nevertheless, through perseverance, we obtained compounds **41–44**, and we were able to evaluate them in our SARS-CoV-2 M^pro^ assay. As predicted by FEP+, the pyrimidine analog **41** did indeed turn out to be significantly less potent than the pyridine analog **3** (Figure 4). However, in contrast to the FEP+ analysis, the pyridazine analogs were not found to be significantly more potent than their pyridine counterparts and in fact also showed some reduction in potency when compared to their original pyridyl parent compounds.^8^

In our final attempt at increasing aqueous solubility and potency within this series of compounds, we once again turned our attention to the pyridone ring occupying the S1’ pocket. Methylation of the pyridone did indeed achieve the goal of increased aqueous solubility, but this came at a significant cost in loss of potency. We hypothesized that this could at least in part be attributable to hindered rotation about the biaryl axis (Figure 2), a problem which would be overcome using a symmetrical motif to replace the pyridone. Given that the pyridone forms two important electrostatic interactions (Figure 5), the choice of a symmetrical isostere was somewhat limited. In the end we opted to try the hydroxypyrimidinone structure, which although not having an obvious plane of symmetry, can achieve this by tautomerization.

**Figure 5:**
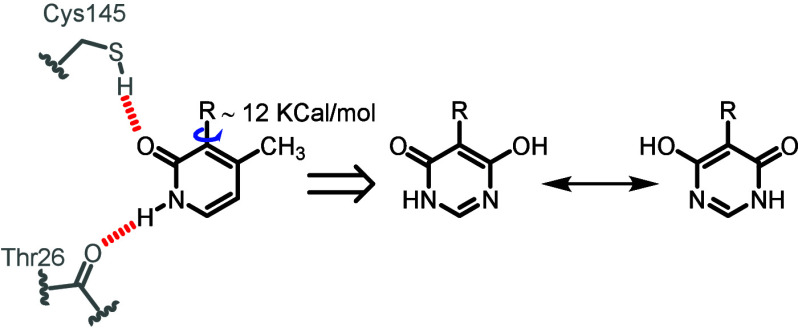
Switching from the methylpyridone to the hydroxypyrimidinone functionality would not only achieve improved aqueous solubility but would also create a plane of symmetry through the ring (by tautomerization), possibly leading to improved binding opportunities within the S1’ pocket.

The synthesis of these anlogs followed a very similar route to that described in Schemes 1 and 2, with the only major difference being the initial Suzuki coupling, which utilized (4,6-dimethoxypyrimidin-5-yl)­boronic acid (SI Scheme S3) in the first step. Interestingly, in the last step of the sequence where we attempted a double demethylation reaction to finally reveal the key hydroxypyrimidinone functionality, we obtained both the singly and doubly demethylated adducts in some cases (Figure 6, **45–49**), allowing us to evaluate both functionalities. The singly demethylated adducts would not have the desired plane of symmetry, but would have a slightly lower barrier to rotation when compared to their methyl counterparts (E.g comparing **48** and **4**). As expected, the aqueous solubility of these polar compounds (**45–47**) was high (>300 μM in PBS buffer) but unfortunately their potencies against SARS-CoV-2 M^pro^ were poor. Interestingly, in the two cases where we inadvertently also obtained the singly demethylated adducts (**48** and **49**), these compounds exhibited an order of magnitude improvement in potency compared to their doubly demethylated counterparts and exhibited good solubility. Unfortunately, **48** demonstrated high clearance (Cl_int_ = 42 mL/min/kg) in our in vitro human liver microsome assay.

**Figure 6:**
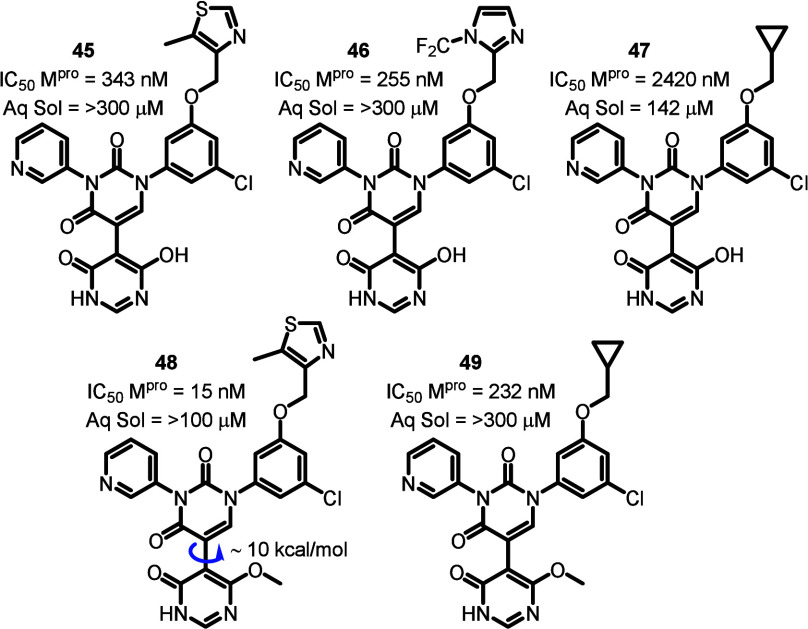
Modification of the pyridone occupying the S1’ pocket to hydroxypyrimidinones resulted in a significant decrease in potency, albeit with vastly improved kinetic aqueous solubility in PBS buffer (Aq Sol).

In summary, in an effort to improve upon the compounds published in our previous work, we attempted to increase the solubility and metabolic stability by modifications to the functionalities occupying the S1, S1’, and S2 pockets. Unfortunately, although for the most part these efforts addressed the issue of solubility, they resulted in an unacceptable loss in potency and/or stability compared to the parent compounds. At this point it became apparent that if we wished to continue with this project, a substantial redesign of the scaffold would be required, and we decided to focus on other projects given our limited synthesis resources. Interestingly, Shionogi, who also developed a competitive, nonpeptidic inhibitor of the SARS-CoV-2 M^pro^ in 2022 (S-217622, Figure 7),^13^ decided on the opposite strategy. After a significant redesign of this compound, including the incorporation of a nitrile covalent warhead (similar to nirmatrelvir), they recently developed S-892216.^14^ This reversibly covalent inhibitor of the SARS-CoV-2 M^pro^ exhibits significantly improved potencies in the biochemical 3CL^pro^ assay and the cellular antiviral assay.

**Figure 7:**
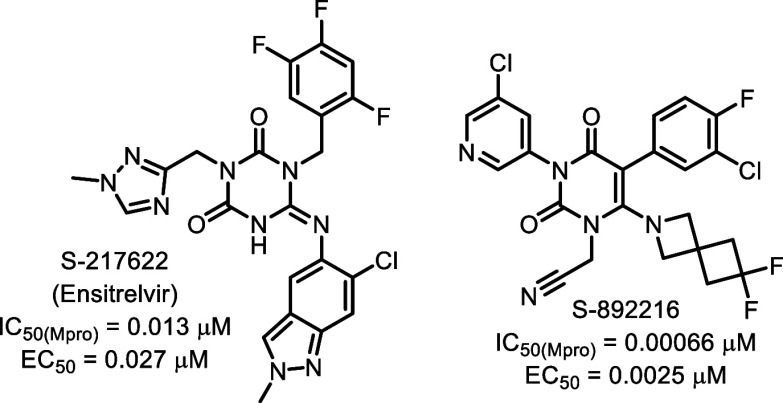
Shionogi’s first-generation SARS-CoV-2 M^pro^ inhibitor (S-217622), and their 2nd generation reversibly covalent inhibitor (S-892216). The 2nd generation compound exhibits excellent potency in their SARS-CoV-2 M^pro^ biochemical assay (IC_50_) and their cellular antiviral assay (EC_50_).

## Supplementary Material



## Notes

All experimental and modeling procedures (PDF)

## Abbreviations

SARS-CoV-2: Severe acute respiratory coronavirus-2
M^pro^: main protease
FEP: free energy perturbation
TMSCl: trimethylsilyl chloride
DIAD: diisopropyl azodicarboxylate
TEMPO: 2,2,6,6-tetramethylpiperidine-1-oxyl
IC_50_: half maximal inhibitory concentration
EC_50_: half maximal effective concentration
DMSO: dimethyl sulfoxide
DMF: dimethylformamide
DME: dimethoxyethane
TMEDA: tetramethylethylenediamine
CC_50_: concentration of test compound required to reduce cell viability by 50%
