# Supplementary material for: Novel Competitive, Nonpeptidic, SARS-CoV‑2 Mpro Inhibitors with Improved Solubility
Source: ACS Med Chem Lett. 2026 May 6;17(5):1132–7. doi: 10.1021/acsmedchemlett.6c00041 (PMC13181476; doi:10.1021/acsmedchemlett.6c00041)
Supplement: Supplementary file 1 [file ml6c00041_si_001.pdf]

Supplementary Information

Novel competitive, non-peptidic, SARS-CoV-2 M^{pro} inhibitors with improved solubility

Zafer Sahin[†], Mario Rivera[†], Yanli Yang[†], Ken Liu[†], Goknil P.C. Sahin[§], John Bacsa[†], Stephen C. Pelly[†]* and Dennis C. Liotta[†]*.

[†] Department of Chemistry, Emory University College of Arts and Sciences, Atlanta, GA, USA.

[§] Department of Pharmaceutical Chemistry, Faculty of Pharmacy, Acibadem Mehmet Ali Aydinlar University, 34752 Istan-bul, Türkiye.

Table of Contents

General 5

Synthesis of Compounds 7-20 6

2,4-Bis(benzyloxy)-5-(2-methoxy-4-methylpyridin-3-yl)pyrimidine (7) 6

5-(2-Methoxy-4-methylpyridin-3-yl)pyrimidine-2,4(1H,3H)-dione (8) 6

1-(3-(Benzyloxy)-5-chlorophenyl)-5-(2-methoxy-4-methylpyridin-3-yl)pyrimidine-2,4(1H,3H)-dione (10) 7

1-(3-(Benzyloxy)-5-chlorophenyl)-5-(2-methoxy-4-methylpyridin-3-yl)-3-(pyridin-3-yl)pyrimidine-2,4(1H,3H)-dione (12) 8

1-(3-Chloro-5-hydroxyphenyl)-5-(2-methoxy-4-methylpyridin-3-yl)-3-(pyridin-3-yl)pyrimidine-2,4(1H,3H)-dione (13) 8

1-(3-Chloro-5-((5-methylthiazol-4-yl)methoxy)phenyl)-5-(2-methoxy-4-methylpyridin-3-yl)-3-(pyridin-3-yl)pyrimidine-2,4(1H,3H)-dione (14) 9

1-(3-Chloro-5-((2,4-dimethylthiazol-5-yl)methoxy)phenyl)-5-(2-methoxy-4-methylpyridin-3-yl)-3-(pyridin-3-yl)pyrimidine-2,4(1*H*,3*H*)-dione (15) 10

1-(3-Chloro-5-((1-(difluoromethyl)-1*H*-imidazol-2-yl)methoxy)phenyl)-5-(2-methoxy-4-methylpyridin-3-yl)-3-(pyridin-3-yl)pyrimidine-2,4(1*H*,3*H*)-dione (16) 10

1-(3-Chloro-5-(cyclopropylmethoxy)phenyl)-5-(2-methoxy-4-methylpyridin-3-yl)-3-(pyridin-3-yl)pyrimidine-2,4(1*H*,3*H*)-dione (17) 11

1-(3-Chloro-5-((5-methylthiazol-4-yl)methoxy)phenyl)-5-(4-methyl-2-oxo-1,2-dihydropyridin-3-yl)-3-(pyridin-3-yl)pyrimidine-2,4(1H,3H)-dione (4) 11

1-(3-Chloro-5-((2,4-dimethylthiazol-5-yl)methoxy)phenyl)-5-(4-methyl-2-oxo-1,2-dihydropyridin-3-yl)-3-(pyridin-3-yl)pyrimidine-2,4(1*H*,3*H*)-dione (18) 12

1-(3-Chloro-5-((1-(difluoromethyl)-1*H*-imidazol-2-yl)methoxy)phenyl)-5-(4-methyl-2-oxo-1,2-dihydropyridin-3-yl)-3-(pyridin-3-yl)pyrimidine-2,4(1*H*,3*H*)-dione (19) 13

1-(3-Chloro-5-(cyclopropylmethoxy)phenyl)-5-(4-methyl-2-oxo-1,2-dihydropyridin-3-yl)-3-(pyridin-3-yl)pyrimidine-2,4(1*H*,3*H*)-dione (20) 14

Scheme S1. Synthesis of compounds 21-23 15

1-(3-((2,4-Dimethylthiazol-5-yl)methoxy)phenyl)-5-(2-methoxy-4-methylpyridin-3-yl)-3-(pyridin-3-yl)pyrimidine-2,4(1*H*,3*H*)-dione (S2) 15

1-(3-((1-(Difluoromethyl)-1*H*-imidazol-2-yl)methoxy)phenyl)-5-(2-methoxy-4-methylpyridin-3-yl)-3-(pyridin-3-yl)pyrimidine-2,4(1*H*,3*H*)-dione (S3) 16

1-(3-(Cyclopropylmethoxy)phenyl)-5-(2-methoxy-4-methylpyridin-3-yl)-3-(pyridin-3-yl)pyrimidine-2,4(1*H*,3*H*)-dione (S4) 16

1-(3-((2,4-Dimethylthiazol-5-yl)methoxy)phenyl)-5-(4-methyl-2-oxo-1,2-dihydropyridin-3-yl)-3-(pyridin-3-yl)pyrimidine-2,4(1*H*,3*H*)-dione (21)....17
1-(3-((1-(Difluoromethyl)-1*H*-imidazol-2-yl)methoxy)phenyl)-5-(4-methyl-2-oxo-1,2-dihydropyridin-3-yl)-3-(pyridin-3-yl)pyrimidine-2,4(1*H*,3*H*)-dione (22)....17
1-(3-(Cyclopropylmethoxy)phenyl)-5-(4-methyl-2-oxo-1,2-dihydropyridin-3-yl)-3-(pyridin-3-yl)pyrimidine-2,4(1*H*,3*H*)-dione (23)....18
2,4-Bis(benzyloxy)-5-(2-methoxypyridin-3-yl)pyrimidine (25)....19
5-(2-Methoxypyridin-3-yl)pyrimidine-2,4(1H,3H)-dione (26)....20
3-(Cyclopropylmethoxy)-1-fluoro-5-iodo-benzene (27)....20
1-Bromo-3-(cyclopropylmethoxy)-5-iodo-benzene (28)....21
1-Chloro-3-(cyclopropylmethoxy)-2-flouro-5-iodo-benzene (29)....21
1-[3-(Cyclopropylmethoxy)-5-flourophenyl]-5-(2-methoxy-3-pyridyl)pyrimide-2,4-dione (30)....22
1-[3-Bromo-5-(cyclopropylmethoxy)phenyl]-5-(2-methoxy-3-pyridyl)pyrimide-2,4-dione (31)....22
1-[3-Chloro-5-(cyclopropylmethoxy)-4-flourophenyl]-5-(2-methoxy-3-pyridyl)pyrimide-2,4-dione (32)....23
1-[3-(Cyclopropylmethoxy)-5-flourophenyl]-5-(2-methoxy-3-pyridyl)-3-(3-pyridyl)pyrimidine-2,4-dione (33)....23
1-[3-Bromo-5-(cyclopropylmethoxy)phenyl]-5-(2-methoxy-3-pyridyl)-3-(3-pyridyl)pyrimidine-2,4-dione (34)....24
1-[3-Chloro-5-(cyclopropylmethoxy)-4-flourophenyl]-5-(2-methoxy-3-pyridyl)-3-(3-pyridyl)pyrimidine-2,4-dione (35)....25
1-[3-(Cyclopropylmethoxy)-5-flourophenyl]-5-(2-oxo-1H-pyridin-3-yl)-3-(3-pyridyl)pyrimidine-2,4-dione (36)....25
1-[3-Bromo-5-(cyclopropylmethoxy)phenyl]-5-(2-oxo-1H-pyridin-3-yl)-3-(3-pyridyl)pyrimidine-2,4-dione (37)....26
1-[3-Chloro-5-(cyclopropylmethoxy)-4-flourophenyl]-5-(2-oxo-1H-pyridin-3-yl)-3-(3-pyridyl)pyrimidine-2,4-dione (38)....27

Scheme S2. Synthesis of compound 41-44 28

1-(3-(Benzyloxy)-5-chlorophenyl)-5-(2-methoxypyridin-3-yl)pyrimidine-2,4(1*H*,3*H*)-dione (S5)....28
1-(3-(Benzyloxy)-5-chlorophenyl)-5-(2-methoxypyridin-3-yl)-3-(pyridazin-3-yl)pyrimidine-2,4(1*H*,3*H*)-dione (S6)....29
1-(3-Chloro-5-hydroxyphenyl)-5-(2-methoxypyridin-3-yl)-3-(pyridazin-3-yl)pyrimidine-2,4(1*H*,3*H*)-dione (S7)....30
1-(3-Chloro-5-((5-methylthiazol-4-yl)methoxy)phenyl)-5-(2-methoxypyridin-3-yl)-3-(pyridazin-3-yl)pyrimidine-2,4(1*H*,3*H*)-dione (S8)....30
1-(3-Chloro-5-((1-(difluoromethyl)-1*H*-imidazol-2-yl)methoxy)phenyl)-5-(2-methoxypyridin-3-yl)-3-(pyridazin-3-yl)pyrimidine-2,4(1*H*,3*H*)-dione (S9)....31
1-(3-Chloro-5-(cyclopropylmethoxy)phenyl)-5-(2-methoxypyridin-3-yl)-3-(pyridazin-3-yl)pyrimidine-2,4(1*H*,3*H*)-dione (S10)....31
1-(3-Chloro-5-(cyclopropylmethoxy)phenyl)-5-(2-methoxypyridin-3-yl)-3-(pyridazin-3-yl)pyrimidine-2,4(1*H*,3*H*)-dione (42)....32
1-(3-Chloro-5-((1-(difluoromethyl)-1*H*-imidazol-2-yl)methoxy)phenyl)-5-(2-oxo-1,2-dihydropyridin-3-yl)-3-(pyridazin-3-yl)pyrimidine-2,4(1*H*,3*H*)-dione (43)....33
1-(3-Chloro-5-(cyclopropylmethoxy)phenyl)-5-(2-oxo-1,2-dihydropyridin-3-yl)-3-(pyridazin-3-yl)pyrimidine-2,4(1*H*,3*H*)-dione (44)....33
3-(3-(Benzyloxy)-5-chlorophenyl)-5-(2-methoxypyridin-3-yl)-2H-[1,5'-bipyrimidine]-2,6(3H)-dione (S11)....34
3-(3-Chloro-5-hydroxyphenyl)-5-(2-methoxypyridin-3-yl)-2H-[1,5'-bipyrimidine]-2,6(3H)-dione (S12)....35
3-(3-Chloro-5-((5-methylthiazol-4-yl)methoxy)phenyl)-5-(2-methoxypyridin-3-yl)-2H-[1,5'-bipyrimidine]-2,6(3H)-dione (S13)....35
3-(3-Chloro-5-((5-methylthiazol-4-yl)methoxy)phenyl)-5-(2-oxo-1,2-dihydropyridin-3-yl)-2H-[1,5'-bipyrimidine]-2,6(3H)-dione (41)....36

Scheme S3. Synthesis of compounds 45-49 37

2,4-Bis(benzyloxy)-4',6'-dimethoxy-5,5'-bipyrimidine (S15)....37

4',6'-Dimethoxy-[5,5'-bipyrimidine]-2,4(1*H*,3*H*)-dione (S16) 38
1-(3-(Benzyloxy)-5-chlorophenyl)-4',6'-dimethoxy-[5,5'-bipyrimidine]-2,4(1*H*,3*H*)-dione (S17) 38
1-(3-(Benzyloxy)-5-chlorophenyl)-4',6'-dimethoxy-3-(pyridin-3-yl)-[5,5'-bipyrimidine]-2,4(1*H*,3*H*)-dione (S18) 39
1-(3-Chloro-5-hydroxyphenyl)-4',6'-dimethoxy-3-(pyridin-3-yl)-[5,5'-bipyrimidine]-2,4(1*H*,3*H*)-dione (S19) 40
1-(3-Chloro-5-((5-methylthiazol-4-yl)methoxy)phenyl)-4',6'-dimethoxy-3-(pyridin-3-yl)-[5,5'-bipyrimidine]-2,4(1*H*,3*H*)-dione (S20) 40
1-(3-Chloro-5-((1-(difluoromethyl)-1*H*-imidazol-2-yl)methoxy)phenyl)-4',6'-dimethoxy-3-(pyridin-3-yl)-[5,5'-bipyrimidine]-2,4(1*H*,3*H*)-dione (S21) 41
1-(3-Chloro-5-(cyclopropylmethoxy)phenyl)-4',6'-dimethoxy-3-(pyridin-3-yl)-[5,5'-bipyrimidine]-2,4(1*H*,3*H*)-dione (S22) 41
1-(3-Chloro-5-((5-methylthiazol-4-yl)methoxy)phenyl)-6'-hydroxy-3-(pyridin-3-yl)-[5,5'-bipyrimidine]-2,4,4'(1*H*,3*H*,3'*H*)-trione (45) 42
1-(3-Chloro-5-((1-(difluoromethyl)-1*H*-imidazol-2-yl)methoxy)phenyl)-6'-hydroxy-3-(pyridin-3-yl)-[5,5'-bipyrimidine]-2,4,4'(1*H*,3*H*,3'*H*)-trione (46) 43
1-(3-Chloro-5-(cyclopropylmethoxy)phenyl)-6'-hydroxy-3-(pyridin-3-yl)-[5,5'-bipyrimidine]-2,4,4'(1*H*,3*H*,3'*H*)-trione (47) 44
1-(3-Chloro-5-((5-methylthiazol-4-yl)methoxy)phenyl)-6'-methoxy-3-(pyridin-3-yl)-[5,5'-bipyrimidine]-2,4,4'(1*H*,3*H*,3'*H*)-trione (48) 44
1-(3-Chloro-5-(cyclopropylmethoxy)phenyl)-6'-methoxy-3-(pyridin-3-yl)-[5,5'-bipyrimidine]-2,4,4'(1*H*,3*H*,3'*H*)-trione (49) 45

Molecular Modelling: 46

SARS Cov-2 Main Protease Enzymatic (IC_{50}) Assay Protocol 47

Kinetic solubility 48
Table S1. Aqueous Solubility of the tested compounds 48
Compound 19: 144.1 μM 50
Compound 20: >300 μM 51
Compound 37: 110.2 μM 52
Compound 38: 23 μM 52
Compound 42: 143.5 μM 53
Compound 43: >300 μM 53
Compound 45: >300 μM 54
Compound 46: >300 μM 54
Compound 47: 142.1 μM 55
Compound 48: >100 μM 55
Compound 49: >300 μM 56

Liver Microsome Stability Assay Protocol 56

Appendix: 58
^{1}H NMR spectrum of **7** 58
^{1}H NMR spectrum of **8** 59
^{1}H NMR spectrum of **10** 59
^{1}H NMR spectrum of **12** 60
^{1}H NMR spectrum of **13** 60
^{1}H NMR spectrum of **14** 61
^{1}H NMR spectrum of **15** 61
^{1}H and ^{19}F NMR spectrum of **16** 62
^{1}H NMR spectrum of **17** 63
^{1}H and ^{13}C NMR spectrum of **4** 64
^{1}H and ^{13}C NMR spectrum of **18** 65
^{1}H, ^{19}F and ^{13}C NMR spectrum of **19** 66
^{1}H and ^{13}C NMR spectrum of **20** 68
^{1}H NMR spectrum of **S2** 69

^{1}H and ^{19}F NMR spectrum of **S3** 70
^{1}H and ^{13}C NMR spectrum of **S4** 71
^{1}H and ^{13}C NMR spectrum of **21** 72
^{1}H, ^{19}F and ^{13}C NMR spectrum of **22** 73
^{1}H and ^{13}C NMR spectrum of **23** 75
^{1}H NMR spectrum of **26** 76
^{1}H and ^{19}F NMR spectrum of **27** 77
^{1}H NMR spectrum of **28** 78
^{1}H and ^{19}F NMR spectrum of **29** 78
^{1}H and ^{19}F NMR spectrum of **30** 79
^{1}H and ^{13}C NMR spectrum of **31** 80
^{1}H and ^{19}F spectrum of **32** 81
^{1}H and ^{19}F NMR spectrum of **33** 82
^{1}H NMR spectrum of **34** 83
^{1}H, ^{13}C and ^{19}F NMR spectrum of **36** 83
^{1}H, ^{13}C NMR spectrum of **37** 85
^{1}H, ^{13}C and ^{19}F NMR spectrum of **38** 86
^{1}H NMR spectrum of **S5** 87
^{1}H NMR spectrum of **S6** 88
^{1}H NMR spectrum of **S8** 89
^{1}H and ^{19}F NMR spectrum of **S9** 90
^{1}H NMR spectrum of **S10** 91
^{1}H NMR spectrum of **S11** 91
^{1}H NMR spectrum of **S12** 92
^{1}H NMR spectrum of **S13** 92
^{1}H and ^{13}C NMR spectrum of **41** 93
^{1}H and ^{13}C NMR spectrum of **42** 94
^{1}H, ^{19}F and ^{13}C NMR spectrum of **43** 95
^{1}H and ^{13}C NMR spectrum of **44** 96
^{1}H NMR spectrum of **S15** 98
^{1}H NMR spectrum of **S16** 98
^{1}H NMR spectrum of **S17** 99
^{1}H NMR spectrum of **S18** 99
^{1}H NMR spectrum of **S19** 100
^{1}H NMR spectrum of **S20** 100
^{1}H and ^{19}F NMR spectrum of **S21** 101
^{1}H NMR spectrum of **S22** 102
^{1}H and ^{13}C NMR spectrum of **45** 103
^{1}H, ^{19}F and ^{13}C NMR spectrum of **46** 105
^{1}H and ^{13}C NMR spectrum of **47** 107
^{1}H and ^{13}C NMR spectrum of **48** 108
^{1}H and ^{13}C NMR spectrum of **49** 109

HRMS Spectra **110**
HRMS spectra of compound **4** 110
HRMS spectra of compound **18** 111
HRMS spectra of compound **19** 113
HRMS spectra of compound **20** 115
HRMS spectra of compound **21** 117
HRMS spectra of compound **22** 118
HRMS spectra of compound **23** 119
HRMS spectra of compound **36** 120
HRMS spectra of compound **37** 121
HRMS spectra of compound **38** 124
HRMS spectra of compound **41** 127
HRMS spectra of compound **42** 128

HRMS spectra of compound **43** 130
HRMS spectrum of compound **44** 133
HRMS spectrum of compound **45** 134
HRMS spectrum of compound **46** 136
HRMS spectrum of compound **47** 137
HRMS spectrum of compound **48** 139
HRMS spectrum of compound **49** 140

X-ray crystallography 142
Compound 10 142

General

Unless otherwise indicated, all reactions requiring anhydrous conditions were conducted in oven dried or flame-dried glassware using distilled and degassed solvents under a positive pressure of dry argon. General chemicals and reagents were obtained from commercial suppliers and were used without further purification. Typically, reaction progress was monitored by either thin layer chromatography (TLC) using silica-precoated glass plates (Merck KGaA; silica gel 60 F_{254}, 0.25 mm thickness) or liquid chromatography-mass spectrometry (LC-MS) on an Agilent Technologies 6100 quadrupole instrument equipped with UV detection at 254 and 210 nm and Agilent C18 XDB eclipse column (50 mm x 4.6 mm, 3.5 µM). Automated flash column chromatography was performed using Teledyne ISCO CombiFlash Companion systems with silica gel-packed columns or RediSepRf reverse-phase C18 gold columns (Teledyne Isco). NMR spectra (^{1}H, ^{13}C, and ^{19}F) were obtained using either a Varian INOVA 600 MHz spectrometer, a Varian INOVA 500 MHz spectrometer, a Varian INOVA 400 MHz spectrometer, a Varian VNMR 400 MHz spectrometer, a Bruker 400 MHz spectrometer, or a Bruker 600 MHz spectrometer. NMR samples were prepared and processed in deuterated chloroform ($CDCl_3$, residual solvent peaks: ^{1}H = 7.26 ppm, ^{13}C = 77.16 ppm) or deuterated methanol (CD_3OD, residual solvent peaks: ^{1}H = 3.31 ppm, ^{13}C = 49.0 ppm) or deuterated DMSO (DMSO, residual solvent peaks: ^{1}H = 2.50 ppm, ^{13}C = 39.52 ppm). The residual chloroform (or TMS if present) or methanol or H_2O peak in ^{1}H NMR was used as an absolute reference for ^{19}F NMR. NMR data were reported to include chemical shifts (δ) reported in ppm, multiplicities indicated as s (singlet), d (doublet), t (triplet), q (quartet), p (pentet), hept (heptet), td (triplet of doublets), m (multiplet), br (broad), coupling constants (*J*) reported in Hz, and integration normalized to 1 atom (H, C, or P). High resolution mass spectrometry (HRMS) was performed by the Emory University Mass Spectrometry Center, directed by Dr. Fred Strobel. Liquid chromatography-mass spectrometry (LC-MS) was performed on an Agilent 1200 HPLC equipped with a 6120 Quadrupole mass spectrometer (ESI-API) eluting with mixtures of HPLC grade MeOH and H_2O (0.1% formic acid) or MeCN and H_2O (0.1% formic acid) using an analytical, reverse-phase, Agilent C18 XDB eclipse column (50 mm x 4.6 mm, 3.5 µM). LC-MS samples were prepared in aqueous solutions of MeOH, or pure MeOH. Final compound purity was assessed using NMR and LC-MS, and the purity of all final compounds reported herein were determined to be ≥95% pure.

Synthesis of Compounds 7-20

2,4-Bis(benzyloxy)-5-(2-methoxy-4-methylpyridin-3-yl)pyrimidine (7)

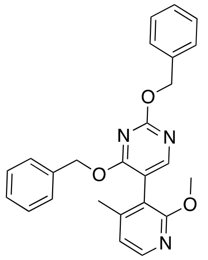

Into a 500 mL three-neck round bottom flask fitted with a condenser and filled with argon, was placed 3-bromo-2-methoxy-4-methyl-pyridine (4.50 mL, 35.3 mmol) followed by monoglyme (200 mL), thus forming a clear solution. To this, (2,4-dibenzyloxypyrimidin-5-yl)boronic acid (13.7 g, 40.7 mmol) was added, which dissolved quickly. A saturated solution of sodium bicarbonate (212 mL) was then added, resulting in the immediate formation of a white precipitate. The solution was then degassed for 10 minutes by bubbling argon into the mixture and then tetrakis(triphenylphosphine)palladium(0) (3.92 g, 3.39 mmol) was added in one portion. The reaction mixture was heated to reflux, resulting in the dissolution of most of the white precipitates and the formation of a yellow solution. The reaction was left to proceed for 6 hours at reflux and the color changed to a darker yellow/orange. After this time, the mixture was cooled, poured into a separating funnel, and diluted with EtOAc (300 mL) and water (200 mL). After vigorously shaking, the layers were allowed to separate, and the organic layer was collected. The aqueous layer was extracted twice more with EtOAc (2 x 200 ml). The organic fractions were combined and dried over anhydrous magnesium sulfate and then filtered through a plug of silica gel. The silica bed was washed with ethyl acetate. The organic solution was then concentrated in vacuo and the crude material purified by column chromatography (EtOAc/Hex) to afford 2,4-dibenzyloxy-5-(2-methoxy-4-methyl-3-pyridyl)pyrimidine (12.7 g, 30.6 mmol, 90 % yield).

^{1}H NMR (400 MHz, MeOD) δ 8.09 (s, 1H), 8.01 (d, *J* = 5.3 Hz, 1H), 7.52 - 7.45 (m, 2H), 7.44 - 7.35 (m, 3H), 7.34 - 7.24 (m, 5H), 6.91 (d, *J* = 5.3 Hz, 1H), 5.54 - 5.38 (m, 4H), 3.79 (s, 3H), 2.08 (s, 3H).

5-(2-Methoxy-4-methylpyridin-3-yl)pyrimidine-2,4(1H,3H)-dione (8)

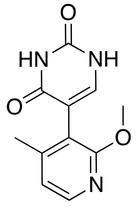

Into a three-neck 1L round-bottom flask containing methanol (300 mL) and THF (300 mL) was placed 2,4-dibenzyloxy-5-(2-methoxy-4-methyl-3-pyridyl)pyrimidine (4.00 g, 9.67 mmol), resulting in a clear solution. The flask was purged with argon, after which Pd/C (1.03 g, 9.67 mmol) was added in one portion. A balloon filled with hydrogen was fitted to the flask, and the system was subsequently purged with hydrogen. The reaction was allowed to proceed for 36 h at 45 °C, after which LC-MS analysis confirmed complete consumption of the starting material and formation of the desired product. The reaction mixture was

filtered through a pad of Celite and concentrated in vacuo to afford 5-(2-methoxy-4-methyl-3-pyridyl)-1H-pyrimidine-2,4-dione (2.03 g, 8.70 mmol, 90% yield) as a white solid. (Do not attempt purification by column chromatography; the compound precipitates on the column and causes significant operational difficulties.)

^{1}H NMR (600 MHz, DMSO-*d6*) δ 11.18 (s, 1H), 11.04 (s, 1H), 7.96 (dd, *J* = 5.1, 0.8 Hz, 1H), 7.35 (s, 1H), 6.85 (dd, *J* = 5.1, 0.8 Hz, 1H), 3.72 (s, 3H), 2.05 (s, 3H).

1-(3-(Benzyloxy)-5-chlorophenyl)-5-(2-methoxy-4-methylpyridin-3-yl)pyrimidine-2,4(1H,3H)-dione (10)

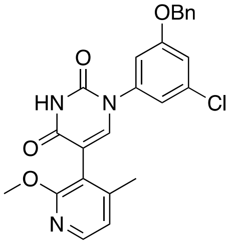

Into a two-neck round-bottom flask under an argon atmosphere was added DMSO (70 mL), followed by 5-(2-methoxy-4-methyl-3-pyridyl)-1H-pyrimidine-2,4-dione (2.00 g, 8.58 mmol), 1-benzyloxy-3-Chloro-5-iodobenzene (3.55 g, 10.3 mmol), N-(2-cyanophenyl)picolinamide (0.230 g, 1.03 mmol), and potassium phosphate (3.82 g, 18.0 mmol), forming a brown suspension. The mixture was degassed by bubbling argon through the solution for 10 min, after which copper(I) iodide (164 mg, 0.860 mmol) was added under a flow of argon. The reaction mixture was heated to 60 °C, during which the mixture turned very dark green, and was stirred under argon at this temperature for three days. The reaction mixture was then diluted with EtOAc (200 mL) and water (500 mL). A suspension formed upon mixing, which was broken by pulling the entire mixture through a pad of celite. The organic layer was separated, and the aqueous layer was extracted twice with EtOAc (2 × 200 mL). The combined organic layers were dried over anhydrous magnesium sulfate and filtered. The solvent was removed in vacuo, and DCM was added to wet the residue. Hexanes were then added to precipitate the majority of the product (2.20 g). The solid exhibited a green coloration, which was removed by washing with methanol. The material was dried on filter paper overnight, and NMR analysis was performed after evaporation of residual hexanes and methanol. A solid-phase cartridge was prepared, and the crude material was purified by column chromatography (EtOAc/hex) to afford 1-(3-benzyloxy-5-chloro-phenyl)-5-(2-methoxy-4-methyl-3-pyridyl)pyrimidine-2,4-dione (2.00 g, 4.45 mmol, 52% yield).

^{1}H NMR (400 MHz, MeOD) δ 8.01 (d, *J* = 5.3 Hz, 1H), 7.60 (s, 1H), 7.47 - 7.42 (m, 2H), 7.41 - 7.29 (m, 3H), 7.13 (d, *J* = 3.1, 2H), 7.08 (t, *J* = 2.1 Hz, 1H), 6.92 (dd, *J* = 5.3, 0.6 Hz, 1H), 5.13 (s, 2H), 3.90 (s, 3H), 2.27 (s, 3H).

1-(3-(Benzyloxy)-5-chlorophenyl)-5-(2-methoxy-4-methylpyridin-3-yl)-3-(pyridin-3-yl)pyrimidine-2,4(1H,3H)-dione (12)

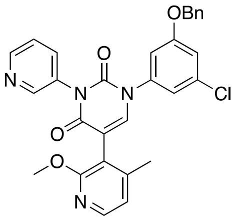

Into a 500 mL round-bottom flask containing 1-(3-benzyloxy-5-chloro-phenyl)-5-(2-methoxy-4-methyl-3-pyridyl)pyrimidine-2,4-dione (2.00 g, 4.45 mmol), were added 3-pyridylboronic acid (1.64 g, 13.3 mmol), copper(II) acetate (1.21 g, 6.67 mmol), DMSO (20 mL) and finally TMEDA (1.50 mL, 10.0 mmol), forming a deep blue solution. The reaction mixture was stirred open to the atmosphere at 60 °C for 18 h, after which LC-MS analysis indicated complete conversion of the starting material to the desired product. The reaction mixture was diluted with EtOAc (200 mL) and water (300 mL) and mixed vigorously. After phase separation, the aqueous layer was extracted twice with EtOAc (2 × 200 mL). The combined organic extracts were dried over anhydrous magnesium sulfate, filtered, and concentrated in vacuo to afford 1-(3-benzyloxy-5-chloro-phenyl)-5-(2-methoxy-4-methyl-3-pyridyl)-3-(3-pyridyl)pyrimidine-2,4-dione (2.21 g, 4.20 mmol, 94% yield).

^{1}H NMR (400 MHz, MeOD) δ 8.64 - 8.58 (m, 2H), 8.02 (dd, *J* = 5.9, 1.8 Hz, 1H), 7.92 (dd, *J* = 8.1, 2.5, 1H), 7.80 - 7.75 (m, 1H), 7.65 - 7.57 (m, 1H), 7.50 - 7.42 (m, 2H), 7.40 - 7.34 (m, 2H), 7.30 - 7.25 (m, 1H), 7.22 (t, *J* = 1.8 Hz, 1H), 7.21-7.17 (m, 2H), 6.96 - 6.90 (m, 1H), 5.14 (s, 2H), 3.91 (d, *J* = 0.7 Hz, 3H), 2.32 (s, 3H).

1-(3-Chloro-5-hydroxyphenyl)-5-(2-methoxy-4-methylpyridin-3-yl)-3-(pyridin-3-yl)pyrimidine-2,4(1H,3H)-dione (13)

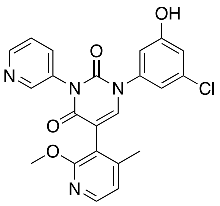

and

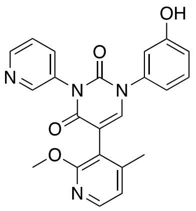

13 S1

Into a two-neck round-bottom flask under an argon atmosphere was placed 1-(3-benzyloxy-5-chloro-phenyl)-5-(2-methoxy-4-methyl-3-pyridyl)-3-(3-pyridyl)pyrimidine-2,4-dione (2.23 g, 4.23 mmol), followed by methanol (15 mL) and THF (15 mL). Upon heating, the starting material solubilized and remained in solution after cooling. Pd/C (0.45 g, 0.42 mmol) was then added, and a balloon filled with hydrogen was fitted to the flask. The atmosphere in the flask was purged with hydrogen, and the reaction was allowed to proceed at 40 °C. After three hours, LC–MS analysis indicated complete consumption of the starting material. The reaction mixture was filtered through a pad of celite and concentrated in vacuo

to afford 1-(3-Chloro-5-hydroxy-phenyl)-5-(2-methoxy-4-methyl-3-pyridyl)-3-(3-pyridyl)pyrimidine-2,4-dione (1.80 g, 4.12 mmol, 97% yield) as a white solid, which was used without further purification.

^{1}H NMR (400 MHz, MeOD) δ 8.63 - 8.57 (m, 2H), 8.01 (d, *J* = 5.3 Hz, 1H), 7.95 - 7.87 (m, 1H), 7.79 - 7.72 (m, 1H), 7.64 - 7.55 (m, 1H), 7.37 - 7.29 (m, 1H), 7.09 - 6.95 (m, 2H), 6.93-6.88 (m, 2H), 3.91 (s, 3H), 2.32 (d, *J* = 1.9 Hz, 3H).

2 tubes of clean compound **13** was isolated for NMR. It was not convenient to isolate compound **13** and **S1** completely clean. Thus, all the material were carried forward. There is no additional experimental for **S1,** Compounds **S2-S4** purification were achieved after derivatization of phenol group. Related experimental has given in **S2**-**S4**.

1-(3-Chloro-5-((5-methylthiazol-4-yl)methoxy)phenyl)-5-(2-methoxy-4-methylpyridin-3-yl)-3-(pyridin-3-yl)pyrimidine-2,4(1H,3H)-dione (14)

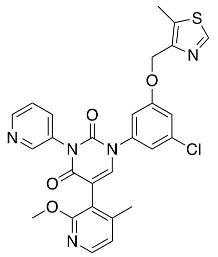

1-(3-Chloro-5-hydroxy-phenyl)-5-(2-methoxy-4-methyl-3-pyridyl)-3-(3-pyridyl)pyrimidine-2,4-dione (0.21 g, 0.48 mmol) was dissolved in DMF (10 mL), and potassium carbonate (200 mg, 1.45 mmol) was added. Immediately afterward, 4-(chloromethyl)-5-methylthiazole hydrochloride (115 mg, 0.620 mmol) was added. The flask was flushed with argon and the mixture was stirred at 60 °C overnight. Upon confirmation of reaction completion by LC–MS and TLC, the mixture was diluted with ethyl acetate and water and extracted. The combined organic layers were dried, filtered, and concentrated in vacuo. The crude product was purified by chromatography using DCM/MeOH (97:3) to afford 180 mg of **14** with 68% yield. The purified compound was analyzed by ^{1}H NMR. (This compound was made from an initial trial batch of **13**, which was performed at room temperature, not 40 °C. Further compounds 15-17 and **S2**-**S4** were made from the new batch of **13**-**S1** mixture as explained above).

^{1}H NMR (400 MHz, MeOD) δ 8.80 (s, 1H), 8.61 (d, *J* = 1.1 Hz, 2H), 8.02 (d, *J* = 5.3 Hz, 1H), 7.97 - 7.89 (m, 1H), 7.82 (d, *J* = 3.8 Hz, 1H), 7.62 (dd, *J* = 8.2, 5.0 Hz, 1H), 7.45 (t, *J* = 8.2 Hz, 1H), 7.29 - 7.18 (m, 1H), 7.18 - 7.10 (m, 1H), 6.93 (d, *J* = 5.3 Hz, 1H), 5.23 (s, 2H), 3.92 (d, *J* = 1.1 Hz, 3H), 2.55 (s, 3H), 2.33 (s, 3H).

1-(3-Chloro-5-((2,4-dimethylthiazol-5-yl)methoxy)phenyl)-5-(2-methoxy-4-methylpyridin-3-yl)-3-(pyridin-3-yl)pyrimidine-2,4(1*H*,3*H*)-dione (15)

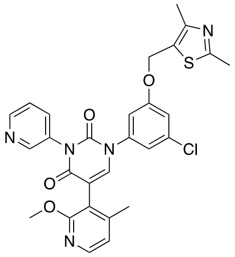

A mixture of 1-(3-Chloro-5-hydroxyphenyl)-5-(2-methoxy-4-methyl-3-pyridyl)-3-(3-pyridyl)pyrimidine-2,4-dione (0.25 g) and 1-(3-hydroxyphenyl)-5-(2-methoxy-4-methyl-3-pyridyl)-3-(3-pyridyl)pyrimidine-2,4-dione (231 mg) was dissolved in DMF (10 mL), and potassium carbonate (237 mg, 1.72 mmol) was added. Immediately afterwards, 5-(chloromethyl)-2,4-dimethylthiazole hydrochloride (147 mg, 0.740 mmol) was added. The flask was flushed with argon, and the reaction mixture was stirred at 60 °C overnight. After confirming completion of the reaction by LC–MS and TLC, the mixture was diluted with ethyl acetate and water and extracted. The combined organic layers were dried, filtered, and concentrated in vacuo. At his point, we were able to separate two compounds (**15** and **S2**). The crude products were first purified by chromatography using DCM/MeOH (97:3), followed by reverse-phase CombiFlash chromatography using water and acetonitrile to afford 70 mg of **15**.

^{1}H NMR (400 MHz, MeOD) δ 8.62 - 8.55 (m, 2H), 8.01 (d, *J* = 5.3 Hz, 1H), 7.89 (dd, *J* = 8.1, 2.5 Hz, 1H), 7.81 (s, 1H), 7.58 (dd, *J* = 8.2, 4.9 Hz, 1H), 7.24 (t, *J* = 1.8 Hz, 1H), 7.16 (t, *J* = 1.9 Hz, 2H), 6.91 (d, *J* = 5.2 Hz, 1H), 5.22 (s, 2H), 3.90 (s, 3H), 2.62 (s, 3H), 2.37 (s, 3H), 2.31 (s, 3H).

1-(3-Chloro-5-((1-(difluoromethyl)-1*H*-imidazol-2-yl)methoxy)phenyl)-5-(2-methoxy-4-methylpyridin-3-yl)-3-(pyridin-3-yl)pyrimidine-2,4(1*H*,3*H*)-dione (16)

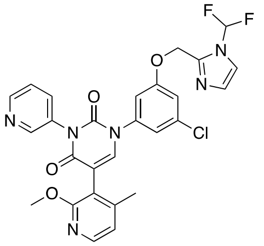

A mixture of 1-(3-Chloro-5-hydroxyphenyl)-5-(2-methoxy-4-methyl-3-pyridyl)-3-(3-pyridyl)pyrimidine-2,4-dione (0.25 g) and 1-(3-hydroxyphenyl)-5-(2-methoxy-4-methyl-3-pyridyl)-3-(3-pyridyl)pyrimidine-2,4-dione (230 mg) was dissolved in DMF (10 mL), and potassium carbonate (237 mg, 1.72 mmol) was added. Immediately afterward, 2-(chloromethyl)-1-(difluoromethyl)imidazole hydrochloride (151 mg, 0.740 mmol) was added. The flask was flushed with argon, and the reaction mixture was stirred at 60 °C overnight. Upon confirming completion of the reaction by LC–MS and TLC, the mixture was diluted with ethyl acetate and water and extracted. The combined organic layers were dried, filtered, and concentrated in vacuo. At his point, we were able to separate two compounds (**16** and **S3**). The crude

product was purified first by chromatography using DCM/MeOH (97:3), followed by reverse-phase CombiFlash purification using water and acetonitrile to afford 110 mg of **16**.

^{1}H NMR (400 MHz, MeOD) δ 8.66 - 8.52 (m, 2H), 8.02 (d, *J* = 5.3 Hz, 1H), 7.93 (d, *J* = 8.2 Hz, 1H), 7.83 (s, 1H), 7.71 (s, 1H), 7.62 (d, *J* = 0.8 Hz, 1H), 7.56 (t, *J* = 1.7 Hz, 1H), 7.30 (t, *J* = 1.8 Hz, 1H), 7.27 - 7.20 (m, 2H), 7.13 (d, *J* = 1.7 Hz, 1H), 6.93 (d, *J* = 0.7 Hz, 1H), 5.36 (s, 2H), 3.92 (s, 3H), 2.33 (s, 3H). **^{19}F NMR (376 MHz, MeOD)** δ -94.42, -94.57.

1-(3-Chloro-5-(cyclopropylmethoxy)phenyl)-5-(2-methoxy-4-methylpyridin-3-yl)-3-(pyridin-3-yl)pyrimidine-2,4(1*H*,3*H*)-dione (17)

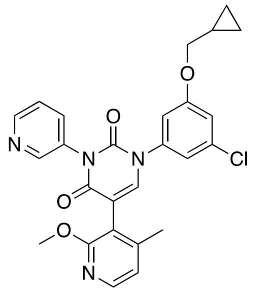

1-(3-Chloro-5-hydroxyphenyl)-5-(2-methoxy-4-methyl-3-pyridyl)-3-(3-pyridyl)pyrimidine-2,4-dione (0.22 g) was dissolved in DMF (10 mL), and potassium carbonate (209 mg) were added. Immediately afterward, bromomethylcyclopropane (0.10 mL, 0.72 mmol) was added. The flask was flushed with argon, and the mixture was stirred at 60 °C overnight. Upon confirming completion of the reaction by LC–MS and TLC, the mixture was diluted with ethyl acetate and water and extracted. The combined organic layers were dried, filtered, and concentrated in vacuo. At his point, we were able to separate two compounds (**17** and **S4**). The crude product was purified first by chromatography using DCM/MeOH (97:3), followed by reverse-phase CombiFlash purification using water and acetonitrile to afford 80 mg of **17**.

^{1}H NMR (400 MHz, MeOD) δ 8.60 (s, 2H), 8.01 (d, *J* = 5.3 Hz, 1H), 7.91 (dd, *J* = 8.2, 2.5 Hz 1H), 7.79 (s, 1H), 7.64 - 7.56 (m, 1H), 7.17 (t, *J* = 1.8 Hz, 1H), 7.08 - 7.04 (m, 2H), 6.92 (d, *J* = 5.3 Hz, 1H), 3.91 (s, 3H), 3.87 (d, *J* = 7.0 Hz, 2H), 2.32 (s, 3H), 1.32 - 1.19 (m, 1H), 0.68 - 0.57 (m, 2H), 0.42 - 0.32 (m, 2H).

1-(3-Chloro-5-((5-methylthiazol-4-yl)methoxy)phenyl)-5-(4-methyl-2-oxo-1,2-dihydropyridin-3-yl)-3-(pyridin-3-yl)pyrimidine-2,4(1H,3H)-dione (4)

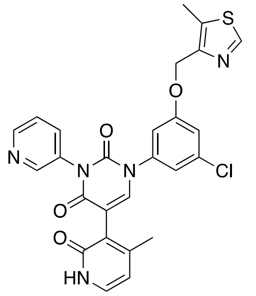

Into a 100 mL two-neck round-bottom flask under an argon atmosphere was placed 1-[3-Chloro-5-[(5-methylthiazol-4-yl)methoxy]phenyl]-5-(2-methoxy-4-methyl-3-pyridyl)-3-(3-pyridyl)pyrimidine-2,4-dione (0.18 g, 0.33 mmol), followed by dry MeCN (10 mL), forming a clear solution. To this solution was added NaI (0.15 g, 0.99 mmol), followed immediately by chlorotrimethylsilane (0.150 mL, 1.14 mmol), resulting in a pale-yellow solution. The reaction mixture was heated to 60 °C for 6 h, after which LC–MS analysis indicated complete consumption of the starting material. After cooling, the reaction mixture was diluted with EtOAc (250 mL), brine (250 mL), and 10% sodium thiosulfate solution (150 mL). The mixture was thoroughly stirred, and the layers were separated. The aqueous layer was extracted with EtOAc (3 × 250 mL). The combined organic layers were washed with brine, dried over anhydrous magnesium sulfate, filtered, and concentrated in vacuo. The crude product was purified by column chromatography (initially EtOAc/hex, followed by 3% MeOH/EtOAc) to afford 1-[3-Chloro-5-[(5-methylthiazol-4-yl)methoxy]phenyl]-5-(4-methyl-2-oxo-1H-pyridin-3-yl)-3-(3-pyridyl)pyrimidine-2,4-dione (0.050 g, 0.10 mmol, 29% yield) as a white powder.

^{1}H NMR (600 MHz, DMSO-*d6*) δ 11.57 (s, 1H), 8.90 (s, 1H), 8.72 - 8.46 (m, 2H), 7.91 (s, 1H), 7.85 (dd, *J* = 8.1, 2.5, Hz, 1H), 7.57 (dd, *J* = 8.2, 4.8, Hz, 1H), 7.43 - 7.06 (m, 4H), 6.14 (d, *J* = 6.7 Hz, 1H), 5.23 (s, 2H), 2.51 (s, 3H), 2.13 (s, 3H). **^{13}C NMR (151 MHz, DMSO-*d6*)** δ 161.99, 161.45, 159.83, 151.59, 151.54, 151.51, 150.52, 150.03, 149.51, 147.54, 144.47, 141.18, 137.23, 134.07, 133.73, 133.09, 124.43, 121.81, 120.03, 115.27, 113.36, 109.33, 108.41, 64.34, 20.77, 11.16. **HRMS calcd for :**$C_{26}H_{21}O_4N_5{}^{35}Cl^{32}S$: 534.09973, found: 534.09886.

1-(3-Chloro-5-((2,4-dimethylthiazol-5-yl)methoxy)phenyl)-5-(4-methyl-2-oxo-1,2-dihydropyridin-3-yl)-3-(pyridin-3-yl)pyrimidine-2,4(1*H*,3*H*)-dione (18)

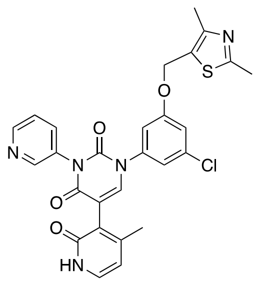

Into a 100 mL two-neck round-bottom flask under an argon atmosphere was placed 1-[3-Chloro-5-[(2,4-dimethylthiazol-5-yl)methoxy]phenyl]-5-(2-methoxy-4-methyl-3-pyridyl)-3-(3-pyridyl)pyrimidine-2,4-dione (0.070 g, 0.12 mmol), followed by dry MeCN (10 mL), forming a clear solution. To this solution was added sodium iodide (0.050 g, 0.35 mmol), followed immediately by chlorotrimethylsilane (0.040 mL, 0.35 mmol), resulting in a pale-yellow solution. The reaction mixture was heated to 60 °C for 6 h, after which LC–MS analysis indicated complete consumption of the starting material. After cooling, the reaction mixture was diluted with EtOAc (250 mL), brine (250 mL), and 10% sodium thiosulfate solution (150 mL). Following thorough mixing, the layers were separated, and the aqueous layer was extracted with EtOAc (3 × 250 mL). The combined organic fractions were washed with brine, dried over anhydrous magnesium

sulfate, filtered, and concentrated in vacuo. The crude product was purified by column chromatography (initially EtOAc/hex, followed by 3% MeOH/EtOAc) to afford 1-[3-Chloro-5-[(2,4-dimethylthiazol-5-yl)methoxy]phenyl]-5-(4-methyl-2-oxo-1H-pyridin-3-yl)-3-(3-pyridyl)pyrimidine-2,4-dione (25 mg, 0.046 mmol, 39% yield) as a white powder.

^{1}H NMR (600 MHz, DMSO-***d6)* δ** 11.59 (s, 1H), 8.61 (dd, *J* = 4.8, 1.5 Hz, 1H), 8.56 (dd, *J* = 2.5, 0.8 Hz, 1H), 7.90 (s, 1H), 7.84 (ddd, *J* = 8.1, 2.5, 1.6 Hz, 1H), 7.57 (dd, *J* = 8.1, 4.8, Hz, 1H), 7.32 (t, *J* = 1.8 Hz, 1H), 7.28 (d, *J* = 6.7 Hz, 1H), 7.24 (t, *J* = 2.1 Hz, 1H), 7.21 (t, *J* = 2.1 Hz, 1H), 6.14 (d, *J* = 6.7 Hz, 1H), 5.30 (d, *J* = 2.8 Hz, 2H), 2.59 (s, 3H), 2.33 (s, 3H), 2.13 (s, 3H). **^{13}C NMR (151 MHz,** DMSO-***d6)* δ** 165.02, 162.00, 161.45, 159.27, 151.60, 151.00, 150.51, 150.02, 149.52, 144.45, 141.19, 137.23, 134.12, 134.04, 133.08, 125.36, 124.45, 121.76, 120.28, 115.38, 113.58, 109.31, 108.42, 62.59, 20.77, 19.28, 15.24. **HRMS** calcd for :$C_{27}H_{23}O_4N_5{}^{35}Cl^{32}S$: 548.11538, found:548.11537.

1-(3-Chloro-5-((1-(difluoromethyl)-1*H*-imidazol-2-yl)methoxy)phenyl)-5-(4-methyl-2-oxo-1,2-dihydropyridin-3-yl)-3-(pyridin-3-yl)pyrimidine-2,4(1*H*,3*H*)-dione (19)

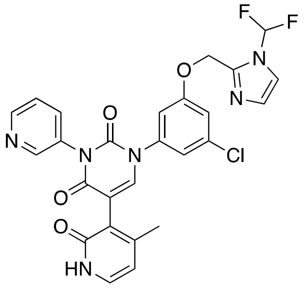

Into a 100 mL two-neck round-bottom flask under an argon atmosphere was placed 1-[3-Chloro-5-[[1-(difluoromethyl)imidazol-2-yl]methoxy]phenyl]-5-(2-methoxy-4-methyl-3-pyridyl)-3-(3-pyridyl)pyrimidine-2,4-dione (0.11 g, 0.19 mmol), followed by dry MeCN (10 mL), forming a clear solution. Sodium iodide (0.090 g, 0.58 mmol) was then added, followed immediately by chlorotrimethylsilane (0.070 mL, 0.58 mmol), resulting in a pale-yellow solution. The reaction mixture was heated to 60 °C for 6 h, after which LC–MS analysis indicated complete consumption of the starting material. After cooling, the reaction mixture was diluted with EtOAc (250 mL), brine (250 mL), and 10% sodium thiosulfate solution (150 mL). After thorough mixing, the layers were separated, and the aqueous layer was extracted with EtOAc (3 × 250 mL). The combined organic fractions were washed with brine, dried over anhydrous magnesium sulfate, filtered, and concentrated in vacuo. The crude product was purified by column chromatography (initially EtOAc/hex, followed by 3% MeOH/EtOAc) to afford 1-[3-Chloro-5-[[1-(difluoromethyl)imidazol-2-yl]methoxy]phenyl]-5-(4-methyl-2-oxo-1H-pyridin-3-yl)-3-(3-pyridyl)pyrimidine-2,4-dione (65 mg, 0.12 mmol, 61% yield) as a white powder.

^{1}H NMR (600 MHz, DMSO-*d6)* δ 11.59 (s, 1H), 8.61 (dd, *J* = 4.8, 1.6 Hz, 1H), 8.57 (dd, *J* = 2.4, 0.8 Hz, 1H), 8.01 (s, 0.27H), 7.91 (s, *J*: 60 Hz, 0.53H), 7.82 (s, 0.26H), 7.89 (s, 1H), 7.85 (dd, *J* = 8.1, 2.5 Hz, 1H), 7.70 (d, *J* = 1.6 Hz, 1H), 7.57 (dd, *J* = 8.1, 4.8, Hz, 1H), 7.36 (t, *J* = 1.8 Hz, 1H), 7.30 (t, *J* = 2.1 Hz, 1H), 7.28 (d, *J* = 6.7 Hz, 1H), 7.25 (t, *J* = 2.1 Hz, 1H), 7.13 (d, *J* = 1.5 Hz, 1H), 6.14 (d, *J* = 6.7 Hz, 1H), 5.41 - 5.33 (m, 2H), 2.13 (s,

3H). **^{19}F NMR (565 MHz, DMSO-*d6*)** δ -91.88, -91.98. **^{13}C NMR (201 MHz, DMSO-*d6*)** δ 161.99, 161.44, 158.99, 151.62, 150.50, 150.03, 149.51, 144.42, 142.45, 141.17, 137.22, 134.08, 133.09, 129.79, 124.43, 121.77, 120.63, 118.15, 115.35, 113.58, 109.34, 108.89 (t, *J*: 185 Hz, C-F_2: 110.12, 108.89, 107.67), 108.43, 107.65, 62.83, 20.77. **HRMS** (APCI+) $[M+H]^+$ calc for**:** $C_{26}H_{20}O_4N_6{}^{35}ClF_2$: 553.11971, found:553.11976.

1-(3-Chloro-5-(cyclopropylmethoxy)phenyl)-5-(4-methyl-2-oxo-1,2-dihydropyridin-3-yl)-3-(pyridin-3-yl)pyrimidine-2,4(1*H*,3*H*)-dione (20)

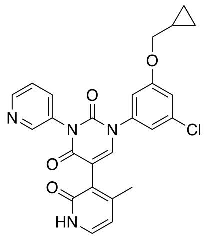

Into a 100 mL two-neck round-bottom flask under an argon atmosphere was placed 1-[3-Chloro-5-(cyclopropylmethoxy)phenyl]-5-(2-methoxy-4-methyl-3-pyridyl)-3-(3-pyridyl)pyrimidine-2,4-dione (0.080 g, 0.15 mmol), followed by dry MeCN (10 mL), forming a clear solution. Sodium iodide (0.070 g, 0.46 mmol) was then added, followed immediately by chlorotrimethylsilane (0.060 mL, 0.46 mmol), resulting in a pale-yellow solution. The reaction mixture was heated to 60 °C for 6 h, after which LC–MS analysis indicated complete consumption of the starting material. After cooling, the reaction mixture was diluted with EtOAc (250 mL), brine (250 mL), and 10% sodium thiosulfate solution (150 mL). The mixture was thoroughly mixed, and the layers were separated. The aqueous phase was extracted with EtOAc (3 × 250 mL). The combined organic fractions were washed with brine, dried over anhydrous magnesium sulfate, filtered, and concentrated in vacuo. The crude material was purified by column chromatography (initially EtOAc/hex, followed by 3% MeOH/EtOAc) to afford 1-[3-Chloro-5-(cyclopropylmethoxy)phenyl]-5-(4-methyl-2-oxo-1H-pyridin-3-yl)-3-(3-pyridyl)pyrimidine-2,4-dione (70 mg, 0.15 mmol, 95% yield) as a white powder.

^{1}H NMR (600 MHz, DMSO-*d6*) δ 11.58 (s, 1H), 8.61 (dd, *J* = 4.8, 1.5 Hz, 1H), 8.56 (d, *J* = 0.8 Hz, 1H), 7.88 (s, 1H), 7.84 (dd, *J* = 8.1, 2.5 Hz, 1H), 7.57 (dd, *J* = 8.1, 4.8 Hz, 1H), 7.28 (d, *J* = 6.7 Hz, 1H), 7.25 (t, *J* = 1.8 Hz, 1H), 7.15-7.11 (m, 2H), 6.14 (d, *J* = 6.7 Hz, 1H), 3.89 (dd, *J* = 7.1, 1.1 Hz, 2H), 2.13 (s, 3H), 1.28 - 1.18 (m, 1H), 0.62 - 0.54 (m, 2H), 0.35 - 0.30 (m, 2H). **^{13}C NMR (151 MHz, DMSO-*d6*)** δ 162.01, 161.47, 160.11, 151.61, 150.53, 150.02, 149.50, 144.54, 141.23, 137.23, 134.11, 134.00, 133.10, 124.44, 121.79, 119.57, 115.17, 112.95, 109.19, 108.45, 73.44, 20.78, 10.35, 3.59. **HRMS calcd for :**$C_{25}H_{22}O_4N_4{}^{35}Cl$: 477.13241, found:477.13233.

Scheme S1. Synthesis of compounds 21-23

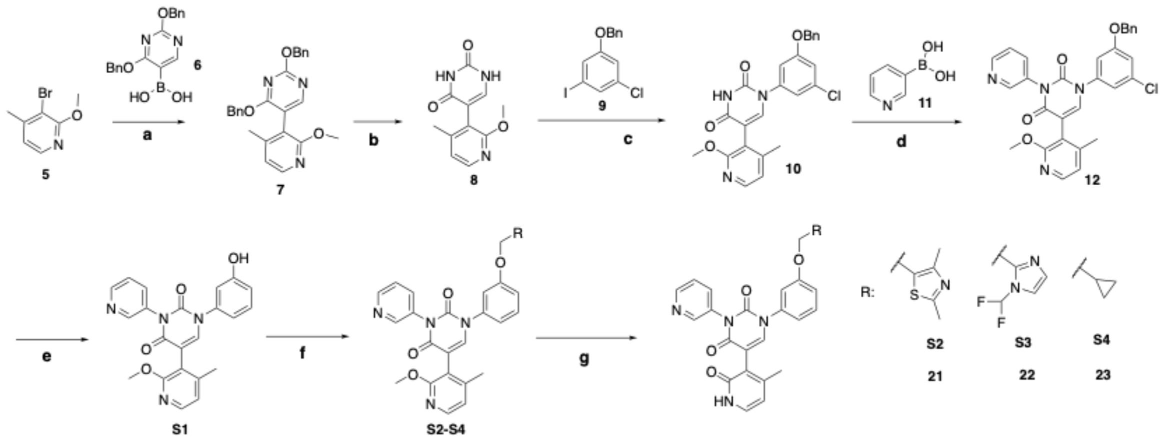

Reagents and conditions: a) $Pd(PPh_3)_4$, $NaHCO_3$, DME/water, 40%; b) 10% Pd/C, H_2, MeOH/THF (1:1), 98% c) CuI, K_3PO_4, N-(2-cyanophenyl)picolinamide, DMSO, 57%; d) $Cu(OAc)_2$, TMEDA, DMSO, 94%; e) 10% Pd/C, H_2, MeOH/THF (1:1), 50%; f) K_2CO_3, R-X, DMF; g) TMS-Cl, NaI, MeCN.

1-(3-((2,4-Dimethylthiazol-5-yl)methoxy)phenyl)-5-(2-methoxy-4-methylpyridin-3-yl)-3-(pyridin-3-yl)pyrimidine-2,4(1*H*,3*H*)-dione (S2)

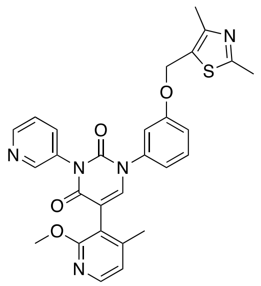

These products were isolated following the reaction of the mixture of compounds **13** and **S1** with the corresponding R–CH_2–X reagents during the synthesis of compounds **15**–**17**. The crude materials were purified by chromatography using DCM/methanol (97:3), followed by reverse-phase CombiFlash purification with water and acetonitrile to afford 110 mg of **S2**.

^{1}H NMR (400 MHz, MeOD) δ 8.60 (d, *J* = 3.1 Hz, 2H), 8.04 - 7.96 (m, 1H), 7.93-7.88 (m, 1H), 7.80 (s, 1H), 7.60 (dd, *J* = 8.2, 4.9 Hz, 1H), 7.45 (t, *J* = 8.2 Hz, 1H), 7.22 (t, *J* = 2.2 Hz, 1H), 7.12 (dd, *J* = 15.5, 8.1 Hz, 2H), 6.92 (d, *J* = 5.2 Hz, 1H), 5.22 (s, 2H), 3.91 (s, 3H), 2.62 (s, 3H), 2.37 (s, 3H), 2.32 (s, 3H).

1-(3-((1-(Difluoromethyl)-1*H*-imidazol-2-yl)methoxy)phenyl)-5-(2-methoxy-4-methylpyridin-3-yl)-3-(pyridin-3-yl)pyrimidine-2,4(1*H*,3*H*)-dione (S3)

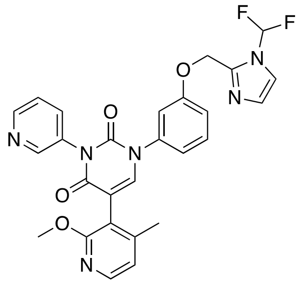

The products were isolated after the reaction of the mixture of compounds **13** and **S1** with the corresponding R–CH_2–X reagents during the synthesis of compounds **15**–**17**. The crude materials were first purified by chromatography using DCM/MeOH (97:3), followed by reverse-phase CombiFlash purification with water and acetonitrile to afford 120 mg of **S3**.

^{1}H NMR (400 MHz, MeOD) δ 8.65 - 8.51 (m, 2H), 8.01 (d, *J* = 5.2 Hz, 1H), 7.89 (dd, *J* = 8.2, 2.5 Hz, 1H), 7.80 (s, 1H), 7.69 (s, 1H), 7.60 - 7.51 (m, 2H), 7.45 (t, *J* = 8.2 Hz, 1H), 7.27 (t, *J* = 2.3 Hz, 1H), 7.19 - 7.12 (m, 2H), 7.10 (d, *J* = 1.6 Hz, 1H), 6.91 (d, *J* = 5.3 Hz, 1H), 5.31 (s, 2H), 3.91 (s, 3H), 2.32 (s, 3H). **^{19}F NMR (376 MHz, MeOD)** δ -94.21, -94.37.

1-(3-(Cyclopropylmethoxy)phenyl)-5-(2-methoxy-4-methylpyridin-3-yl)-3-(pyridin-3-yl)pyrimidine-2,4(1*H*,3*H*)-dione (S4)

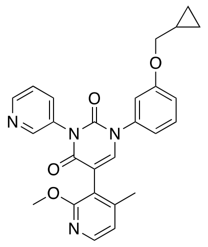

The products were isolated after the reaction of the mixture of compounds **13** and **S1** with the corresponding R–CH_2–X reagents during the synthesis of compounds **15**–**17**. The crude products were first purified by chromatography using DCM/MeOH (97:3), followed by reverse-phase CombiFlash purification with water and acetonitrile to afford 70 mg of **S4**.

^{1}H NMR (400 MHz, MeOD) δ 8.62 - 8.53 (m, 2H), 8.01 (d, *J* = 5.2 Hz, 1H), 7.91 (ddd, *J* = 8.2, 2.5, 1.5 Hz, 1H), 7.78 (s, 1H), 7.60 (dd, *J* = 8.2, 4.9 Hz, 1H), 7.41 (t, *J* = 8.1 Hz, 1H), 7.11 (t, *J* = 2.2 Hz, 1H), 7.06 (dd, *J* = 7.9, 0.9 Hz, 1H), 7.02 (dd, *J* = 8.4, 0.9 Hz, 1H), 6.92 (dd, *J* = 5.3, 0.7 Hz, 1H), 3.91 (s, 3H), 3.87 (d, *J* = 6.9 Hz, 2H), 2.32 (s, 3H), 1.33 - 1.20 (m, 1H), 0.72 - 0.51 (m, 2H), 0.45 - 0.24 (m, 2H).

1-(3-((2,4-Dimethylthiazol-5-yl)methoxy)phenyl)-5-(4-methyl-2-oxo-1,2-dihydropyridin-3-yl)-3-(pyridin-3-yl)pyrimidine-2,4(1*H*,3*H*)-dione (21)

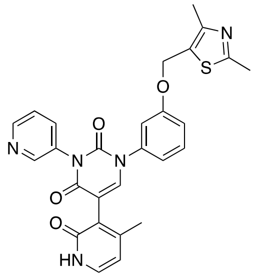

Into a 100 mL two-neck round-bottom flask under an argon atmosphere was placed 1-[3-[(2,4-dimethylthiazol-5-yl)methoxy]phenyl]-5-(2-methoxy-4-methyl-3-pyridyl)-3-(3-pyridyl)pyrimidine-2,4-dione (0.11 g, 0.21 mmol), followed by dry MeCN (10 mL), forming a clear solution. Sodium iodide (0.090 g, 0.63 mmol) was then added, followed immediately by chlorotrimethylsilane (0.080 mL, 0.63 mmol), resulting in a pale-yellow solution. The reaction mixture was heated to 60 °C for 6 h, after which LC–MS analysis indicated complete consumption of the starting material. After cooling, the reaction mixture was diluted with EtOAc (250 mL), brine (250 mL), and 10% sodium thiosulfate solution (150 mL). The mixture was thoroughly mixed, and the layers were separated. The aqueous layer was extracted with EtOAc (3 × 250 mL). The combined organic layers were washed with brine, dried over anhydrous magnesium sulfate, filtered, and concentrated in vacuo. The crude product was purified by column chromatography (initially EtOAc/hex, followed by 3% MeOH/EtOAc) to afford 1-[3-[(2,4-dimethylthiazol-5-yl)methoxy]phenyl]-5-(4-methyl-2-oxo-1H-pyridin-3-yl)-3-(3-pyridyl)pyrimidine-2,4-dione (24 mg, 0.048 mmol, 23% yield) as a white powder.

^{1}H NMR (400 MHz, DMSO-*d6*) δ 11.58 (s, 1H), 8.66 - 8.60 (m, 1H), 8.58 (d, *J* = 2.5 Hz, 1H), 7.85 (d, *J* = 5.9 Hz, 2H), 7.57 (dd, *J* = 8.1, 4.8 Hz, 1H), 7.44 (s, 1H), 7.27 (d, *J* = 6.7 Hz, 1H), 7.22 (t, *J* = 2.3 Hz, 1H), 7.15 (dd, *J* = 7.9, 2.0 Hz, 1H), 7.09 (dd, *J* = 8.4, 2.5 Hz, 1H), 6.14 (d, *J* = 6.7 Hz, 1H), 5.26 (s, 2H), 2.58 (s, 3H), 2.33 (s, 3H), 2.14 (s, 3H). **^{13}C NMR (101 MHz, DMSO-*d6*)** δ 164.75, 162.03, 161.51, 158.65, 151.56, 150.63, 150.60, 150.06, 149.48, 144.93, 140.53, 137.26, 133.97, 133.18, 130.40, 125.91, 124.44, 121.82, 119.92, 115.49, 114.10, 109.02, 108.46, 62.20, 20.81, 19.26, 15.26.. **HRMS calcd for** :$C_{27}H_{24}O_4N_5{}^{32}S$: 514.15435, found:514.15438.

1-(3-((1-(Difluoromethyl)-1*H*-imidazol-2-yl)methoxy)phenyl)-5-(4-methyl-2-oxo-1,2-dihydropyridin-3-yl)-3-(pyridin-3-yl)pyrimidine-2,4(1*H*,3*H*)-dione (22)

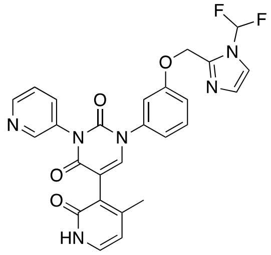

Into a 100 mL two-neck round-bottom flask under an argon atmosphere was placed 1-[3-[[1-(difluoromethyl)imidazol-2-yl]methoxy]phenyl]-5-(2-methoxy-4-methyl-3-pyridyl)-3-(3-pyridyl)pyrimidine-2,4-dione (0.12 g, 0.23 mmol), followed by dry MeCN (10 mL), forming a clear solution. Sodium iodide (0.10 g, 0.68 mmol) was then added, followed immediately by chlorotrimethylsilane (0.090 mL, 0.68 mmol), resulting in a pale-yellow solution. The reaction mixture was heated to 60 °C for 6 h, after which LC–MS analysis indicated complete consumption of the starting material. After cooling, the reaction mixture was diluted with EtOAc (250 mL), brine (250 mL), and 10% sodium thiosulfate solution (150 mL). The mixture was thoroughly mixed, and the layers were separated. The aqueous phase was extracted with EtOAc (3 × 250 mL). The combined organic layers were washed with brine, dried over anhydrous magnesium sulfate, filtered, and concentrated in vacuo. The crude material was purified by column chromatography (initially EtOAc/hex, followed by 3% MeOH/EtOAc) to afford 1-[3-[[1-(difluoromethyl)imidazol-2-yl]methoxy]phenyl]-5-(4-methyl-2-oxo-1H-pyridin-3-yl)-3-(3-pyridyl)pyrimidine-2,4-dione (65 mg, 0.13 mmol, 56% yield) as a white powder.

^{1}H NMR (600 MHz, DMSO-*d6)* δ 11.55 (s, 1H), 8.61 (dd, *J* = 4.8, 1.5 Hz, 1H), 8.58 (dd, *J* = 2.4, 0.8 Hz, 1H), 7.81, 7.91, 8.01 (t, *J*: 60 Hz, 1H (0.25, 0.5, 0.25), CH-F_2), 7.89 - 7.83 (m, 2H), 7.81 (s, 0H), 7.69 (d, *J* = 1.6 Hz, 1H), 7.57 (ddd, *J* = 8.1, 4.8, 0.8 Hz, 1H), 7.46 (t, *J* = 8.2 Hz, 1H), 7.30 - 7.24 (m, 2H), 7.18 (dd, *J* = 8.0, 2.0 Hz, 1H), 7.15 (dd, *J* = 8.4, 2.5 Hz, 1H), 7.12 (d, *J* = 1.6 Hz, 1H), 6.14 (d, *J* = 6.7 Hz, 1H), 5.37 - 5.29 (m, 2H), 2.14 (s, 3H). **^{19}F NMR (565 MHz, DMSO-*d6)*** δ -91.85, -91.96. **^{13}C NMR (151 MHz, DMSO-*d6)*** δ 162.04, 161.52, 158.38, 151.61, 150.62, 150.06, 149.47, 144.91, 142.89, 140.51, 137.27, 133.96, 133.20, 130.39, 129.70, 124.44, 121.81, 120.28, 118.02, 115.32, 114.12, 109.04, 108.91 (t, *J*: 247 Hz, C-F_2: 110.55, 108.91, 107.26), 108.49, 62.49, 20.81. **HRMS calcd for :**$C_{26}H_{21}O_4N_6F_2$: 519.15869, found:519.15867.

1-(3-(Cyclopropylmethoxy)phenyl)-5-(4-methyl-2-oxo-1,2-dihydropyridin-3-yl)-3-(pyridin-3-yl)pyrimidine-2,4(1*H*,3*H*)-dione (23)

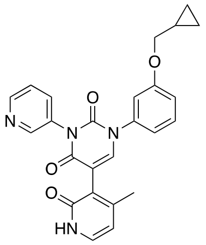

Into a 100 mL two-neck round-bottom flask under an argon atmosphere was placed 1-[3-(cyclopropylmethoxy)phenyl]-5-(2-methoxy-4-methyl-3-pyridyl)-3-(3-pyridyl)pyrimidine-2,4-dione (0.070 g, 0.15 mmol), followed by dry MeCN (10 mL), forming a clear solution. Sodium iodide (0.070 g, 0.46 mmol) was then added, followed immediately by chlorotrimethylsilane (0.060 mL, 0.46 mmol), resulting in a pale-yellow solution. The reaction mixture was heated to 60 °C for 6 h, after which LC–MS analysis indicated complete consumption of the starting material. After cooling, the reaction mixture was diluted with EtOAc (250 mL), brine (250 mL), and 10% sodium thiosulfate solution (150 mL). The mixture was thoroughly mixed, and the layers were separated. The aqueous phase was extracted with EtOAc (3 × 250 mL). The

combined organic layers were washed with brine, dried over anhydrous magnesium sulfate, filtered, and concentrated in vacuo. The crude product was purified by column chromatography (initially EtOAc/hex, followed by 3% MeOH/EtOAc) to afford 1-[3-(cyclopropylmethoxy)phenyl]-5-(4-methyl-2-oxo-1H-pyridin-3-yl)-3-(3-pyridyl)pyrimidine-2,4-dione (61 mg, 0.16 mmol, 90% yield) as a white powder.

^{1}H NMR (600 MHz, DMSO-*d6*) δ 11.56 (s, 1H), 8.60 (dd, *J* = 4.8, 1.5 Hz, 1H), 8.57 (d, *J* = 0.8 Hz, 1H), 7.85 (dd, *J* = 8.1, 2.5 Hz, 1H), 7.83 (s, 1H), 7.56 (dd, *J* = 8.1, 4.8 Hz, 1H), 7.41 (t, *J* = 8.1 Hz, 1H), 7.27 (d, *J* = 6.7 Hz, 1H), 7.12 (t, *J* = 2.2 Hz, 1H), 7.10 - 7.06 (m, 1H), 7.01 (dd, *J* = 8.4, 0.9 Hz, 1H), 6.14 (d, *J* = 6.7 Hz, 1H), 3.86 (d, *J* = 7.0 Hz, 2H), 2.13 (s, 3H), 1.28 - 1.19 (m, 1H), 0.62 - 0.53 (m, 2H), 0.35 - 0.30 (m, 2H). **^{13}C NMR (151 MHz, DMSO-*d6*)** δ 162.05, 161.54, 159.45, 151.58, 150.65, 150.06, 149.45, 145.01, 140.56, 137.26, 133.93, 133.21, 130.35, 124.43, 121.83, 119.13, 115.32, 113.46, 108.90, 108.50, 72.83, 20.81, 10.51, 3.58, 3.56. **HRMS calcd for :**$C_{25}H_{23}O_4N_4$: 443.17138, found:443.17104.

2,4-Bis(benzyloxy)-5-(2-methoxypyridin-3-yl)pyrimidine (25)

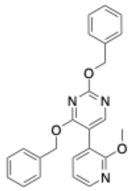

Into a 500 mL three-neck round-bottom flask fitted with a condenser and under an argon atmosphere was placed 3-bromo-2-methoxy-pyridine 8 (2.80 mL, 23.0 mmol), followed by DME (200 mL), forming a clear solution. To this was added (2,4-dibenzyloxypyrimidin-5-yl)boronic acid 9 (9.65 g, 28.7 mmol), which dissolved rapidly, after which a saturated aqueous sodium bicarbonate solution (150 mL) was added, resulting in immediate formation of a white precipitate. The mixture was degassed for 10 min by bubbling argon through the solution, and tetrakis(triphenylphosphine)palladium(0) (5.53 g, 4.79 mmol) was added in one portion. The reaction mixture was heated to reflux under argon, during which most of the white precipitate dissolved, forming a yellow solution. The mixture was refluxed for 6 h, during which the color gradually deepened to a dark orange. After this time, the reaction was cooled, poured into a separating funnel, and diluted with EtOAc (400 mL) and water (300 mL). After vigorous shaking, the layers were allowed to separate, and the organic phase was collected. The aqueous layer was extracted with EtOAc (2 × 200 mL). The combined organic layers were dried over anhydrous magnesium sulfate and filtered. The filtrate was concentrated to approximately 300 mL and passed through a silica gel pad, which was subsequently washed with ethyl acetate. The organic solution was concentrated in vacuo, and the crude product was purified by column chromatography (EtOAc/hex) to afford 2,4-dibenzyloxy-5-(2-methoxy-3-pyridyl)pyrimidine (8.86 g, 22.2 mmol, 93% yield) as a white solid.

^{1}H NMR (400 MHz, $CDCl_3$) δ 8.28 (s, 1H), 8.18 (dd, *J* = 5.1, 1.9 Hz, 1H), 7.56 (dd, *J* = 7.3, 1.9 Hz, 1H), 7.52 - 7.48 (m, 2H), 7.42 - 7.27 (m, 8H), 6.94 (dd, *J* = 7.3, 5.0 Hz, 1H), 5.47 (s, 2H), 5.45 (s, 2H), 3.87 (s, 3H).

5-(2-Methoxypyridin-3-yl)pyrimidine-2,4(1H,3H)-dione (26)

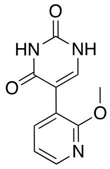

Into a 1000 mL three-neck round-bottom flask containing methanol (300 mL) and THF (300 mL) was placed 2,4-dibenzyloxy-5-(2-methoxy-3-pyridyl)pyrimidine (13.5 g, 33.7 mmol), forming a clear solution. The flask was purged with argon, and Pd/C (3.58 g, 33.7 mmol) was added in one portion. A hydrogen-filled balloon was fitted to the flask, and the atmosphere was purged with hydrogen. The reaction mixture was stirred at 45 °C for 36 h, after which LC–MS analysis indicated complete conversion of the starting material. The reaction mixture was filtered through celite and concentrated in vacuo to afford 5-(2-methoxy-3-pyridyl)-1H-pyrimidine-2,4-dione (7.30 g, 33.3 mmol, 99% yield) as a white solid. *(Attempt purification by column chromatography; the compound precipitates on the column and causes severe operational difficulties.)*

^{1}H NMR (600 MHz, DMSO-*d6*) δ 11.20 (s, 1H), 11.04 (s, 1H), 8.10-8.05 (m, 1H), 7.64 (dd, *J* = 7.3, 1.8 Hz, 1H), 7.53 (s, 1H), 6.96 (dd, *J* = 7.3, 5.0 Hz, 1H), 3.78 (s, 3H).

3-(Cyclopropylmethoxy)-1-fluoro-5-iodo-benzene (27)

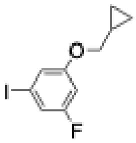

To a flame-dried 50 mL round-bottom flask equipped with a Teflon-coated stir bar were added 5-fluoro-3-iodophenol (900 mg, 3.78 mmol) and potassium carbonate (522 mg, 3.78 mmol). Acetone (11 mL) was added, and the mixture was stirred for 10 min at room temperature. (bromomethyl)cyclopropane (562 mg, 4.16 mmol) was then added, and the reaction mixture was heated to reflux overnight. After cooling to room temperature, water was added followed by EtOAc. The aqueous layer was extracted with EtOAc (3 × 50 mL). The combined organic layers were dried over sodium sulfate, filtered, concentrated under reduced pressure, and purified by silica gel flash chromatography (EtOAc/Hex, 1:9) to afford the product as a clear oil (830 mg, 2.84 mmol, 75% yield).

^{1}H NMR (400 MHz, $CDCl_3$) δ 7.07 - 6.98 (m, 2H), 6.61-6.56 (m, 1H), 3.75 (d, *J* = 7.0 Hz, 2H), 1.31 - 1.17 (m, 1H), 0.71 - 0.59 (m, 2H), 0.38 - 0.30 (m, 2H). **^{19}F NMR (400 MHz, $CDCl_3$)** δ -110.4.

1-Bromo-3-(cyclopropylmethoxy)-5-iodo-benzene (28)

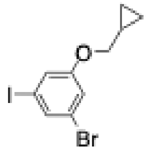

In a flame-dried 50 mL round-bottom flask equipped with a Teflon-coated stir bar containing 5-bromo-3-iodophenol (1.00 g, 3.35 mmol) and potassium carbonate (460 mg, 3.35 mmol), was added acetone (11 mL) and the mixture was stirred for 10 min at room temperature. (bromomethyl)cyclopropane (500 mg, 3.68 mmol) was then added, and the reaction mixture was heated to reflux overnight. After cooling to room temperature, water and EtOAc were added to the reaction mixture. The aqueous phase was extracted with EtOAc (3 × 50 mL). The combined organic extracts were dried over sodium sulfate, filtered, concentrated under reduced pressure, and purified via silica gel flash chromatography (EtOAc/hex, 1:9) to afford the product as a clear oil (1.0 g, 2.83 mmol, 85% yield).

^{1}H NMR (400 MHz, $CDCl_3$) δ 7.42 (s, 1H), 7.18 (s, 1H), 7.01 (s, 1H), 3.75 (d, *J* = 7.0 Hz, 2H), 1.31 - 1.16 (m, 1H), 0.71 - 0.59 (m, 2H), 0.33 (dt, *J* = 6.4, 4.7 Hz, 2H).

1-Chloro-3-(cyclopropylmethoxy)-2-flouro-5-iodo-benzene (29)

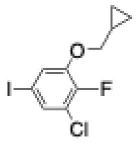

In a flame-dried microwave vial equipped with a Teflon-coated stir bar containing 3-Chloro-2-fluoro-5-iodophenol (600 mg, 2.21 mmol) and potassium carbonate (310 mg, 2.21 mmol), was added acetone (8 mL) and the reaction mixture was stirred for 10 min at room temperature. (bromomethyl)cyclopropane (330 mg, 2.43 mmol) was then added, and the microwave vial was capped; the reaction mixture was heated to reflux overnight. After cooling to room temperature, water and EtOAc were added to the reaction mixture. The aqueous phase was extracted with EtOAc (3 × 15 mL). The combined organic layers were dried over sodium sulfate, filtered, and concentrated under reduced pressure. Purification via silica gel flash chromatography (EtOAc/hex, 1:9 to 3:7) afforded 1-chloro-3-(cyclopropylmethoxy)-2-fluoro-5-iodobenzene as a clear oil (570 mg, 1.75 mmol, 79% yield).

^{1}H NMR (400 MHz, $CDCl_3$) δ 7.29 - 7.23 (m, 1H), 7.09 (dd, *J* = 7.0, 2.1 Hz, 1H), 3.82 (d, *J* = 7.1 Hz, 2H), 1.33 - 1.19 (m, 1H), 0.70 - 0.58 (m, 2H), 0.38 - 0.29 (m, 2H). **^{19}F NMR (400 MHz, $CDCl_3$) δ** -135.4

1-[3-(Cyclopropylmethoxy)-5-flourophenyl]-5-(2-methoxy-3-pyridyl)pyrimide-2,4-dione (30)

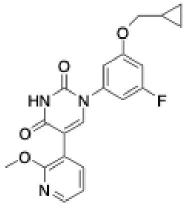

In a flame-dried microwave vial under an argon atmosphere was placed DMSO (10 mL), followed by 5-(2-methoxy-3-pyridyl)-1H-pyrimidine-2,4-dione (520 mg, 2.36 mmol), 1-(cyclopropylmethoxy)-3-fluoro-5-iodobenzene (830 mg, 2.84 mmol), and potassium phosphate (1.00 g, 4.96 mmol). The reaction mixture was degassed by bubbling argon through the solution for 1 h, after which copper(I)iodide (50 mg, 0.24 mmol) and N-(2-cyanophenyl)picolinamide (60 mg, 0.28 mmol) were added under a flow of argon. The reaction mixture was heated to 95 °C and stirred for 24 h. The reaction mixture was then passed through a pad of celite and washed with EtOAc (200 mL). Water (300 mL) was added to the organic layer, which was further extracted with EtOAc (2 × 300 mL). The combined organic layers were dried over sodium sulfate, filtered, and concentrated under reduced pressure. Purification via silica gel flash chromatography (EtOAc/hex, 2:8 to 6:4) afforded 1-[3-(cyclopropylmethoxy)-5-fluorophenyl]-5-(2-methoxy-3-pyridyl)pyrimidine-2,4-dione (651 mg, 1.70 mmol, 72% yield) as a white amorphous solid.

^{1}H NMR (400 MHz, $CDCl_3$) δ 9.40 (d, *J* = 12.8 Hz, 1H), 8.14 (dd, *J* = 5.0, 1.9 Hz, 1H), 7.85 (dd, *J* = 7.4, 1.9 Hz, 1H), 7.70 (s, 1H), 6.94 (dd, *J* = 7.4, 5.0 Hz, 1H), 6.74 (dd, *J* = 8.8, 2.2 Hz, 2H), 6.68 (dt, *J* = 10.4, 2.2 Hz, 1H), 3.95 (s, 3H), 3.80 (d, *J* = 7.0 Hz, 2H), 1.33 - 1.18 (m, 1H), 0.71 - 0.58 (m, 2H), 0.34 (d, *J* = 6.0, Hz, 2H. **^{19}F NMR (400 MHz, $CDCl_3$)** δ -109.1

1-[3-Bromo-5-(cyclopropylmethoxy)phenyl]-5-(2-methoxy-3-pyridyl)pyrimide-2,4-dione (31)

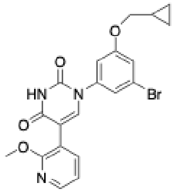

Into a two-neck round-bottom flask under an argon atmosphere was placed DMSO (70 mL), followed by 5-(2-methoxy-3-pyridyl)-1H-pyrimidine-2,4-dione (50 mg, 0.23 mmol), 1-bromo-3-(cyclopropylmethoxy)-5-iodobenzene (100 mg, 0.270 mmol), N-(2-cyanophenyl)picolinamide (10 mg, 0.030 mmol), and potassium phosphate (100 mg, 0.480 mmol), forming a brown suspension. The reaction mixture was degassed by bubbling argon through the solution for 10 min, after which copper(I)iodide (4 mg, 0.02 mmol) was added under a flow of argon. The mixture was heated to 60 °C and stirred for three days. The reaction mixture was diluted with ethyl acetate (200 mL) and water (500 mL). A suspension formed, which was broken by passing the entire mixture through a pad of celite. The organic layer was separated, and

the aqueous layer was extracted with EtOAc (2 × 200 mL). The combined organic layers were dried over sodium sulfate, filtered, and concentrated under reduced pressure. Purification via silica gel flash chromatography (EtOAc/hex, 1:9 to 1:1) afforded 1-[3-bromo-5-(cyclopropylmethoxy)phenyl]-5-(2-methoxy-3-pyridyl)pyrimidine-2,4-dione (80 mg, 0.018 mmol, 80% yield) as a white solid.

^{1}H NMR (400 MHz, $CDCl_3$) δ 8.16 (dd, *J* = 5.0, 1.9 Hz, 1H), 7.83 (dd, *J* = 7.4, 1.9 Hz, 1H), 7.68 (s, 1H), 7.18 - 7.10 (m, 2H), 6.97 (dd, *J* = 7.4, 5.0 Hz, 1H), 6.91 (t, *J* = 2.1 Hz, 1H), 3.96 (s, 3H), 3.82 (d, *J* = 7.0 Hz, 2H), 1.29-1.24 (m, 2H), 0.73 - 0.60 (m, 2H), 0.39 - 0.31 (m, 2H).

1-[3-Chloro-5-(cyclopropylmethoxy)-4-flourophenyl]-5-(2-methoxy-3-pyridyl)pyrimide-2,4-dione (32)

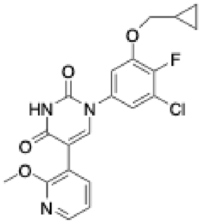

In a flame-dried 20 mL microwave vial under an argon atmosphere was placed DMSO (10 mL), followed by 5-(2-methoxy-3-pyridyl)-1H-pyrimidine-2,4-dione (320 mg, 1.46 mmol), 1-chloro-3-(cyclopropylmethoxy)-2-fluoro-5-iodobenzene (570 mg, 1.75 mmol), and potassium phosphate (650 mg, 3.07 mmol). The reaction mixture was degassed by bubbling argon through the solution for 1 h, after which copper(I)iodide (30 mg, 0.15 mmol) and N-(2-cyanophenyl)picolinamide (60 mg, 0.28 mmol) were added under a flow of argon. The mixture was heated to 95 °C and stirred for 24 h. The reaction mixture was then passed through a pad of celite and washed with EtOAc (200 mL). Water (300 mL) was added to the organic layer, which was extracted with EtOAc (2 × 300 mL). The combined organic layers were dried over sodium sulfate, filtered, concentrated under reduced pressure, and purified via silica gel flash chromatography (EtOAc/hex, 1:4 to 3:2) to afford 1-[3-Chloro-5-(cyclopropylmethoxy)-4-fluorophenyl]-5-(2-methoxy-3-pyridyl)pyrimidine-2,4-dione (150 mg, 0.359 mmol, 25% yield) as a white amorphous solid.

^{1}H NMR (400 MHz, $CDCl_3$) δ 9.71 (s, 1H), 8.14 (dd, *J* = 5.0, 1.9 Hz, 1H), 7.84 (dd, *J* = 7.5, 1.9 Hz, 1H), 7.66 (s, 1H), 7.01 (dd, *J* = 5.4, 2.4 Hz, 1H), 6.94 (d, *J* = 7.4, Hz, 2H), 3.94 (s, 3H), 3.88 (d, *J* = 7.0 Hz, 2H), 1.29-1.25 (m, 1H), 0.72 - 0.59 (m, 2H), 0.35 (t, *J* = 5.2 Hz, 2H). **^{19}F NMR (400 MHz, $CDCl_3$) δ** -133.3

1-[3-(Cyclopropylmethoxy)-5-flourophenyl]-5-(2-methoxy-3-pyridyl)-3-(3-pyridyl)pyrimidine-2,4-dione (33)

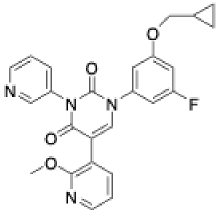

Into a 100 mL round-bottom flask was placed in order 1-[3-(cyclopropylmethoxy)-5-fluorophenyl]-5-(2-methoxy-3-pyridyl)pyrimidine-2,4-dione (300 mg, 0.780 mmol), 3-pyridylboronic acid (290 mg, 2.35 mmol), copper(II)acetate (210 mg, 1.17 mmol), DMSO (31 mL), and TMEDA (0.250 mL, 1.71 mmol), forming a deep blue solution. The reaction mixture was stirred open to the atmosphere at 60 °C for 18 h. LC–MS analysis confirmed complete conversion of the starting material. The reaction mixture was diluted with EtOAc (200 mL) and water (300 mL) and mixed vigorously. The layers were separated, and the aqueous phase was extracted with EtOAc (2 × 300 mL). The combined organic layers were dried over anhydrous sodium sulfate, filtered, and concentrated under reduced pressure. Purification via silica gel flash chromatography (EtOAc/hex, 1:4 to 1:1) afforded 1-[3-(cyclopropylmethoxy)-5-fluorophenyl]-5-(2-methoxy-3-pyridyl)-3-(3-pyridyl)pyrimidine-2,4-dione (200 mg, 0.434 mmol, 56% yield).

^{1}H NMR (400 MHz, $CDCl_3$) δ 8.63 (dd, *J* = 4.9, 1.5 Hz, 1H), 8.60 (d, *J* = 2.5 Hz, 1H), 8.14 (dd, *J* = 4.9, 1.9 Hz, 1H), 7.84 - 7.76 (m, 2H), 7.74-7.69 (m, 1H), 7.45 (dd, *J* = 8.2, 4.8 Hz, 1H), 6.93 (dd, *J* = 7.5, 4.9 Hz, 1H), 6.83 - 6.74 (m, 2H), 6.68 (d, *J* = 10.4 Hz, 1H), 3.95 (s, 3H), 3.79 (d, *J* = 7.0 Hz, 2H), 1.25-1.22 (m, 1H), 0.70 - 0.57 (m, 2H), 0.34-0.31 (m, *J* = 5.0 Hz, 2H). **^{19}F NMR (400 MHz, $CDCl_3$)** δ -109.0

1-[3-Bromo-5-(cyclopropylmethoxy)phenyl]-5-(2-methoxy-3-pyridyl)-3-(3-pyridyl)pyrimidine-2,4-dione (34)

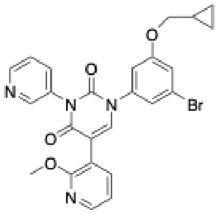

Into a 100 mL round-bottom flask was placed in order 1-[3-bromo-5-(cyclopropylmethoxy)phenyl]-5-(2-methoxy-3-pyridyl)pyrimidine-2,4-dione (230 mg, 0.510 mmol), 3-pyridylboronic acid (190 mg, 1.54 mmol), copper(II)acetate (140 mg, 0.770 mmol), DMSO (20 mL), and TMEDA (0.150 mL, 1.03 mmol), forming a deep blue solution. The reaction mixture was stirred open to the atmosphere at 60 °C for 18 h. LC–MS analysis confirmed complete conversion of the starting material. The reaction mixture was diluted with EtOAc (200 mL), water (300 mL), and mixed vigorously. The layers were separated, and the aqueous phase was extracted with EtOAc (2 × 300 mL). The combined organic extracts were dried over anhydrous sodium sulfate, filtered, and concentrated under reduced pressure. Purification via silica gel flash chromatography (EtOAc/hex, 1:4 to 1:1) afforded 1-[3-bromo-5-(cyclopropylmethoxy)phenyl]-5-(2-methoxy-3-pyridyl)-3-(3-pyridyl)pyrimidine-2,4-dione (260 mg, 0.499 mmol, 96% yield) as a light brown solid.

^{1}H NMR (600 MHz, $CDCl_3$) δ 8.67 (d, *J* = 4.8 Hz, 1H), 8.61 (d, *J* = 2.5 Hz, 1H), 8.31 (d, *J* = 2.3 Hz, 1H), 8.17 (dd, *J* = 5.0, 1.9 Hz, 2H), 7.84 (dd, *J* = 7.4, 1.9 Hz, 1H), 7.79 (s, 1H), 7.70 (dd, *J* = 8.2, 2.5 Hz, 1H), 7.46 (dd, *J* = 8.1, 4.8 Hz, 1H), 7.01 - 6.94 (m, 2H), 3.98 (s, 3H), 3.83 (d, *J* = 6.9 Hz, 2H), 1.31 - 1.21 (m, 1H), 0.69 - 0.62 (m, 2H), 0.39-0.33 (m, 2H).

1-[3-Chloro-5-(cyclopropylmethoxy)-4-flourophenyl]-5-(2-methoxy-3-pyridyl)-3-(3-pyridyl)pyrimidine-2,4-dione (35)

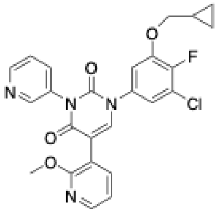

Into a 100 mL round-bottom flask was placed in order 1-[3-Chloro-5-(cyclopropylmethoxy)-4-fluorophenyl]-5-(2-methoxy-3-pyridyl)pyrimidine-2,4-dione (150 mg, 0.360 mmol), 3-pyridylboronic acid (130 mg, 1.08 mmol), copper(II)acetate (100 mg, 0.540 mmol), DMSO (15 mL), and TMEDA (0.20 mL, 0.80 mmol), forming a deep blue solution. The reaction mixture was stirred open to the atmosphere at 60 °C for 18 h. LC–MS analysis confirmed complete conversion of the starting material. The reaction mixture was diluted with EtOAc (100 mL), water (200 mL), and were stirred vigorously. The layers were separated, and the aqueous phase was extracted with EtOAc (2 × 200 mL). The combined organic extracts were dried over anhydrous sodium sulfate, filtered, and concentrated under reduced pressure. The resulting crude material was moved forward without further purification (120 mg, 0.250 mmol, 68% yield).

1-[3-(Cyclopropylmethoxy)-5-flourophenyl]-5-(2-oxo-1H-pyridin-3-yl)-3-(3-pyridyl)pyrimidine-2,4-dione (36)

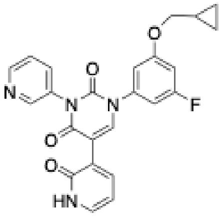

Into a 100 mL two-neck round-bottom flask under an argon atmosphere was placed 1-[3-(cyclopropylmethoxy)-5-fluorophenyl]-5-(2-methoxy-3-pyridyl)-3-(3-pyridyl)pyrimidine-2,4-dione (200 mg, 1.84 mmol), followed by anhydrous MeCN (15 mL). Sodium iodide (280 mg, 1.84 mmol) was added to the reaction mixture while stirring. Chlorotrimethylsilane (200 mg, 1.84 mmol) was added to the reaction mixture, forming a pale-yellow solution. The reaction mixture was heated to 60 °C for 6 h, after which LC–MS analysis indicated conversion to the desired product. The mixture was cooled to room temperature and diluted with EtOAc (250 mL) and 10% aqueous sodium thiosulfate (150 mL). After thorough mixing, the layers were separated, and the aqueous layer was extracted with EtOAc (3 × 250 mL). The combined organic extracts were washed with brine, dried over anhydrous sodium sulfate, filtered, and concentrated under reduced pressure. Purification via silica gel flash chromatography (EtOAc/hex, 7:3 to 9:1) afforded 1-[3-(cyclopropylmethoxy)-5-fluorophenyl]-5-(2-oxo-1H-pyridin-3-yl)-3-(3-pyridyl)pyrimidine-2,4-dione (112 mg, 0.250 mmol, 41% yield) as a white solid.

^{1}H NMR (600 MHz, DMSO) δ 11.89 (s, 1H), 8.71 (s, 1H), 8.61 (dd, *J* = 4.8, 1.6 Hz, 1H), 8.57 (d, *J* = 2.5 Hz, 1H), 8.04 (dd, *J* = 7.2, 2.1 Hz, 1H), 7.84 (d, *J* = 8.1 Hz, 1H), 7.57 (dd, *J* = 8.1, 4.8 Hz, 1H), 7.37 (s, 1H), 7.07 - 7.00 (m, 2H), 6.99-6.94 (m, 1H), 6.30 (t, *J* = 6.8 Hz, 1H), 3.88 (d, *J* = 7.1 Hz, 2H), 1.26-1.21 (m, 1H), 0.61 - 0.54 (m, 2H), 0.36 - 0.30 (m, 2H). **^{13}C NMR (151 MHz, DMSO)** δ 162.7 (d, *J* = 243.3 Hz: 163.4, 162.0), 161.2, 160.9, 160.2, 150.0, 149.4, 149.1, 143.5, 141.0, 139.9, 136.8, 134.3, 132.8, 124.0, 121.3, 110.0, 107.8, 106.4 (d, *J* = 24.7 Hz: 106.5, 106.3,) 105.1, 102.4 (d, *J* = 25.0 Hz: 102.5, 102.3) 73.0, 9.9, 3.1. **^{19}F NMR (400 MHz, $CDCl_3$) δ** -110.1. **HRMS:** (APCI+) $[M+H]^+$ calc for $C_{24}H_{20}FN_4O_4$, 447.1463, found 447.1458.

1-[3-Bromo-5-(cyclopropylmethoxy)phenyl]-5-(2-oxo-1H-pyridin-3-yl)-3-(3-pyridyl)pyrimidine-2,4-dione (37)

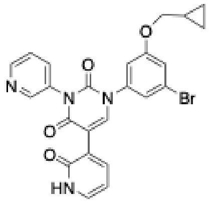

Into a 100 mL two-neck round-bottom flask under an argon atmosphere was placed 1-[3-bromo-5-(cyclopropylmethoxy)phenyl]-5-(2-methoxy-3-pyridyl)-3-(3-pyridyl)pyrimidine-2,4-dione (260 mg, 0.490 mmol), followed by anhydrous MeCN (15 mL). Sodium iodide (220 mg, 1.47 mmol) was added under stirring. Chlorotrimethylsilane (160 mg, 1.47 mmol) was added to the reaction mixture, forming a pale-yellow solution. The reaction mixture was heated to 60 °C for 6 h, after which LC–MS analysis indicated conversion to the desired product. The mixture was cooled to room temperature and diluted with EtOAc (250 mL) and 10% aqueous sodium thiosulfate (150 mL). After thorough mixing, the layers were separated, and the aqueous phase was extracted with EtOAc (3 × 250 mL). The combined organic layers were washed with brine, dried over anhydrous sodium sulfate, filtered, and concentrated under reduced pressure. Purification via silica gel flash chromatography (EtOAc/hex, 7:3 to 9:1) afforded 1-[3-bromo-5-(cyclopropylmethoxy)phenyl]-5-(2-oxo-1H-pyridin-3-yl)-3-(3-pyridyl)pyrimidine-2,4-dione (140 mg, 0.276 mmol, 56% yield) as a light-brown solid.

^{1}H NMR (600 MHz, DMSO) δ 11.89 (s, 1H), 8.70 (s, 1H), 8.61 (dd, *J* = 4.8, 1.5 Hz, 1H), 8.58 - 8.54 (m, 1H), 8.04 (dd, *J* = 7.1, 2.1 Hz, 1H), 7.84 (d, *J* = 8.1, 1H), 7.57 (d, *J* = 8.1 Hz, 2H), 7.41 - 7.34 (m, 2H), 7.28-7.25 (m, 1H), 7.20-7.16 (m, 1H), 6.31-6.28 (m, 1H), 3.89 (d, *J* = 7.0 Hz, 2H), 1.27 - 1.18 (m, 1H), 0.61 - 0.53 (m, 2H), 0.36 - 0.29 (m, 2H). **^{13}C NMR (151 MHz, DMSO)** δ 161.7, 160.9, 159.8, 149.6, 149.5, 149.1, 143.4, 141.3, 139.9, 136.8, 134.2, 132.7, 124.0, 121.9, 121.8, 121.3, 117.6, 113.0, 107.8, 105.1, 73.0, 9.9, 3.1. **HRMS:** (APCI+) $[M+H]^+$ Calc. for $C_{24}H_{20}BrN_4O_4$, 507.0662, found 507.0661.

1-[3-Chloro-5-(cyclopropylmethoxy)-4-flourophenyl]-5-(2-oxo-1H-pyridin-3-yl)-3-(3-pyridyl)pyrimidine-2,4-dione (38)

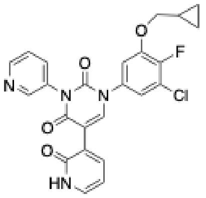

Into a 100 mL two-neck round-bottom flask under an argon atmosphere was added 1-[3-Chloro-5-(cyclopropylmethoxy)-4-fluorophenyl]-5-(2-methoxy-3-pyridyl)pyrimidine-2,4-dione (120 mg, 0.250 mmol), followed by anhydrous MeCN (10 mL). Sodium iodide (110 mg, 0.750 mmol) was added to the reaction mixture while stirring. Chlorotrimethylsilane (0.10 mL, 0.83 mmol) was added to the reaction mixture, forming a pale-yellow solution. The reaction mixture was heated to 60 °C for 6 h, after which LC–MS analysis indicated conversion to the desired product. The mixture was cooled to room temperature and diluted with EtOAc (250 mL) and 10% aqueous sodium thiosulfate (150 mL). After thorough mixing, the layers were separated, and the aqueous phase was extracted with EtOAc (3 × 250 mL). The combined organic layers were washed with brine, dried over anhydrous sodium sulfate, filtered, and concentrated under reduced pressure. Purification via silica gel flash chromatography (EtOAc/hex, 7:3 to 9:1) afforded 1-[3-Chloro-5-(cyclopropylmethoxy)-4-fluorophenyl]-5-(2-oxo-1H-pyridin-3-yl)-3-(3-pyridyl)pyrimidine-2,4-dione (40 mg, 0.083 mmol, 33% yield) as a white powder.

^{1}H NMR (800 MHz, DMSO) δ 11.89 (s, 1H), 8.65 (s, 1H), 8.61 (dd, *J* = 4.8, 1.6 Hz, 1H), 8.55 (d, *J* = 2.4 Hz, 1H), 8.00 (dd, *J* = 7.2, 2.1 Hz, 1H), 7.82 (ddd, *J* = 8.1, 2.5, 1.6 Hz, 1H), 7.57 (ddd, *J* = 8.1, 4.8, 0.7 Hz, 1H), 7.43 (ddd, *J* = 10.4, 6.3, 2.5 Hz, 2H), 7.37 (d, *J* = 6.4 Hz, 1H), 6.29 (t, *J* = 6.7 Hz, 1H), 3.96 (d, *J* = 7.2 Hz, 2H), 1.27 (pt, *J* = 7.5, 4.8 Hz, 1H), 0.62 - 0.56 (m, 2H), 0.37 - 0.32 (m, 2H). **^{13}C NMR (201 MHz, DMSO) δ** 161.6, 160.9, 149.6 (d, *J* = 10.6 Hz: 149.59, 149.54), 149.1, 147.4 (d, *J* = 249 Hz: 148.01, 146.77), 147.6 (d, *J* = 11.0 Hz), 146.8, 143.5, 140.0, 136.7, 135.6 (d, *J* = 4.3 Hz), 134.3, 132.6, 124.0, 121.4, 120.2 (d, *J* = 16.0 Hz), 120.0, 113.4, 107.8, 105.0, 74.2, 9.8, 3.1. **^{19}F NMR (400 MHz, $CDCl_3$) δ** -136.1. **HRMS:** (APCI+) $[M+H]^+$ calc for $C_{24}H_{19}FClN_4O_4$, 481.1073, found 481.1069

Scheme S2. Synthesis of compound 41-44

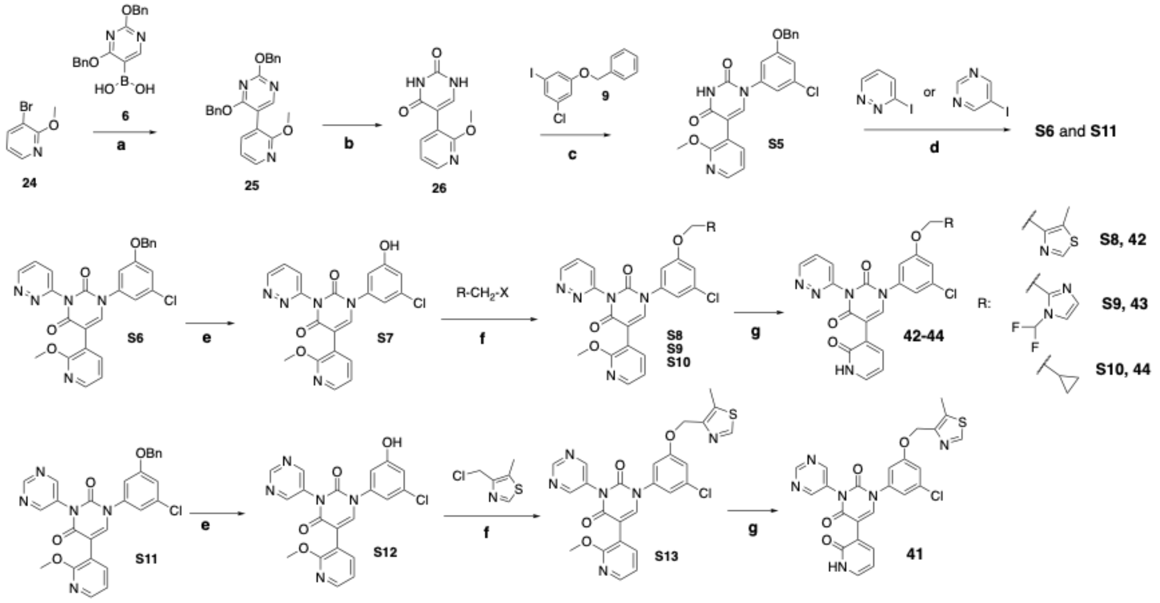

Reagents and conditions: a) $Pd(PPh_3)_4$, $NaHCO_3$, DME/water, 40%; b) 10% Pd/C, H_2, MeOH/THF (1:1), 98% c) CuI, K_3PO_4, N-(2-cyanophenyl)picolinamide, DMSO, 57%; d) CuI, K_3PO_4, N-(2-cyanophenyl)picolinamide, DMSO, 15%; e) 10% Pd/C, H_2, MeOH/THF (1:1), 90%; f) K_2CO_3, R-X, DMF; g) TMS-Cl, NaI, MeCN.

1-(3-(Benzyloxy)-5-chlorophenyl)-5-(2-methoxypyridin-3-yl)pyrimidine-2,4(1*H*,3*H*)-dione (S5)

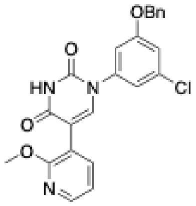

Into a two-neck round-bottom flask under an argon atmosphere was placed DMSO (70 mL), followed by 5-(2-methoxy-3-pyridyl)-1H-pyrimidine-2,4-dione (4.00 g, 18.3 mmol), 1-benzyloxy-3-Chloro-5-iodobenzene (7.55 g, 21.9 mmol), N-(2-cyanophenyl)picolinamide (0.490 g, 2.19 mmol), and potassium phosphate (8.13 g, 38.3 mmol), forming a brown suspension. The mixture was degassed by bubbling argon through the solution for 10 min, after which copper(I)iodide (0.350 g, 1.82 mmol) was added under a flow of argon. The reaction mixture was heated to 60 °C, during which the color turned very dark green. The reaction was stirred under argon at this temperature for three days. The mixture was diluted with EtOAc (200 mL) and water (500 mL). A suspension formed, which was broken by passing the entire mixture through a celite pad. The organic layer was separated, and the aqueous layer was extracted with EtOAc (2 × 200 mL). The combined organic layers were dried over anhydrous magnesium sulfate and filtered.

The solvent was removed in vacuo, and DCM was added to wet the residue. Hexanes were then added to precipitate the majority of the product (2.20 g). The precipitate contained residual green coloration, which was removed by washing with methanol. The solid was dried overnight on filter paper, and NMR analysis was performed after complete evaporation of hexanes and methanol. The crude material was purified by column chromatography (EtOAc/hex) to afford 1-(3-benzyloxy-5-chloro-phenyl)-5-(2-methoxy-3-pyridyl)pyrimidine-2,4-dione (3.30 g, 7.57 mmol, 42% yield) accompanied by an additional ~1 g of white solid.

^{1}H NMR (600 MHz, DMSO-*d6*) δ 11.66 (s, 1H), 8.11 (dd, *J* = 5.0, 1.9 Hz, 1H), 7.84 (d, *J* = 1.5 Hz, 1H), 7.69 (dd, *J* = 7.3, 1.9 Hz, 1H), 7.41 (d, *J* = 6.9 Hz, 2H), 7.38 - 7.34 (m, 2H), 7.33-7.29 (m, 1H), 7.21 (t, *J* = 1.8 Hz, 1H), 7.17 (d, *J* = 1.8 Hz, 2H), 6.99 (dd, *J* = 7.3, 5.0 Hz, 1H), 5.12 (s, 2H), 3.78 (s, 3H).

1-(3-(Benzyloxy)-5-chlorophenyl)-5-(2-methoxypyridin-3-yl)-3-(pyridazin-3-yl)pyrimidine-2,4(1*H*,3*H*)-dione (S6)

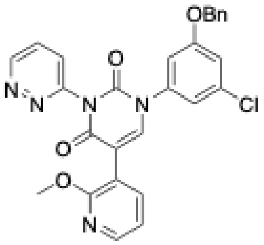

Into a two-neck round-bottom flask under an argon atmosphere was placed DMSO (5 mL), followed by 1-(3-benzyloxy-5-chloro-phenyl)-5-(2-methoxy-3-pyridyl)pyrimidine-2,4-dione (0.20 g, 0.46 mmol), 3-bromopyridazine (0.090 g, 0.55 mmol), N-(2-cyanophenyl)picolinamide (0.01 g, 0.06 mmol), and potassium phosphate (0.20 g, 0.96 mmol), forming a brown suspension. The mixture was degassed by bubbling argon through the solution for 10 min, after which copper(I)iodide (0.01 g, 0.05 mmol) was added under a flow of argon. The reaction mixture was heated to 60 °C, during which it turned very dark green, and was stirred under argon overnight. The mixture was diluted with EtOAc (200 mL) and water (500 mL). The organic layer was separated, and the aqueous phase was extracted with EtOAc (2 × 200 mL). The combined organic extracts were dried over anhydrous magnesium sulfate and filtered. The crude material was purified via column chromatography (EtOAc/hex) to afford 1-(3-benzyloxy-5-chloro-phenyl)-5-(2-methoxy-3-pyridyl)-3-(pyridazin-3-yl)pyrimidine-2,4-dione (15 mg, 0.030 mmol, 6% yield) as a brown liquid.

^{1}H NMR (400 MHz, MeOD) δ 9.32 (dd, *J* = 4.6, 1.8 Hz, 1H), 8.16 (dd, *J* = 5.0, 1.9 Hz, 1H), 8.00 (s, 1H), 7.98 - 7.92 (m, 2H), 7.82 (dd, *J* = 7.4, 1.9 Hz, 1H), 7.52 - 7.42 (m, 2H), 7.42 - 7.31 (m, 3H), 7.24 (t, *J* = 1.8 Hz, 1H), 7.19 (dt, *J* = 8.2, 2.2 Hz, 2H), 7.03 (dd, *J* = 7.4, 5.0 Hz, 1H), 5.15 (s, 2H), 3.95 (s, 3H).

1-(3-Chloro-5-hydroxyphenyl)-5-(2-methoxypyridin-3-yl)-3-(pyridazin-3-yl)pyrimidine-2,4(1*H*,3*H*)-dione (S7)

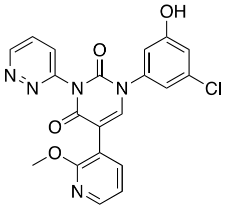

Into a two-neck round-bottom flask under an argon atmosphere was placed 1-(3-benzyloxy-5-chloro-phenyl)-5-(2-methoxy-3-pyridyl)-3-(pyridazin-3-yl)pyrimidine-2,4-dione (0.640 g, 1.25 mmol), followed by methanol (15 mL) and THF (15 mL). Upon heating, the starting material dissolved and remained in solution upon cooling. Pd/C (0.13 g, 0.12 mmol) was then added, and a hydrogen-filled balloon was fitted to the flask. The flask was purged with hydrogen gas, and the reaction mixture was stirred at 40 °C. After 3 h, LC–MS analysis indicated complete conversion of the starting material. The reaction mixture was filtered through a pad of celite and concentrated in vacuo to afford 1-(3-Chloro-5-hydroxy-phenyl)-5-(2-methoxy-3-pyridyl)-3-(pyridazin-3-yl)pyrimidine-2,4-dione (0.15 g, 0.35 mmol, 28% yield) as a white solid.

^{1}H NMR (400 MHz, MeOD) δ 9.32 (dd, *J* = 4.6, 1.9 Hz, 1H), 8.15 (dd, *J* = 5.0, 1.9 Hz, 1H), 8.00 (s, 1H), 7.98 - 7.90 (m, 2H), 7.87 - 7.74 (m, 1H), 7.08 (t, *J* = 1.9 Hz, 1H), 7.02 (dd, *J* = 7.4, 5.0 Hz, 1H), 6.92 (h, *J* = 2.2 Hz, 2H), 3.96 (s, 3H), 1.17 (d, *J* = 6.1 Hz, 1H).

1-(3-Chloro-5-((5-methylthiazol-4-yl)methoxy)phenyl)-5-(2-methoxypyridin-3-yl)-3-(pyridazin-3-yl)pyrimidine-2,4(1*H*,3*H*)-dione (S8)

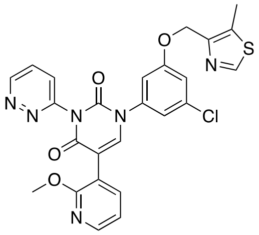

1-(3-Chloro-5-hydroxy-phenyl)-5-(2-methoxy-3-pyridyl)-3-(pyridazin-3-yl)pyrimidine-2,4-dione (0.15 g, 0.35 mmol) was dissolved in DMF (10 mL), and potassium carbonate (147 mg, 1.06 mmol) was added. Immediately thereafter, 4-(chloromethyl)-5-methylthiazole hydrochloride (85 mg, 0.46 mmol) was added, the flask was flushed with argon, and the reaction mixture was stirred at 60 °C overnight. Completion of the reaction was confirmed by LC–MS and TLC analysis. The mixture was diluted with ethyl acetate and water, and the layers were separated. The organic layer was collected, dried over sodium sulfate, filtered, and concentrated under reduced pressure. The crude product was purified by silica flash chromatography (DCM/MeOH, 97:3) to afford 150 mg of **S8** with 79% yield. ^{1}H NMR spectra were recorded for characterization.

^{1}H NMR (400 MHz, MeOD) δ 9.33 (dd, *J* = 4.6, 1.8 Hz, 1H), 8.81 (s, 1H), 8.16 (dd, *J* = 5.1, 1.9 Hz, 1H), 8.04 (s, 1H), 8.02 - 7.92 (m, 2H), 7.84 (dd, *J* = 7.4, 1.9 Hz, 1H), 7.29 - 7.25 (m, 1H), 7.25 - 7.20 (m, 2H), 7.03 (dd, *J* = 7.4, 5.1 Hz, 1H), 5.24 (s, 2H), 3.96 (s, 3H), 2.56 (s, 3H).

1-(3-Chloro-5-((1-(difluoromethyl)-1*H*-imidazol-2-yl)methoxy)phenyl)-5-(2-methoxypyridin-3-yl)-3-(pyridazin-3-yl)pyrimidine-2,4(1*H*,3*H*)-dione (S9)

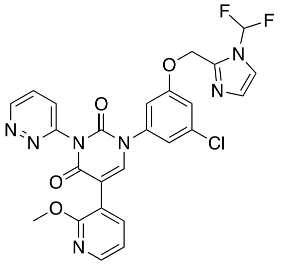

1-(3-Chloro-5-hydroxy-phenyl)-5-(2-methoxy-3-pyridyl)-3-(pyridazin-3-yl)pyrimidine-2,4-dione (155 mg, 0.370 mmol) was dissolved in dry DMF (10 mL), after which potassium carbonate (150 mg, 1.08 mmol) was added. To the reaction mixture, 2-(chloromethyl)-1-(difluoromethyl)imidazole (79 mg, 0.48 mmol) was added, and the mixture was stirred at 60 °C overnight. The following day, completion of the reaction was confirmed by TLC and LC–MS analysis. The mixture was worked up with ethyl acetate and water, and the organic layer was collected. The crude product was adsorbed onto silica gel and purified by column chromatography (DCM/MeOH), gradually increasing the methanol content to 10% to afford 65 mg (0.12 mmol) of **S9** with 32% yield.

^{1}H NMR (400 MHz, MeOD) δ 9.32 (dd, *J* = 4.6, 1.8 Hz, 1H), 8.16 (dd, *J* = 5.0, 1.9 Hz, 1H), 8.04 (s, 1H), 8.01 - 7.91 (m, 2H), 7.84 (dd, *J* = 7.4, 1.9 Hz, 1H), 7.86 (0.3H), 7.72 (0.57H), 7.57 (0.3H) (CH-F_2, *J:* 42 Hz, 1H), 7.57 (d, *J* = 1.7 Hz, 1H), 7.32 (t, *J* = 1.8 Hz, 1H), 7.28-7.24 (m, 2H), 7.14 (d, *J* = 1.6 Hz, 1H), 7.03 (dd, *J* = 7.4, 5.0 Hz, 1H), 5.37 (s, 2H), 3.95 (s, 3H). **^{19}F NMR (376 MHz, MeOD)** δ -94.43, -94.59.

1-(3-Chloro-5-(cyclopropylmethoxy)phenyl)-5-(2-methoxypyridin-3-yl)-3-(pyridazin-3-yl)pyrimidine-2,4(1*H*,3*H*)-dione (S10)

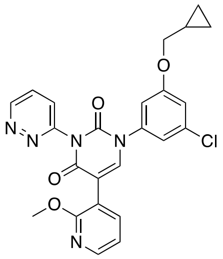

1-(3-Chloro-5-hydroxy-phenyl)-5-(2-methoxy-3-pyridyl)-3-(pyridazin-3-yl)pyrimidine-2,4-dione (145 mg, 0.340 mmol) was dissolved in DMF (10 mL), and potassium carbonate (208 mg, 1.51 mmol) was added. Immediately thereafter, (bromomethyl)cyclopropane (0.10 mL, 0.72 mmol) was added, the flask was

flushed with argon, and the reaction mixture was stirred at 60 °C overnight. Completion of the reaction was confirmed by LC–MS and TLC analysis. The mixture was diluted with EtOAc and water, and the layers were separated. The organic extracts were collected, dried over sodium sulfate, filtered, and concentrated under reduced pressure. The crude product was purified via silica flash chromatography (DCM/MeOH, 97:3) to afford 50 mg (0.10 mmol) of **S10** with 15% yield.

^{1}H NMR (400 MHz, MeOD) δ 9.31 (dd, *J* = 4.6, 1.8 Hz, 1H), 8.15 (dd, *J* = 5.0, 1.9 Hz, 1H), 8.01 (s, 1H), 7.99 - 7.88 (m, 2H), 7.82 (dd, *J* = 7.4, 1.9 Hz, 1H), 7.21-7.18 (m, 1H), 7.10-7.06 (m, 2H), 7.01 (dd, *J* = 7.4, 5.0 Hz, 1H), 3.95 (s, 3H), 3.88 (d, *J* = 7.0 Hz, 2H), 1.34 - 1.20 (m, 1H), 0.68 - 0.57 (m, 2H), 0.41 - 0.33 (m, 2H).

1-(3-Chloro-5-(cyclopropylmethoxy)phenyl)-5-(2-methoxypyridin-3-yl)-3-(pyridazin-3-yl)pyrimidine-2,4(1*H*,3*H*)-dione (42)

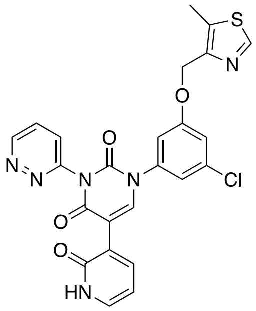

Into a 100 mL two-neck round-bottom flask under an argon atmosphere was placed 1-[3-Chloro-5-[(5-methylthiazol-4-yl)methoxy]phenyl]-5-(2-methoxy-3-pyridyl)-3-(pyridazin-3-yl)pyrimidine-2,4-dione (0.03 g, 0.06 mmol), followed by dry MeCN (10 mL), forming a clear solution. Sodium iodide (0.030 g, 0.17 mmol) was added, immediately followed by chlorotrimethylsilane (0.020 mL, 0.17 mmol), yielding a pale-yellow solution. The mixture was heated to 60 °C for 6 h, after which LC–MS analysis confirmed complete consumption of the starting material. After cooling, the reaction mixture was diluted with EtOAc (250 mL), brine (250 mL), and 10% aqueous sodium thiosulfate (150 mL). After thorough mixing, the layers were separated, and the aqueous phase was extracted with EtOAc (3 × 250 mL). The combined organic fractions were washed with brine, dried over anhydrous magnesium sulfate, filtered, and concentrated in vacuo. Purification by silica gel column chromatography (EtOAc/hex, followed by 3% MeOH/EtOAc) afforded 1-[3-Chloro-5-[(5-methylthiazol-4-yl)methoxy]phenyl]-5-(2-oxo-1H-pyridin-3-yl)-3-(pyridazin-3-yl)pyrimidine-2,4-dione (25 mg, 0.049 mmol, 86% yield) as a white powder.

^{1}H NMR (600 MHz, DMSO-*d6*) δ 11.95 (s, 1H), 9.36 (dd, *J* = 4.8, 1.6 Hz, 1H), 8.91 (s, 1H), 8.76 (s, 1H), 8.03 - 7.97 (m, 2H), 7.94 (dd, *J* = 8.6, 1.6 Hz, 1H), 7.40 (dd, *J* = 6.4, 2.1 Hz, 1H), 7.33 (d, *J* = 2.0 Hz, 2H), 7.29 (t, *J* = 2.1 Hz, 1H), 6.31 (dd, *J* = 7.1, 6.4 Hz, 1H), 5.23 (s, 2H), 2.51 (s, 3H). **^{13}C NMR (151 MHz, DMSO-*d6*)** δ 162.05, 161.36, 159.96, 154.54, 152.81, 151.55, 149.67, 147.47, 144.42, 141.11, 140.52, 134.98, 134.32, 133.78, 130.03, 129.23, 121.58, 119.95, 115.61, 113.52, 108.74, 105.57, 64.36, 11.16. **HRMS calcd for :**$C_{24}H_{18}O_4N_6{}^{35}Cl^{32}S$: 521.07933, found:521.07932.

1-(3-Chloro-5-((1-(difluoromethyl)-1*H*-imidazol-2-yl)methoxy)phenyl)-5-(2-oxo-1,2-dihydropyridin-3-yl)-3-(pyridazin-3-yl)pyrimidine-2,4(1*H*,3*H*)-dione (43)

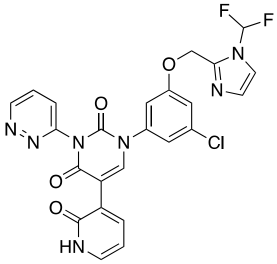

Into a 100 mL two-neck round-bottom flask under an argon atmosphere was placed 1-[3-Chloro-5-[[1-(difluoromethyl)imidazol-2-yl]methoxy]phenyl]-5-(2-methoxy-3-pyridyl)-3-(pyridazin-3-yl)pyrimidine-2,4-dione (0.080 g, 0.15 mmol), followed by dry MeCN (10 mL), forming a clear solution. Sodium iodide (0.070 g, 0.45 mmol) was added, immediately followed by chlorotrimethylsilane (0.060 mL, 0.45 mmol), giving a pale-yellow solution. The reaction mixture was heated to 60 °C for 6 h, after which LC–MS analysis confirmed complete consumption of the starting material. After cooling, the mixture was diluted with EtOAc (250 mL), brine (250 mL), and 10% aqueous sodium thiosulfate (150 mL). After thorough mixing, the layers were separated, and the aqueous phase was extracted with EtOAc (3 × 250 mL). The combined organic layers were washed with brine, dried over anhydrous magnesium sulfate, filtered, and concentrated in vacuo. Purification via silica gel chromatography (EtOAc/hex, followed by 3% MeOH/EtOAc) afforded 1-[3-Chloro-5-[[1-(difluoromethyl)imidazol-2-yl]methoxy]phenyl]-5-(2-oxo-1H-pyridin-3-yl)-3-(pyridazin-3-yl)pyrimidine-2,4-dione (65 mg, 0.12 mmol, 80% yield) as a white powder.

^{1}H NMR (600 MHz, DMSO-*d6)* δ 11.95 (s, 1H), 9.36 (dd, *J* = 4.8, 1.6 Hz, 1H), 8.75 (s, 1H), 8.05 - 7.79 (m, 4H), 7.70 (d, *J* = 1.6 Hz, 1H), 7.43 - 7.23 (m, 4H), 7.14 (d, *J* = 1.6 Hz, 1H), 6.31 (t, *J* = 6.7 Hz, 1H), 5.38 (s, 2H). **^{13}C NMR (151 MHz, DMSO-*d6)*** δ 162.06, 161.36, 159.13, 154.54, 152.81, 149.65, 144.37, 142.40, 141.11, 140.54, 134.98, 134.33, 130.03, 129.82, 129.24, 121.54, 120.56, 118.13, 115.70, 113.73, 110.53, 108.88 (t, C-F_2, *J:* 248 Hz; 110.53, 108.88, 107.24), 108.75, 105.59, 62.84. **^{19}F NMR (376 MHz, DMSO-*d6)*** δ -91.87, -92.02. **HRMS calcd for** $C_{24}H_{17}O_4N_7{}^{35}ClF_2$: 540.09931, found:540.09936.

1-(3-Chloro-5-(cyclopropylmethoxy)phenyl)-5-(2-oxo-1,2-dihydropyridin-3-yl)-3-(pyridazin-3-yl)pyrimidine-2,4(1*H*,3*H*)-dione (44)

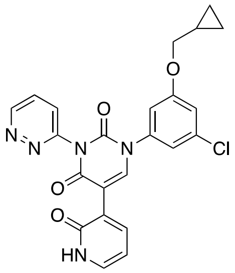

Into a 100 mL two-neck round-bottom flask under an argon atmosphere was placed 1-[3-Chloro-5-(cyclopropylmethoxy)phenyl]-5-(2-methoxy-3-pyridyl)-3-(pyridazin-3-yl)pyrimidine-2,4-dione (0.050 g,

0.10 mmol), followed by dry MeCN (10 mL), forming a clear solution. Sodium iodide (0.050 g, 0.31 mmol) was added, immediately followed by chlorotrimethylsilane (0.040 mL, 0.31 mmol), yielding a pale-yellow solution. The reaction mixture was heated to 60 °C for 6 h, after which LC–MS analysis confirmed complete consumption of the starting material. After cooling, the mixture was diluted with EtOAc (250 mL), brine (250 mL), and 10% aqueous sodium thiosulfate (150 mL). After thorough mixing, the phases were separated, and the aqueous layer was extracted with EtOAc (3 × 250 mL). The combined organic extracts were washed with brine, dried over anhydrous magnesium sulfate, filtered, and concentrated in vacuo. Purification by silica gel chromatography (EtOAc/hex, followed by 3% MeOH/EtOAc) afforded 1-[3-Chloro-5-(cyclopropylmethoxy)phenyl]-5-(2-oxo-1H-pyridin-3-yl)-3-(pyridazin-3-yl)pyrimidine-2,4-dione (30 mg, 0.070 mmol, 62% yield) as a white powder.

^{1}H NMR (600 MHz, DMSO-*d6*) δ 11.94 (s, 1H), 9.36 (d, *J* = 4.9 Hz, 1H), 8.76 (s, 1H), 8.02 (d, *J* = 7.2 Hz, 1H), 7.99 (dd, *J* = 8.6, 4.8 Hz, 1H), 7.93 (d, *J* = 8.6 Hz, 1H), 7.40 (d, *J* = 6.3 Hz, 1H), 7.30-7.26 (m, 1H), 7.20-1.15 (m, *J* = 14.1, 2.2 Hz, 2H), 6.30 (dd, *J* = 7.2, 6.4 Hz, 1H), 3.90 (d, *J* = 7.1 Hz, 2H), 1.28 - 1.19 (m, 1H), 0.62 - 0.54 (m, 2H), 0.36 - 0.31 (m, 2H). **^{13}C NMR (151 MHz, DMSO-*d6*)** δ 162.09, 161.43, 160.27, 154.55, 152.80, 149.65, 144.45, 141.18, 140.44, 135.06, 134.34, 130.02, 129.23, 121.47, 119.48, 115.48, 113.02, 108.65, 105.58, 73.52, 10.34, 3.61. **HRMS calcd for :** $C_{23}H_{19}O_4N_5{}^{35}Cl$: 464.11201, found:464.11174.

3-(3-(Benzyloxy)-5-chlorophenyl)-5-(2-methoxypyridin-3-yl)-2H-[1,5'-bipyrimidine]-2,6(3H)-dione (S11)

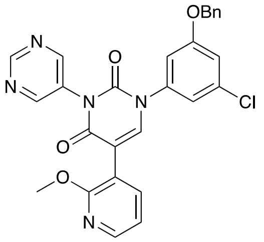

Into a two-neck round-bottom flask under an argon atmosphere was placed DMSO (5 mL), followed by 1-(3-benzyloxy-5-chloro-phenyl)-5-(2-methoxy-3-pyridyl)pyrimidine-2,4-dione (0.50 g, 1.2 mmol), 5-iodopyrimidine (0.280 g, 1.38 mmol), N-(2-cyanophenyl)picolinamide (0.030 g, 0.14 mmol), and potassium phosphate (0.510 g, 2.41 mmol), forming a brown suspension. The mixture was degassed by bubbling argon through the solution for 10 min, after which copper(I)iodide (0.020 g, 0.11 mmol) was added under a flow of argon. The reaction was heated to 60 °C, during which the mixture turned very dark green, and was stirred under argon overnight. The reaction mixture was diluted with EtOAc (200 mL) and water (500 mL). The organic layer was separated, and the aqueous layer was extracted with EtOAc (2 × 200 mL). The combined organic extracts were dried over anhydrous magnesium sulfate, filtered, and concentrated. The crude material was adsorbed onto a solid cartridge and purified via silica gel column chromatography (EtOAc/hex) to afford 1-(3-benzyloxy-5-chloro-phenyl)-5-(2-methoxy-3-pyridyl)-3-(pyrimidin-5-yl)pyrimidine-2,4-dione (100 mg, 0.200 mmol, 17% yield) as a brown liquid.

^{1}H NMR (400 MHz, Acetone-*d6*) δ 9.20 (s, 1H), 8.90 (s, 2H), 8.16 (dd, *J* = 5.0, 1.9 Hz, 1H), 8.12 (s, 1H), 7.87 (dd, *J* = 7.4, 1.9 Hz, 1H), 7.55 - 7.47 (m, 2H), 7.48 - 7.35 (m, 3H), 7.32 - 7.26 (m, 2H), 7.21 (d, *J* = 2.3 Hz, 1H), 7.03 (dd, *J* = 7.4, 5.0 Hz, 1H), 5.24 (s, 2H), 3.92 (s, 3H), 1.98 (s, 3H).

3-(3-Chloro-5-hydroxyphenyl)-5-(2-methoxypyridin-3-yl)-2H-[1,5'-bipyrimidine]-2,6(3H)-dione (S12)

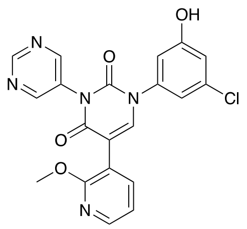

Into a two-neck round-bottom flask under an argon atmosphere was placed 1-(3-benzyloxy-5-chloro-phenyl)-5-(2-methoxy-3-pyridyl)-3-(pyrimidin-5-yl)pyrimidine-2,4-dione (0.26 g, 0.50 mmol), followed by methanol (15 mL) and THF (15 mL). Upon heating, the starting material dissolved and remained in solution upon cooling. Pd/C (0.05 g, 0.05 mmol) was then added, and a hydrogen-filled balloon was attached to the flask. The atmosphere in the flask was purged with hydrogen, and the reaction mixture was stirred at 40 °C. After 3 h, LC–MS analysis confirmed complete conversion of the starting material. The reaction mixture was filtered through a pad of celite and concentrated in vacuo to afford 1-(3-Chloro-5-hydroxy-phenyl)-5-(2-methoxy-3-pyridyl)-3-(pyrimidin-5-yl)pyrimidine-2,4-dione (0.15 g, 0.35 mmol, 71% yield) as a white solid, which was used without further purification.

^{1}H NMR (400 MHz, Acetone-*d6*) δ 9.50 (s, 1H), 9.21 (s, 1H), 8.92 (s, 2H), 8.15 (dd, *J* = 5.0, 1.9 Hz, 1H), 8.10 (s, 1H), 7.88 (dd, *J* = 7.4, 1.9 Hz, 1H), 7.17-7.13 (m, 1H), 7.09-7.05 (m, *J* = 2.0 Hz, 1H), 7.06 - 6.95 (m, 2H), 3.93 (s, 3H).

3-(3-Chloro-5-((5-methylthiazol-4-yl)methoxy)phenyl)-5-(2-methoxypyridin-3-yl)-2H-[1,5'-bipyrimidine]-2,6(3H)-dione (S13)

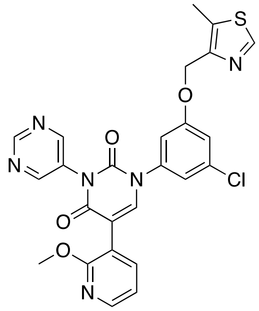

1-(3-Chloro-5-hydroxy-phenyl)-5-(2-methoxy-3-pyridyl)-3-(pyrimidin-5-yl)pyrimidine-2,4-dione (0.070 g, 0.17 mmol) was dissolved in DMF (10 mL). Potassium carbonate (69 mg, 0.50 mmol) and 4-(chloromethyl)-

5-methylthiazole hydrochloride (40 mg, 0.21 mmol) were added, and the reaction mixture was stirred at 60 °C overnight. Completion of the reaction was confirmed by LC–MS and TLC analysis. The mixture was diluted with ethyl acetate and water, and the layers were separated. The organic layers were combined, dried over sodium sulfate, filtered, and concentrated under reduced pressure. The crude product was purified by silica gel chromatography (DCM/MeOH, 97:3) to afford 70 mg of **S13** with 79% yield.

^{1}H NMR (600 MHz, CD_3OD) δ 9.19 - 9.11 (m, 1H), 8.88 - 8.81 (m, 2H), 8.74 (d, *J* = 3.5 Hz, 1H), 8.13-8.07 (m, 1H), 7.94 (d, *J* = 0.9 Hz, 1H), 7.80 - 7.72 (m, 1H), 7.22 - 7.17 (m, 1H), 7.17 - 7.14 (m, 2H), 7.01 - 6.92 (m, 1H), 5.16 (s, 2H), 3.89 (s, 3H), 2.49 (s, 3H).

3-(3-Chloro-5-((5-methylthiazol-4-yl)methoxy)phenyl)-5-(2-oxo-1,2-dihydropyridin-3-yl)-2H-[1,5'-bipyrimidine]-2,6(3H)-dione (41)

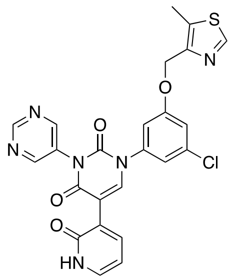

Into a 100 mL two-neck round-bottom flask under an argon atmosphere was placed 1-[3-Chloro-5-[(5-methylthiazol-4-yl)methoxy]phenyl]-5-(2-methoxy-3-pyridyl)-3-(pyrimidin-5-yl)pyrimidine-2,4-dione (0.070 g, 0.12 mmol), followed by dry MeCN (10 mL), forming a clear solution. Sodium iodide (0.060 g, 0.37 mmol) was added, immediately followed by chlorotrimethylsilane (0.050 mL, 0.37 mmol), giving a pale-yellow solution. The reaction mixture was heated to 60 °C for 6 h, after which LC–MS analysis confirmed complete consumption of the starting material. After cooling, the mixture was diluted with EtOAc (250 mL), brine (250 mL), and 10% aqueous sodium thiosulfate (150 mL). After thorough mixing, the layers were separated, and the aqueous phase was extracted with EtOAc (3 × 250 mL). The combined organic layers were washed with brine, dried over anhydrous magnesium sulfate, filtered, and concentrated in vacuo. Purification by silica gel chromatography (EtOAc/hex, followed by 3% MeOH/EtOAc) afforded 1-[3-Chloro-5-[(5-methylthiazol-4-yl)methoxy]phenyl]-5-(2-oxo-1H-pyridin-3-yl)-3-(pyrimidin-5-yl)pyrimidine-2,4-dione (21 mg, 0.041 mmol, 33% yield) as a white powder.

^{1}H NMR (800 MHz, DMSO-*d6*) δ 11.98 (s, 1H), 9.30 (s, 1H), 8.96 (d, *J* = 10.0 Hz, 3H), 8.79 (s, 1H), 8.08 (dd, *J* = 7.1, 2.1 Hz, 1H), 7.44 (dd, *J* = 6.4, 2.1 Hz, 1H), 7.43-7.35 (m, 2H), 7.32-7.28 (m, 1H), 6.38-6.34 (m, 1H), 5.29 (s, 2H), 2.56 (s, 3H). **^{13}C NMR (201 MHz, DMSO-*d6*)** δ 161.86, 161.35, 159.95, 158.17, 157.61, 157.55, 151.54, 149.78, 147.48, 144.09, 141.31, 140.55, 134.92, 134.32, 133.75, 132.01, 121.58, 119.86, 115.48, 113.48, 108.43, 105.55, 64.39, 11.16. **HRMS calcd for :** $C_{24}H_{18}O_4N_6{}^{35}Cl^{32}S$: 521.07933, found: 521.07988.

Scheme S3. Synthesis of compounds 45-49

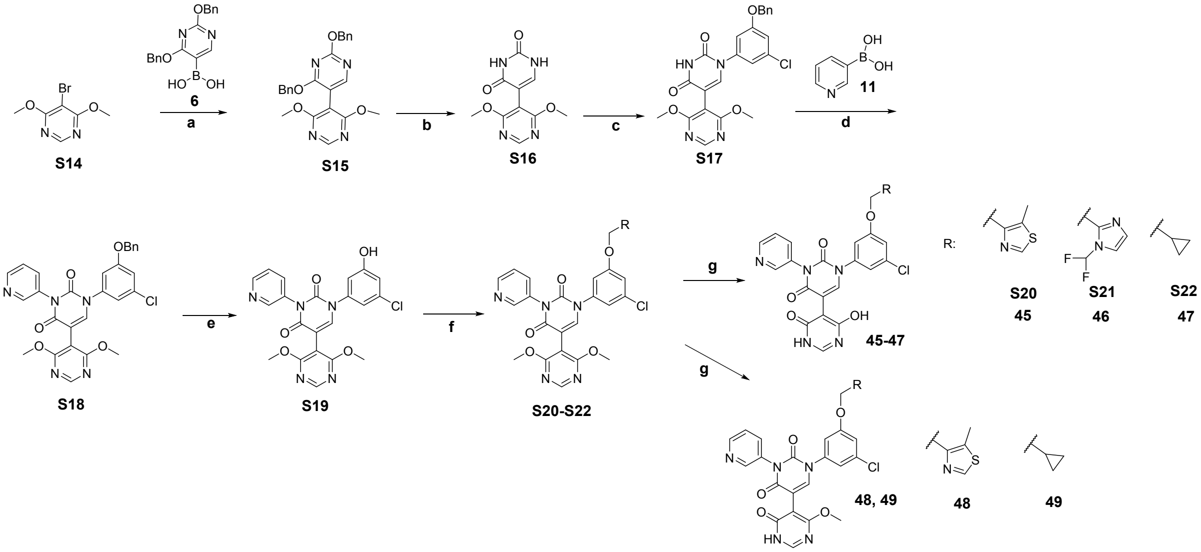

Reagents and conditions: a) $Pd(PPh_3)_4$, $NaHCO_3$, DME/water, 31%; b) 10% Pd/C, H_2, MeOH/THF (1:1), 81%); c) CuI, K_3PO_4, N-(2-cyanophenyl)picolinamide, DMSO, 27%; d) $Cu(OAc)_2$, TMEDA, DMSO, 75%; e) 10% Pd/C, H_2, MeOH/THF (1:1), 98%; f) K_2CO_3, R-X, DMF; g) TMS-Cl, NaI, MeCN.

2,4-Bis(benzyloxy)-4',6'-dimethoxy-5,5'-bipyrimidine (S15)

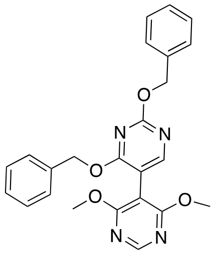

Into a 500 mL three-neck round-bottom flask fitted with a condenser and under an argon atmosphere was placed 5-bromo-4,6-dimethoxypyrimidine (3.15 g, 22.8 mmol), followed by monoglyme (150 mL), forming a clear solution. To this was added (2,4-dibenzyloxypyrimidin-5-yl)boronic acid (9.21 g, 27.4 mmol), which dissolved rapidly. A saturated aqueous sodium bicarbonate solution (143 mL, 114 mmol) was then added, resulting in the immediate formation of a white precipitate. The mixture was degassed by bubbling argon through the solution for 10 min, after which tetrakis(triphenylphosphine)palladium(0) (2.64 g, 2.28 mmol) was added in one portion. The reaction mixture was heated to reflux, during which most of the precipitate dissolved, giving a yellow solution. The mixture was refluxed for 6 h, during which the color deepened to a darker yellow/orange. After cooling, the reaction was poured into a separating funnel and diluted with EtOAc (300 mL) and water (200 mL). After vigorous shaking and phase separation, the organic layer was collected. The aqueous phase was extracted with EtOAc (2 × 200 mL). The combined organic extracts were dried over anhydrous magnesium sulfate and filtered through a silica gel plug, which was rinsed with ethyl acetate. The filtrate was concentrated in vacuo, and the crude product was purified by column

chromatography (EtOAc/hex) to afford 2,4-dibenzyloxy-5-(4,6-dimethoxypyrimidin-5-yl)pyrimidine (3.00 g, 6.97 mmol, 31% yield).

^{1}H NMR (400 MHz, MeOD) δ 8.43 (d, *J* = 1.5 Hz, 1H), 8.16 (d, *J* = 1.6 Hz, 1H), 7.51 - 7.44 (m, 2H), 7.43 - 7.26 (m, 8H), 5.46 (s, 2H), 5.44 (s, 2H), 3.88 (d, *J* = 1.5 Hz, 6H).

4',6'-Dimethoxy-[5,5'-bipyrimidine]-2,4(1*H*,3*H*)-dione (S16)

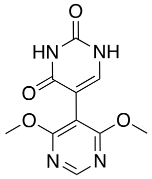

Into a 1L three-neck round-bottom flask containing methanol (300 mL) and THF (300 mL) was placed 2,4-dibenzyloxy-5-(4,6-dimethoxypyrimidin-5-yl)pyrimidine (3.21 g, 7.45 mmol), forming a clear solution. The flask was purged with argon, and palladium on carbon (0.790 g, 7.45 mmol) was added in one portion. A hydrogen-filled balloon was attached, and the reaction vessel was purged with hydrogen. The reaction mixture was stirred at 45 °C for 36 h, after which LC–MS analysis confirmed complete conversion of the starting material. The reaction mixture was filtered through a Celite pad and concentrated in vacuo to afford 5-(4,6-dimethoxypyrimidin-5-yl)-1H-pyrimidine-2,4-dione (1.50 g, 6.00 mmol, 81% yield) as a white solid. Approximately half of the product co-precipitated with palladium on the filter. Attempts to recover additional material by washing the palladium residue with methanol were unsuccessful. Ultimately, the palladium-containing solid was diluted with celite and purified using reverse-phase column chromatography (water/acetonitrile).
(*Note: Do not attempt purification by normal-phase silica chromatography; the compound precipitates in the column and causes significant obstruction).*

^{1}H NMR (400 MHz, DMSO-*d6*) δ 11.23 (s, 1H), 8.49 (s, 1H), 7.44 (s, 1H), 6.77 (s, 1H), 3.86 (s, 6H).

1-(3-(Benzyloxy)-5-chlorophenyl)-4',6'-dimethoxy-[5,5'-bipyrimidine]-2,4(1*H*,3*H*)-dione (S17)

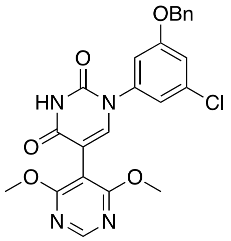

Into a two-neck round-bottom flask under argon was placed DMSO (10 mL), followed by 5-(4,6-dimethoxypyrimidin-5-yl)-1H-pyrimidine-2,4-dione (0.50 g, 2.0 mmol), 1-benzyloxy-3-Chloro-5-iodobenzene (0.83 g, 2.4 mmol), N-(2-cyanophenyl)picolinamide (0.050 g, 0.24 mmol), and potassium

phosphate (0.89 g, 4.2 mmol), forming a brown suspension. The mixture was degassed by bubbling argon through the solution for 10 min, after which copper(I)iodide (38 mg, 0.20 mmol) was added under a stream of argon. The reaction mixture was heated at 60 °C, during which the color gradually turned very dark green. The reaction was stirred under argon at this temperature for three days. The mixture was then diluted with EtOAc (200 mL) and water (500 mL). A thick suspension formed and was broken by pulling the entire mixture through a celite pad. The organic layer was separated, and the aqueous layer was extracted with EtOAc (2 × 200 mL). The combined organic extracts were dried over anhydrous magnesium sulfate and filtered. The solvent was removed in vacuo, and DCM was added to wet the residue. Hexanes were then added to precipitate the majority of the product (2.20 g). The solid exhibited a green tint, which was removed by washing thoroughly with methanol. The product was dried on filter paper overnight, and NMR was taken after evaporation of residual solvents. The crude material was finally purified by flash column chromatography (EtOAc/Hexane) to afford 1-(3-benzyloxy-5-chloro-phenyl)-5-(4,6-dimethoxypyrimidin-5-yl)pyrimidine-2,4-dione (0.25 g, 0.54 mmol, 27% yield) as an off-white solid (an additional ~1 g of less pure solid was also obtained during workup).

^{1}H NMR (400 MHz, MeOD) δ 8.44 (s, 1H), 7.67 (s, 1H), 7.49 - 7.43 (m, 2H), 7.42 - 7.28 (m, 3H), 7.17 - 7.04 (m, 3H), 5.14 (s, 2H), 3.99 - 3.90 (m, 6H).

1-(3-(Benzyloxy)-5-chlorophenyl)-4',6'-dimethoxy-3-(pyridin-3-yl)-[5,5'-bipyrimidine]-2,4(1*H*,3*H*)-dione (S18)

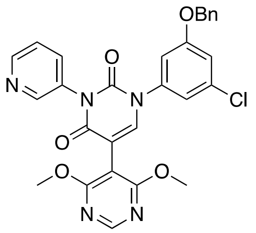

Into a 500 mL round-bottom flask was placed 1-(3-benzyloxy-5-chloro-phenyl)-5-(4,6-dimethoxypyrimidin-5-yl)pyrimidine-2,4-dione (0.25 g, 0.54 mmol), followed by 3-pyridylboronic acid (0.20 g, 1.6 mmol), copper(II)acetate (0.15 g, 0.81 mmol), and DMSO (20 mL). Finally, TMEDA (0.160 mL, 1.08 mmol) was added, forming a deep blue solution. The reaction mixture was stirred open to the atmosphere at 60 °C for 18 h, after which LCMS analysis indicated complete consumption of the starting material and formation of the desired product. The reaction mixture was diluted with EtOAc (200 mL) and water (300 mL) and mixed vigorously. After phase separation, the aqueous layer was extracted with EtOAc (2 × 200 mL). The combined organic layers were dried over anhydrous magnesium sulfate, filtered, and concentrated in vacuo to afford 1-(3-benzyloxy-5-chloro-phenyl)-5-(4,6-dimethoxypyrimidin-5-yl)-3-(3-pyridyl)pyrimidine-2,4-dione (0.22 g, 0.40 mmol, 75% yield) as a solid.

^{1}H NMR (400 MHz, MeOD) δ 8.65 - 8.57 (m, 2H), 8.44 (d, *J* = 3.7 Hz, 1H), 8.13 - 8.07 (m, 1H), 8.02 (dd, *J* = 4.3, 1.8 Hz, 1H), 7.91 (dd, *J* = 8.2, 2.4 Hz, 1H), 7.84 (s, 1H), 7.61 (dd, *J* = 8.2, 5.0 Hz, 1H), 7.44 (d, *J* = 1.2 Hz,

1H), 7.41 - 7.38 (m, 1H), 7.28 (d, *J* = 0.9 Hz, 1H), 7.23-7.19 (m, 1H), 7.17 (d, *J* = 1.8 Hz, 2H), 5.14 (s, 2H), 3.99 (s, 6H).

1-(3-Chloro-5-hydroxyphenyl)-4',6'-dimethoxy-3-(pyridin-3-yl)-[5,5'-bipyrimidine]-2,4(1*H*,3*H*)-dione (S19)

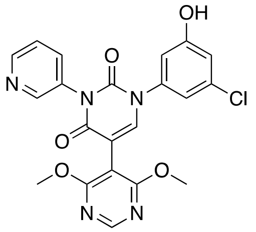

Into a two-neck round-bottom flask containing argon was placed 1-(3-benzyloxy-5-chloro-phenyl)-5-(4,6-dimethoxypyrimidin-5-yl)-3-(3-pyridyl)pyrimidine-2,4-dione (0.22 g, 0.40 mmol), followed by methanol (100 mL) and THF (100 mL). To the resulting solution was added Pd/C (0.04 g, 0.04 mmol), and a balloon filled with hydrogen gas was attached. The reaction vessel was purged with hydrogen, and the mixture was stirred at room temperature. After 3 h, LC-MS analysis confirmed complete consumption of the starting material. The reaction mixture was filtered through a pad of celite, and the filtrate was concentrated in vacuo to afford 1-(3-Chloro-5-hydroxy-phenyl)-5-(4,6-dimethoxypyrimidin-5-yl)-3-(3-pyridyl)pyrimidine-2,4-dione (0.18 g, 0.40 mmol, 98% yield) as a white solid.

^{1}H NMR (400 MHz, DMSO-*d6*) δ 10.42 (s, 1H), 8.64 - 8.55 (m, 2H), 8.52 (s, 1H), 8.06 (s, 1H), 7.87 (d, *J* = 8.1 Hz, 1H), 7.56 (dd, *J* = 4.8, 0.8 Hz, 1H), 7.13-7.09 (m, 1H), 6.95-6.91 (m, 1H), 6.93-6.88 (m, 1H), 3.91 (s, 6H).

1-(3-Chloro-5-((5-methylthiazol-4-yl)methoxy)phenyl)-4',6'-dimethoxy-3-(pyridin-3-yl)-[5,5'-bipyrimidine]-2,4(1*H*,3*H*)-dione (S20)

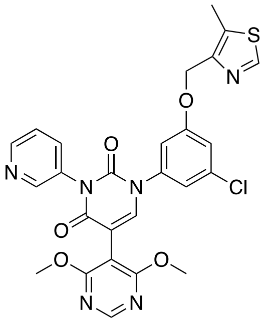

1-(3-Chloro-5-hydroxy-phenyl)-5-(4,6-dimethoxypyrimidin-5-yl)-3-(3-pyridyl)pyrimidine-2,4-dione (0.17 g, 0.37 mmol) was dissolved in DMF (10 mL). To this solution were added potassium carbonate (155 mg, 1.12 mmol) and 4-(chloromethyl)-5-methylthiazole hydrochloride (90 mg, 0.49 mmol). The reaction mixture was stirred at 60 °C overnight under argon. Completion of the reaction was confirmed by LC-MS and TLC. The mixture was then diluted with EtOAc and water, and the layers were separated. The organic phase was collected, washed, dried, filtered, and concentrated in vacuo. The crude product was purified by silica gel column chromatography (DCM:MeOH 97:3) to afford 110 mg (0.19 mmol) of **S20** with 52% yield. ^{1}H NMR spectra were recorded for characterization.

^{1}H NMR (400 MHz, MeOD) δ 8.81 (s, 1H), 8.73 - 8.55 (m, 2H), 8.45 (s, 1H), 7.94-7.89 (m, 1H), 7.88 (s, 1H), 7.61 (dd, *J* = 8.2, 4.9 Hz, 1H), 7.26-7.21 (m, 1H), 7.21 (d, *J* = 1.9 Hz, 2H), 5.23 (s, 2H), 3.99 (s, 6H), 2.55 (s, 3H).

1-(3-Chloro-5-((1-(difluoromethyl)-1*H*-imidazol-2-yl)methoxy)phenyl)-4',6'-dimethoxy-3-(pyridin-3-yl)-[5,5'-bipyrimidine]-2,4(1*H*,3*H*)-dione (S21)

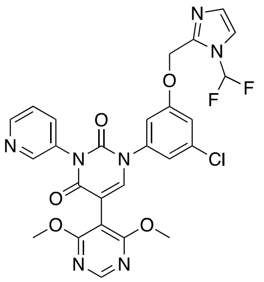

1-(3-Chloro-5-hydroxy-phenyl)-5-(4,6-dimethoxypyrimidin-5-yl)-3-(3-pyridyl)pyrimidine-2,4-dione (110 mg, 0.240 mmol) was dissolved in DMF (10 mL). To this solution were added 2-(chloromethyl)-1-(difluoromethyl)imidazole (53 mg, 0.32 mmol) and potassium carbonate (67 mg, 0.48 mmol). The reaction mixture was stirred at 60 °C overnight. The next day, the mixture was diluted with ethyl acetate and water, and the layers were separated. The organic phase was dried over anhydrous magnesium sulfate, filtered, and concentrated in vacuo. The crude residue was adsorbed onto silica (solid-phase loading) and purified by flash chromatography using DCM/MeOH, ramping the methanol content to 10% over 40 minutes. The product eluted very early, likely co-eluting with residual DMF. A second purification was therefore performed on a fresh silica cartridge using hexanes/EtOAc, which improved separation to afford 90 mg of **S21** with 64% yield.

^{1}H NMR (400 MHz, MeOD) δ 8.53 - 8.45 (m, 2H), 8.33 (s, 1H), 7.80 (dd, *J* = 8.2, 2.5 Hz, 1H), 7.75 (s, 1H), 7.59 (s, 1H), 7.50 (dd, *J* = 4.9, 0.8 Hz, 1H), 7.46-7.42 (m, 1H), 7.20-7.15 (m, 1H), 7.12 (dd, *J* = 1.8, 0.7 Hz, 2H), 7.01 (d, *J* = 1.6 Hz, 1H), 5.24 (s, 2H), 3.87 (s, 6H). **^{19}F NMR (376 MHz, MeOD)** δ -94.44, -94.60.

1-(3-Chloro-5-(cyclopropylmethoxy)phenyl)-4',6'-dimethoxy-3-(pyridin-3-yl)-[5,5'-bipyrimidine]-2,4(1*H*,3*H*)-dione (S22)

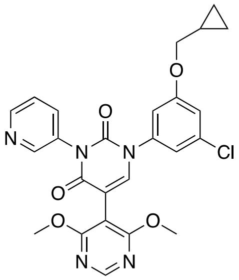

1-(3-Chloro-5-hydroxy-phenyl)-5-(4,6-dimethoxypyrimidin-5-yl)-3-(3-pyridyl)pyrimidine-2,4-dione (150 mg, 0.330 mmol) was dissolved in DMF (10 mL), and potassium carbonate (91 mg, 0.66 mmol) was added. (Bromomethyl)cyclopropane (0.10 mL, 0.72 mmol) was added, the flask was flushed with argon, and the reaction mixture was stirred at 60 °C overnight. Completion of the reaction was confirmed by LC-MS and TLC. The mixture was diluted with EtOAc and water, and the layers were separated. The organic extracts were combined, dried over anhydrous magnesium sulfate, filtered, and concentrated in vacuo. The crude material was purified by flash chromatography using DCM/MeOH (97:3) to afford 130 mg (0.26 mmol) of **S22** with 53% yield.

^{1}H NMR (400 MHz, MeOD) δ 8.63 - 8.55 (m, 2H), 8.43 (s, 1H), 7.89 (dd, *J* = 8.1, 2.4 Hz, 1H), 7.83 (s, 1H), 7.59 (dd, *J* = 8.2, 4.9 Hz, 1H), 7.18-7.13 (m, 1H), 7.05 (d, *J* = 1.8 Hz, 2H), 3.97 (s, 6H), 3.86 (d, *J* = 7.0 Hz, 2H), 1.31 - 1.15 (m, 1H), 0.67 - 0.56 (m, 2H), 0.39 - 0.31 (m, 2H).

1-(3-Chloro-5-((5-methylthiazol-4-yl)methoxy)phenyl)-6'-hydroxy-3-(pyridin-3-yl)-[5,5'-bipyrimidine]-2,4,4'(1*H*,3*H*,3'*H*)-trione (45)

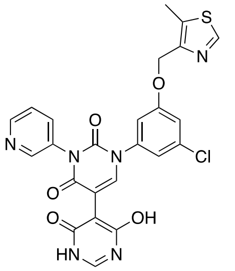

Into a 100 mL two-neck round-bottom flask under argon was placed 1-[3-Chloro-5-[(5-methylthiazol-4-yl)methoxy]phenyl]-5-(4,6-dimethoxypyrimidin-5-yl)-3-(3-pyridyl)pyrimidine-2,4-dione (0.070 g, 0.12 mmol), followed by dry MeCN (10 mL), forming a clear solution. Sodium iodide (93 mg, 0.62 mmol) was then added, followed immediately by chlorotrimethylsilane (0.080 mL, 0.62 mmol), resulting in a pale-yellow solution. The reaction mixture was heated at 60 °C for 6 h, after which LC-MS analysis confirmed complete consumption of the starting material. The mixture was cooled and diluted with EtOAc (250 mL), brine (250 mL), and 10% sodium thiosulfate solution (150 mL). After thorough mixing, the phases were separated, and the aqueous layer was extracted with EtOAc (3 × 250 mL). The combined organic extracts were washed with brine, dried over anhydrous magnesium sulfate, filtered, and concentrated in vacuo. The crude material was purified by column chromatography (initially EtOAc/hexanes, then 3% MeOH/EtOAc) to afford 1-[3-Chloro-5-[(5-methylthiazol-4-yl)methoxy]phenyl]-5-(4-hydroxy-6-oxo-1H-pyrimidin-5-yl)-3-(3-pyridyl)pyrimidine-2,4-dione (50 mg, 0.090 mmol, 75% yield) as a white powder. NMR were taken with 2 different solvents (MeOD, DMSO-*d6*) as the methyl peak was shadowed under DMSO peak.

^{1}H NMR (600 MHz, MeOD) δ 8.81 (s, 1H), 8.62 (d, *J* = 2.4 Hz, 1H), 8.59 (dd, *J* = 4.9, 1.6 Hz, 1H), 7.96 - 7.91 (m, 1H), 7.82 (s, 1H), 7.61 (dd, *J* = 8.1, 4.9 Hz, 1H), 7.55 (s, 1H), 7.31-7.27 (m, 2H), 7.20-7.15 (m, 1H), 5.24 (s, 2H), 2.55 (s, 3H). (OH and NH exchanged) **^{1}H NMR (400 MHz, DMSO-*d6*)** δ 10.34 (s, 1H), 8.88 (s, 1H),

8.56 (dd, J = 4.8, 1.5 Hz, 1H), 8.47 (d, J = 2.4 Hz, 1H), 7.80 - 7.71 (m, 1H), 7.53 (dd, J = 8.1, 4.8 Hz, 1H), 7.45 (s, 2H), 7.24 - 7.15 (m, 4H), 5.20 (s, 2H). (One of the tautomer protons exchanged. The other appeared at 10.34. The methyl group on the thiazole stands under DMSO peak can be shown in MeOD NMR.) **^{13}C NMR (151 MHz, MeOD)** δ 162.86, 159.77, 151.09, 150.95, 149.18, 148.26, 147.59, 146.85, 144.29, 141.12, 137.62, 134.80, 133.57, 133.47, 124.16, 119.69, 115.46, 112.28, 109.58, 94.13, 63.70, 9.57. **HRMS calcd for :**$C_{24}H_{18}O_5N_6{}^{35}Cl^{32}S$: 537.0742, found:537.07396

1-(3-Chloro-5-((1-(difluoromethyl)-1*H*-imidazol-2-yl)methoxy)phenyl)-6'-hydroxy-3-(pyridin-3-yl)-[5,5'-bipyrimidine]-2,4,4'(1*H*,3*H*,3'*H*)-trione (46)

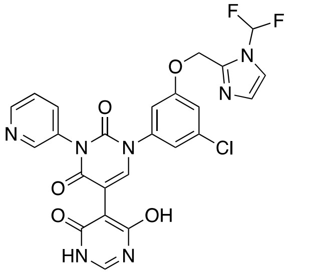

Into a 100 mL two-neck round-bottom flask under argon was placed 1-[3-Chloro-5-[[1-(difluoromethyl)imidazol-2-yl]methoxy]phenyl]-5-(4,6-dimethoxypyrimidin-5-yl)-3-(3-pyridyl)pyrimidine-2,4-dione (0.090 g, 0.16 mmol), followed by dry MeCN (10 mL), forming a clear solution. Sodium iodide (118 mg, 0.790 mmol) was then added, followed immediately by chlorotrimethylsilane (0.10 mL, 0.79 mmol), giving a pale-yellow solution. The reaction mixture was heated at 60 °C for 6 h, after which LC-MS analysis confirmed complete consumption of the starting material. The mixture was cooled and diluted with EtOAc (250 mL), brine (250 mL), and 10% sodium thiosulfate solution (150 mL). After thorough mixing, the layers were separated, and the aqueous phase was extracted with EtOAc (3 × 250 mL). The combined organic extracts were washed with brine, dried over anhydrous magnesium sulfate, filtered, and concentrated in vacuo. The crude product was purified by column chromatography (initially EtOAc/hexanes then 3% MeOH/EtOAc) to afford 1-[3-Chloro-5-[[1-(difluoromethyl)imidazol-2-yl]methoxy]phenyl]-5-(4-hydroxy-6-oxo-1H-pyrimidin-5-yl)-3-(3-pyridyl)pyrimidine-2,4-dione (50 mg, 0.090 mmol, 57% yield) as a white powder.

^{1}H NMR (600 MHz, DMSO-*d6*) δ 8.60 - 8.56 (m, 1H), 8.53 - 8.50 (m, 1H), 7.96 (d, J = 58.7 Hz, 1H), 7.84 - 7.80 (m, 2H), 7.79 (s, 1H), 7.69 (s, 1H), 7.59 - 7.48 (m, 2H), 7.31 - 7.28 (m, 1H), 7.26 - 7.23 (m, 1H), 7.14 - 7.11 (m, 1H), 5.36 (s, 2H). **^{13}C NMR (151 MHz, DMSO-*d6*)** δ 161.69, 159.01, 150.57, 150.12, 149.22, 142.99, 142.49, 141.56, 137.27, 134.03, 133.49, 129.81, 124.33, 122.16, 120.56, 118.05, 115.29, 113.20, 110.52 (t, C-F_2, J: 248 Hz; 110.52, 108.88, 107.23), 92.81, 62.78. **^{19}F NMR (565 MHz, DMSO-*d6*)** δ -91.87, -91.98. **HRMS calcd for :**$C_{24}H_{17}O_5N_7{}^{35}ClF_2$: 556.09423 found:556.09414

1-(3-Chloro-5-(cyclopropylmethoxy)phenyl)-6'-hydroxy-3-(pyridin-3-yl)-[5,5'-bipyrimidine]-2,4,4'(1*H*,3*H*,3'*H*)-trione (47)

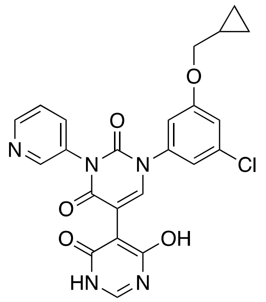

Into a 100 mL two-neck round-bottom flask under argon was placed 1-[3-Chloro-5-(cyclopropylmethoxy)phenyl]-5-(4,6-dimethoxypyrimidin-5-yl)-3-(3-pyridyl)pyrimidine-2,4-dione (0.15 g, 0.30 mmol), followed by dry MeCN (10 mL), forming a clear solution. Sodium iodide (221 mg, 1.48 mmol) was then added, followed immediately by chlorotrimethylsilane (0.190 mL, 1.48 mmol), resulting in a pale-yellow solution. The reaction mixture was heated at 60 °C for 6 h, after which LCMS analysis confirmed complete consumption of the starting material. The mixture was cooled and diluted with EtOAc (250 mL), brine (250 mL), and 10% sodium thiosulfate solution (150 mL). After thorough mixing, the layers were separated, and the aqueous phase was extracted with EtOAc (3 × 250 mL). The combined organic extracts were washed with brine, dried over anhydrous magnesium sulfate, filtered, and concentrated in vacuo. The crude material was purified by column chromatography (initially EtOAc/hexanes, then 3% MeOH/EtOAc) to afford 1-[3-Chloro-5-(cyclopropylmethoxy)phenyl]-5-(4-hydroxy-6-oxo-1H-pyrimidin-5-yl)-3-(3-pyridyl)pyrimidine-2,4-dione (95 mg, 0.20 mmol, 66% yield) as a white powder.

^{1}H NMR (600 MHz, DMSO-*d6)* δ 11.17 (s, 1H), 8.62 - 8.55 (m, 1H), 8.51 (s, 1H), 7.80 - 7.76 (m, 1H), 7.62 (s, 1H), 7.56 - 7.49 (m, 2H), 7.18 (s, 1H), 7.07 (d, *J* = 6.2 Hz, 2H), 3.87 (d, *J* = 6.9 Hz, 2H), 2.09 (s, 1H), 1.21 (dd, *J* = 13.7, 7.1 Hz, 1H), 0.56 (d, *J* = 7.8 Hz, 2H), 0.34 - 0.30 (m, 2H). **^{13}C NMR (151 MHz, DMSO-*d6)*** δ 207.04, 161.99, 160.13, 150.61, 150.13, 149.18, 147.37, 142.94, 141.63, 137.28, 134.11, 133.52, 124.32, 119.40, 117.66, 114.93, 112.73, 111.21, 92.25, 73.43, 31.17, 10.37, 3.60. **HRMS calcd for :** $C_{23}H_{19}O_5N_5{}^{35}Cl$: 480.10692 found:480.10647

1-(3-Chloro-5-((5-methylthiazol-4-yl)methoxy)phenyl)-6'-methoxy-3-(pyridin-3-yl)-[5,5'-bipyrimidine]-2,4,4'(1*H*,3*H*,3'*H*)-trione (48)

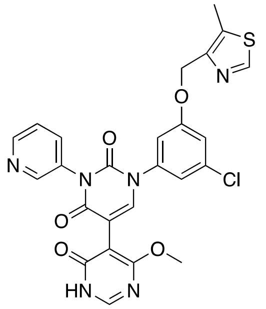

Into a 100 mL two-neck round-bottom flask under argon was placed 1-[3-Chloro-5-[(5-methylthiazol-4-yl)methoxy]phenyl]-5-(4,6-dimethoxypyrimidin-5-yl)-3-(3-pyridyl)pyrimidine-2,4-dione (0.070 g, 0.12 mmol), followed by dry MeCN (10 mL), forming a clear solution. Sodium iodide (93 mg, 0.62 mmol) was then added, followed immediately by chlorotrimethylsilane (0.080 mL, 0.62 mmol), giving a pale-yellow solution. The reaction mixture was heated at 60 °C for 6 h, after which LC-MS analysis confirmed complete consumption of the starting material. The mixture was cooled and diluted with EtOAc (250 mL), brine (250 mL), and 10% sodium thiosulfate solution (150 mL). After thorough mixing, the phases were separated, and the aqueous layer was extracted with EtOAc (3 × 250 mL). The combined organic layers were washed with brine, dried over anhydrous magnesium sulfate, filtered, and concentrated in vacuo. The crude product was purified by column chromatography (initially EtOAc/hexanes, then 3% MeOH/EtOAc) to afford 1-[3-Chloro-5-[(5-methylthiazol-4-yl)methoxy]phenyl]-5-(4-hydroxy-6-oxo-1H-pyrimidin-5-yl)-3-(3-pyridyl)pyrimidine-2,4-dione (50 mg, 0.090 mmol, 75% yield) as a white powder.

^{1}H NMR (600 MHz, MeOD) δ 8.81 (s, 1H), 8.65 - 8.54 (m, 2H), 8.06 (s, 1H), 7.96 - 7.91 (m, 1H), 7.65 (d, *J* = 0.7 Hz, 1H), 7.62 (dd, *J* = 8.1, 4.9 Hz, 1H), 7.32-7.28 (m, 1H), 7.27 (dd, *J* = 2.3, 1.8 Hz, 1H), 7.18 (dd, *J* = 2.3, 1.8 Hz, 1H), 5.24 (s, 2H), 3.86 (s, 3H), 2.56 (s, 3H). **^{13}C NMR (151 MHz, MeOD)** δ 172.95, 168.11, 162.50, 159.77, 155.45, 150.99, 150.92, 149.08, 148.40, 146.83, 143.93, 140.96, 137.56, 134.86, 133.62, 133.29, 124.22, 119.63, 115.53, 112.26, 108.43, 96.34, 63.66, 53.05, 9.56. **HRMS calcd for :**$C_{25}H_{20}O_5N_6{}^{35}Cl^{32}S$: 551.08989, found: 551.08948

1-(3-Chloro-5-(cyclopropylmethoxy)phenyl)-6'-methoxy-3-(pyridin-3-yl)-[5,5'-bipyrimidine]-2,4,4'(1*H*,3*H*,3'*H*)-trione (49)

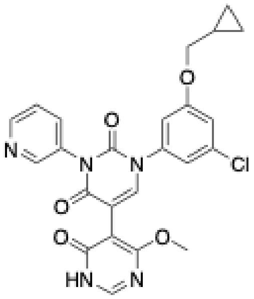

Into a 100 mL two-neck round-bottom flask under argon was placed 1-[3-Chloro-5-(cyclopropylmethoxy)phenyl]-5-(4,6-dimethoxypyrimidin-5-yl)-3-(3-pyridyl)pyrimidine-2,4-dione (0.12 g, 0.24 mmol), followed by dry MeCN (10 mL), forming a clear solution. Sodium iodide (106 mg, 0.710 mmol) was then added, followed immediately by chlorotrimethylsilane (0.090 mL, 0.71 mmol), giving a pale-yellow solution. The reaction mixture was heated at 60 °C for 6 h, after which LCMS analysis confirmed complete consumption of the starting material. The mixture was cooled and diluted with EtOAc (250 mL), brine (250 mL), and 10% sodium thiosulfate solution (150 mL). After thorough mixing, the phases were separated, and the aqueous layer was extracted with EtOAc (3 × 250 mL). The combined organic extracts were washed with brine, dried over anhydrous magnesium sulfate, filtered, and concentrated in vacuo. The crude product was purified by column chromatography (initially EtOAc/hexanes, then 3%

MeOH/EtOAc) to afford 1-(3-Chloro-5-(cyclopropylmethoxy)phenyl)-6′-methoxy-3-(pyridin-3-yl)-[5,5′-bipyrimidine]-2,4,4′(1H,3H,3′H)-trione (40 mg, 0.080 mmol, 34% yield) as a white powder.

^{1}H NMR (400 MHz, DMSO-*d6)* δ 12.47 (s, 1H), 8.61 (d, J = 4.8 Hz, 1H), 8.57 - 8.52 (m, 1H), 8.20 (s, 1H), 7.90 (s, 1H), 7.83 (d, J = 8.2 Hz, 1H), 7.56 (dd, J = 8.2, 4.8 Hz, 1H), 7.25 (s, 1H), 7.12 (s, 2H), 3.89 (d, J = 7.1 Hz, 2H), 3.84 (s, 3H), 1.23 (d, J = 10.6 Hz, 1H), 0.63 - 0.53 (m, 2H), 0.33 (d, J = 5.0 Hz, 2H). **^{13}C NMR (151 MHz, DMSO-*d6)*** δ 167.04, 162.39, 160.93, 160.16, 150.39, 150.05, 149.99, 149.49, 144.52, 141.22, 137.22, 134.14, 133.03, 124.43, 119.53, 115.27, 112.89, 105.76, 98.18, 73.47, 54.97, 10.35, 3.60. **HRMS calcd for :** $C_{24}H_{21}ClN_5O_5$: 494.12257 found: 494.12258.

Molecular Modelling:

Docking studies were carried out using Glide-SP (Schrödinger modelling suite, versions 2024-2 to 2025-4). Advanced settings changes included using enhanced sampling (4x) and increasing the settings under Selection of initial poses (50000, 1000, 4000 resp. and “use expanded sampling”). The maximum number of minimization steps was increased to 1000. When docking was used for the purposes of preparing structures for FEP analysis, the same settings were employed but with the additional setting of using core constraints such that all ligands were restricted to placement of a common core and only the S4 pocket moiety conformationally sampled. Relative free energy binding values were obtained using FEP+ from Schrödinger. For the purposes of docking and FEP analysis, the SARS-CoV-2 Mpro receptor PDB ID 7M8N was utilized. Only chain A was used for FEP even though the active form is the homodimer (retrospective analysis of both the monomers and homodimers did not afford superior results when using the homodimer and so the monomer was used going forward due to significantly reduced calculation times). In all cases, receptors were prepared using the Protein Preparation workflow, including adding missing side chains (and optimization thereof) as necessary.

Rotational barrier calculations for compounds **3, 4** and **48** were carried out on simplified scaffolds (please see below) using Jaguar – Relaxed Coordinate Scan. All settings were left at default with the exception that a PBF solvation model was employed. For each of the three simplified scaffolds, the dihedral was defined using four atoms and furthermore all four options (with 10° steps) were calculated for each scaffold and the average of all four torsion scans, per scaffold, was used to estimate the rotational barrier, for each of the three scaffold types.

Simplified scaffold for Compound **3**

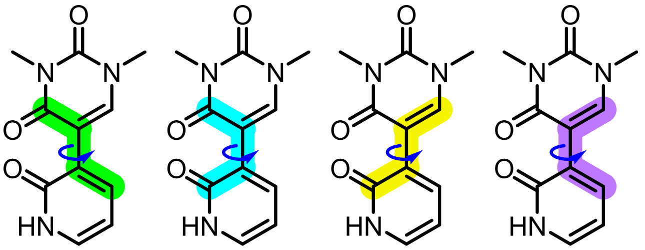

Simplified scaffold for Compound **4**

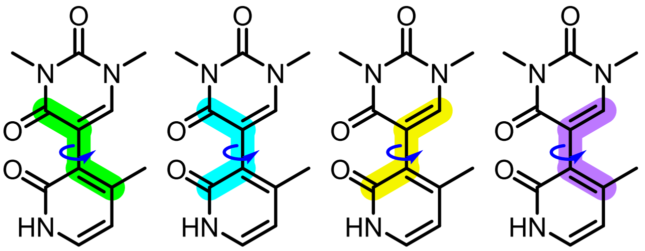

Simplified scaffold for Compound **48**

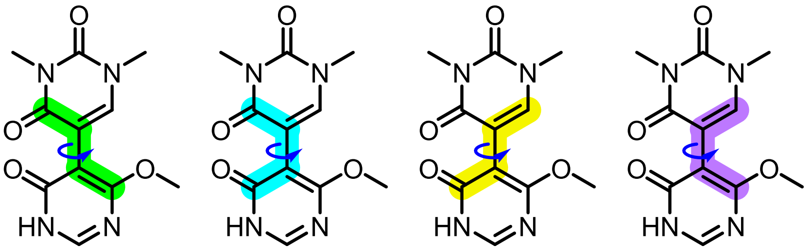

SARS Cov-2 Main Protease Enzymatic (IC_{50}) Assay Protocol

Reaction Buffer:

50 mM Tris-HCl pH 7.3, 1 mM EDTA, 0.005% Triton X-100, 1% DMSO, +/- 1 mM DTT.

Enzymes:

SARS-CoV-2 Mpro (3CLpro): Reaction Biology Corp. product, MSC-11-519. Recombinant protease (GenBank accession: QHD43415, aa3264-3569) expressed in *E. coli* with no tag, MW = 33.8 kDa. 25 μM in reaction.

Substrate:

Covidyte ED450: AAT Bioquest Cat# 13538; [NH2-C(EDANS)VNSTQSGLRK(DABCYL)M-COOH] FRET peptide substrate. MW=2,034 Da. 5 μM in reaction.

Control compound:

GC376

Measurement: EnVision (PE). Ex/Em 340/492 nm

Reaction Procedure: Prepare indicated enzyme and substrate in freshly prepared reaction buffer. Deliver enzyme solution into the reaction well. Then deliver compounds in DMSO into the reaction mixture by using Acoustic Technology (Echo 550, LabCyte Inc. Sunnyvale, CA) in nanoliter range. After 20 min pre-

incubation, deliver substrate solution into the reaction well to initiate the reaction. The enzyme activities were monitored every 5 min as a time-course measurement of the increase in fluorescence signal from fluorescently-labelled peptide substrate for 120 min. at room temperature.

Data Analysis: Take slope (signal/time) of linear portion of measurement. Slope is calculated using Excel, and curve fits are performed using Prism software. The IC_{50} curve fits were performed using GraphPad Prism 4 software with 4 parameters fit using the following formula:

*Y = Bottom + (Top-Bottom)/(1+10^((LogIC50-X)*HillSlope))*

Prism setting: 4 parameters sigmoidal dose-response (variable slope), constraint; Bottom = 0, Top less than 120. Curve fits were performed when the % enzyme activities at the highest concentration of compounds were less than 65%.

Kinetic solubility

The test compounds were first dissolved in DMSO to provide stock solutions of 20 or 30 mM. Kinetic solubility experiments were then performed in a 96-well microtiter plate (Clear-Bottom, Black, Corning® Costar) with a final volume of 250 µL per well in rows B-H. Each plate was prepared as follows: Add 240 µL of DMSO to well A1 (well A1 served as a DMSO blank), 48 µL to well A3, 60 µL to well A4, 80 µL to well A5, and 120 µL to all remaining wells in row A (A6-A12). Next, add 70 µL of the test compound (DMSO stock solution) directly to well A2 without further dilution. Add 192 µL of the test compound (DMSO stock solution) to well A3. Further dilutions were then conducted by removing 180 µL from well A3 and adding it to A4, followed by removing 160 µL from well A4 and adding it to A5, followed by removing 120 µL from well A5 and adding it to A6, and sequentially removing 120 µL from the remaining wells up to A12. Next, 30 µL of Dulbecco's phosphate-buffered saline (DPBS, Gibco®, 1X, no Ca2+ and Mg2+, pH 7.1-7.3) was added to all wells in rows B-H. Then, transfer 2.5 µL of the sample solutions prepared in row A to the corresponding wells in rows B-H and the 96-well plate was incubated at 25℃ for 1 min. Next, DPBS (217.5 µL) was added to all wells in rows B-H, and the 96-well plate was once more incubated at 25℃ for 2 h. This procedure provided seven replicate experiments with final concentrations of the test compound (1% DMSO in DPBS) ranging from either 200 to 0.625 µM or 300 to 0.9375 µM in rows B-H, depending on whether a 20 mM or 30 mM DMSO stock solution was used, respectively (i.e. a 100-fold dilution from sample preparation in row A). After incubation, the plates were analyzed using a NEPHELOstar® microplate reader, and the segmental linear regression data was processed with MARS analysis software (BMG LabTech).

Table S1. Aqueous Solubility of the tested compounds

Compound No	Structure	Aqueous Solubility µM

1	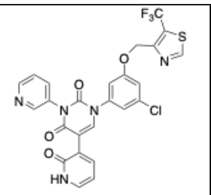	44.7
2	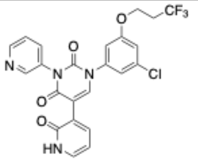	51.8
4	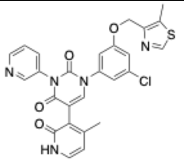	>300
19	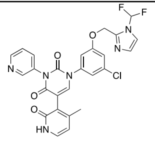	144.1
20	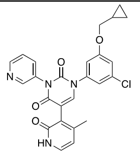	>300
37	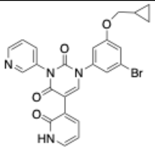	110.2
38	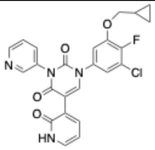	23
42	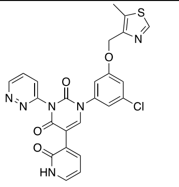	143.5
43	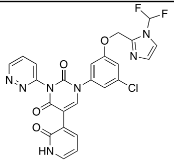	300
45	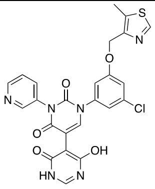	>300

46	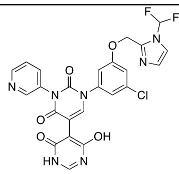	>300
47	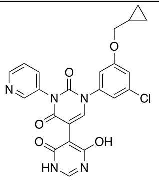	>300
48	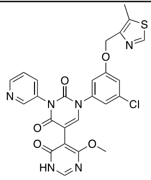	>100
49	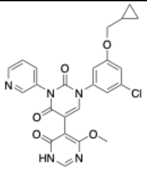	>300

Compound 19: 144.1 μM

Test Name: ZS-MPRO-34_30MMPBS Date: 3/6/2026 Time: 11:57:31 AM
ID1: 30 mM PBS
Nephelometry **Standard Curve(s) / Fit Results**

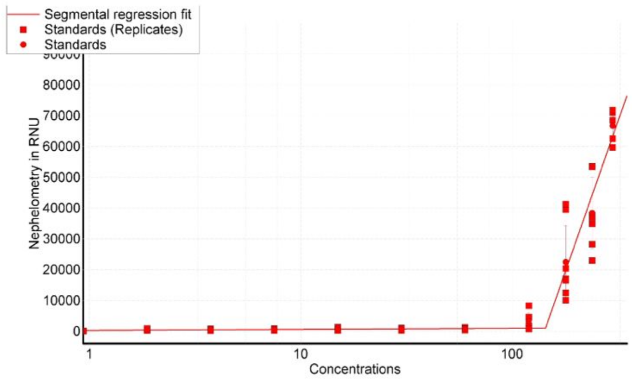

Segmental regression fit

Formula: Y = Slope1 * log(x) + Offset1; Y = Slope2 * log(x) + Offset2

Parameter	Value
Slope1	330.76273
Offset1	283
r1	0.90878
r1²	0.82588
Slope2	195865.69921
Offset2	-421837
r2	0.97275
r2²	0.94624
intSectX	144.143
intSectY	997
Curve Color:	

Compound 20: >300 μM

Test Name: ZS-MPRO-35_20MM PBS Date: 3/5/2026 Time: 12:23:32 PM
ID1: 20 mM PBS
Nephelometry **Standard Curve(s) / Fit Results**

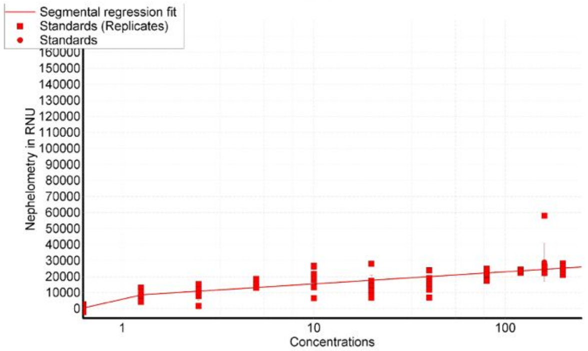

Segmental regression fit

Formula: Y = Slope1 * log(x) + Offset1; Y = Slope2 * log(x) + Offset2

Parameter	Value
Slope1	26905.24476
Offset1	5880
r1	1.00000
r1²	1.00000
Slope2	7556.36742
Offset2	7861
r2	0.86611
r2²	0.75014
intSectX	1.266
intSectY	8634
Curve Color:	

Compound 37: 110.2 µM

Test Name: MER-MPRO-008_30MMPBS Date: 3/6/2026 Time: 12:16:37 PM
ID1: 30 mM PBS
Nephelometry **Standard Curve(s) / Fit Results**

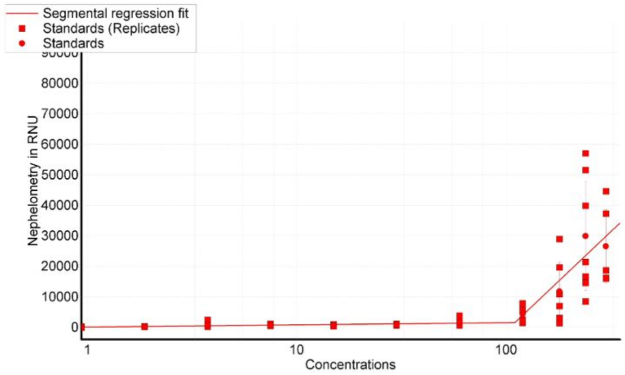

Segmental regression fit

Formula: Y = Slope1 * log(x) + Offset1; Y = Slope2 * log(x) + Offset2

Parameter	Value
Slope1	709.99434
Offset1	43
r1	0.90002
r1²	0.81003
Slope2	65044.81235
Offset2	-131340
r2	0.92324
r2²	0.85238
intSectX	110.199
intSectY	1493
Curve Color:	

Compound 38: 23 µM

ID1: 20 mM PBS
Nephelometry **Standard Curve(s) / Fit Results**

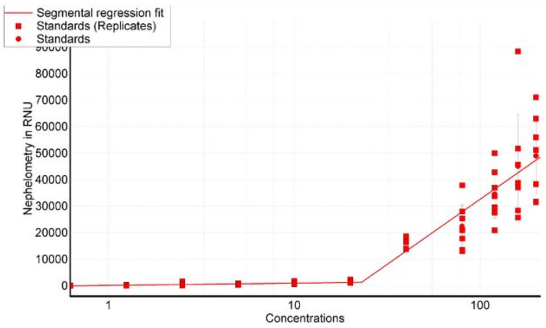

Segmental regression fit

Formula: Y = Slope1 * log(x) + Offset1; Y = Slope2 * log(x) + Offset2

Parameter	Value
Slope1	842.30538
Offset1	130
r1	0.95647
r1²	0.91484
Slope2	49107.98121
Offset2	-65590
r2	0.96524
r2²	0.93168
intSectX	22.995
intSectY	1277
Curve Color:	

Compound 42: 143.5 µM

Test Name: ZS-MPRO-42_30MMPBS Date: 3/5/2026 Time: 11:29:38 AM
ID1: 30 mM PBS
Nephelometry **Standard Curve(s) / Fit Results**

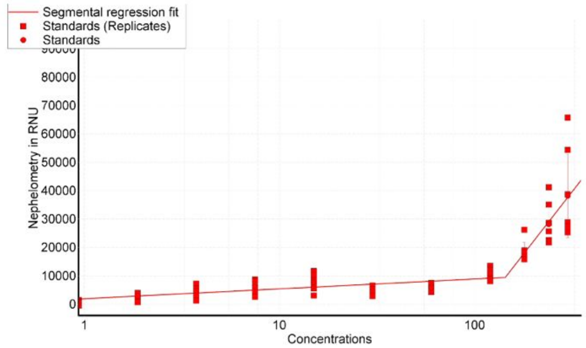

Segmental regression fit

Formula: Y = Slope1 * log(x) + Offset1; Y = Slope2 * log(x) + Offset2

Parameter	Value
Slope1	3471.62389
Offset1	1924
r1	0.82950
r1²	0.68807
Slope2	88174.52857
Offset2	-180763
r2	0.99689
r2²	0.99378
intSectX	143.481
intSectY	9411
Curve Color:	

Compound 43: >300 µM

Test Name: ZS-MPRO-43_30MMPBS Date: 3/5/2026 Time: 11:55:32 AM
ID1: 30 mM PBS
Nephelometry **Standard Curve(s) / Fit Results**

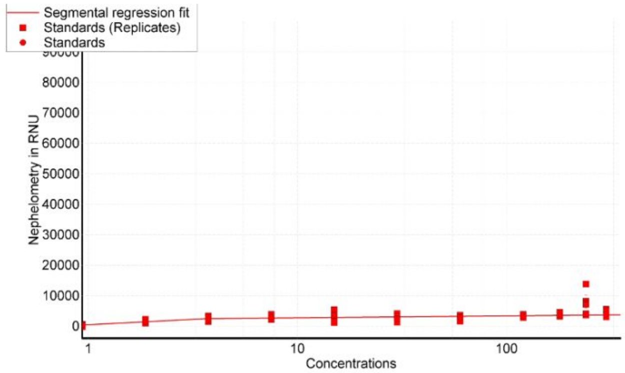

Segmental regression fit

Formula: Y = Slope1 * log(x) + Offset1; Y = Slope2 * log(x) + Offset2

Parameter	Value
Slope1	3538.32798
Offset1	448
r1	1.00000
r1²	1.00000
Slope2	646.35740
Offset2	2068
r2	0.84635
r2²	0.71631
intSectX	3.630
intSectY	2429
Curve Color:	

Compound 45: >300 µM

ID1: 30mM PBS
Nephelometry **Standard Curve(s) / Fit Results**

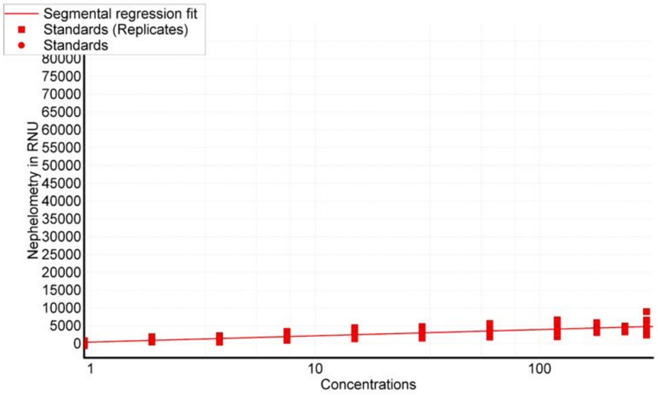

Segmental regression fit

Formula: Y = Slope * log(x) + Offset

Parameter	Value
Slope	1755.21554
Offset	416
r	0.98473
r²	0.96970
Curve Color:	

Compound 46: >300 µM

Test Name: ZS-MPRO-68_30MMPBS Date: 3/6/2026 Time: 12:35:23 PM
ID1: 30 mM PBS
Nephelometry **Standard Curve(s) / Fit Results**

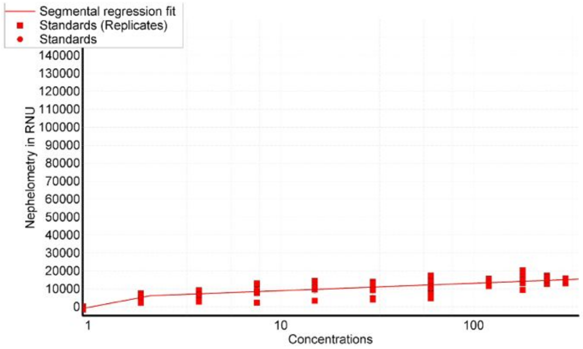

Segmental regression fit

Formula: Y = Slope1 * log(x) + Offset1; Y = Slope2 * log(x) + Offset2

Parameter	Value
Slope1	20240.50788
Offset1	-371
r1	1.00000
r1²	1.00000
Slope2	4143.48450
Offset2	4808
r2	0.92691
r2²	0.85917
intSectX	2.097
intSectY	6141
Curve Color:	

Compound 47: 142.1 μM

Test Name: ZS-MPRO-70_30MMPBS Date: 3/5/2026 Time: 12:50:42 PM
ID1: 30 mM PBS
Nephelometry **Standard Curve(s) / Fit Results**

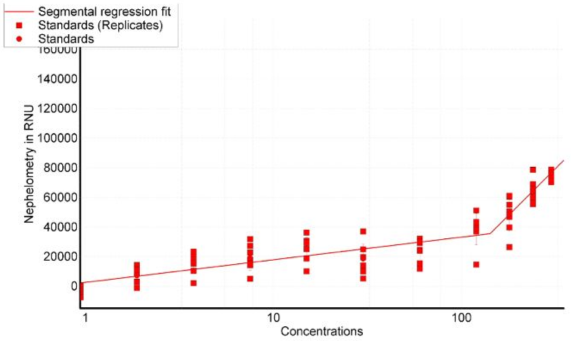

Segmental regression fit

Formula: Y = Slope1 * log(x) + Offset1; Y = Slope2 * log(x) + Offset2

Parameter	Value
Slope1	15274.35532
Offset1	2551
r1	0.91593
r1²	0.83892
Slope2	126521.33927
Offset2	-236921
r2	0.99984
r2²	0.99969
intSectX	142.107
intSectY	35431
Curve Color:	

Compound 48: >100 μM

Test Name: ZS-MPRO-56-2_10MMPBS Date: 3/6/2026 Time: 12:54:29 PM
ID1: 10 mM PBS
Nephelometry **Standard Curve(s) / Fit Results**

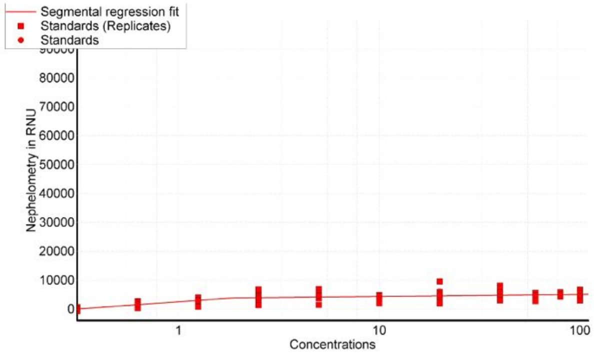

Segmental regression fit

Formula: Y = Slope1 * log(x) + Offset1; Y = Slope2 * log(x) + Offset2

Parameter	Value
Slope1	4951.65020
Offset1	2507
r1	0.99700
r1²	0.99401
Slope2	703.36242
Offset2	3630
r2	0.60068
r2²	0.36081
intSectX	1.838
intSectY	3816
Curve Color:	

Compound 49: >300 µM

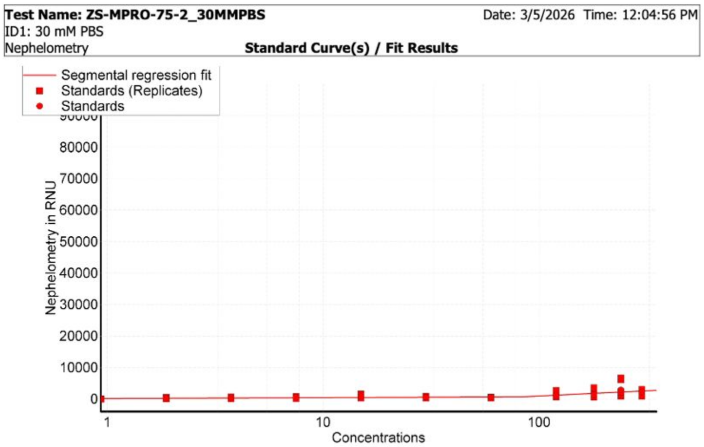

Segmental regression fit

Formula: Y = Slope1 * log(x) + Offset1; Y = Slope2 * log(x) + Offset2

Parameter	Value
Slope1	273.67490
Offset1	171
r1	0.76692
r1²	0.58817
Slope2	3309.77590
Offset2	-5663
r2	0.75710
r2²	0.57320
intSectX	83.475
intSectY	697
Curve Color:	

Liver Microsome Stability Assay Protocol

Human liver microsomes (HLMs, 20 mg/mL), CD-1 mouse liver microsomes (MLMs, 20 mg/mL), Sprague Dawley rat liver microsomes (RLMs, 20 mg/mL) were purchased from Xenotech. NADPH was purchased from Sigma-Aldrich and prepared in 10 mM stock solutions of distilled H_2O (Invitrogen™ UltraPure™). Verapamil and diphenhydramine were both purchased from Sigma-Aldrich and served as positive controls for LM stability assays. Test compounds and positive controls were initially dissolved in DMSO to make 10 mM stock solutions. Sample solutions were then further diluted in ~~100%~~ 70% MeCN to 500 µM. Next, the reactions were prepared by mixing human or mouse liver microsomes (55 µL) with potassium phosphate buffer (100 mM, 928.4 µL) in 1.5 mL Eppendorf tubes. The test compounds (6.6 µL of 500 µM solution) were subsequently added to the suspensions, and the reaction mixtures were incubated at 37 °C for 5 min. Afterward, the liver microsome reactions were initiated with 110 µL of 10 mM NADPH and further incubated at 37 °C for the designated time course of the study. This procedure provided experiments with a final volume of 1100 µL (0.6% organic solvent content) and a final test compound concentration of 3 µM. Aliquots (100 µL) were removed from each reaction mixture in duplicate at 0, 5, 10, 15, 30 min, and 60 min time intervals and quenched with 200 µL of cold internal standard solution (ISTD, 3 µM 7-ethoxy-d_5-coumarin in MeCN). Quenched aliquots were then centrifuged at 12,500 g for 10 min, and the resulting supernatants were withdrawn and placed in LC-MS vials to be analyzed by LC-MS/MS (Agilent G6460C QQQ MS coupled with an Infinity II 1260 HPLC). Each test compound was run in tandem with positive and negative control experiments for quality assurance. Positive control reactions were

conducted at a final volume of 550 μL for a single run of each time point. Lastly, the negative control experiment was conducted with test compounds and liver microsomes in the absence of NADPH (150 μL) and analyzed at the 60min time point.

Data Analysis. Each data point was analyzed in triplicate using in-between blank washes to avoid carry over and to equilibrate the column for the subsequent runs. Averages of these triplicates for individual compounds at each time point were then normalized to the data at 0 min, representing 100% test compound remaining or 0% metabolism. Half-lives ($t_{1/2}$) were calculated by plotting ln of % test compound remaining versus time and performing linear regression to determine slope. Slope = −k and $t_{1/2}$ = 0.693/k for first-order kinetics.

Liver Microsome Stability Assay Setup Example:Test Compound: 928.4 μL Potassium Phosphate Buffer (100 mM) + 55 μL HLM or MLM + 110 μL NADPH (10 mM) + 6.6 μL TC (500 μM). Positive Control: 464.2 μL Potassium Phosphate Buffer (100 mM) + 28μL HLM or MLM + 55 μL NADPH (10 mM) + 3.3 μL Verapamil or Diphenhydramine (500 μM). Negative Control: ~~141.6~~ 519.2 μL Potassium Phosphate Buffer (100 mM) + 28 μL HLM or MLM + 3.3 μL TC (500 μM). Quenching Mixture: 200 μL MeCN with ISTD (3 μM d5-7-ethoxy coumarin). Final Volume After Quenching: 300 μL (100 μL from reaction mixture + 200 μL quencher solution; ISTD final concentration of 1.0 μM).

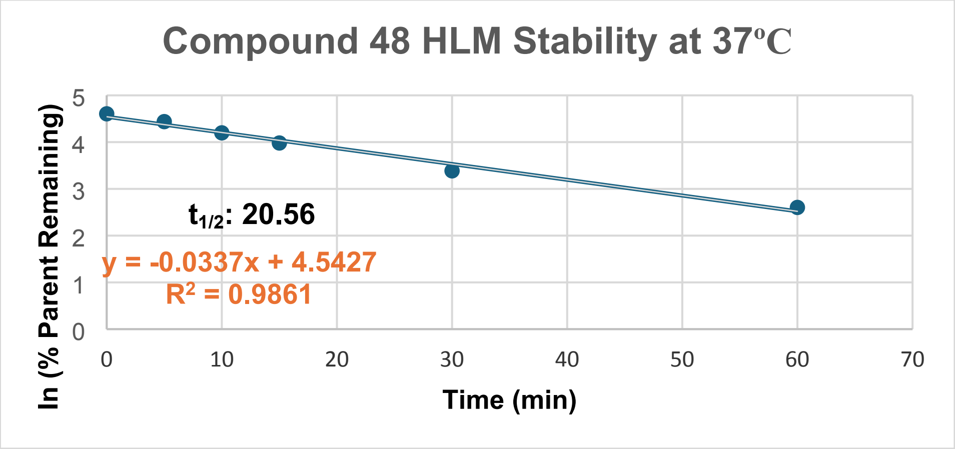

Time	Response ratio		Average RR	Rel %	ln
	A	B			
0	0,038760807	0,038490393	0,03862527	100	4,605170186
5	0,032723589	0,032648092	0,032685738	84,62267931	4,438202308
10	0,025684594	0,025732429	0,025708525	66,55882226	4,198086102
15	0,02069601	0,020684057	0,020690024	53,56602982	3,980915095
30	0,011356587	0,01145937	0,011408068	29,53524599	3,38558433
60	0,00517913	0,005252871	0,005215902	13,50385977	2,602975553

Appendix:

^{1}H NMR spectrum of **7**

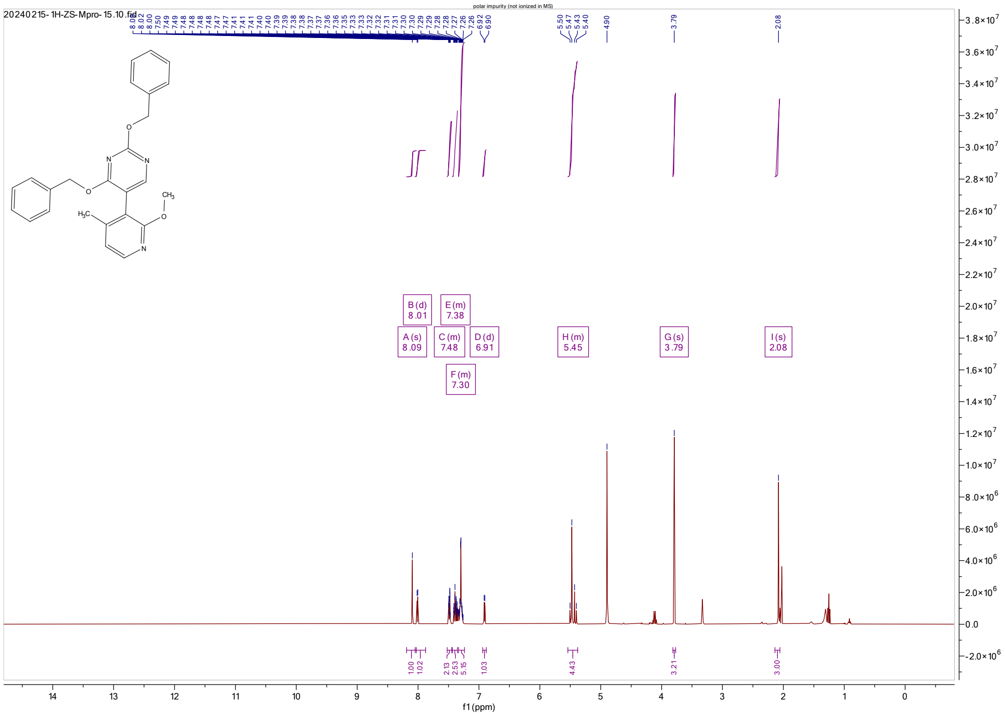

^{1}H NMR spectrum of **8**

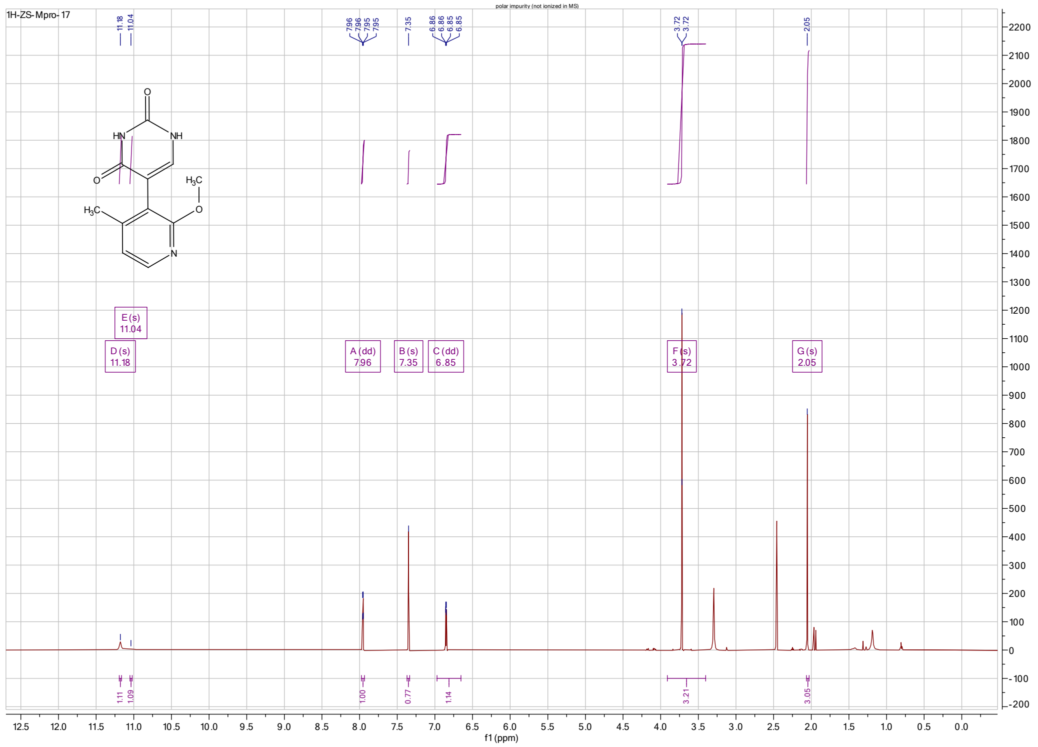

^{1}H NMR spectrum of **10**

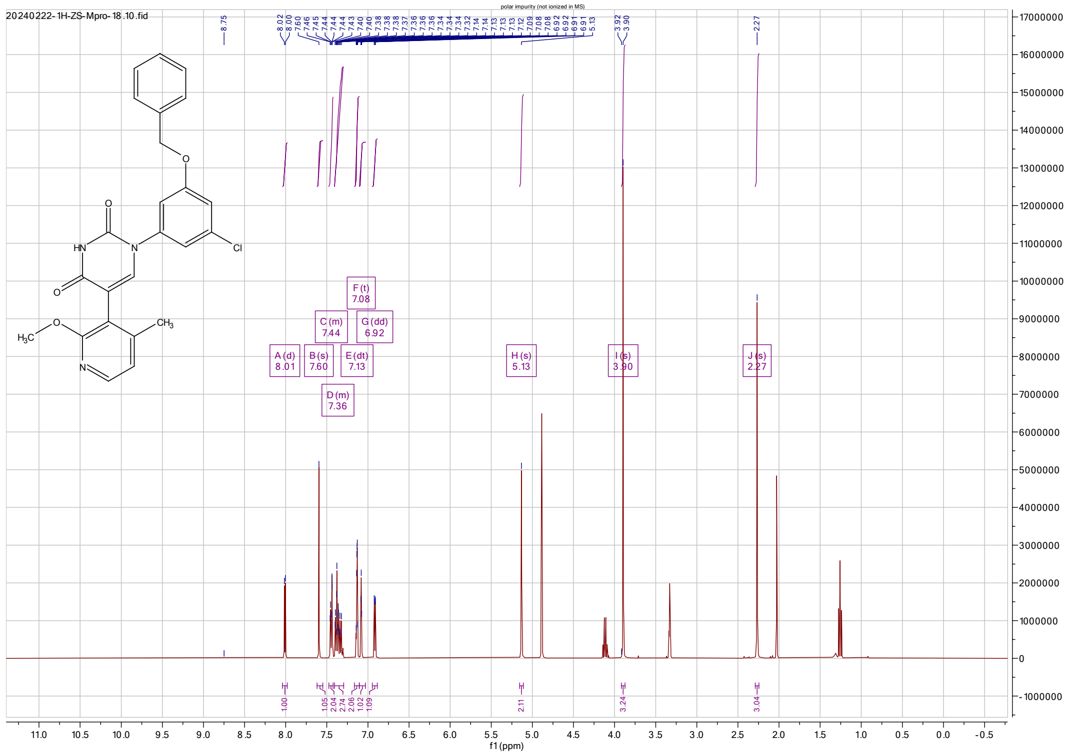

^{1}H NMR spectrum of **12**

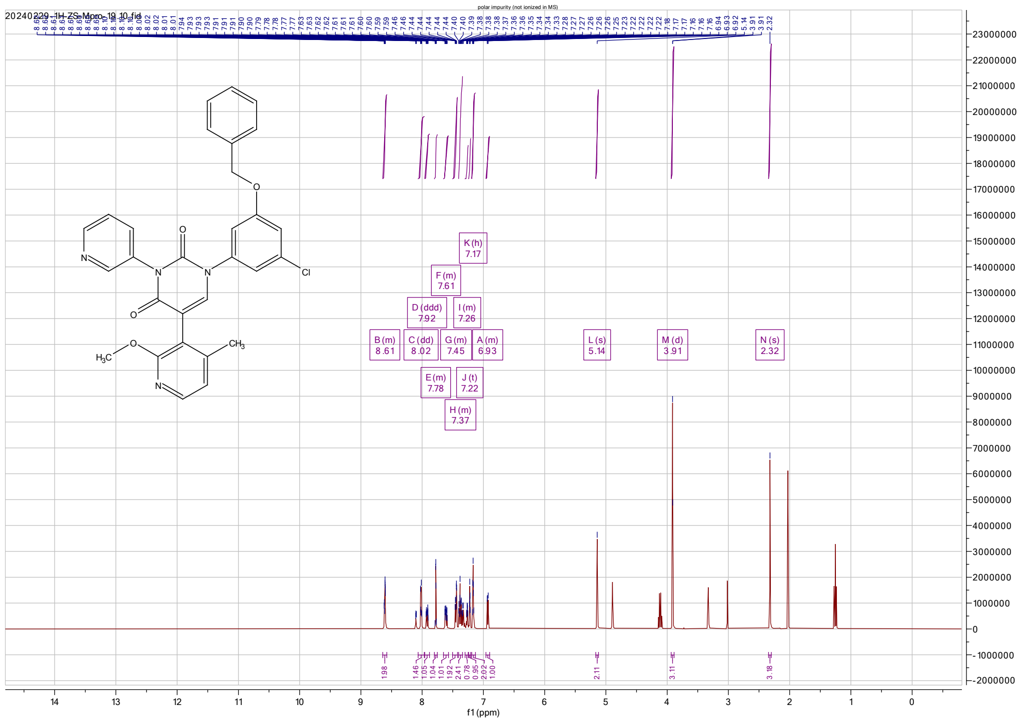

^{1}H NMR spectrum of **13**

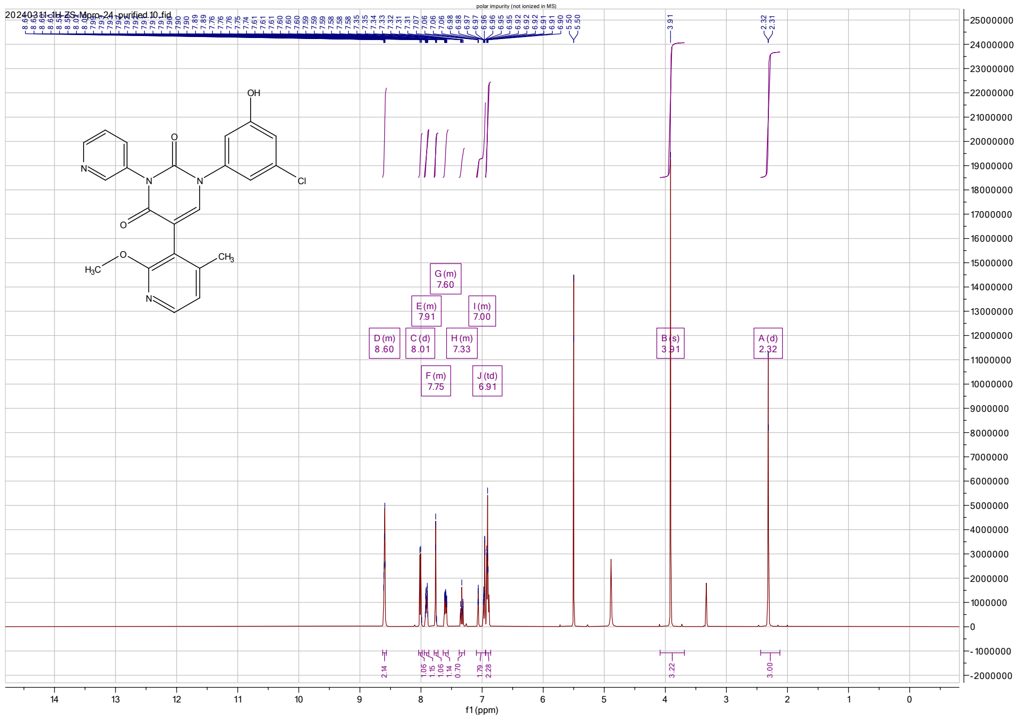

^{1}H NMR spectrum of **14**

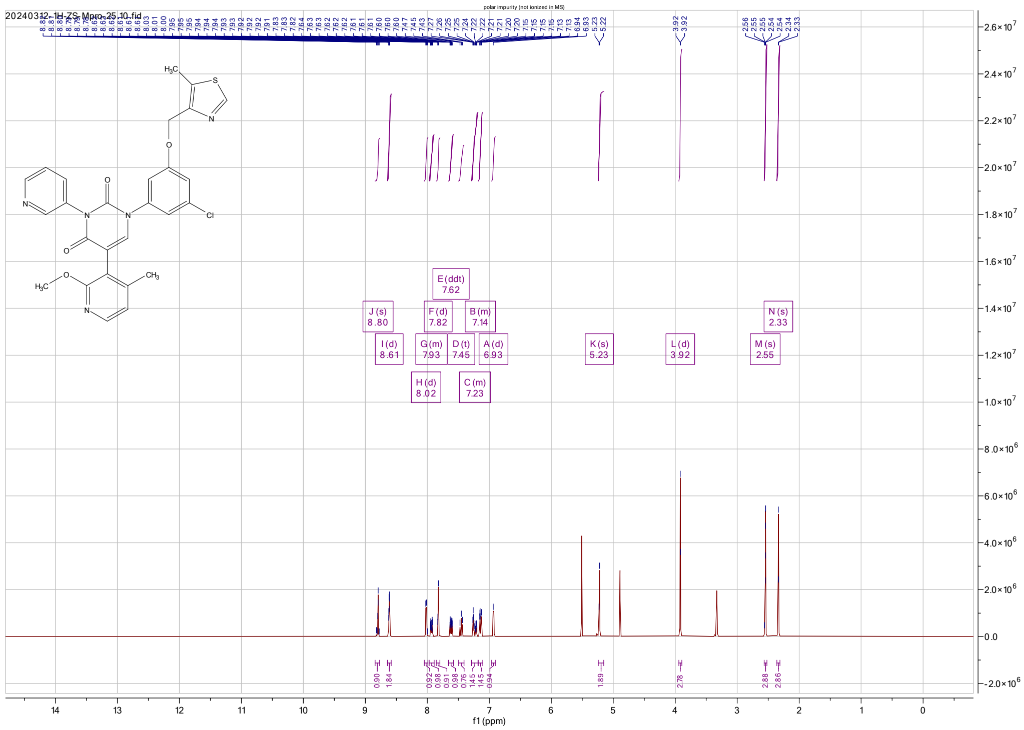

^{1}H NMR spectrum of **15**

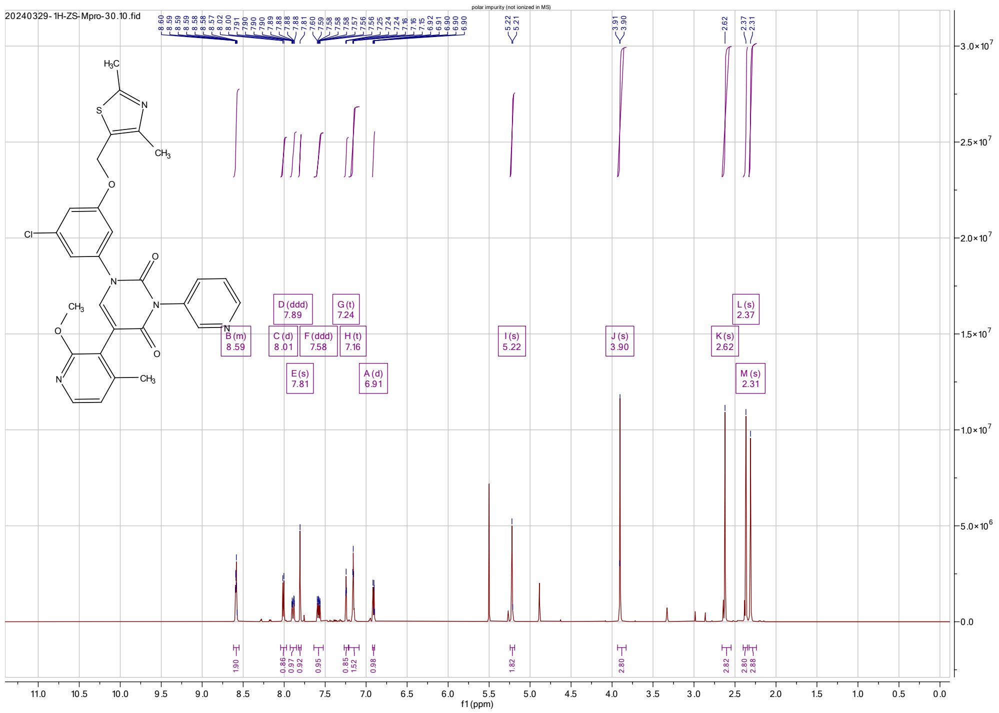

^{1}H and ^{19}F NMR spectrum of **16**

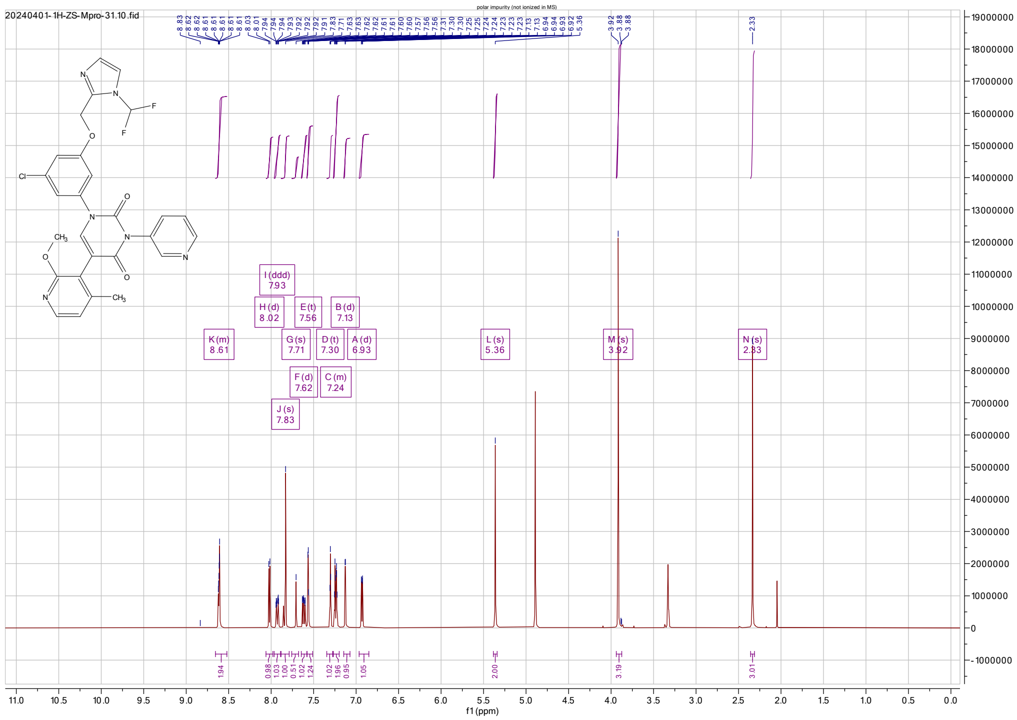

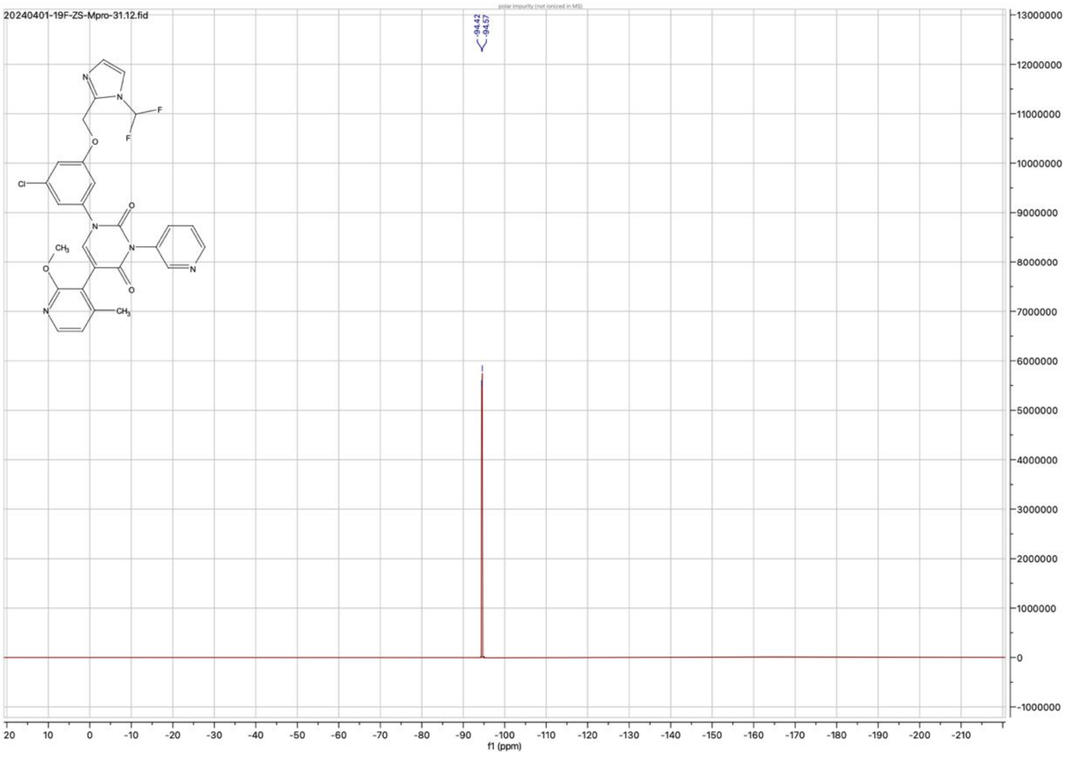

^{1}H NMR spectrum of **17**

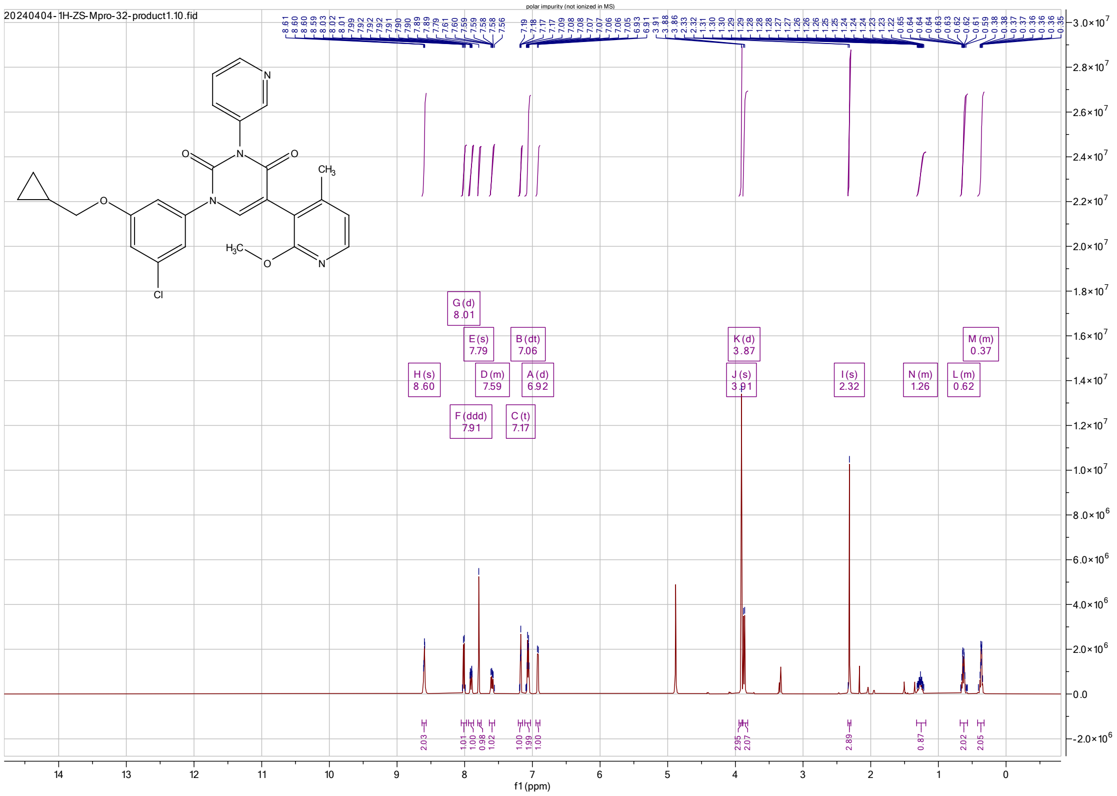

^{1}H and ^{13}C NMR spectrum of **4**

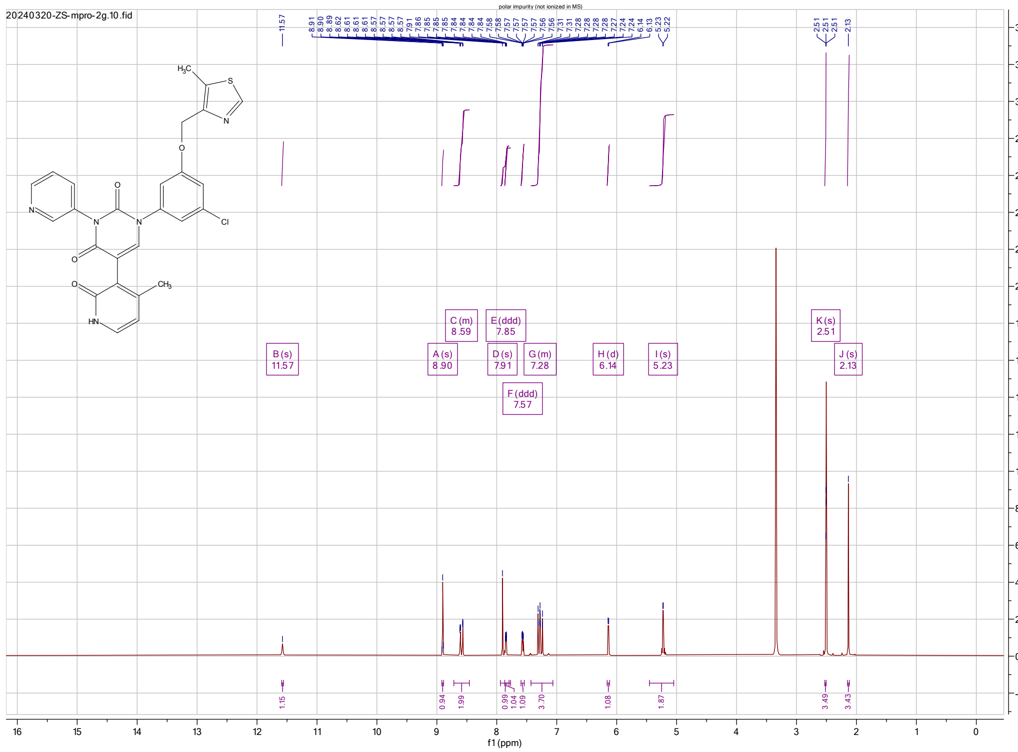

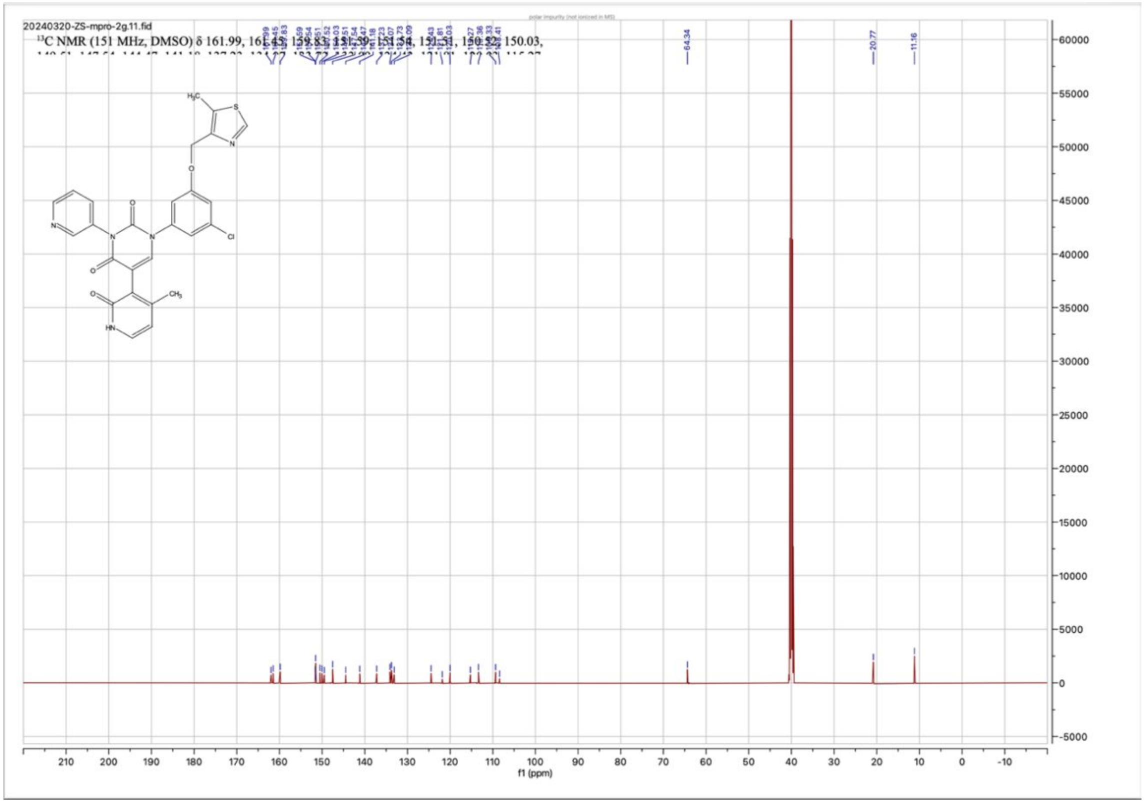

^{1}H and ^{13}C NMR spectrum of **18**

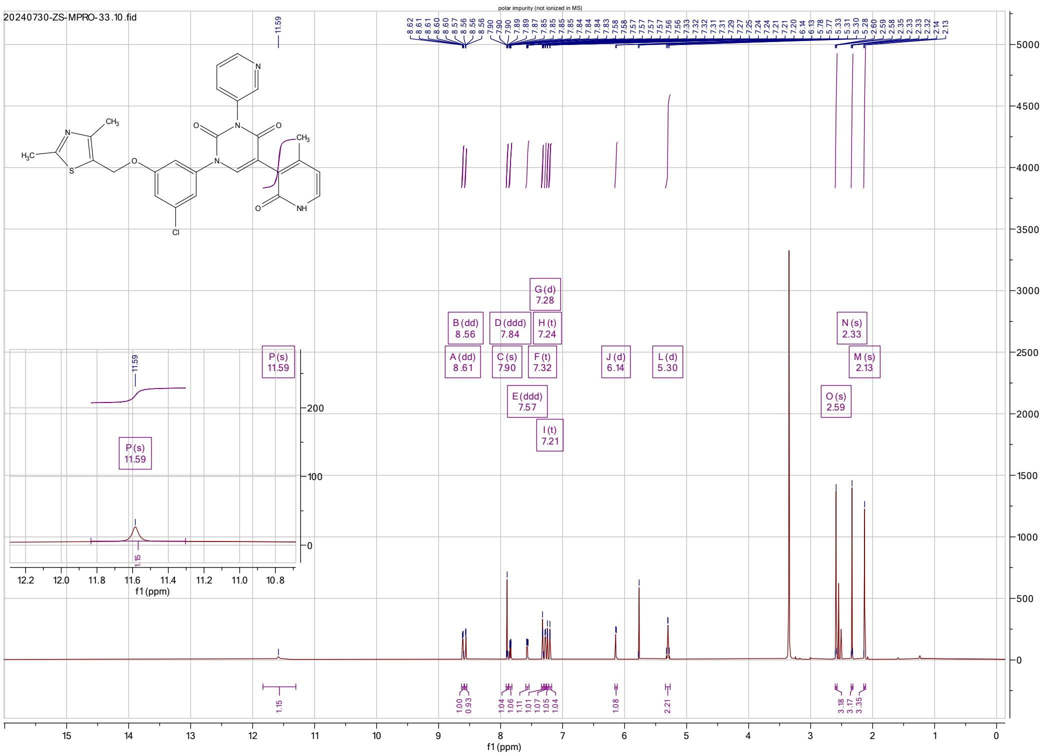

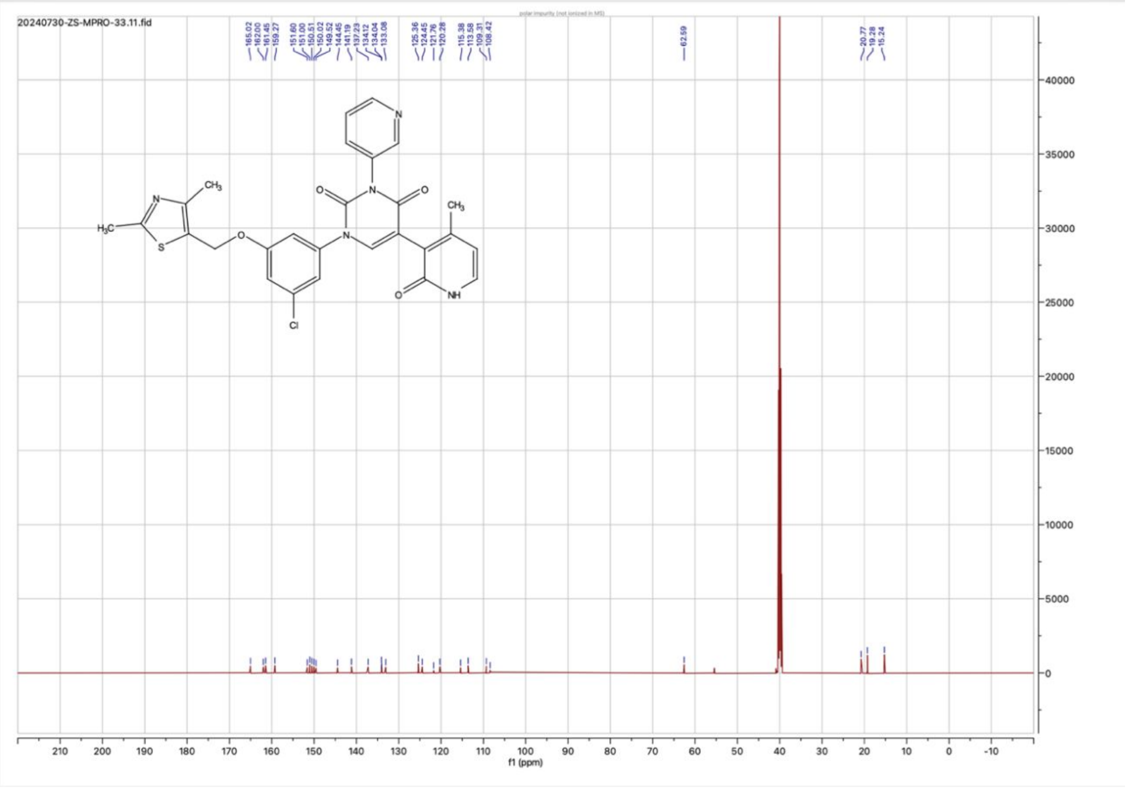

^{1}H, ^{19}F and ^{13}C NMR spectrum of **19**

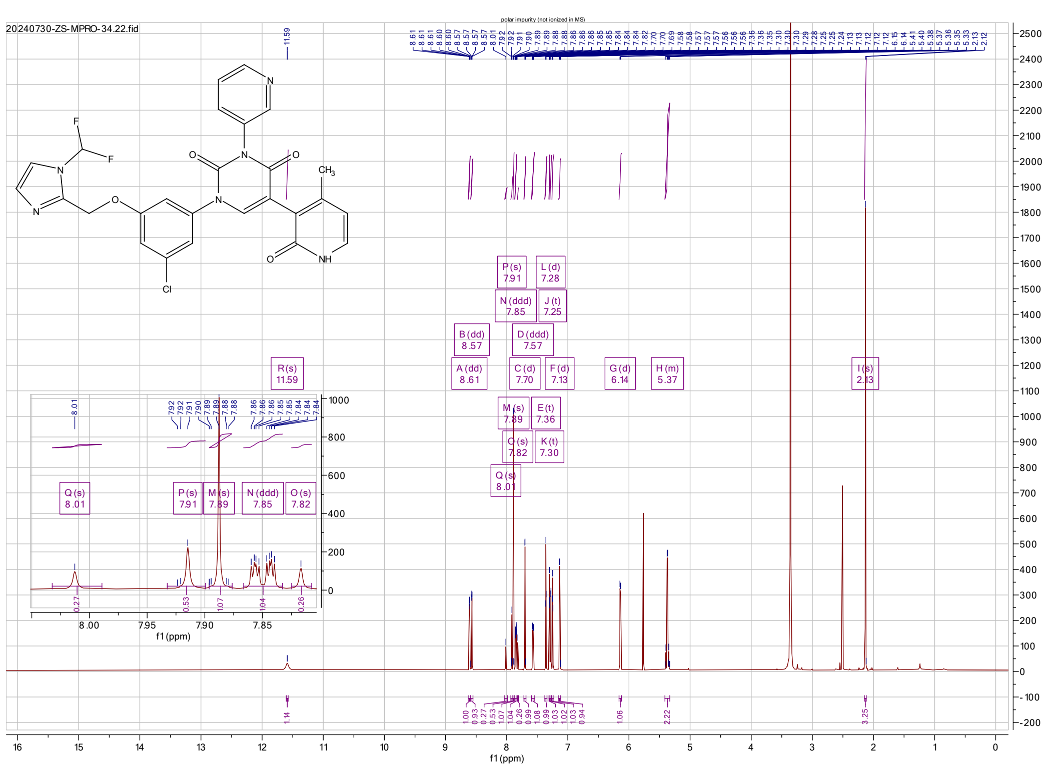

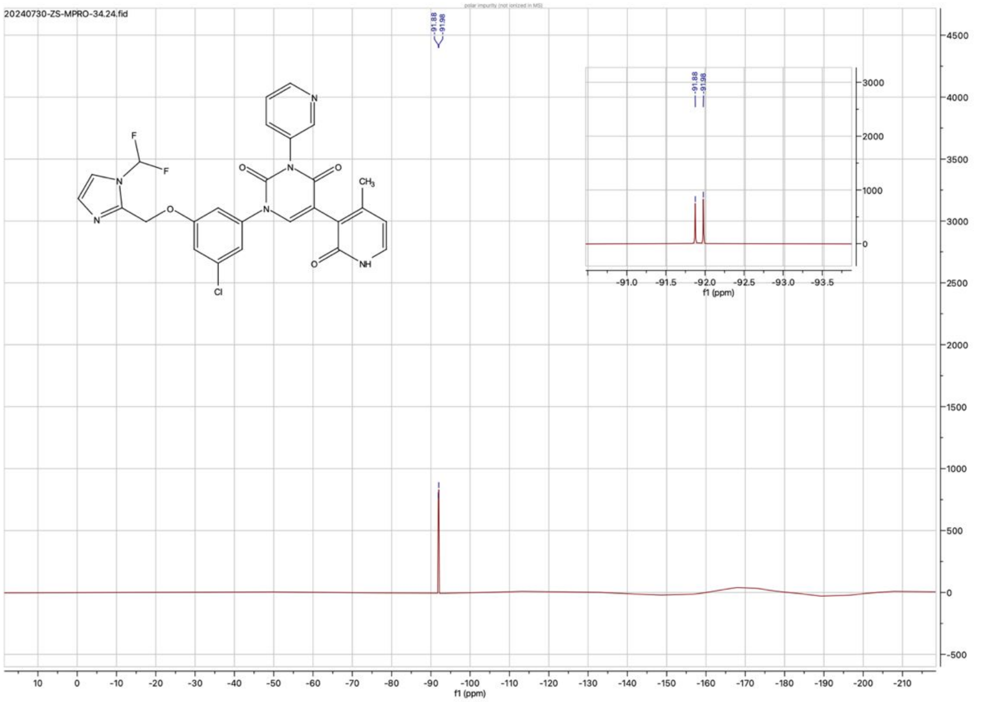

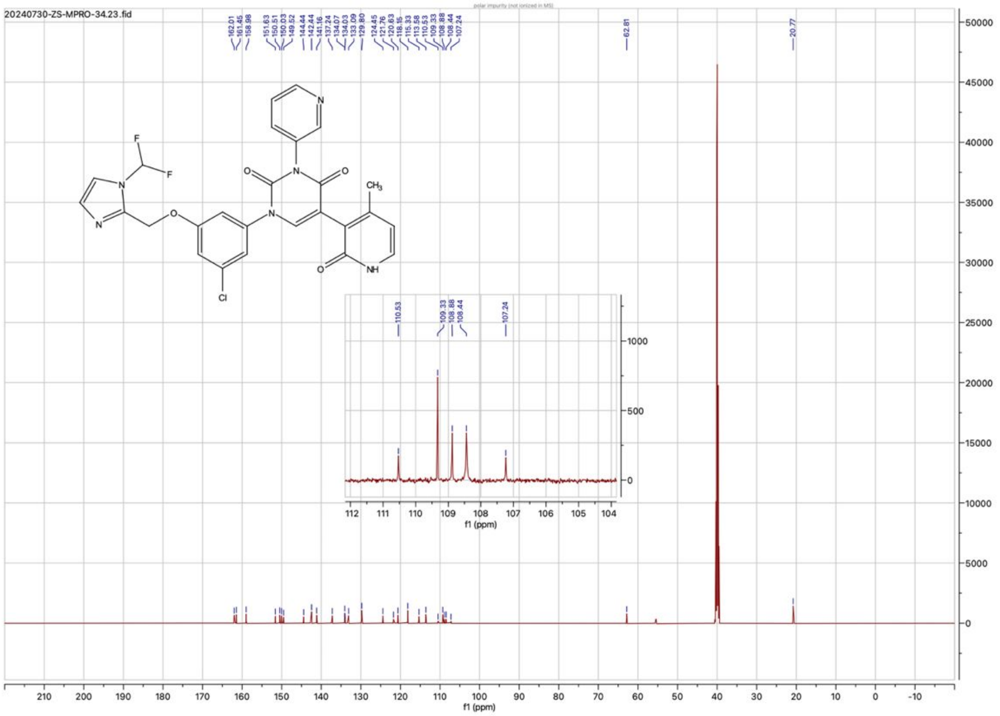

20240730-ZS-MPRO-34.23.fid
polar impurity (not ionized in MS)
162.01
161.45
158.98
151.63
150.51
150.03
149.52
144.44
142.44
141.16
137.24
134.07
134.03
133.09
129.80
124.45
121.76
120.63
118.15
115.33
113.58
110.53
109.33
108.88
108.44
107.24
62.81
20.77
110.53
109.33
108.88
108.44
107.24
f1 (ppm)
f1 (ppm)

^{1}H and ^{13}C NMR spectrum of **20**

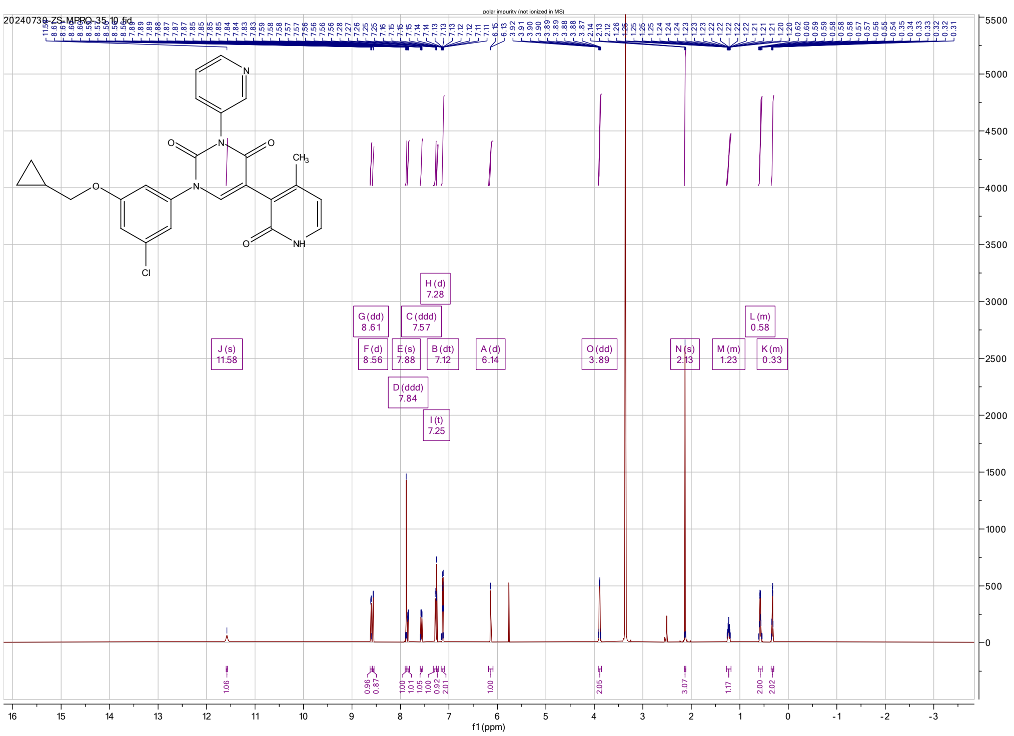

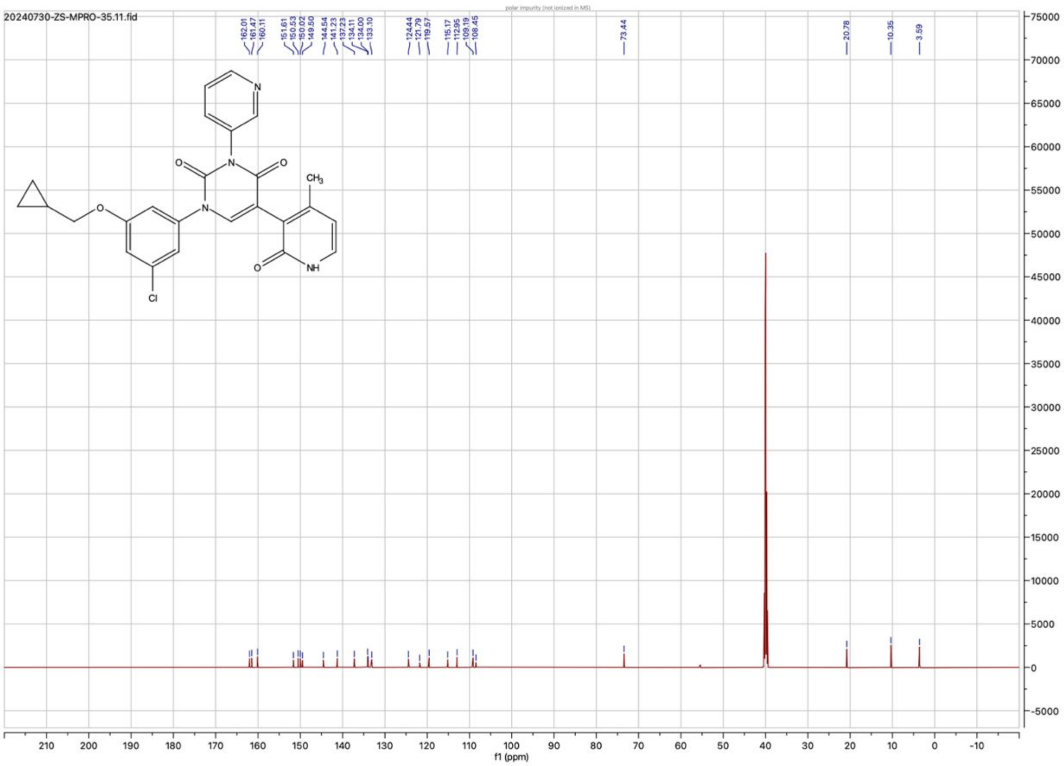

^{1}H NMR spectrum of **S2**

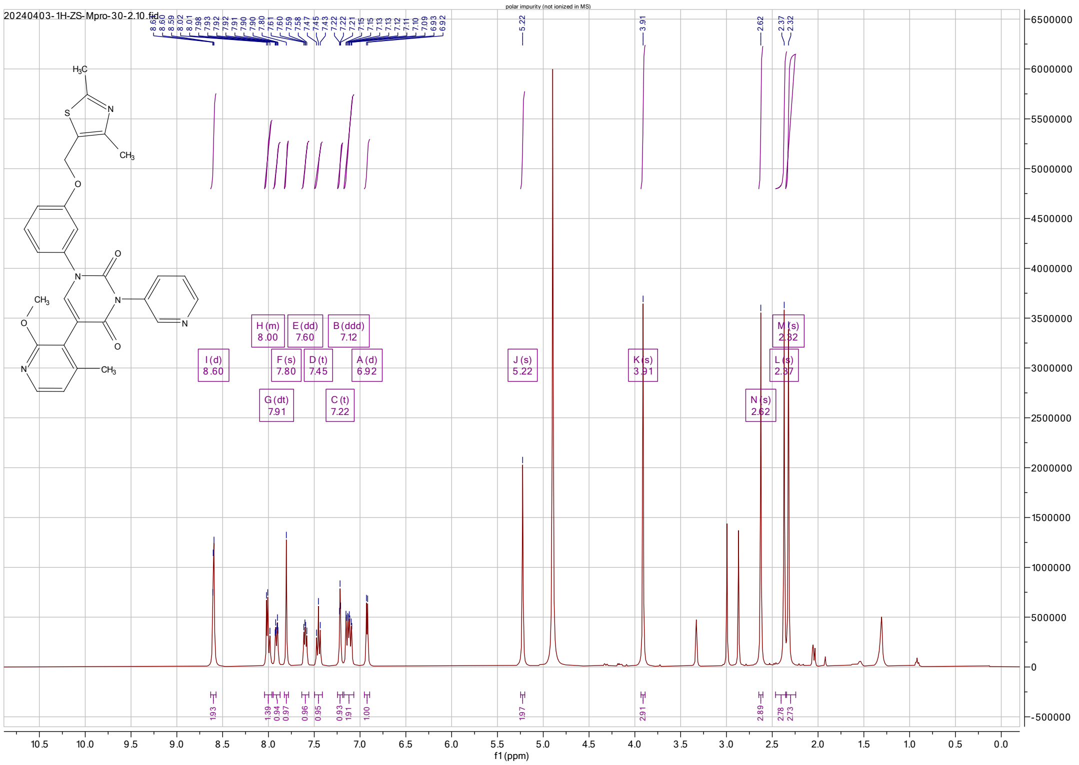

^{1}H and ^{19}F NMR spectrum of **S3**

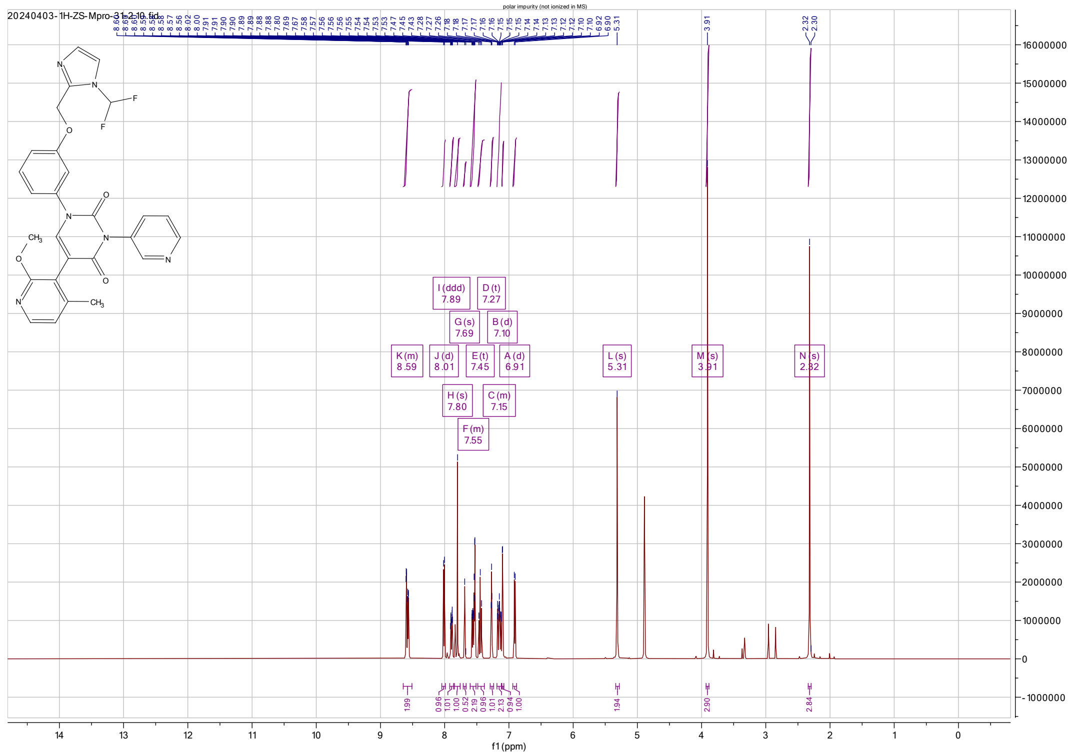

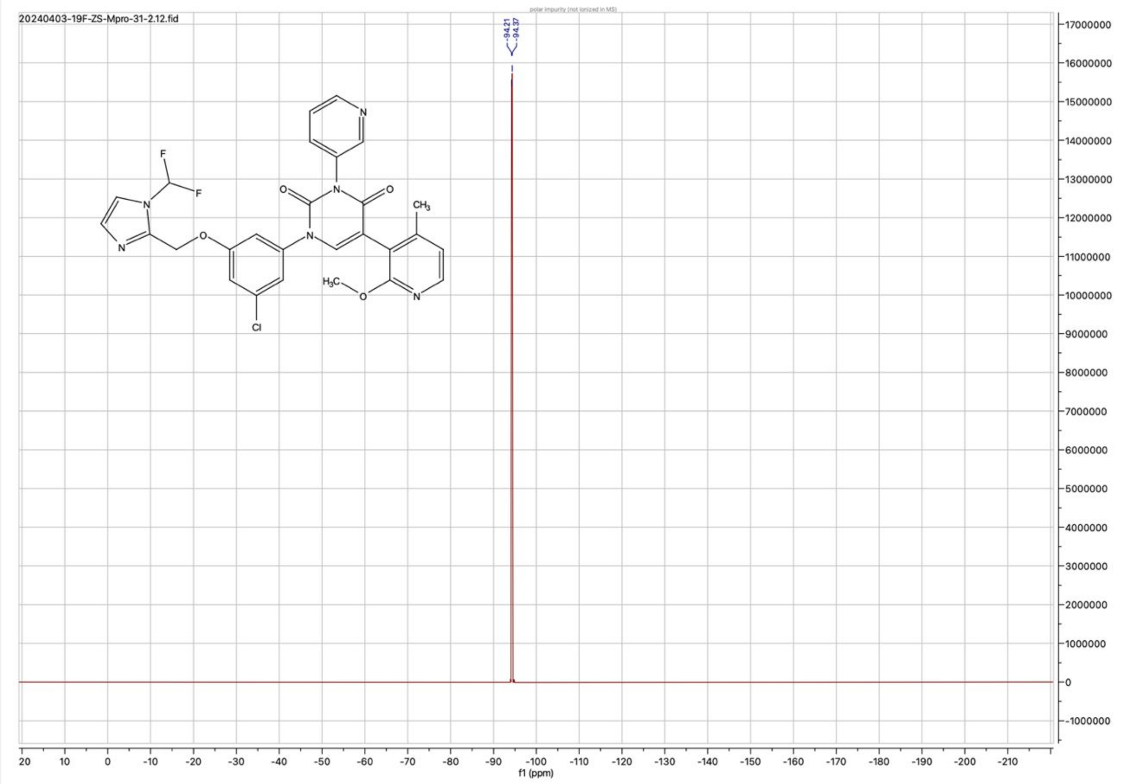

^{1}H and ^{13}C NMR spectrum of **S4**

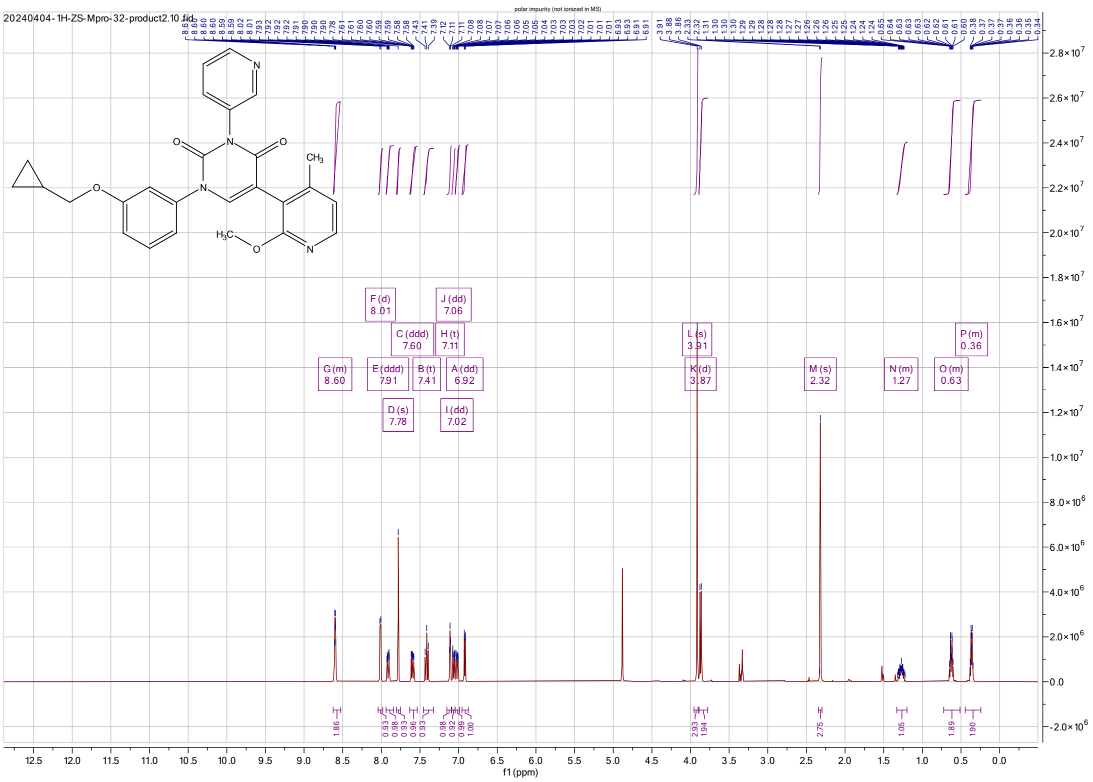

^{1}H and ^{13}C NMR spectrum of **21**

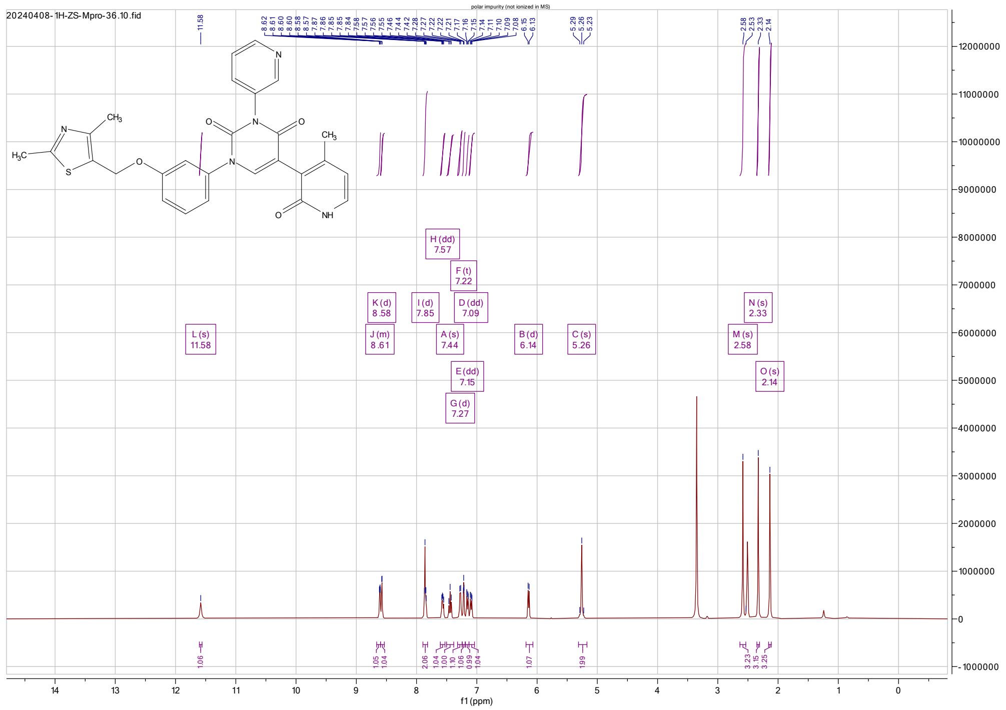

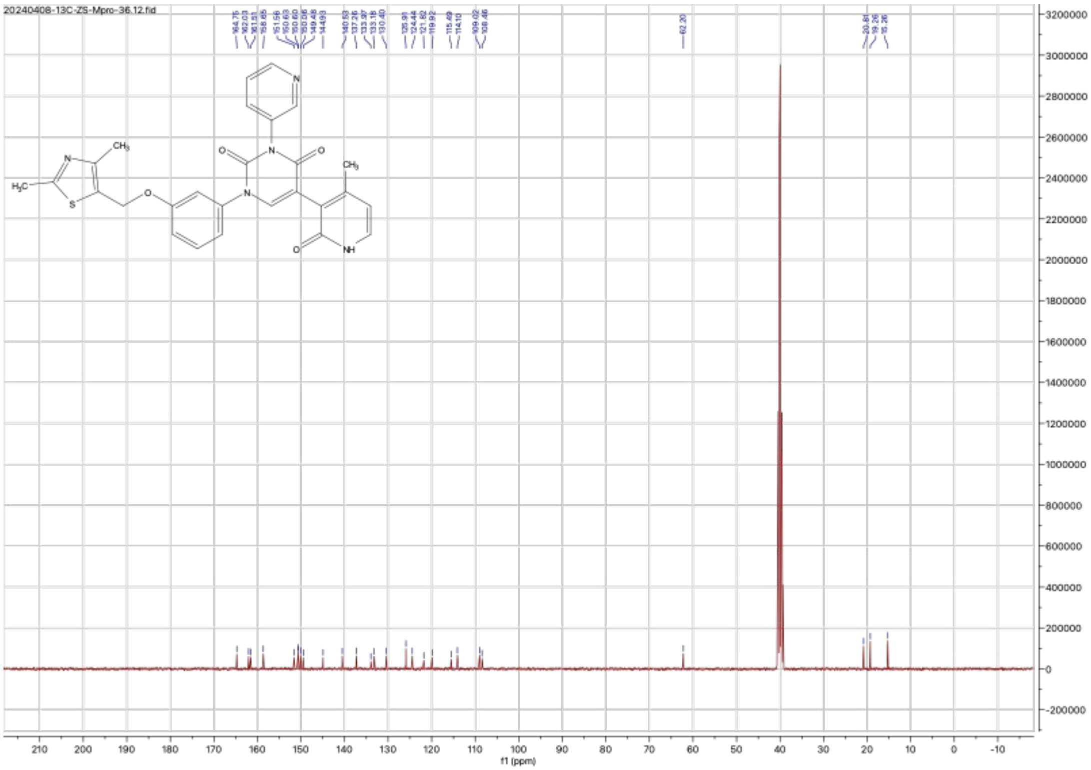

^{1}H, ^{19}F and ^{13}C NMR spectrum of **22**

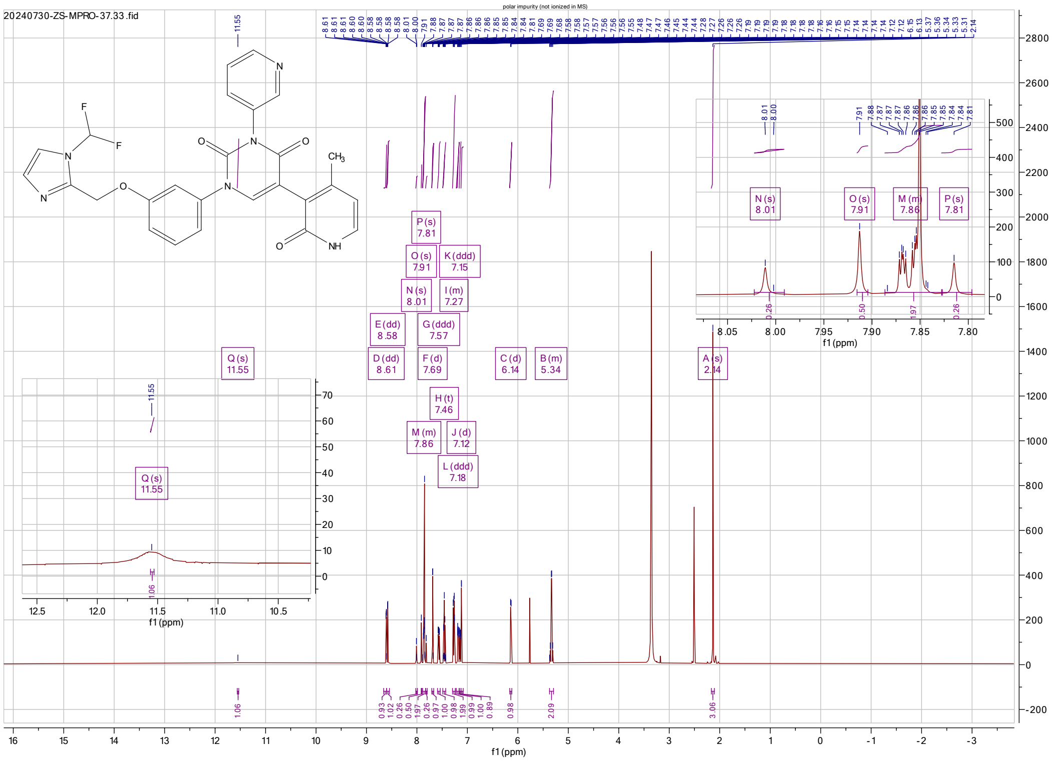

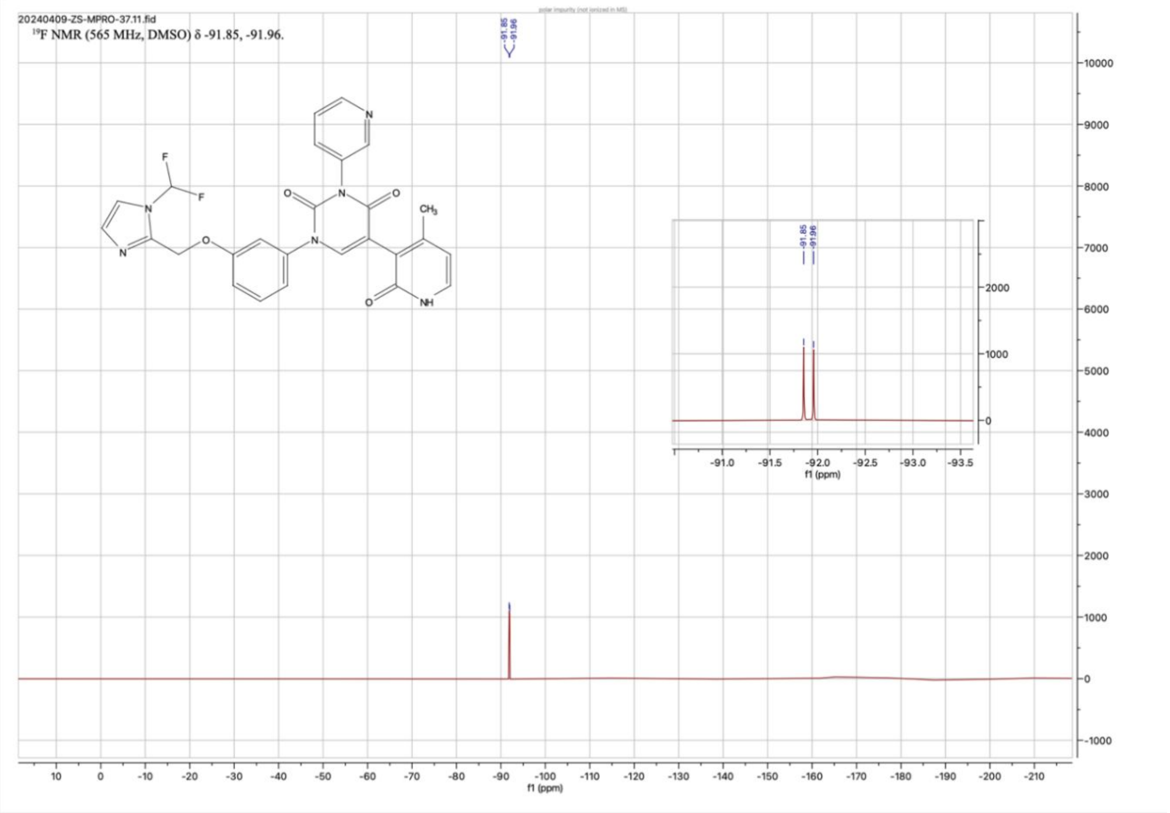

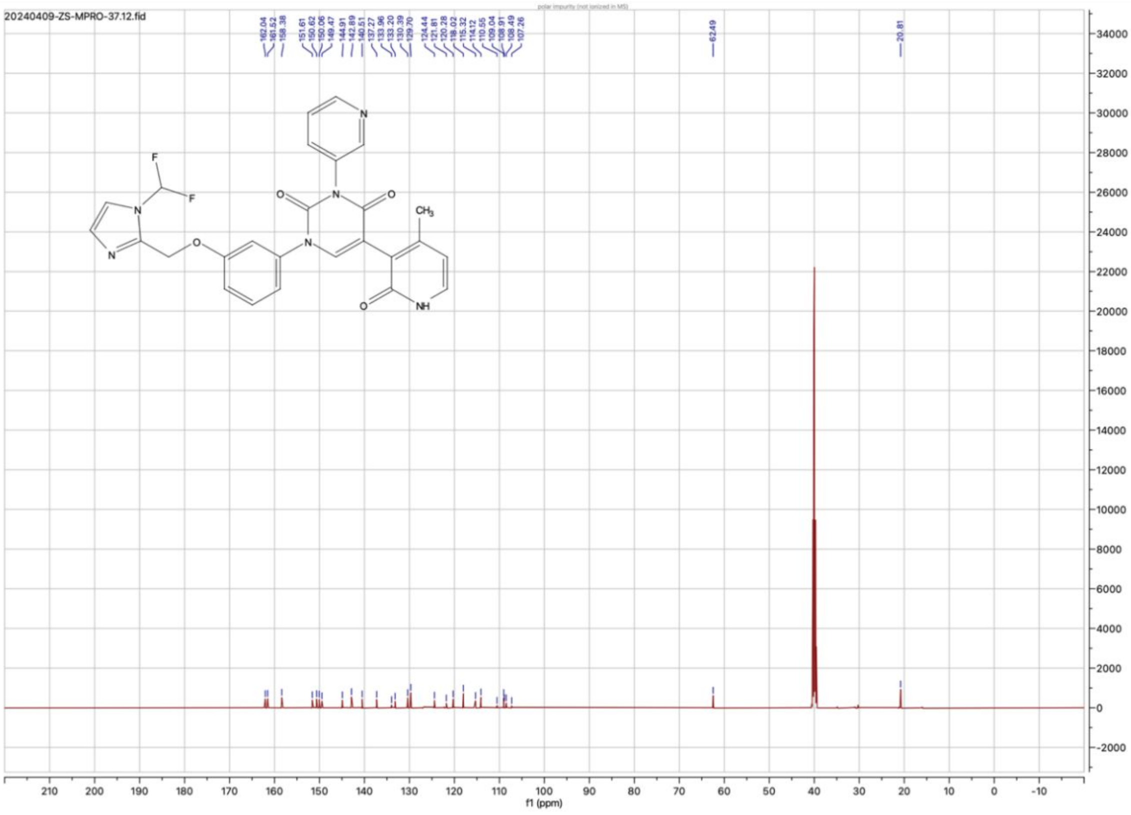

^{1}H and ^{13}C NMR spectrum of **23**

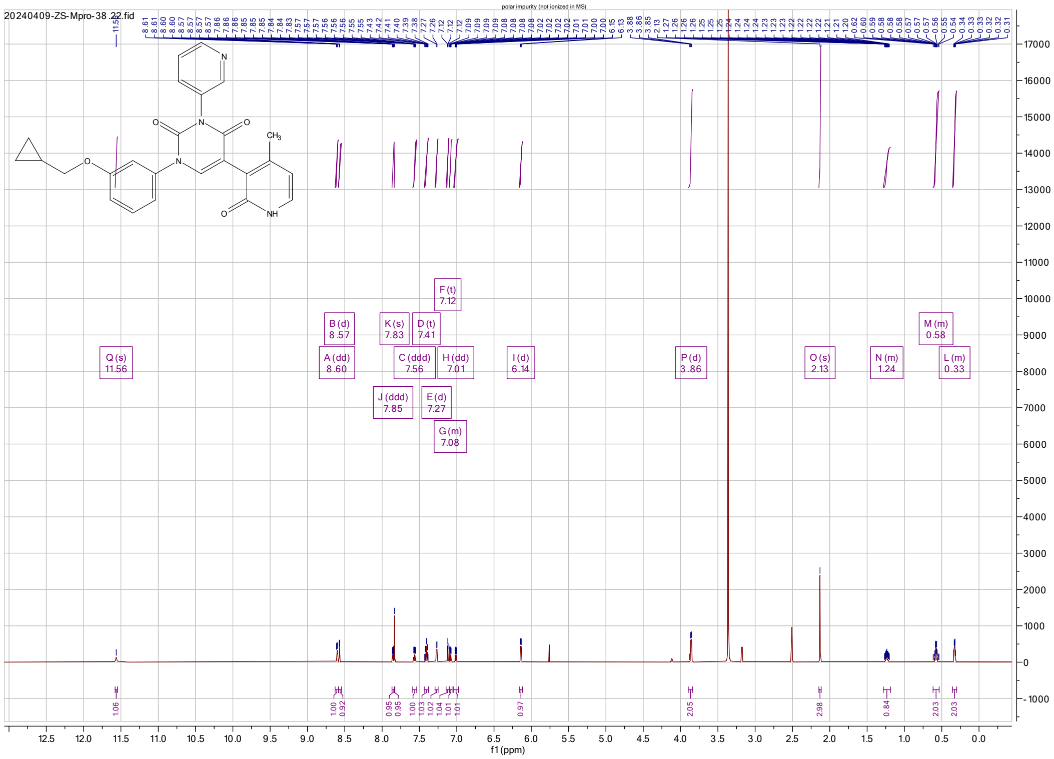

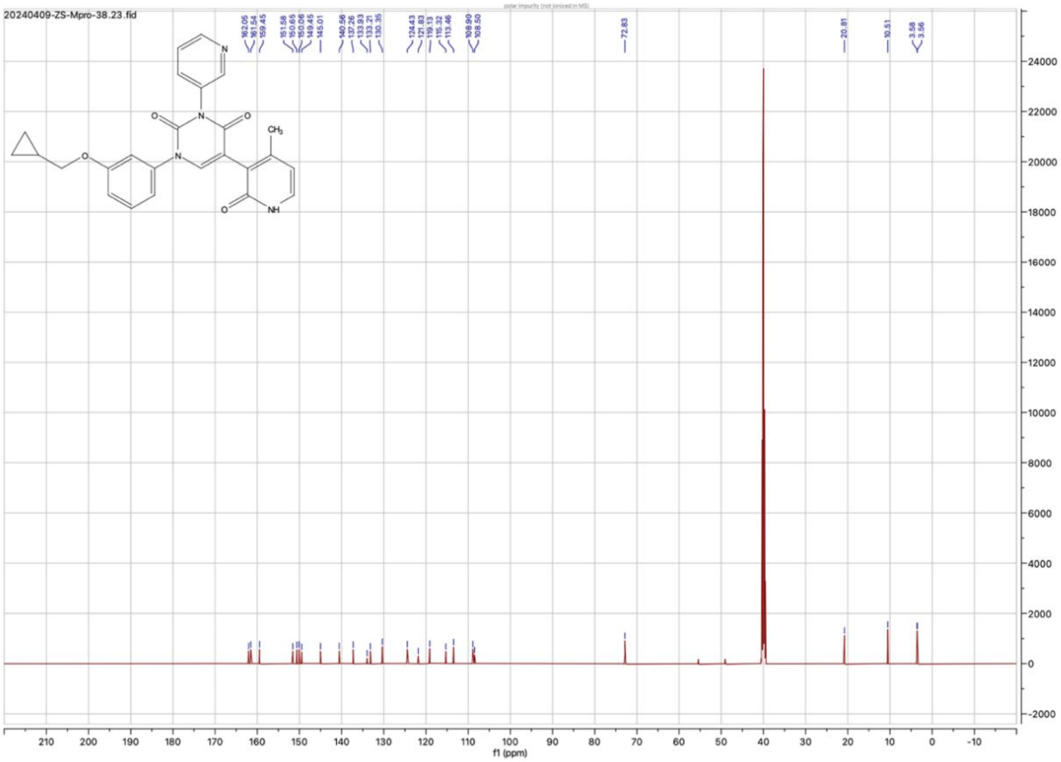

^{1}H NMR spectrum of **26**

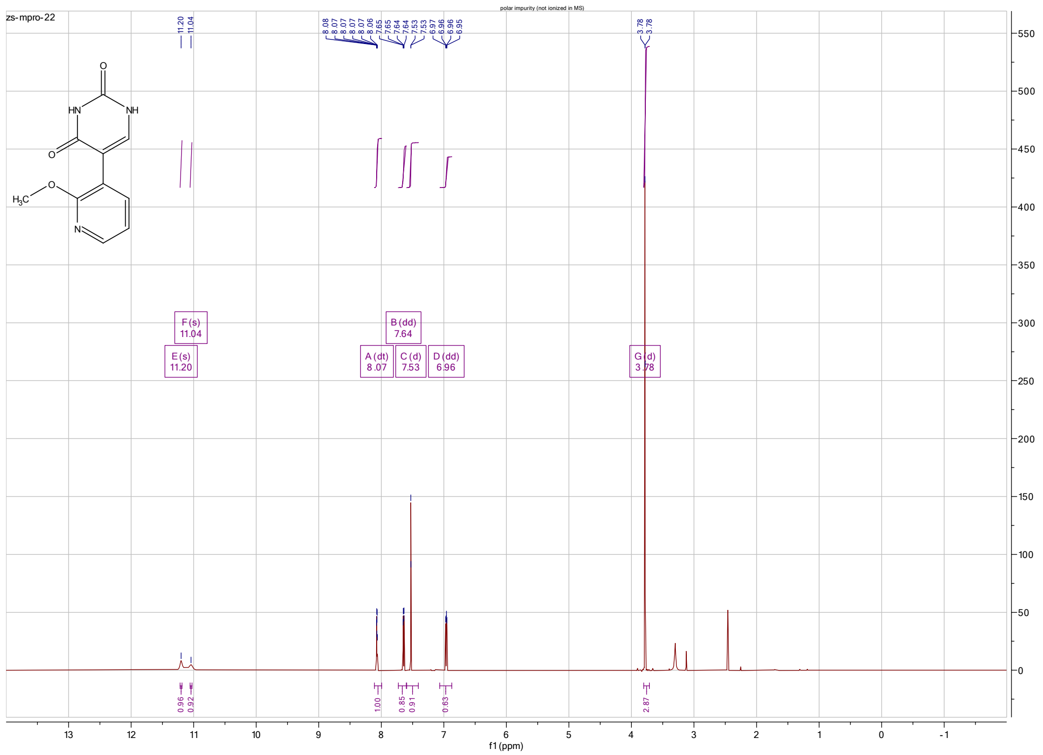

^{1}H and ^{19}F NMR spectrum of **27**

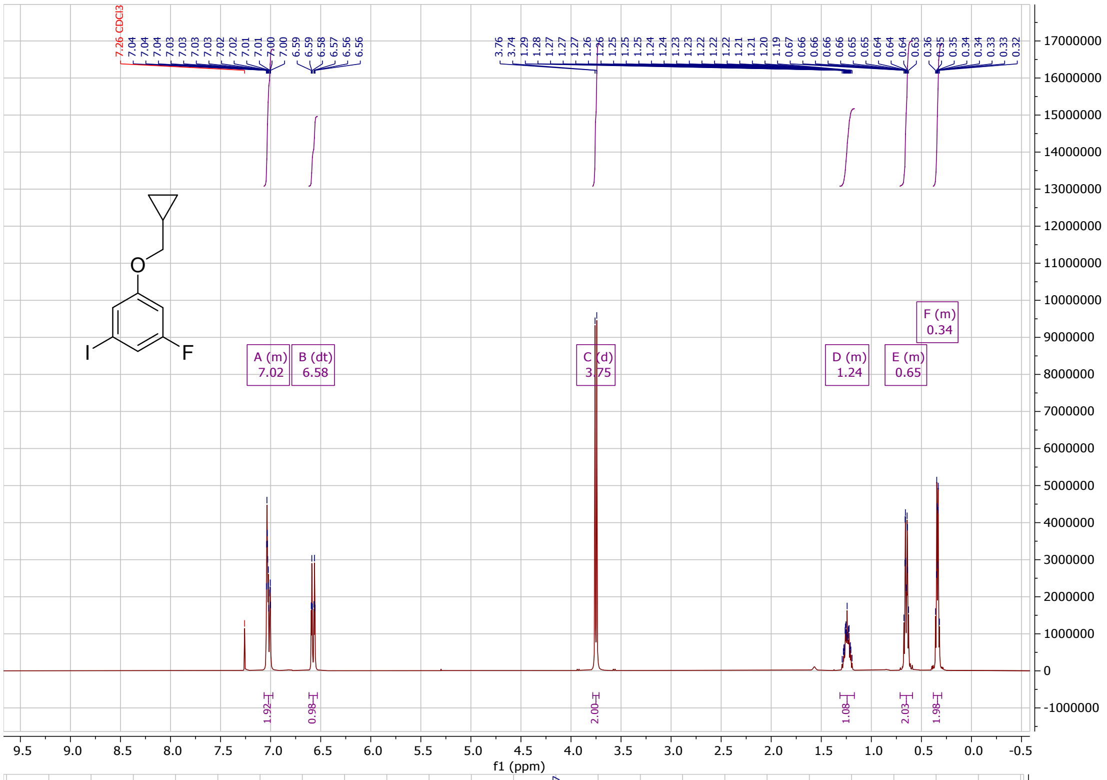

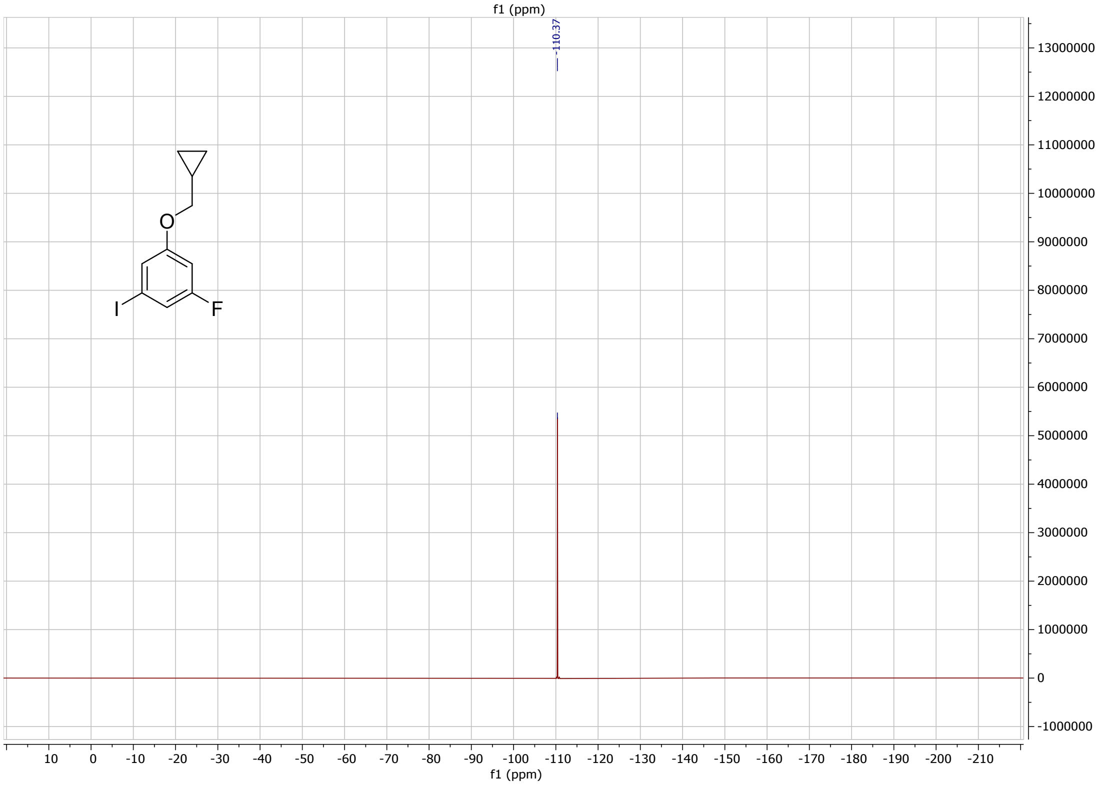

^{1}H NMR spectrum of **28**

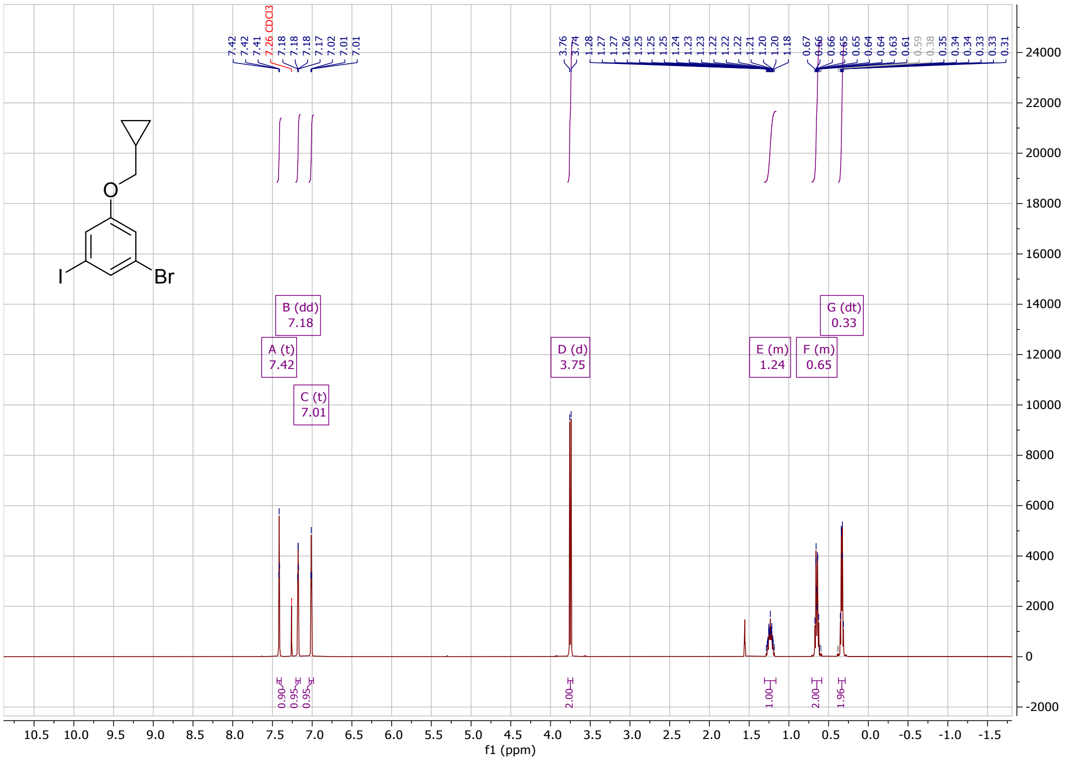

^{1}H and ^{19}F NMR spectrum of **29**

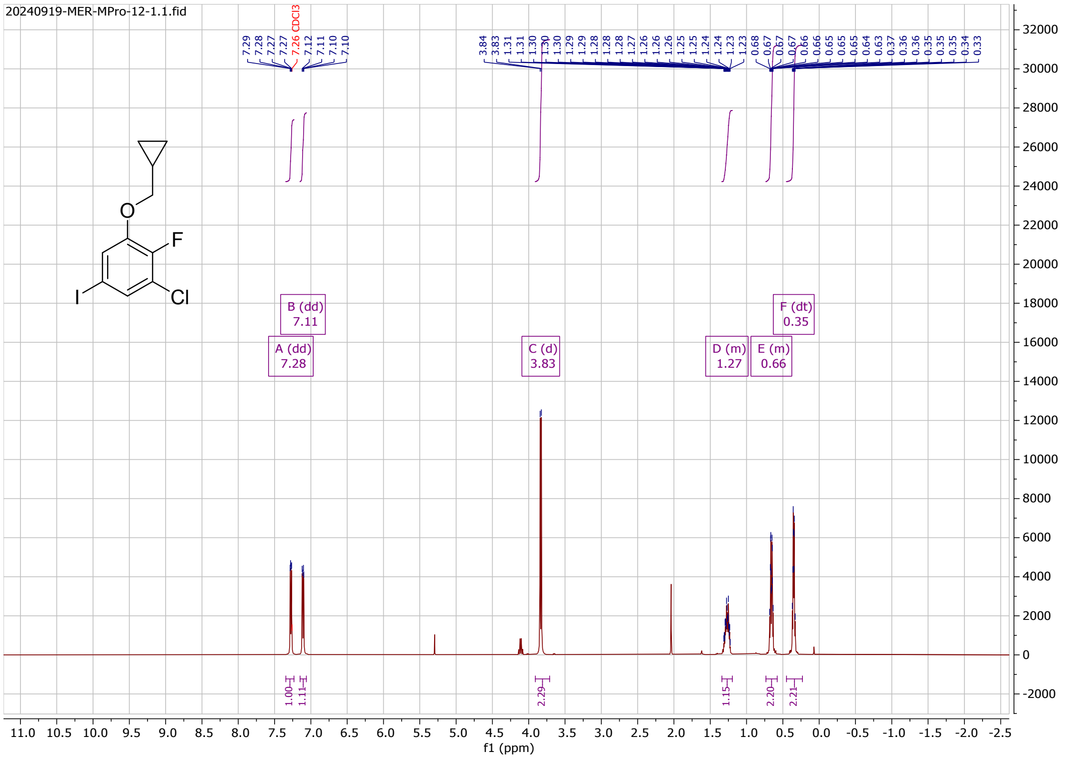

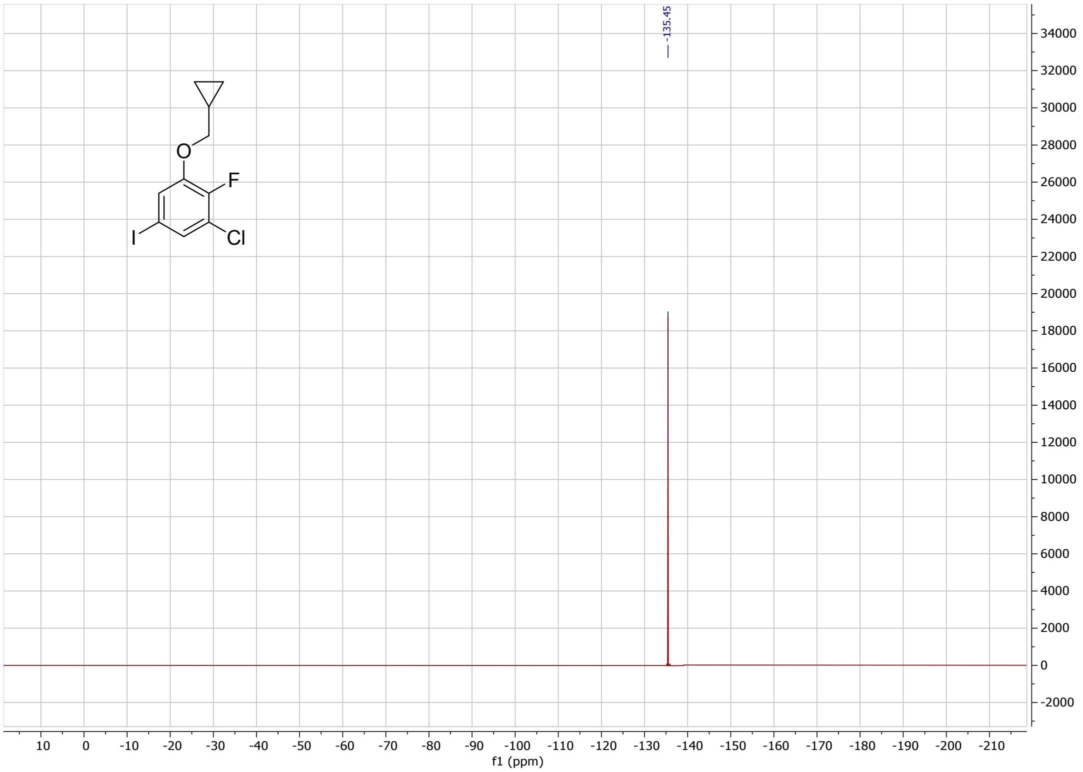

^{1}H and ^{19}F NMR spectrum of **30**

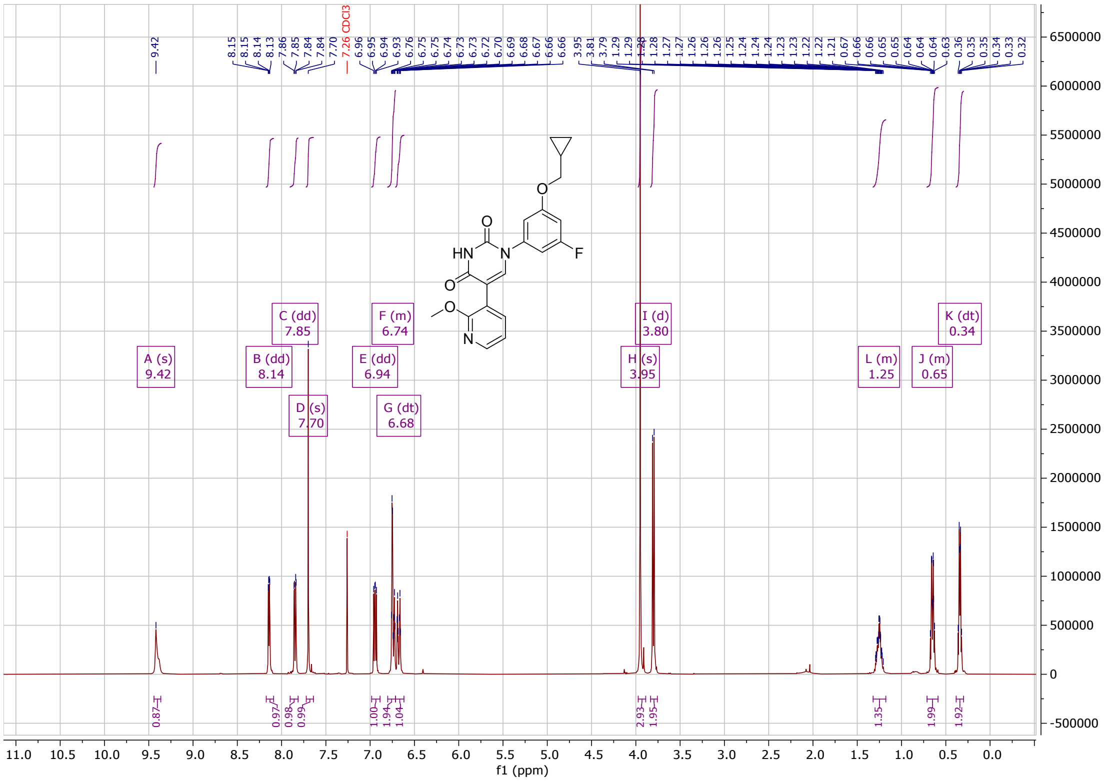

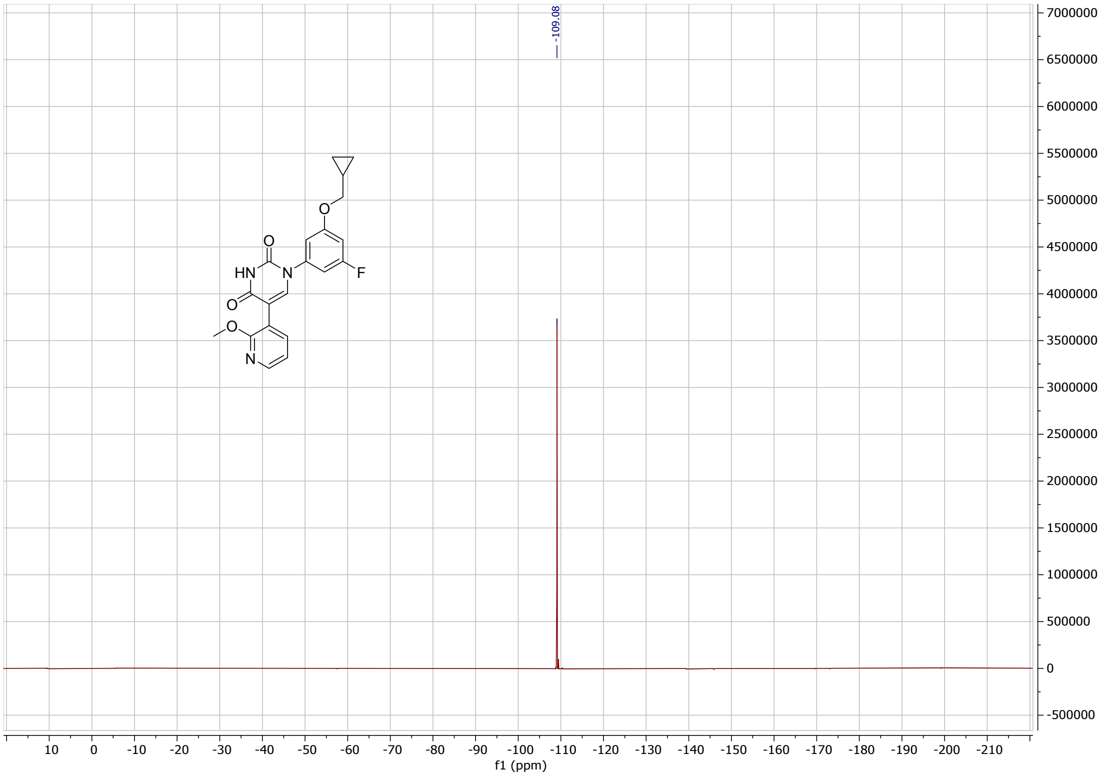

^{1}H and ^{13}C NMR spectrum of **31**

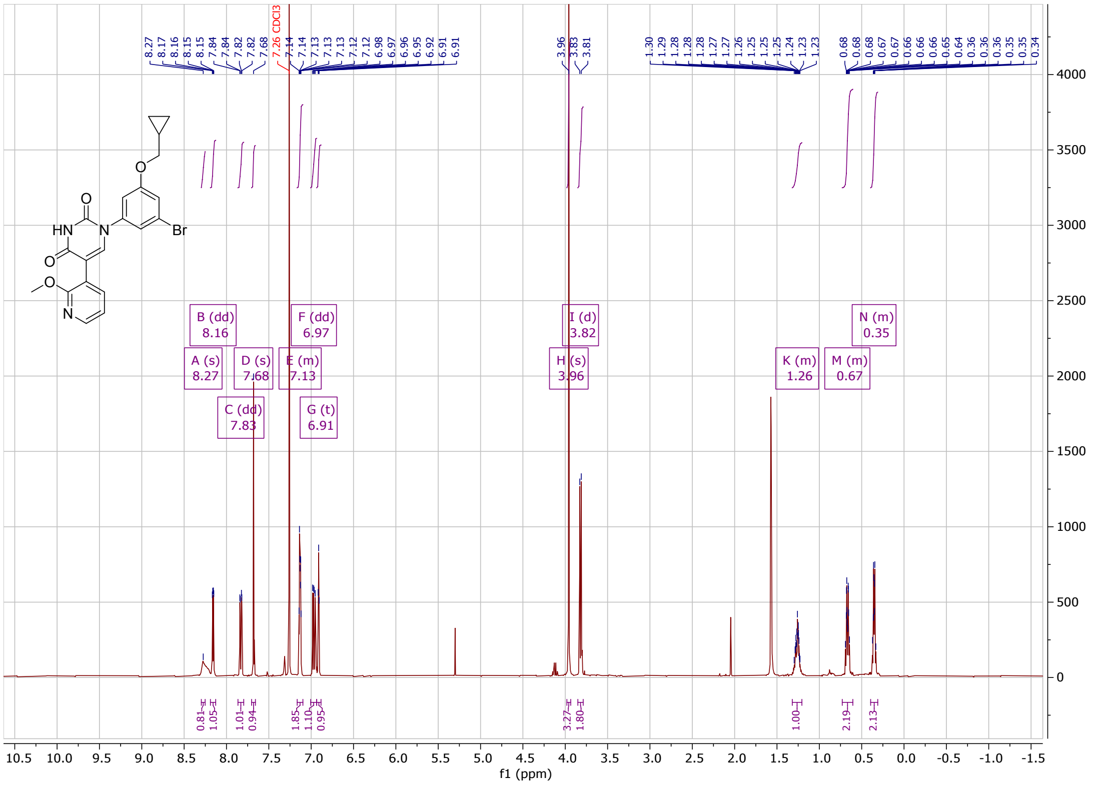

^{1}H and ^{19}F spectrum of **32**

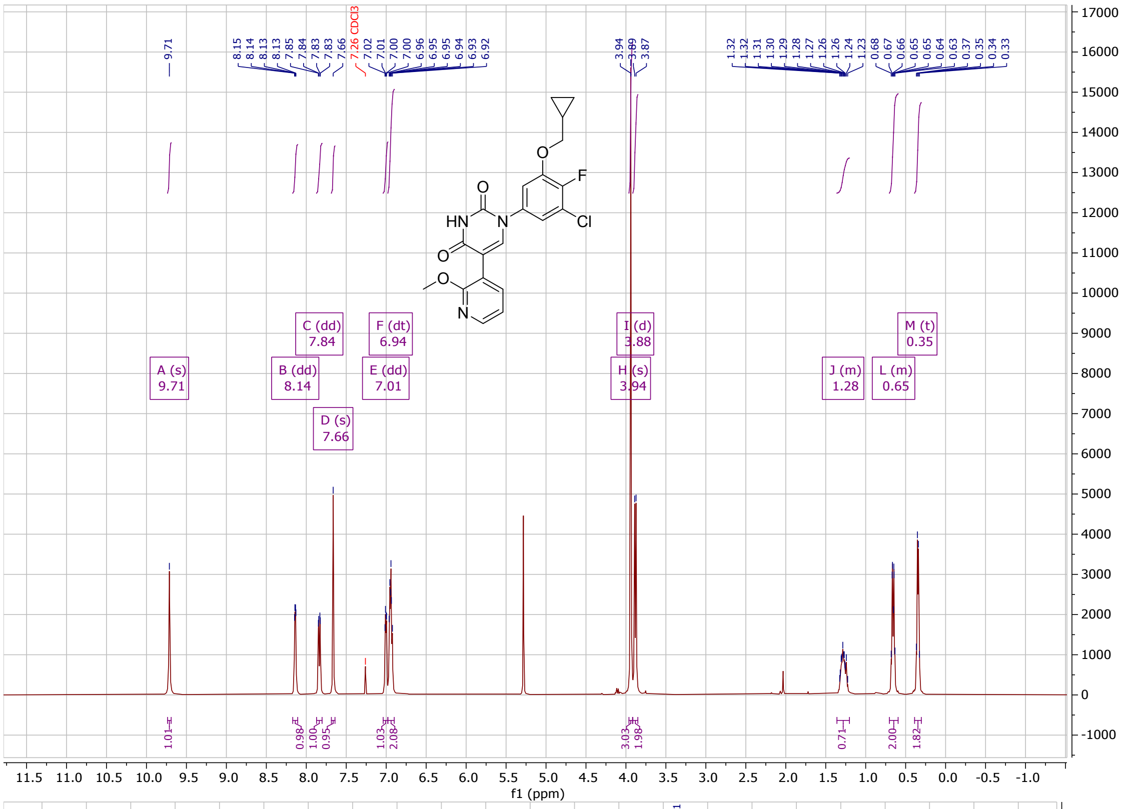

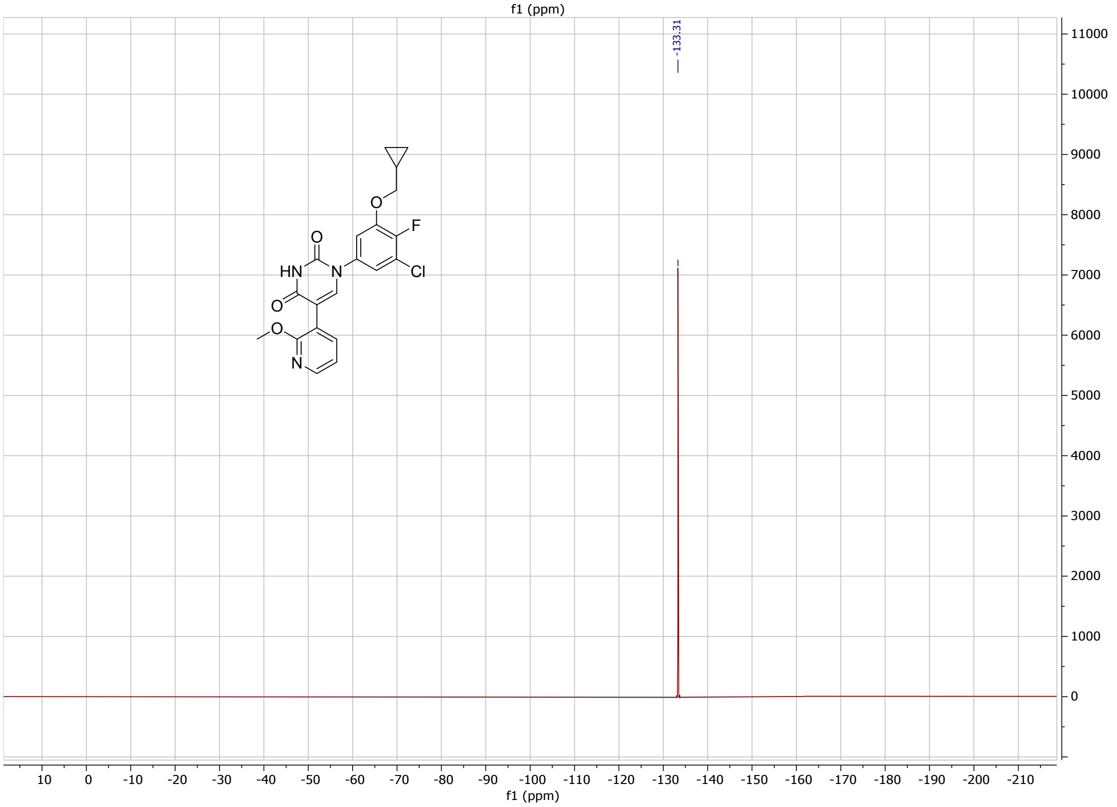

^{1}H and ^{19}F NMR spectrum of **33**

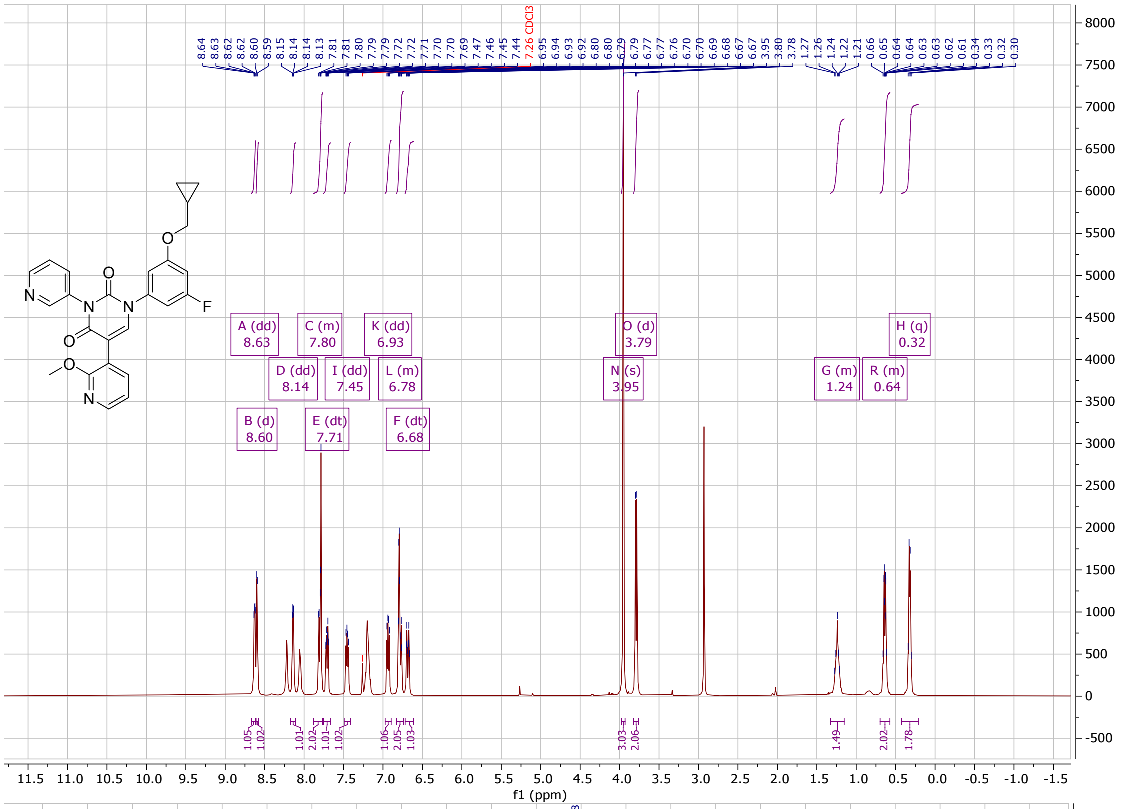

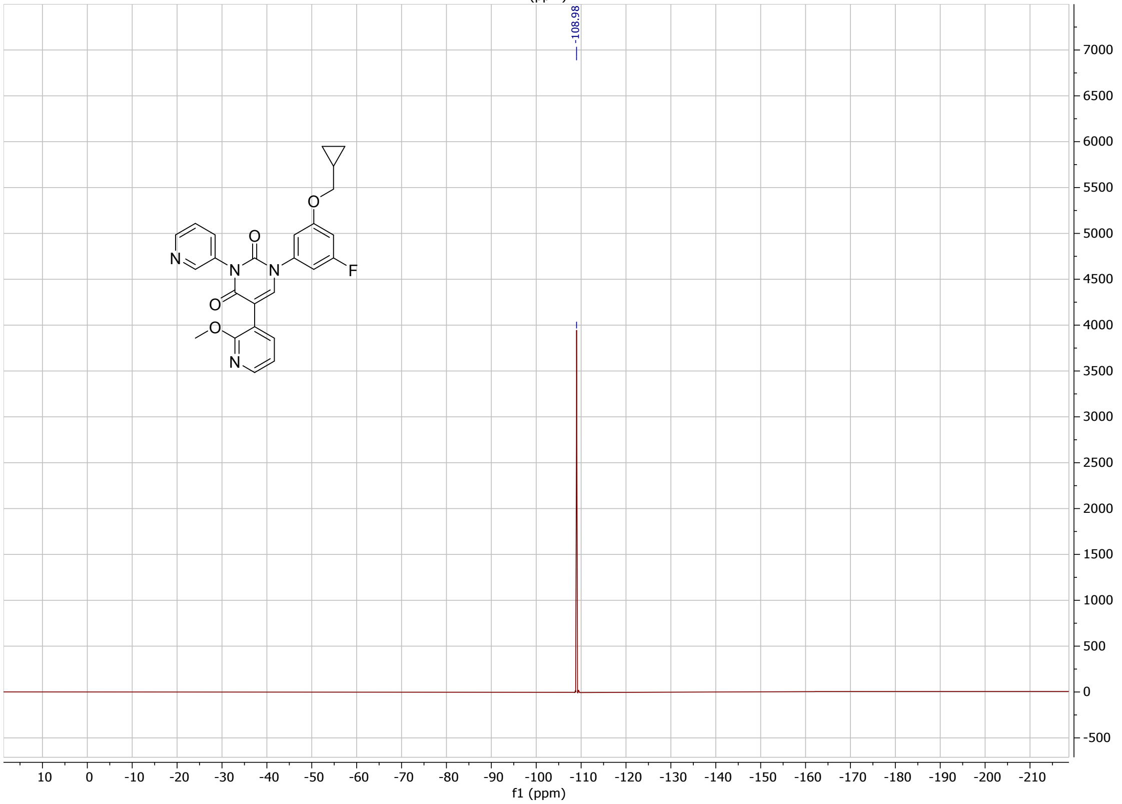

^{1}H NMR spectrum of **34**

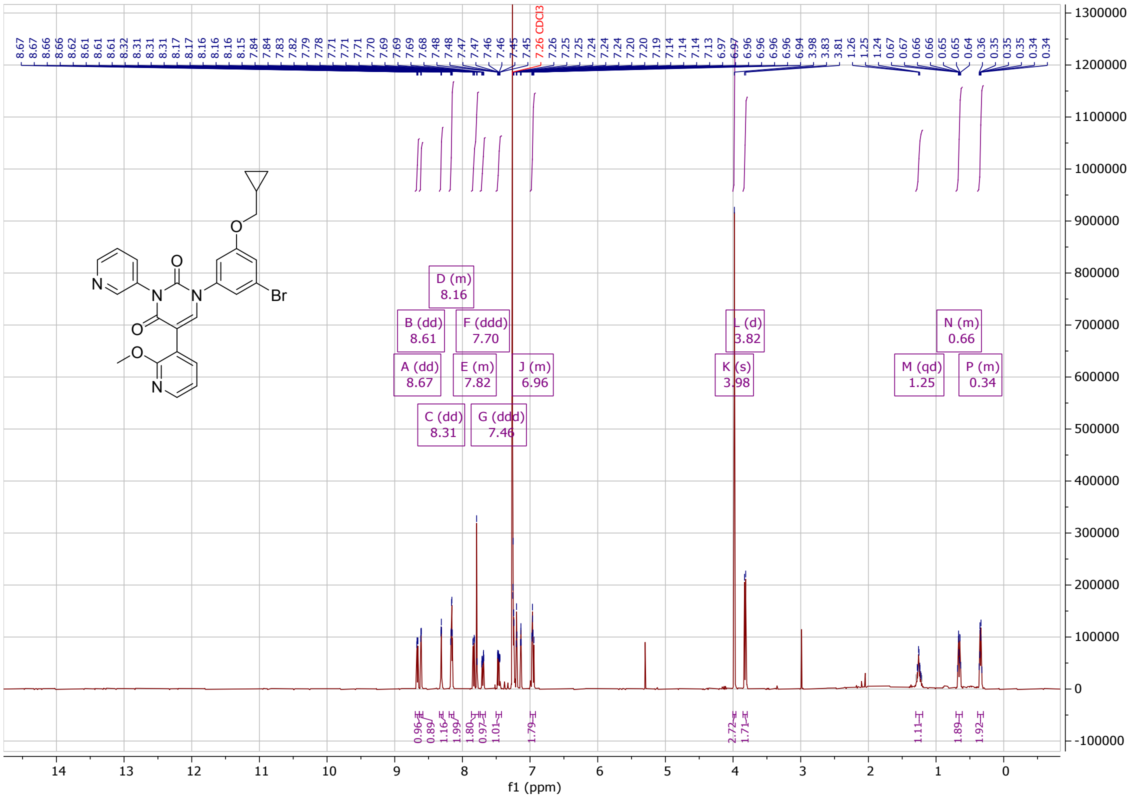

^{1}H, ^{13}C and ^{19}F NMR spectrum of **36**

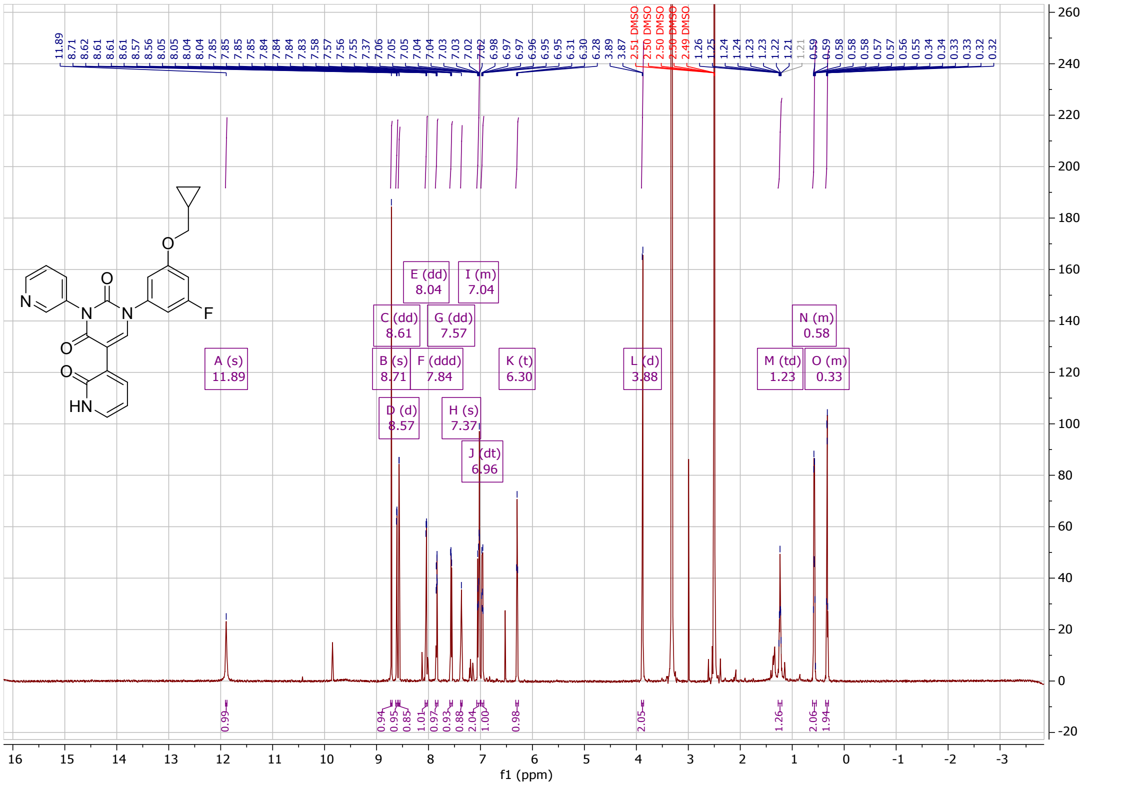

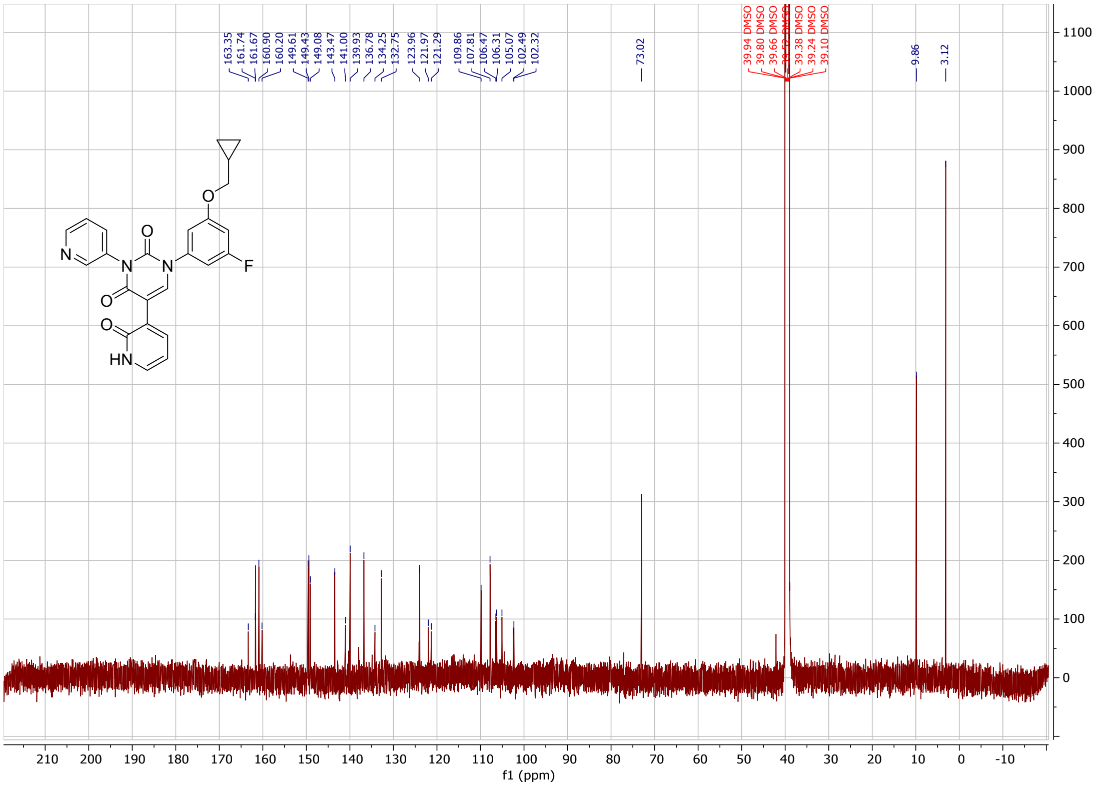
163.35
161.74
161.67
160.90
160.20
149.61
149.43
149.08
143.47
141.00
139.93
136.78
134.25
132.75
123.96
121.97
121.29
109.86
107.81
106.47
106.31
105.07
102.49
102.32
73.02
39.94 DMSO
39.80 DMSO
39.66 DMSO
39.38 DMSO
39.24 DMSO
39.10 DMSO
9.86
3.12
N
O
N
N
O
O
F
O
HN
f1 (ppm)

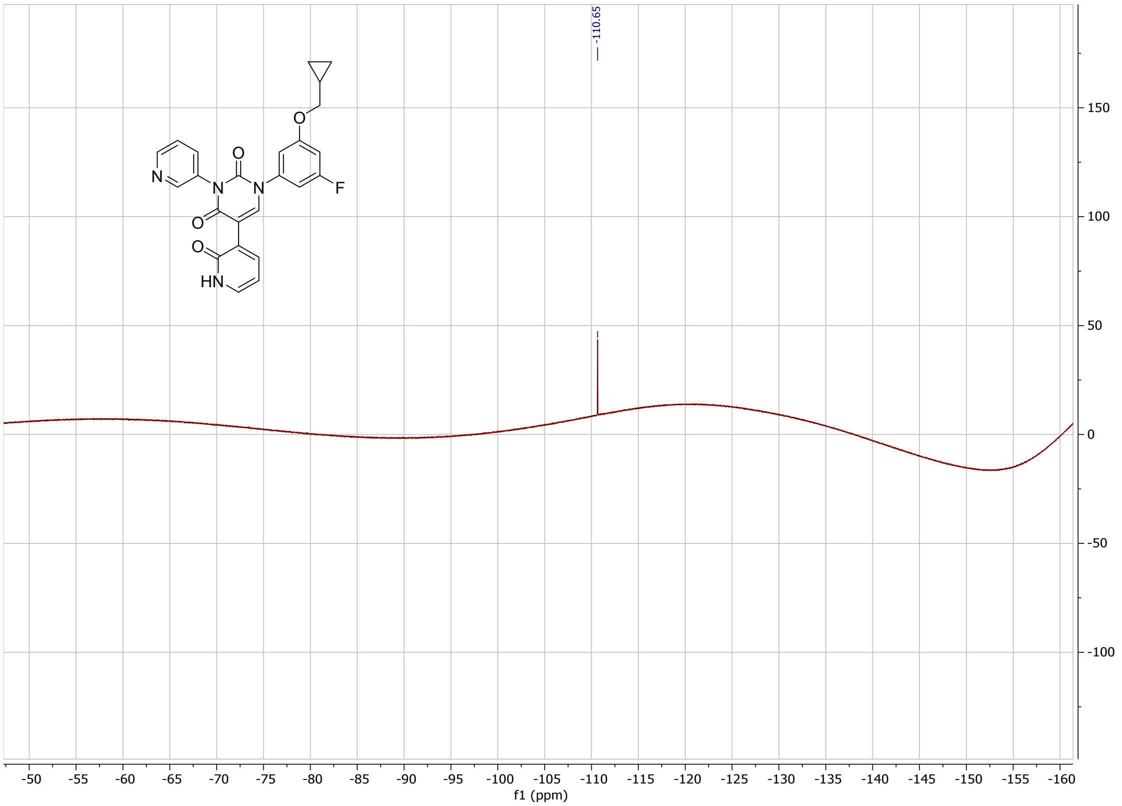
-110.65
N
O
N
N
O
O
F
O
HN
f1 (ppm)

^{1}H, ^{13}C NMR spectrum of **37**

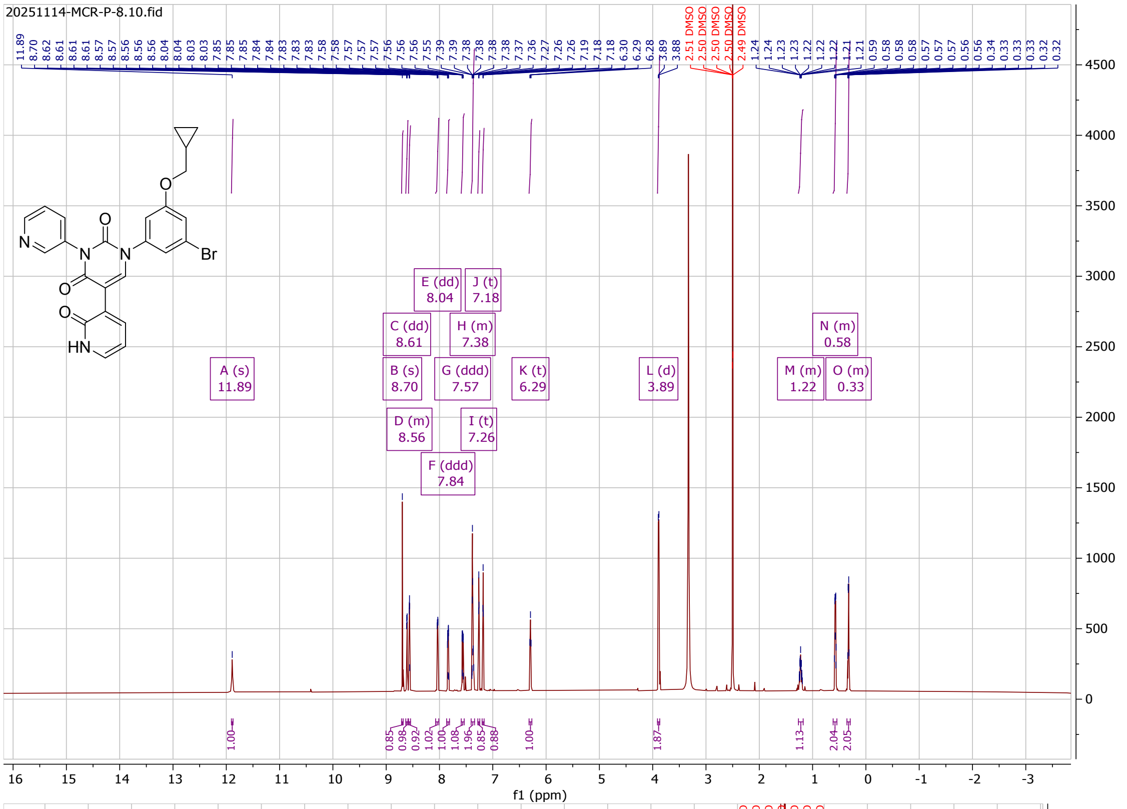

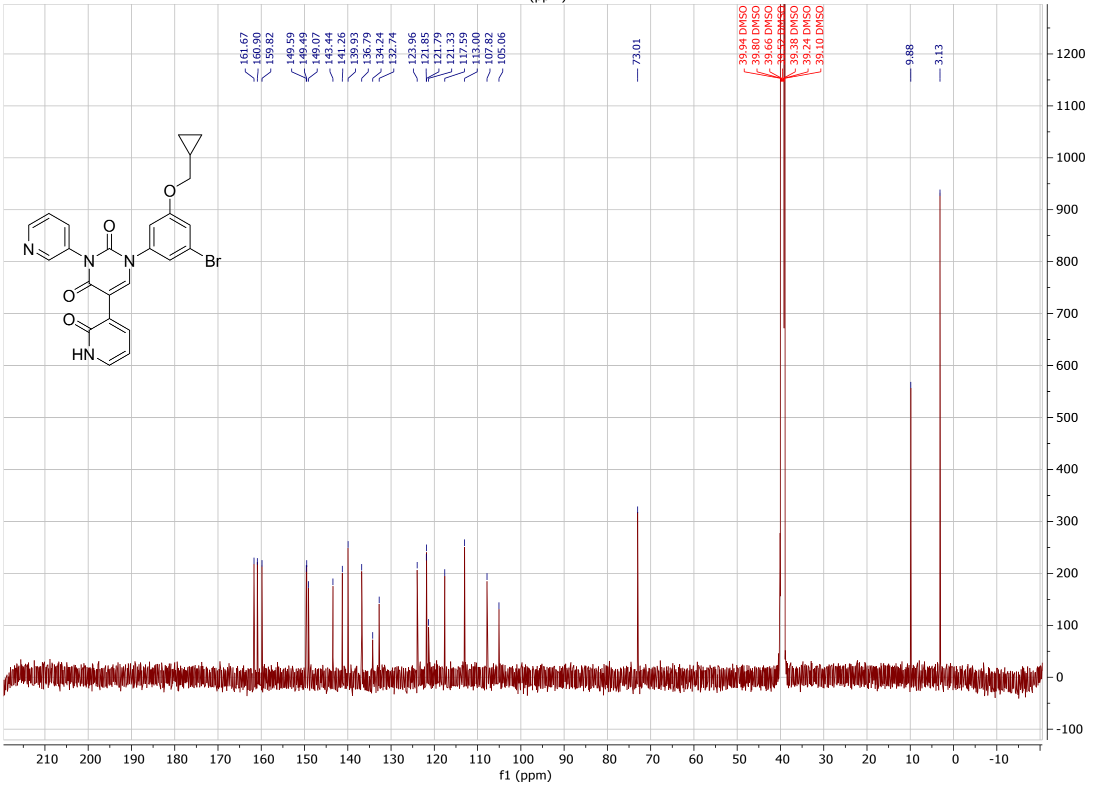

^{1}H, ^{13}C and ^{19}F NMR spectrum of **38**

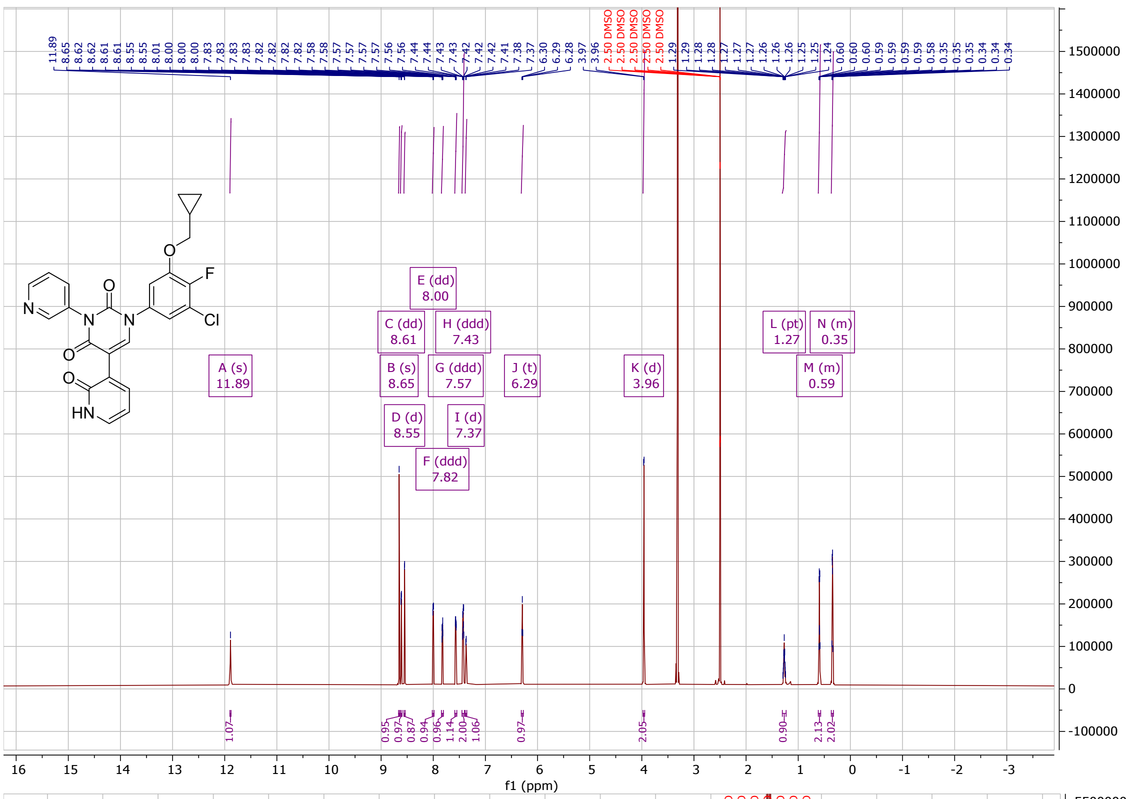

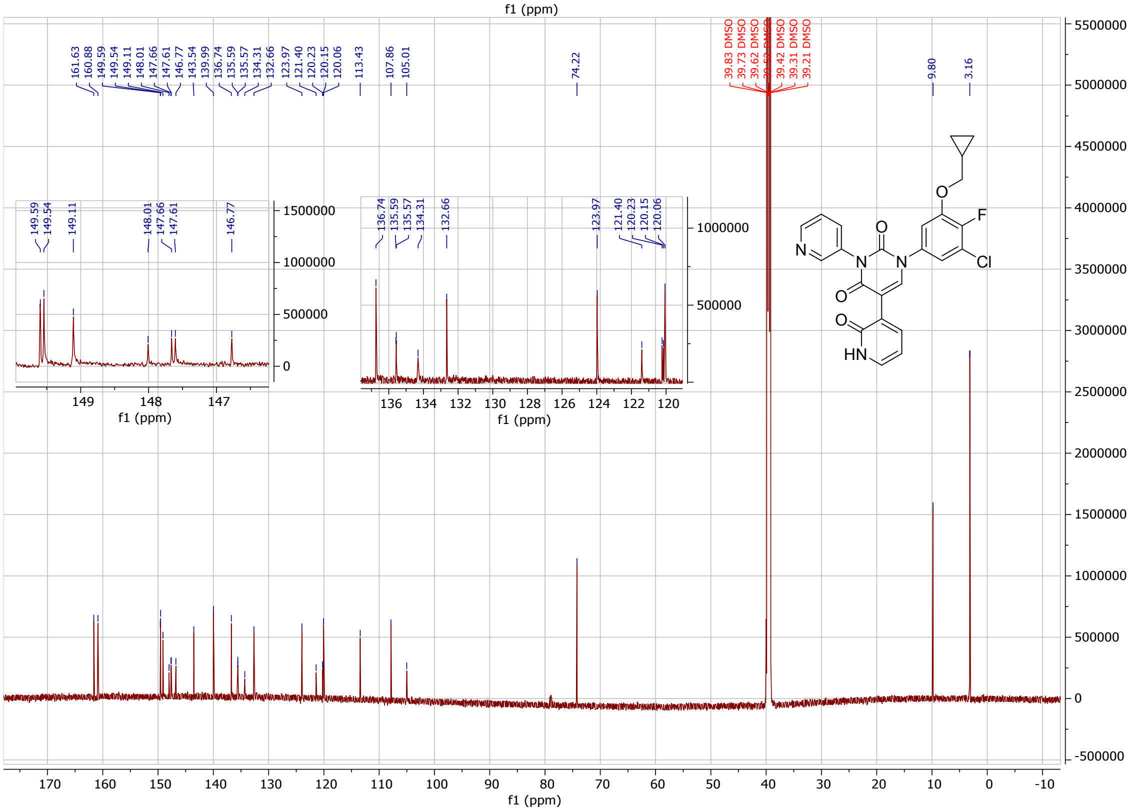

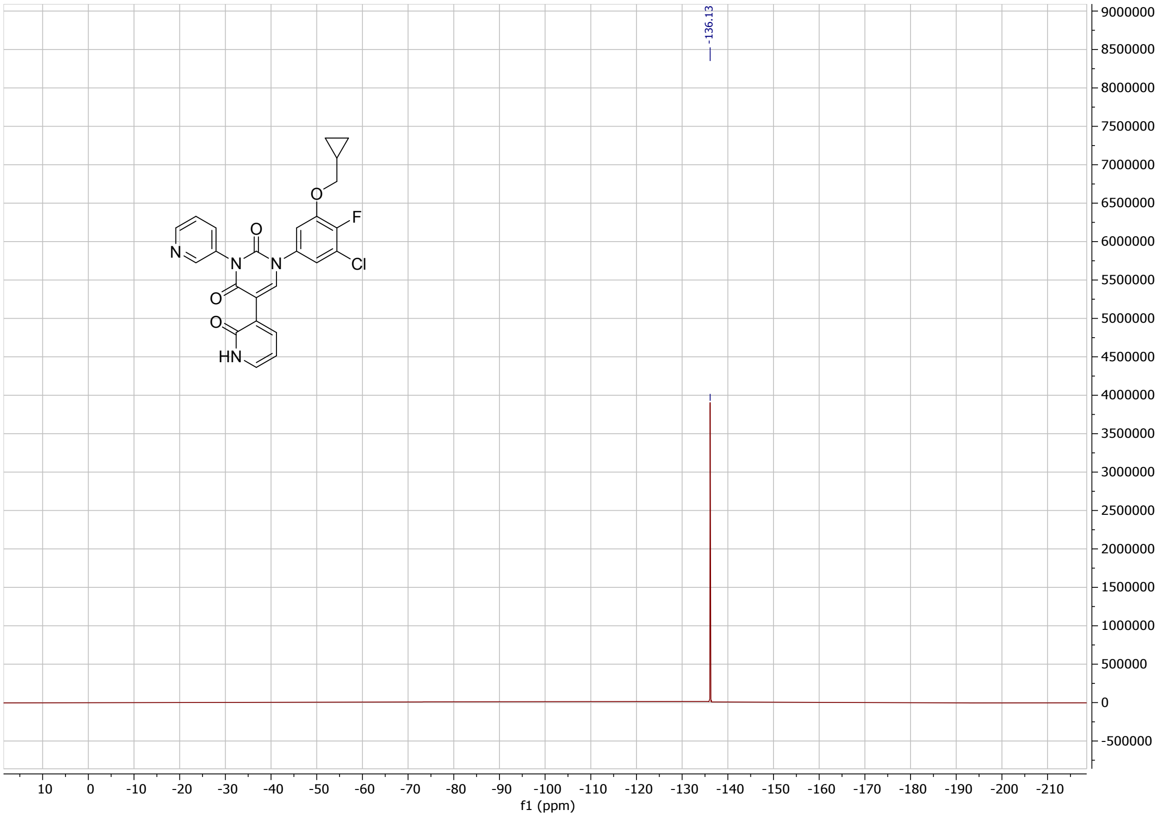

^{1}H NMR spectrum of **S5**

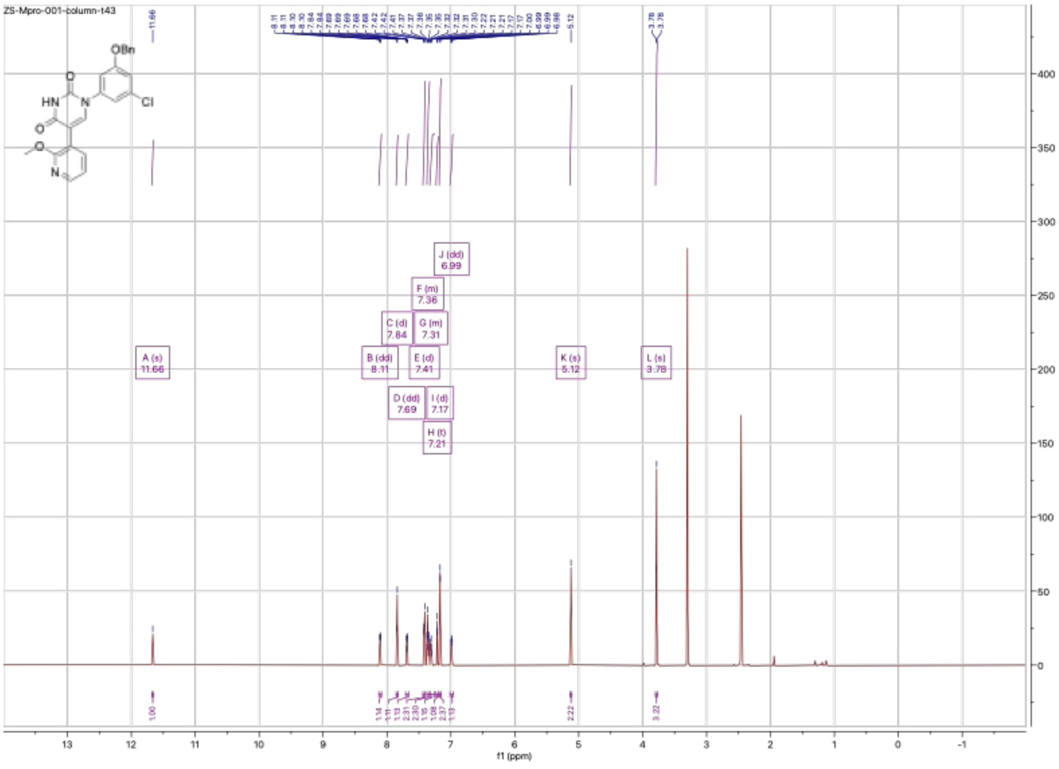

^{1}H NMR spectrum of **S6**

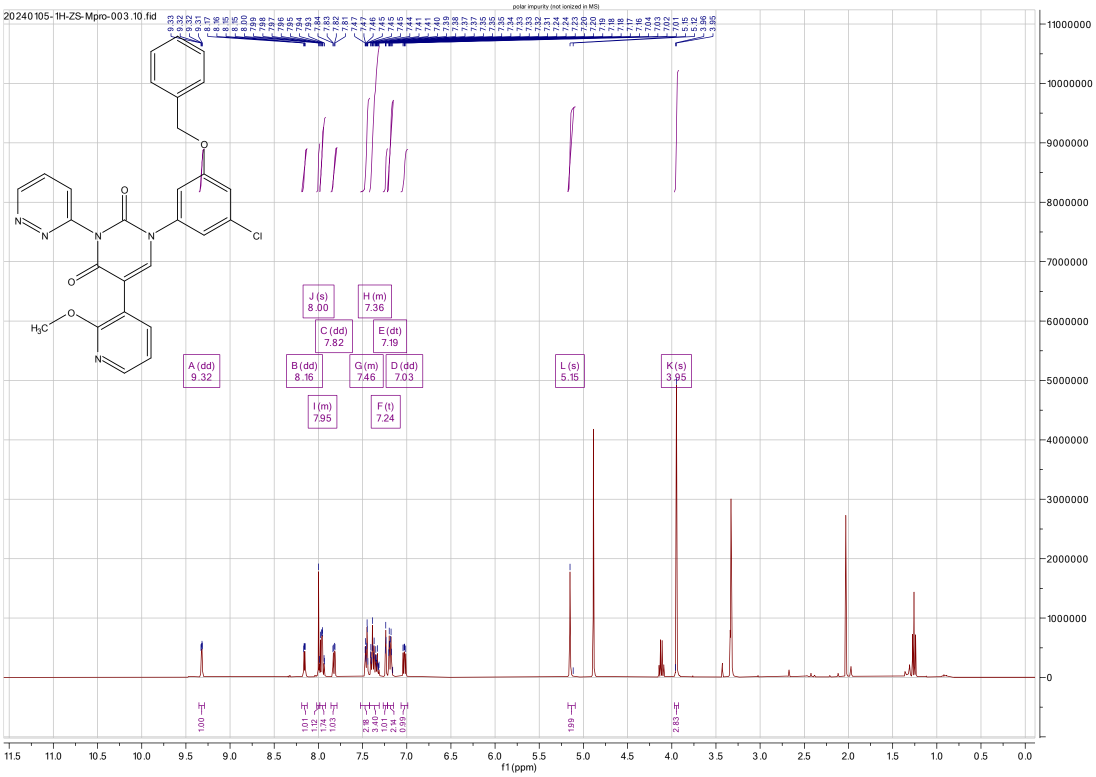

^{1}H NMR spectrum of **S7**

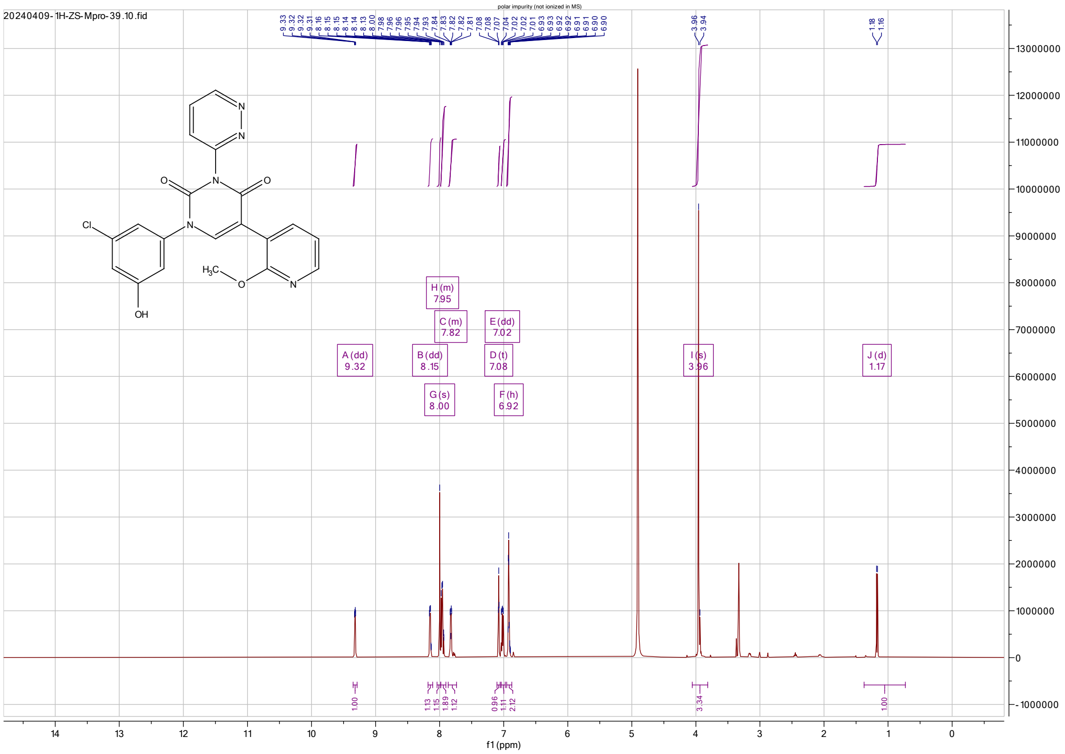

^{1}H NMR spectrum of **S8**

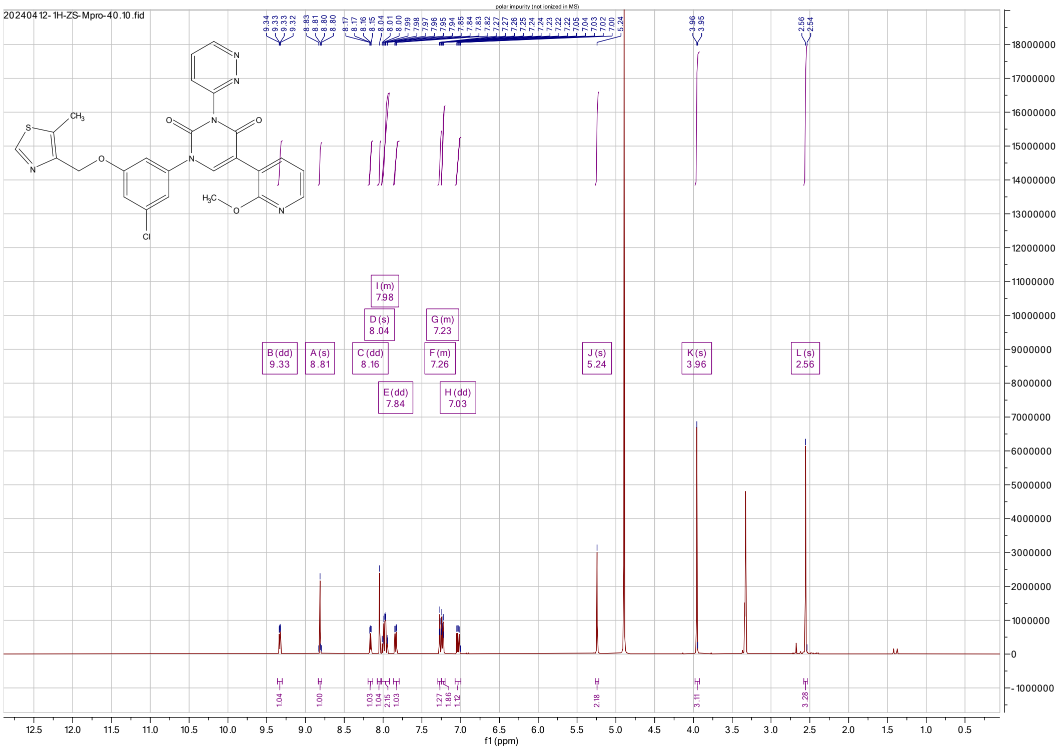

^{1}H and ^{19}F NMR spectrum of **S9**

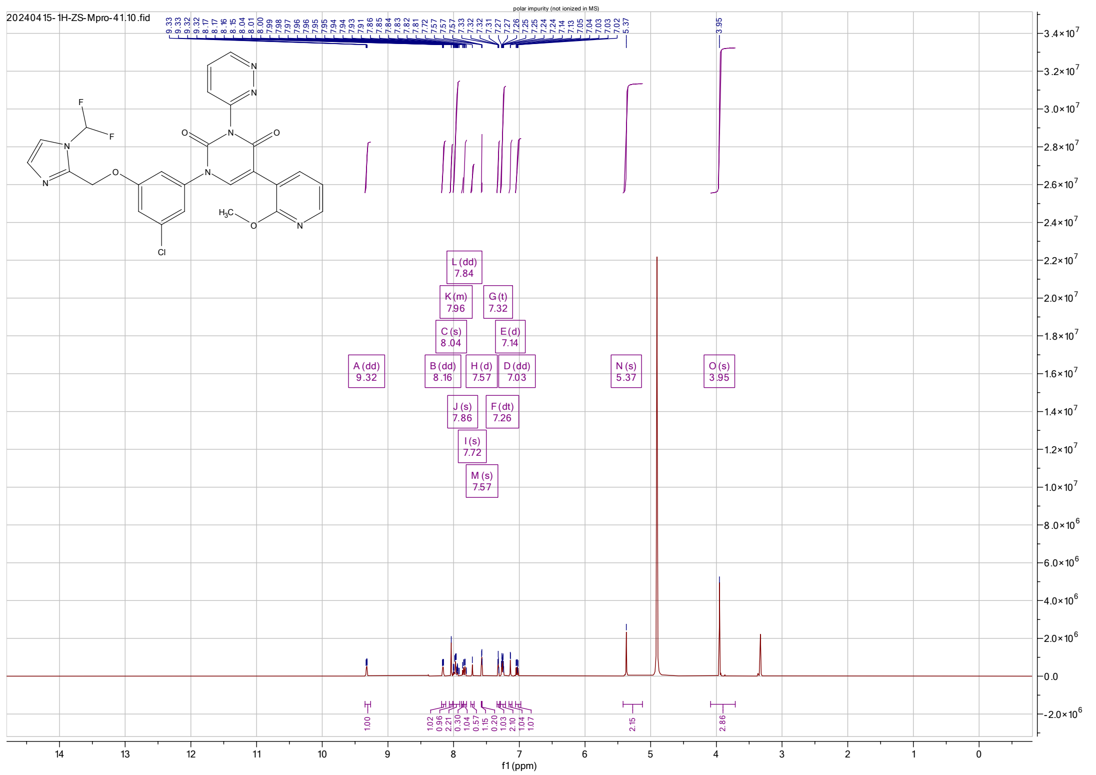

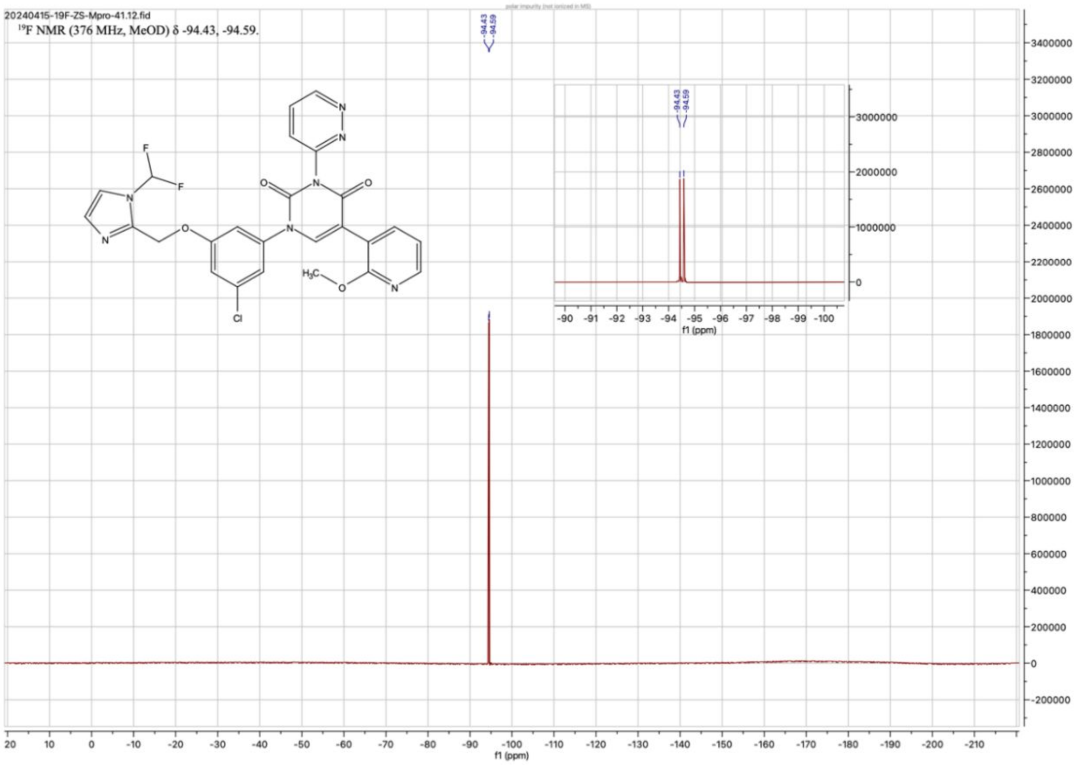

^{1}H NMR spectrum of **S10**

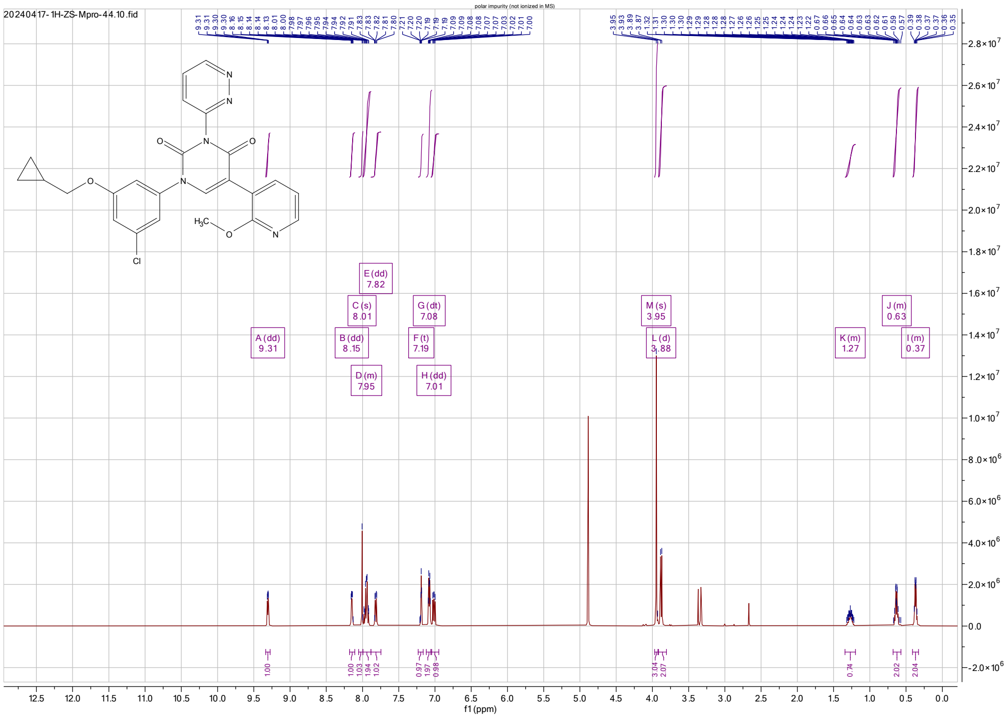

^{1}H NMR spectrum of **S11**

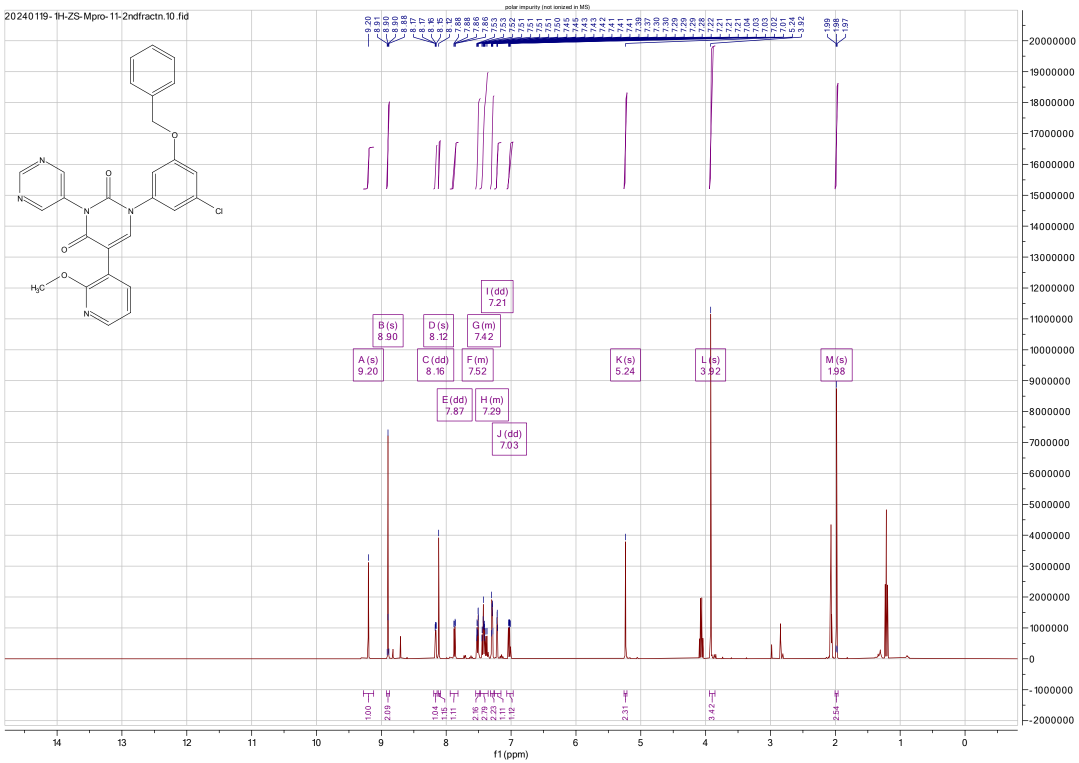

^{1}H NMR spectrum of **S12**

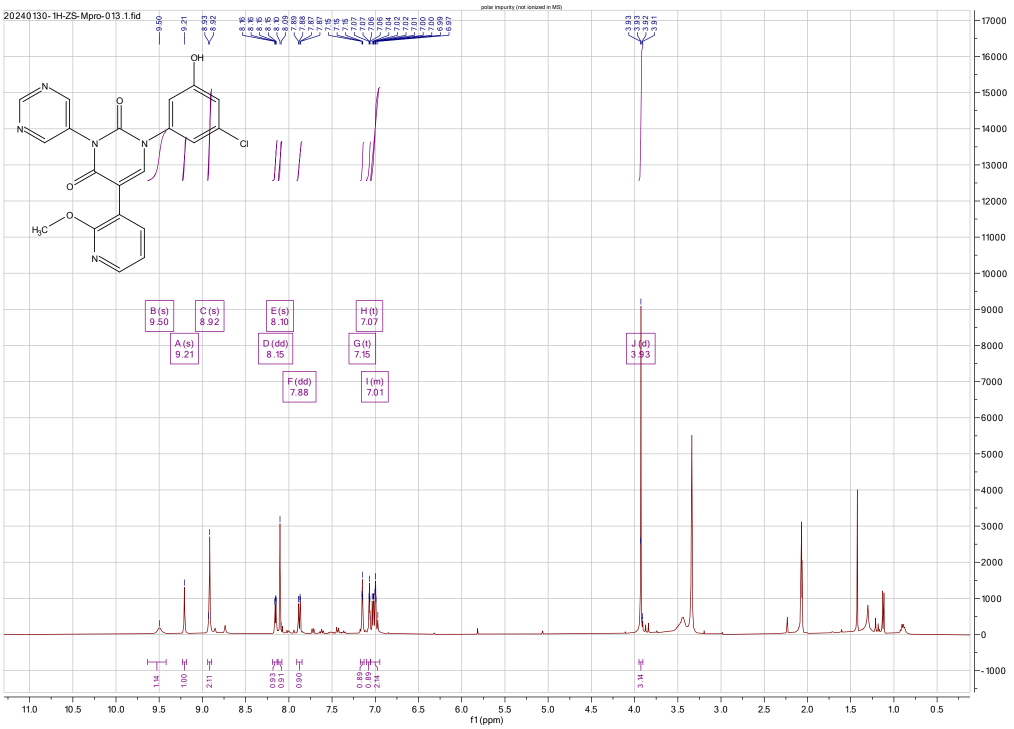

^{1}H NMR spectrum of **S13**

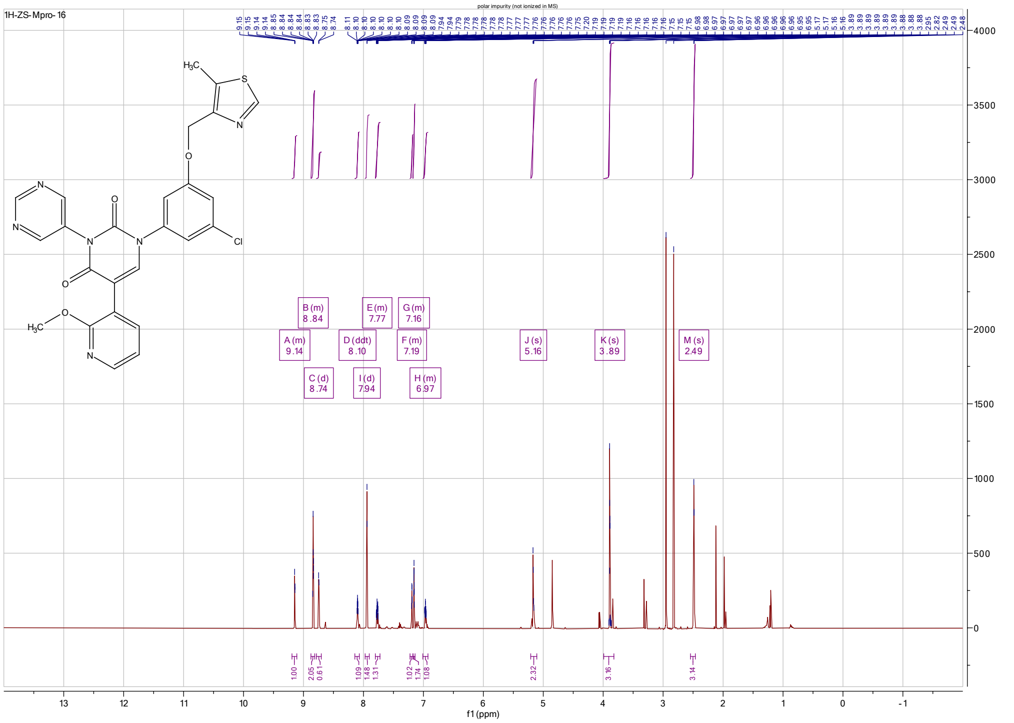

^{1}H and ^{13}C NMR spectrum of **41**

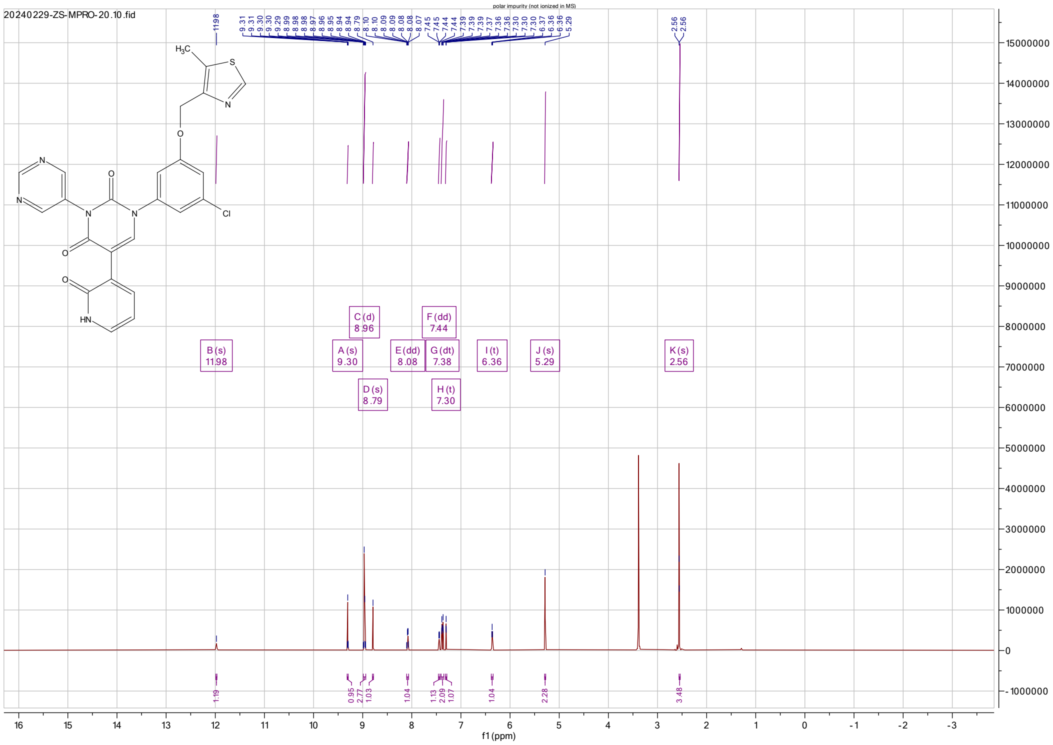

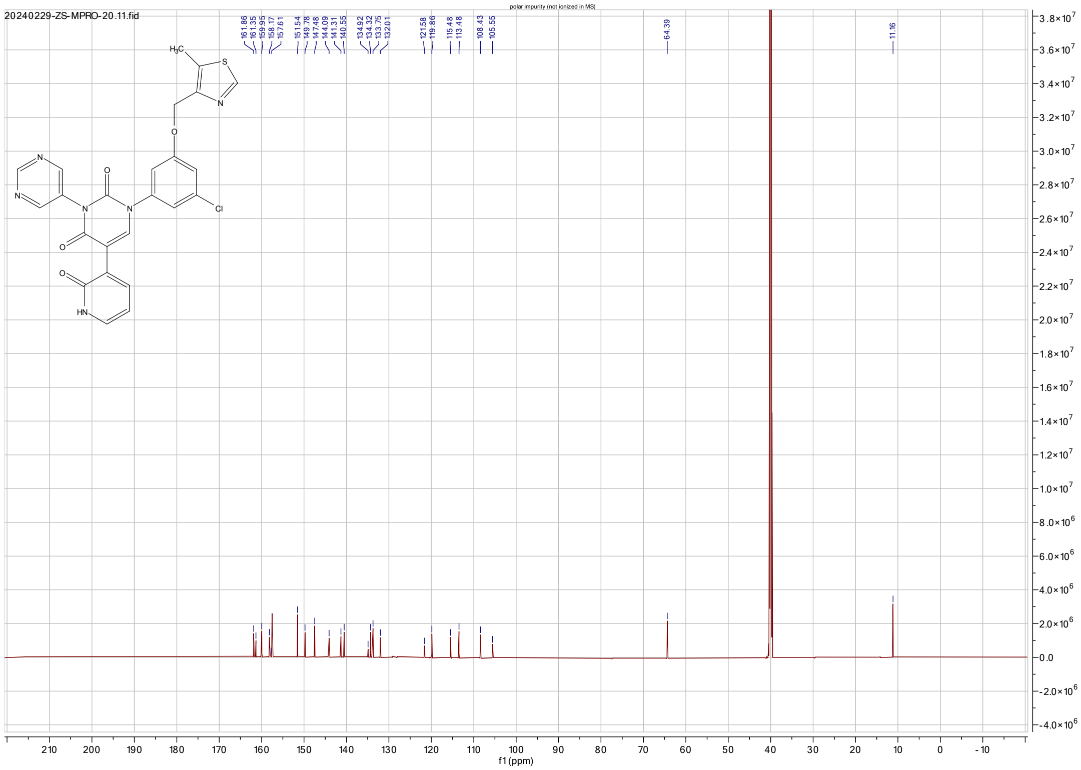

^{1}H and ^{13}C NMR spectrum of **42**

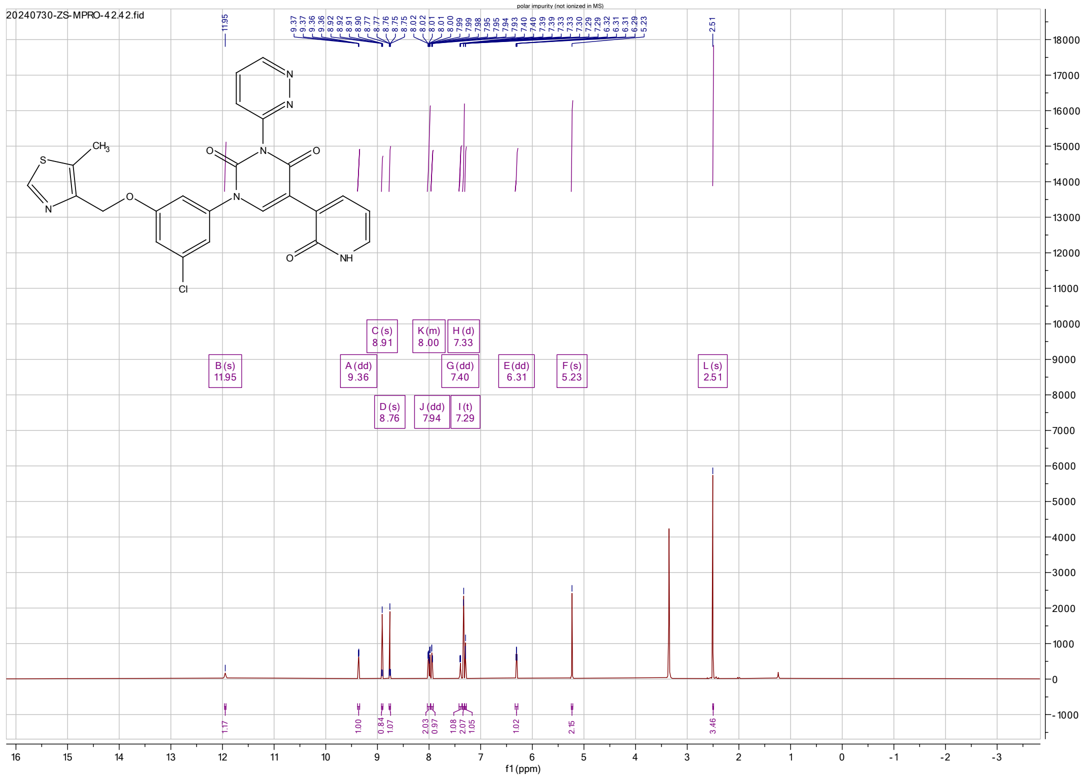

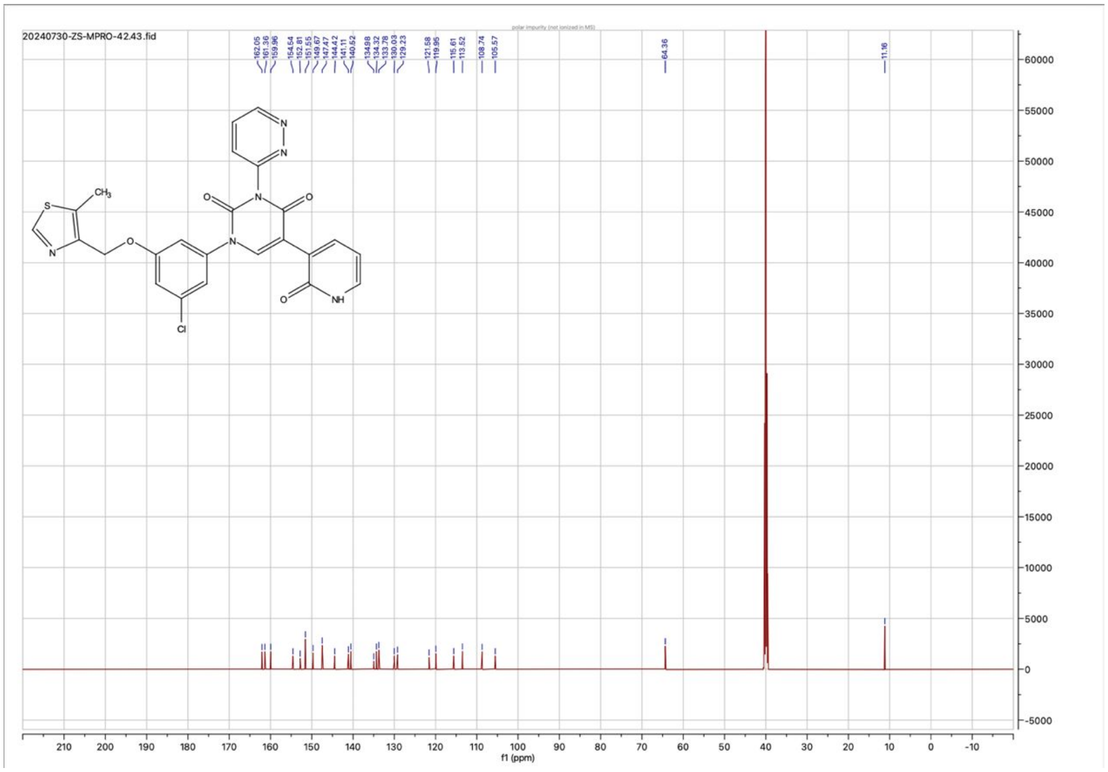

^{1}H, ^{19}F and ^{13}C NMR spectrum of **43**

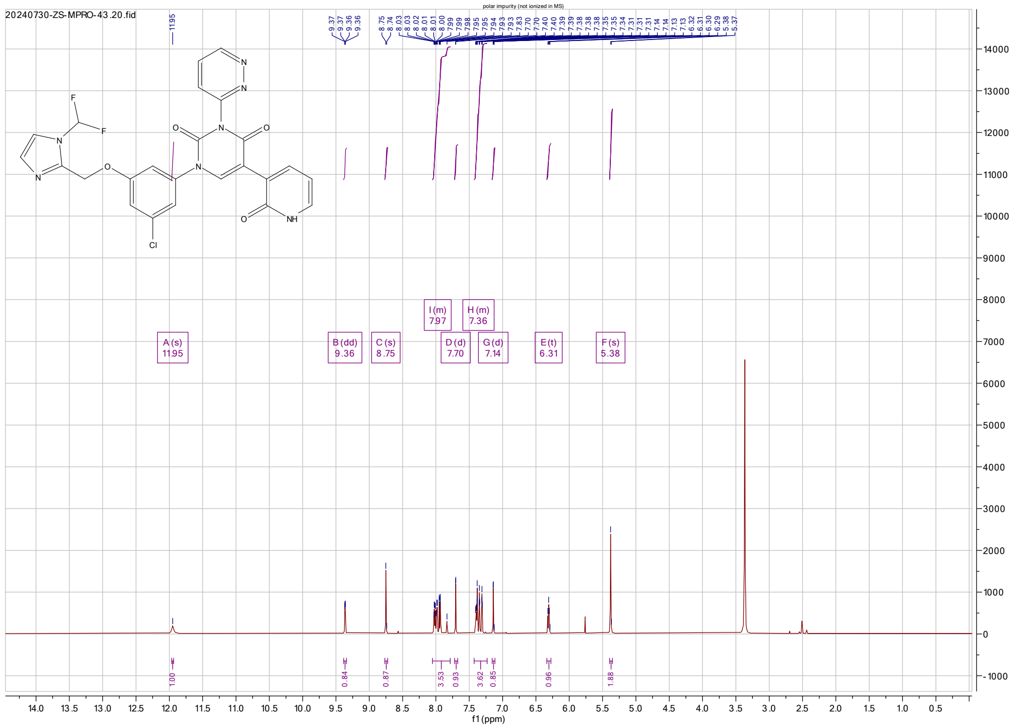

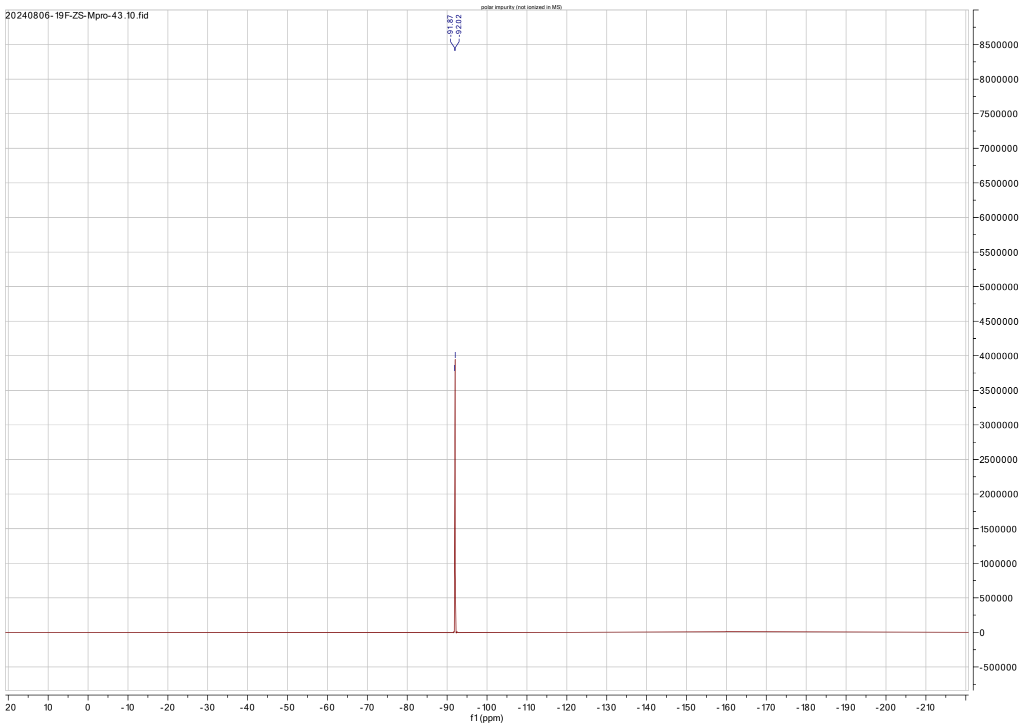

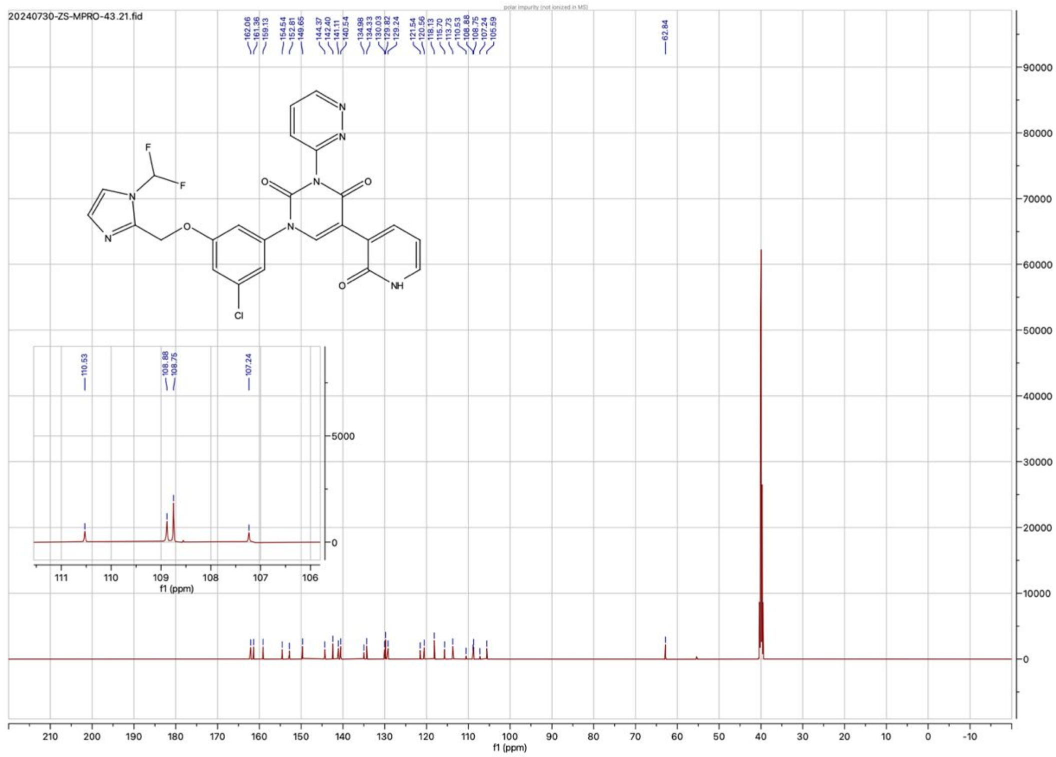

^1H and ^{13}C NMR spectrum of **44**

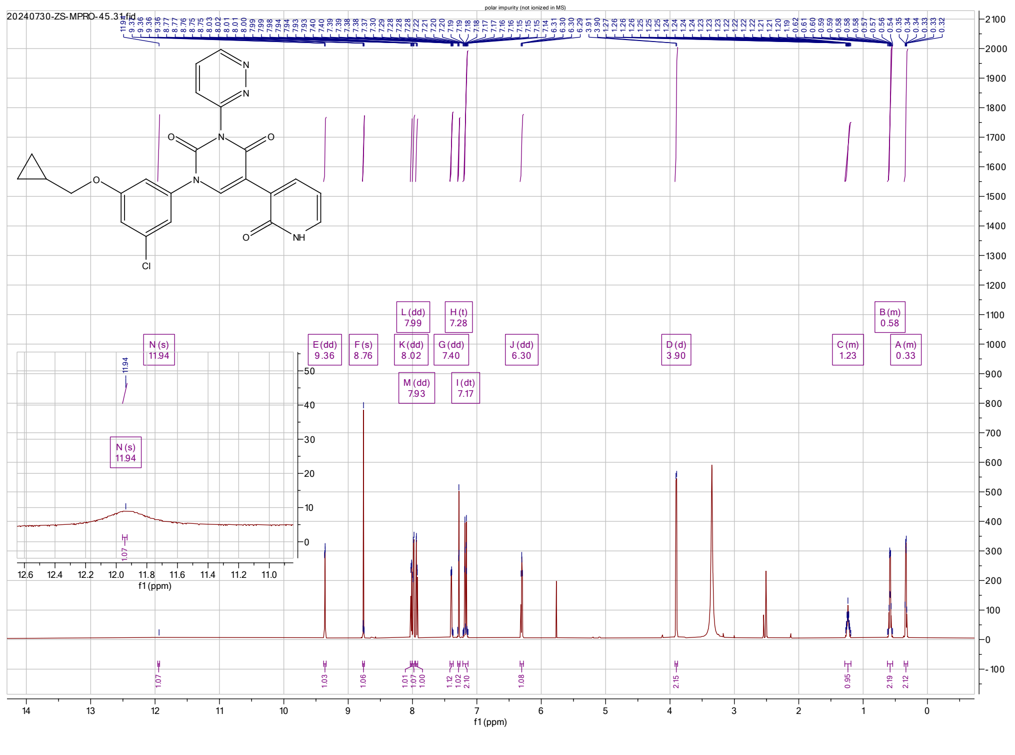

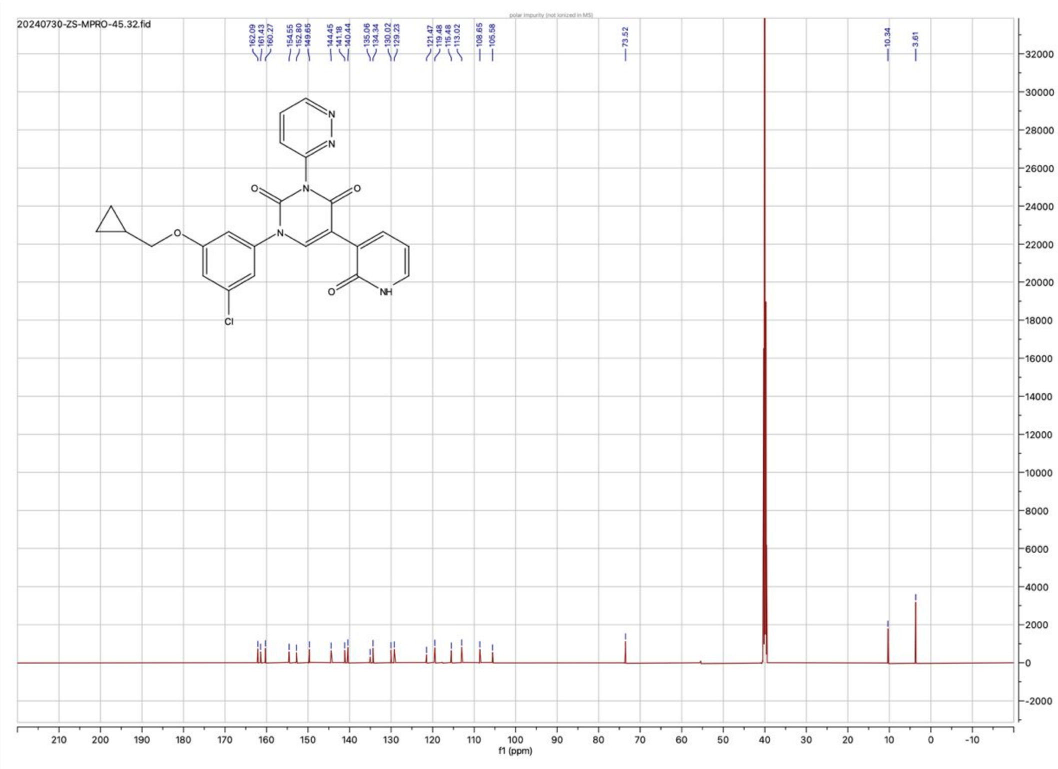
20240730-ZS-MPRO-45.32.fid
162.09
161.43
160.27
154.55
152.80
149.65
144.45
141.18
140.44
135.06
134.34
130.02
129.23
121.47
119.48
115.48
113.02
108.65
105.58
73.52
10.34
3.61
f1 (ppm)

^{1}H NMR spectrum of **S15**

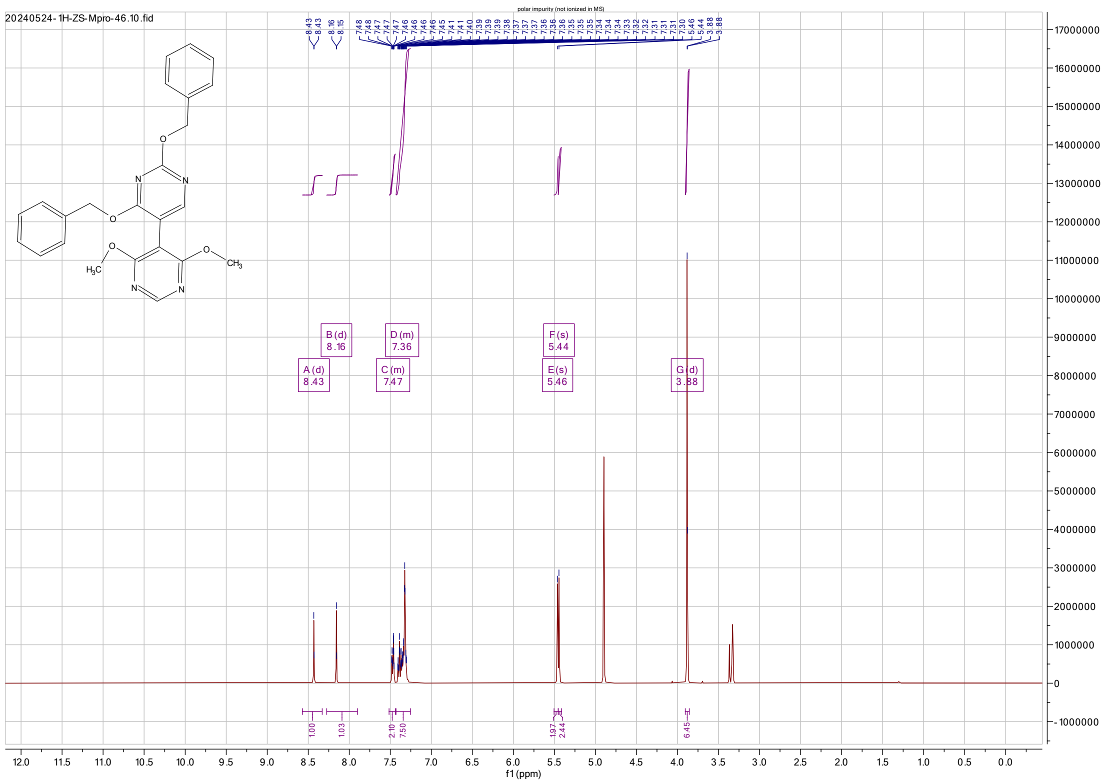

^{1}H NMR spectrum of **S16**

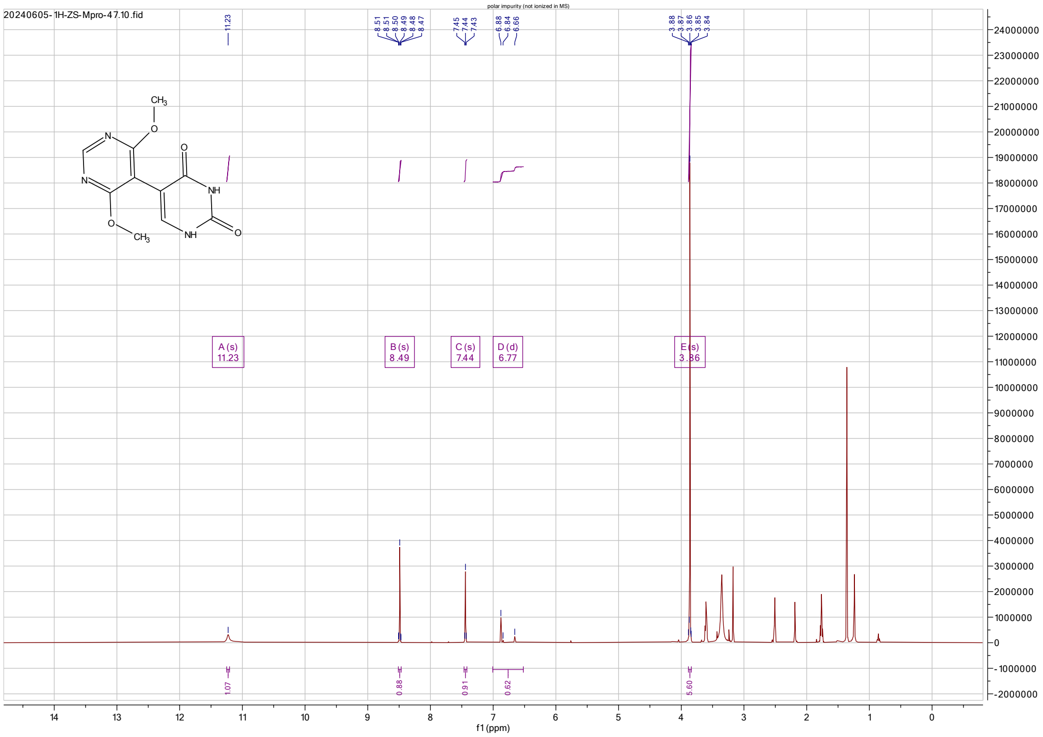

^{1}H NMR spectrum of **S17**

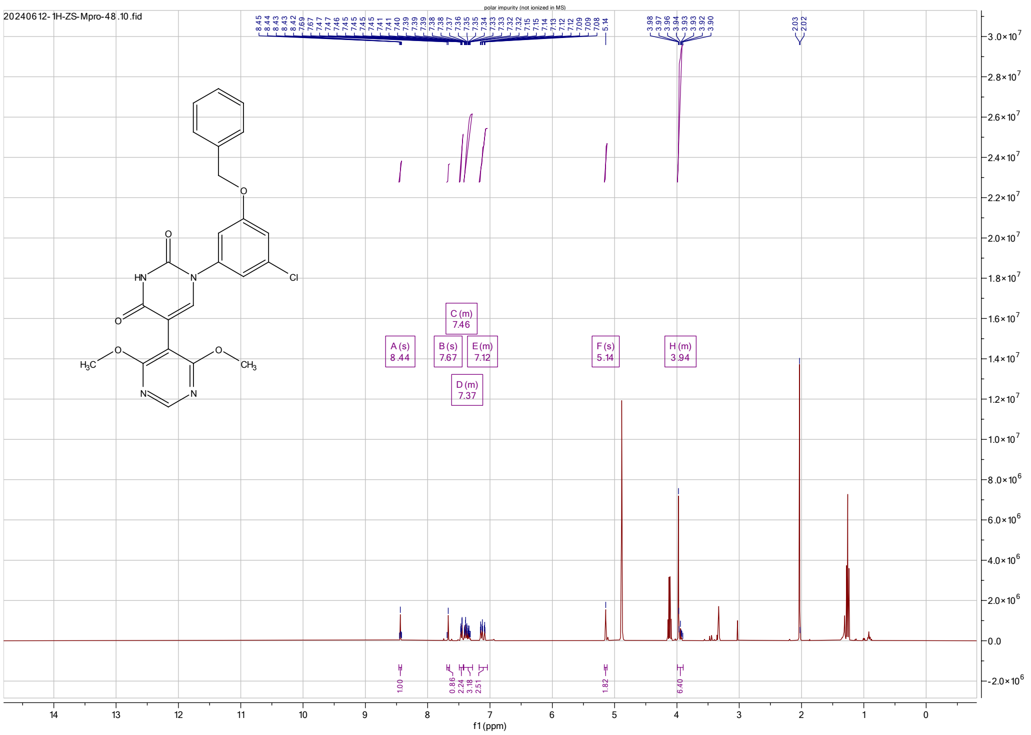

^{1}H NMR spectrum of **S18**

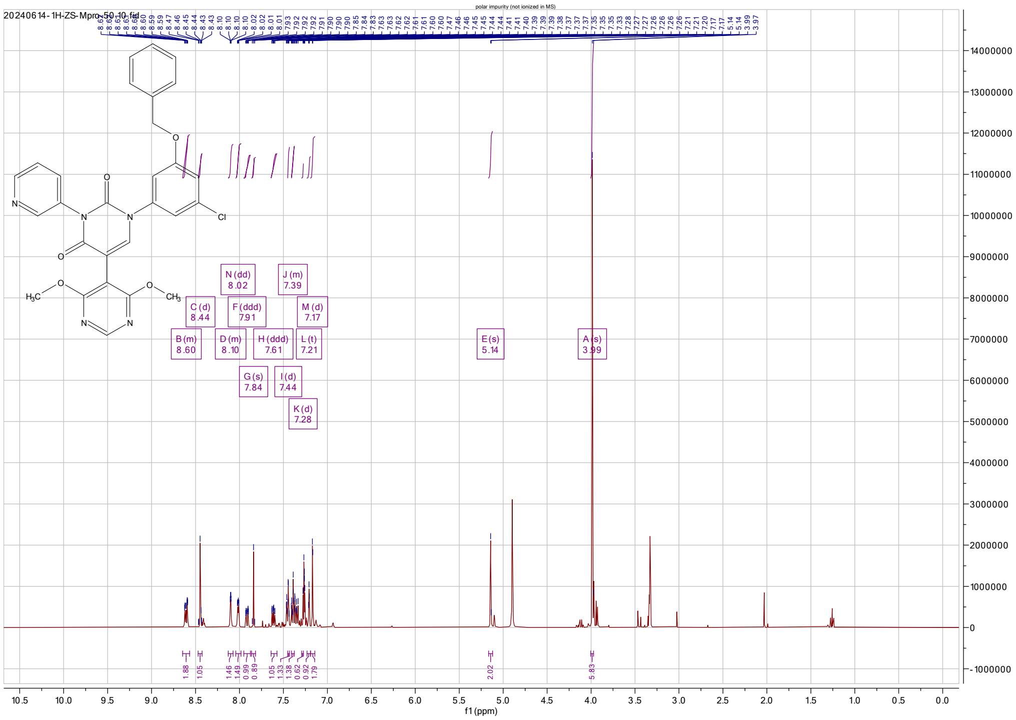

^{1}H NMR spectrum of **S19**

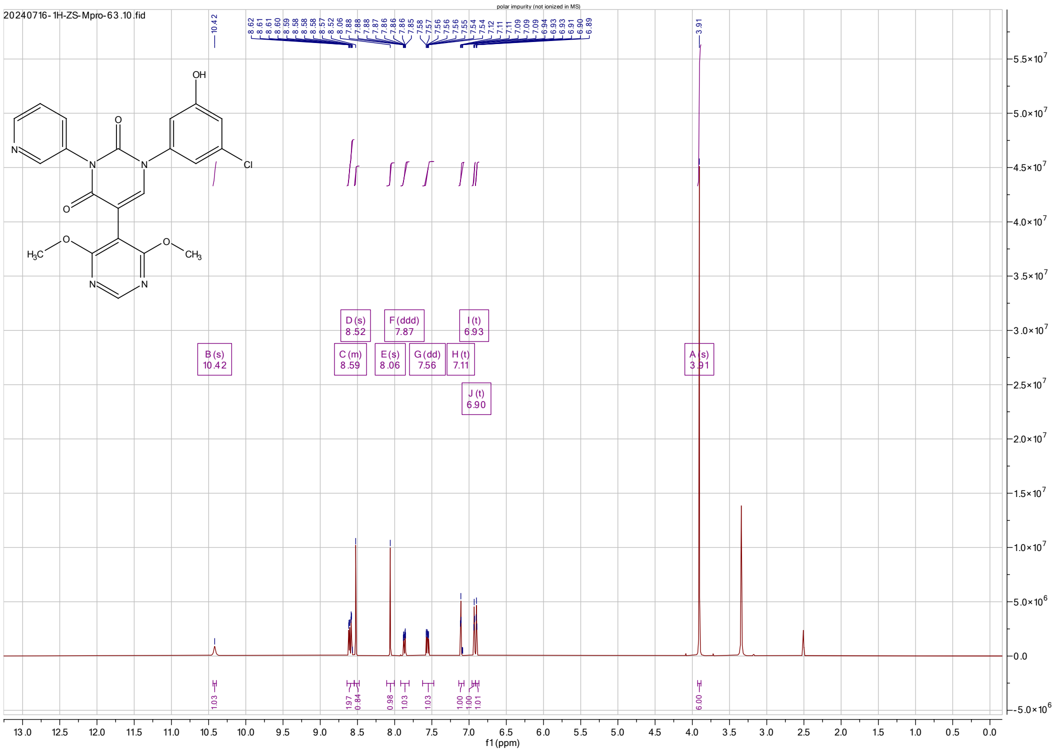

^{1}H NMR spectrum of **S20**

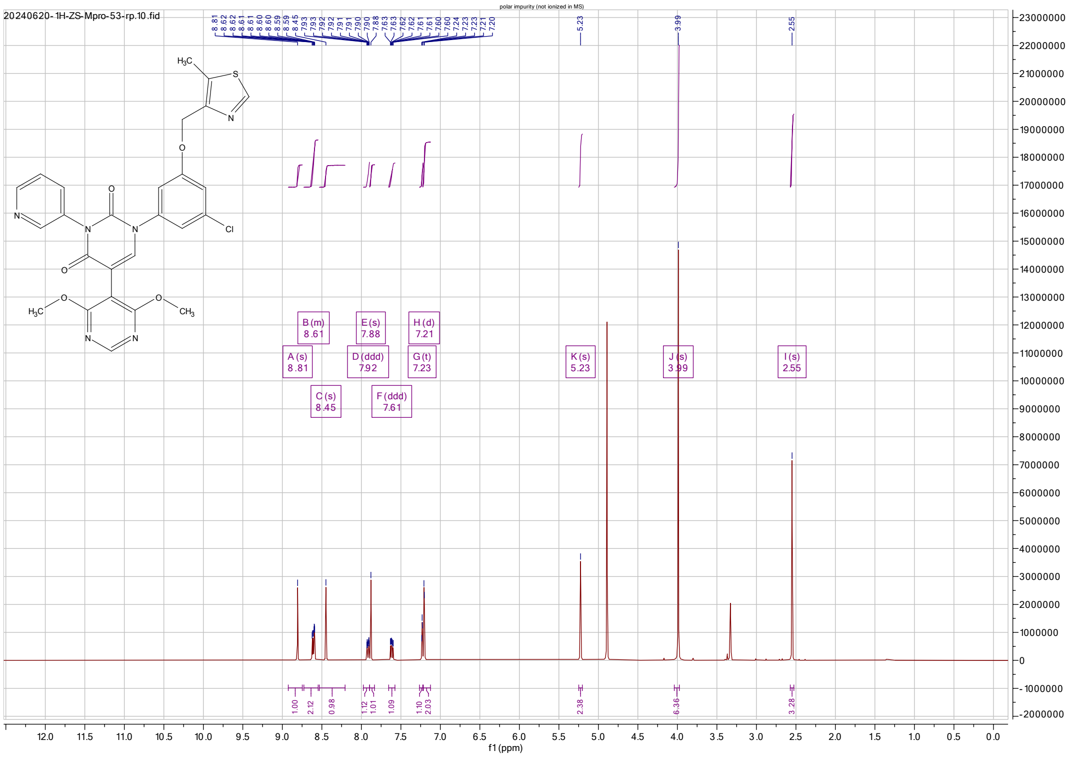

^{1}H and ^{19}F NMR spectrum of **S21**

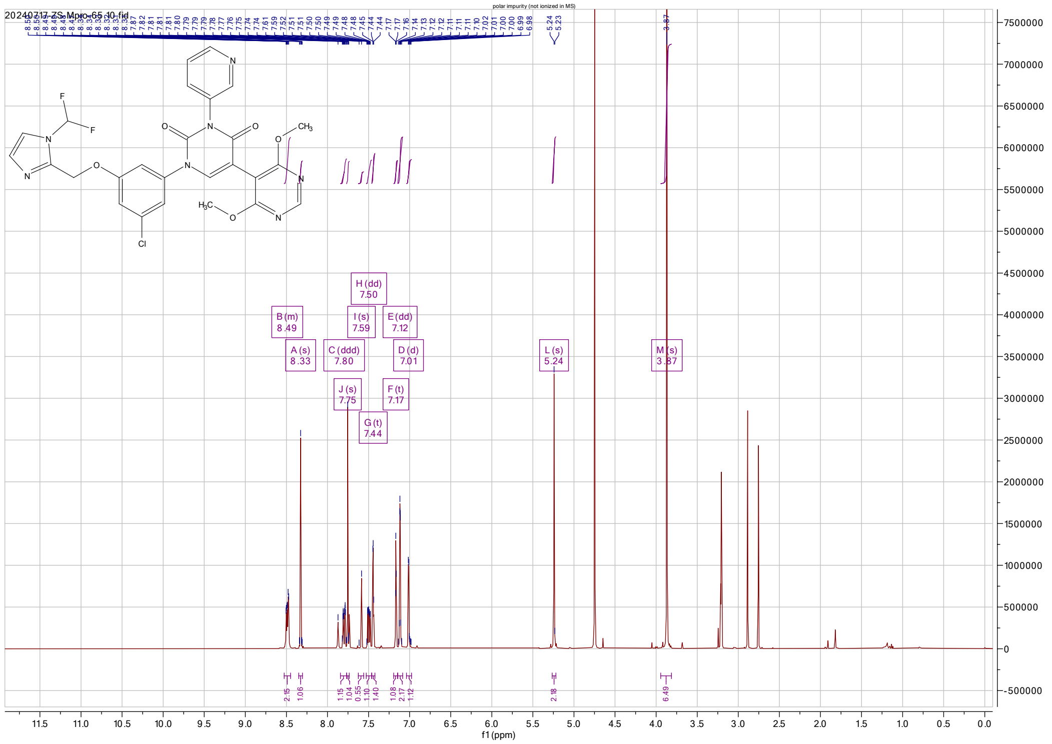

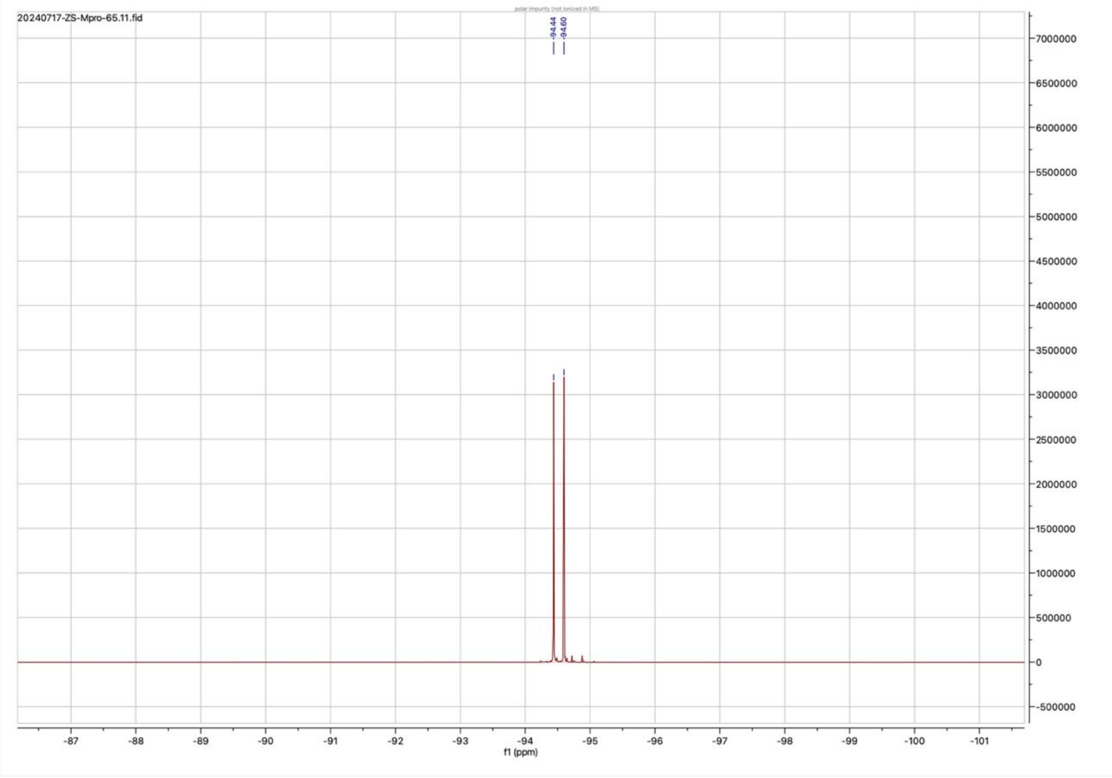

^{1}H NMR spectrum of **S22**

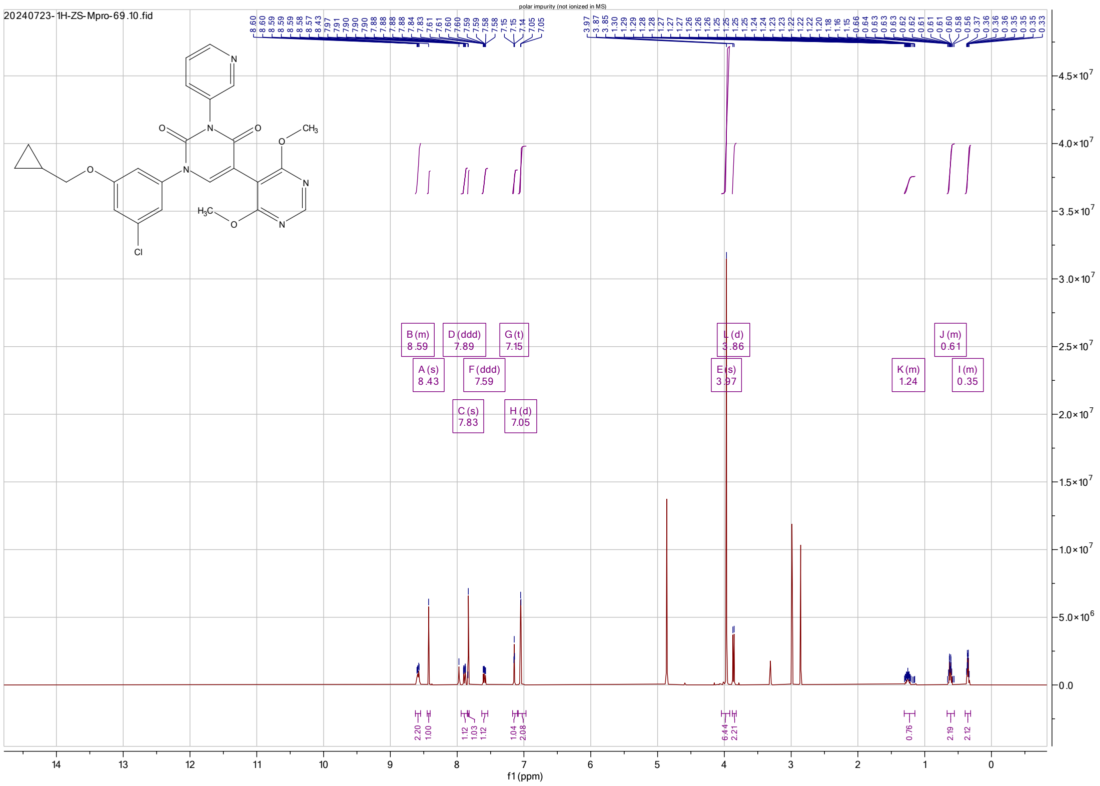

^{1}H and ^{13}C NMR spectrum of **45**

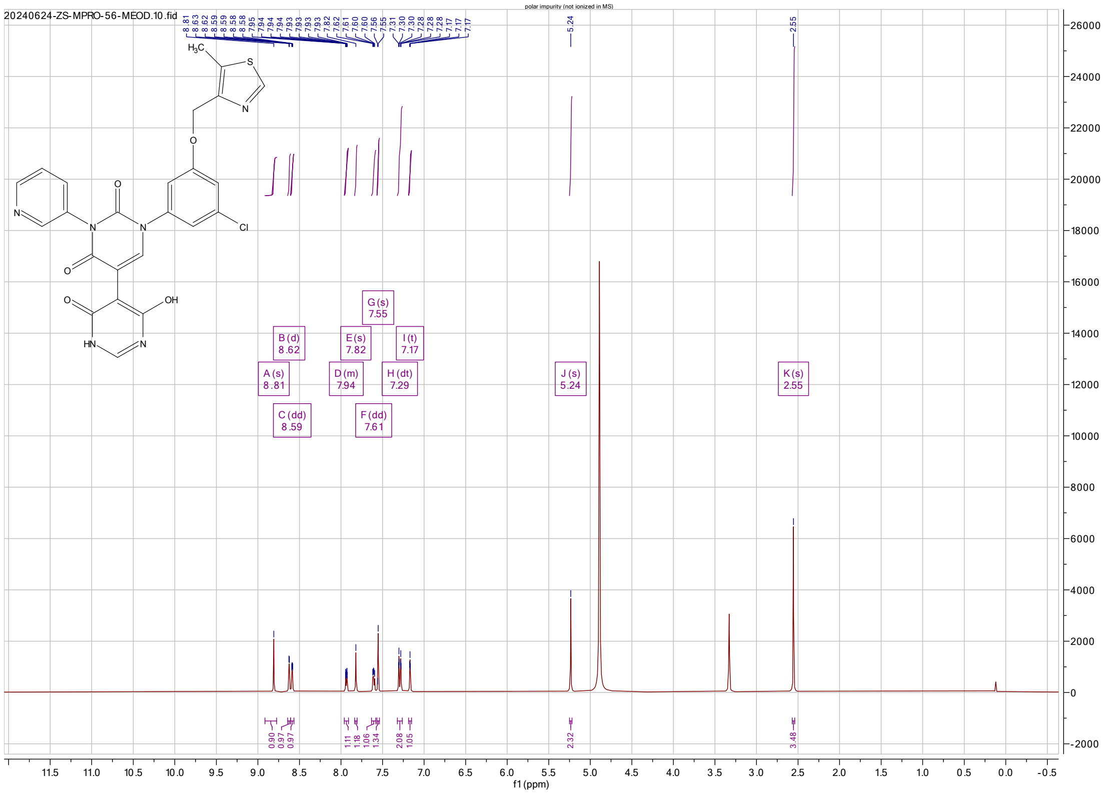

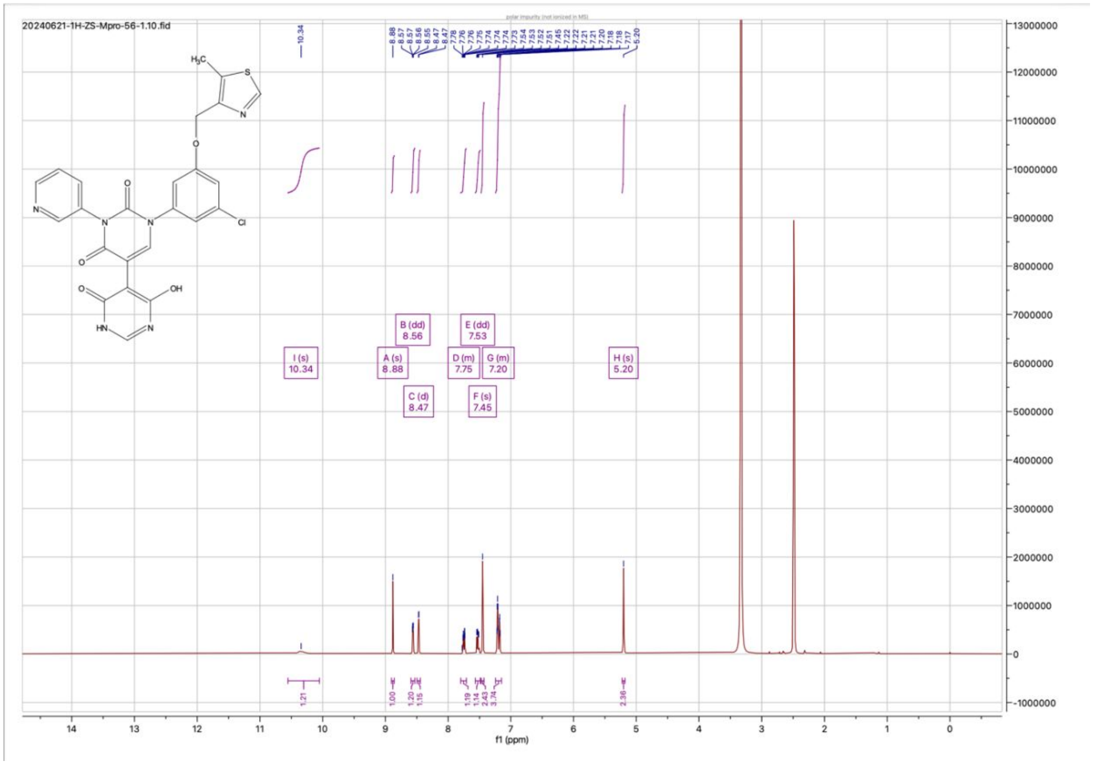

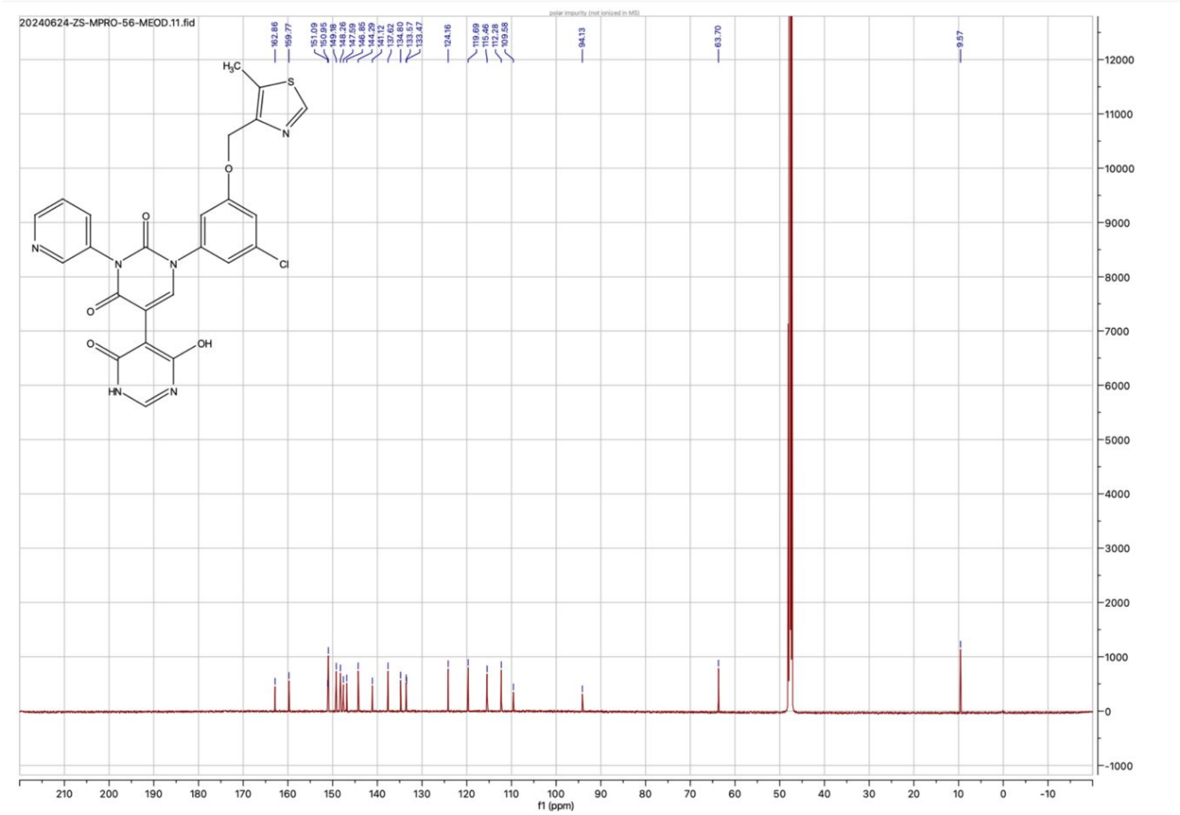

20240624-ZS-MPRO-56-MEOD.11.fid
polar impurity (not ionized in MS)
162.86
159.77
151.09
150.95
149.18
148.26
147.59
146.85
144.29
141.12
137.62
134.80
133.57
133.47
124.16
119.69
115.46
112.28
109.58
94.13
63.70
9.57
f1 (ppm)

^{1}H, ^{19}F and ^{13}C NMR spectrum of **46**

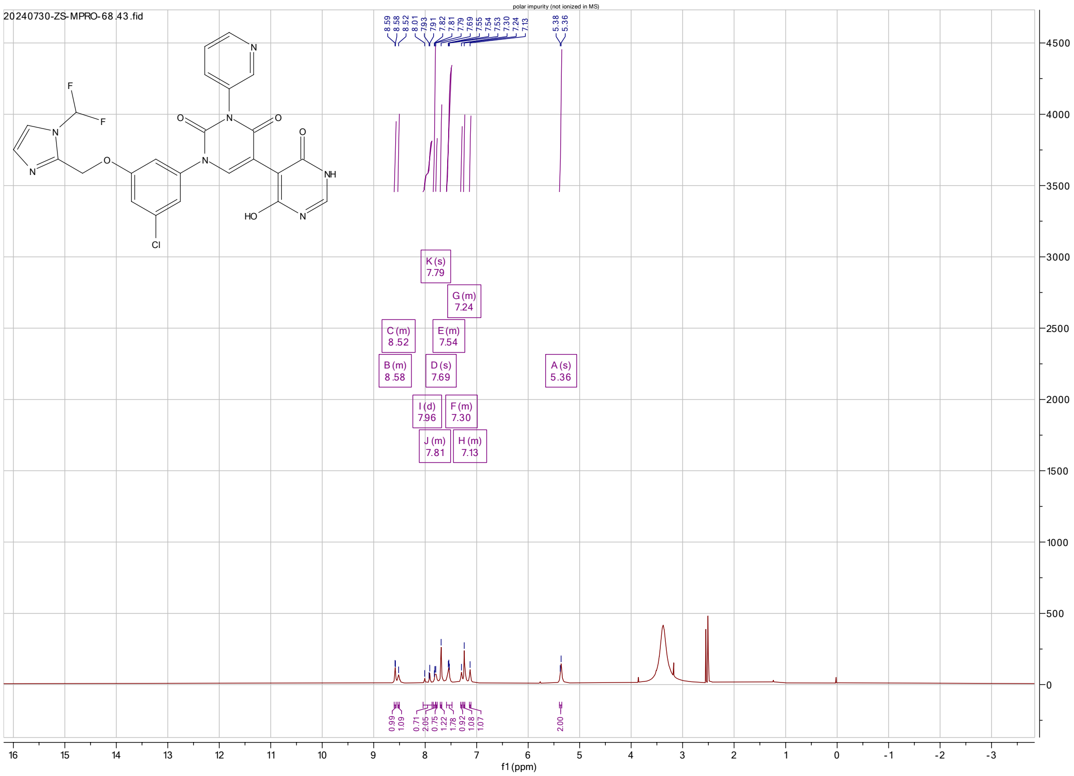

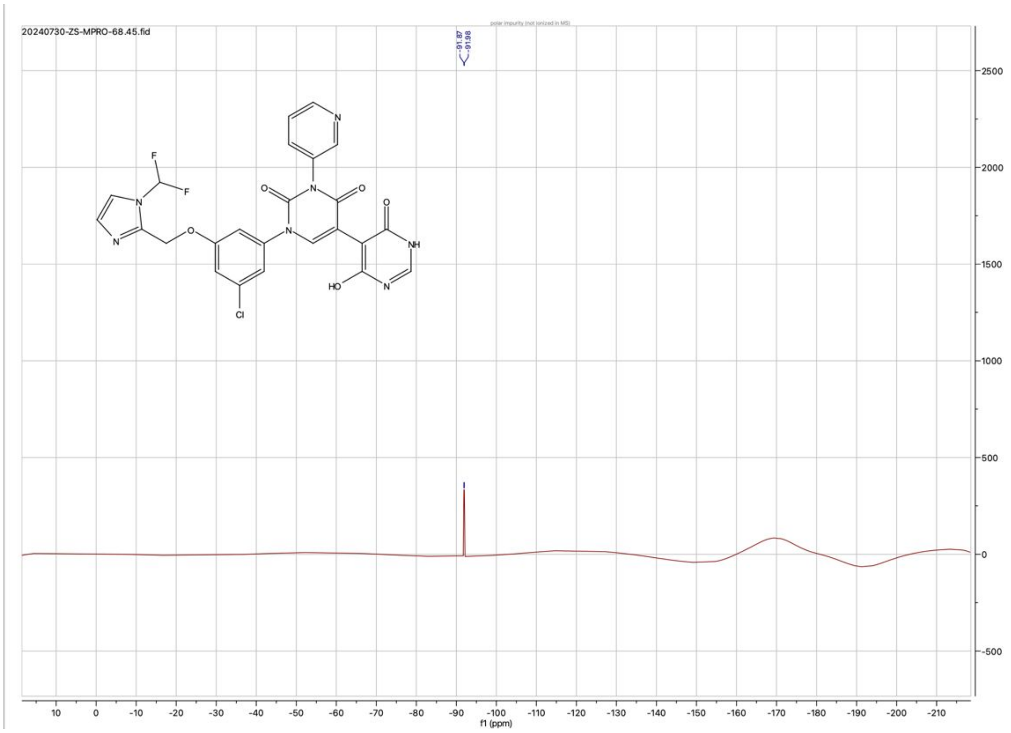

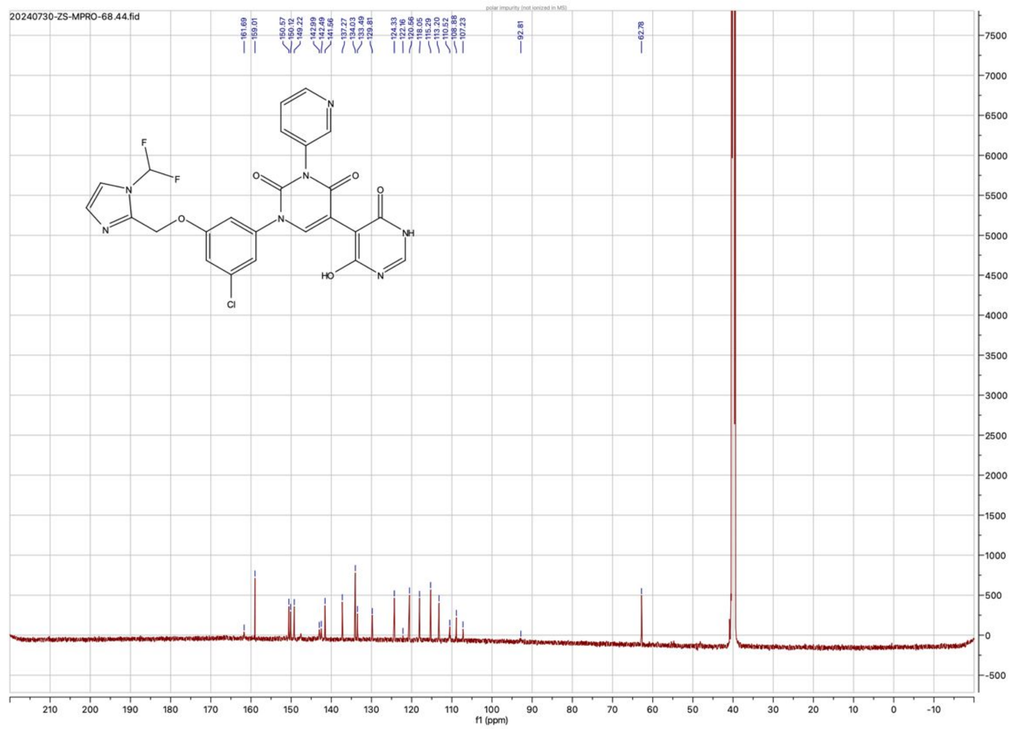

^{1}H and ^{13}C NMR spectrum of **47**

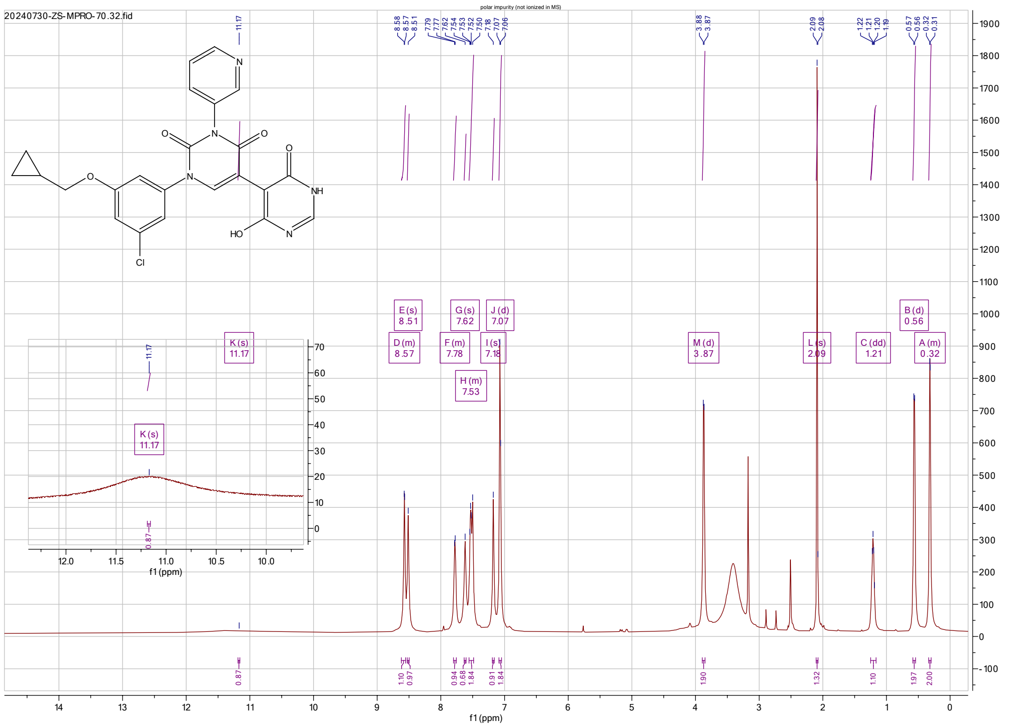

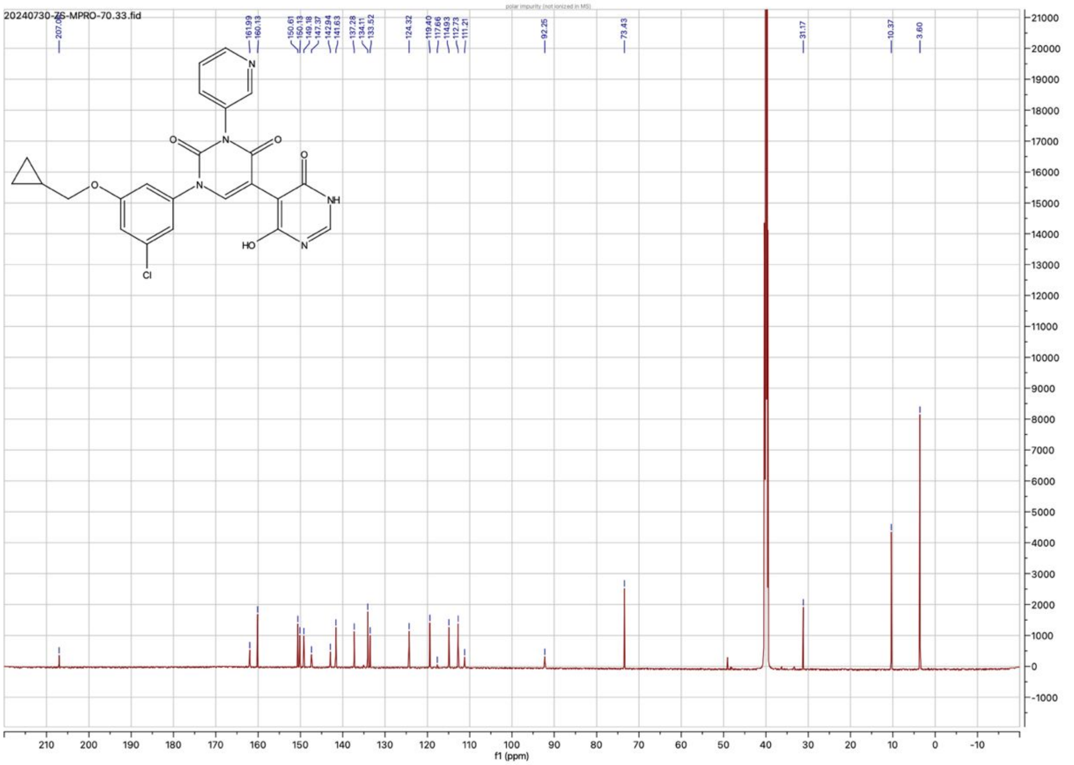

^{1}H and ^{13}C NMR spectrum of **48**

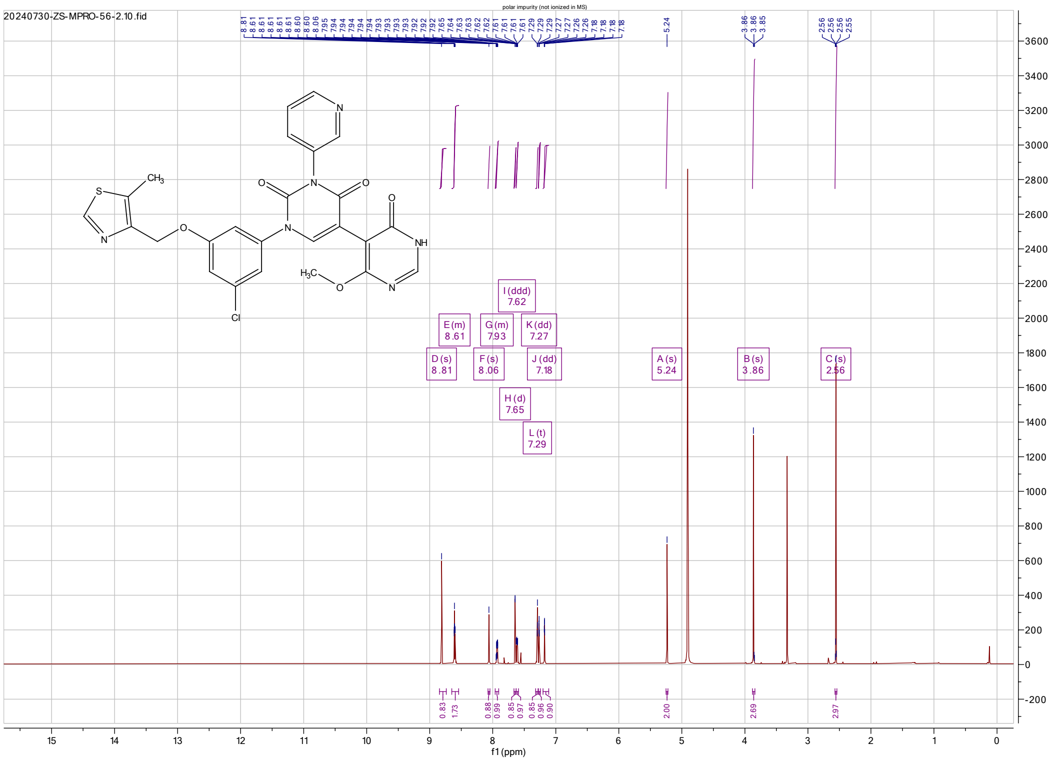

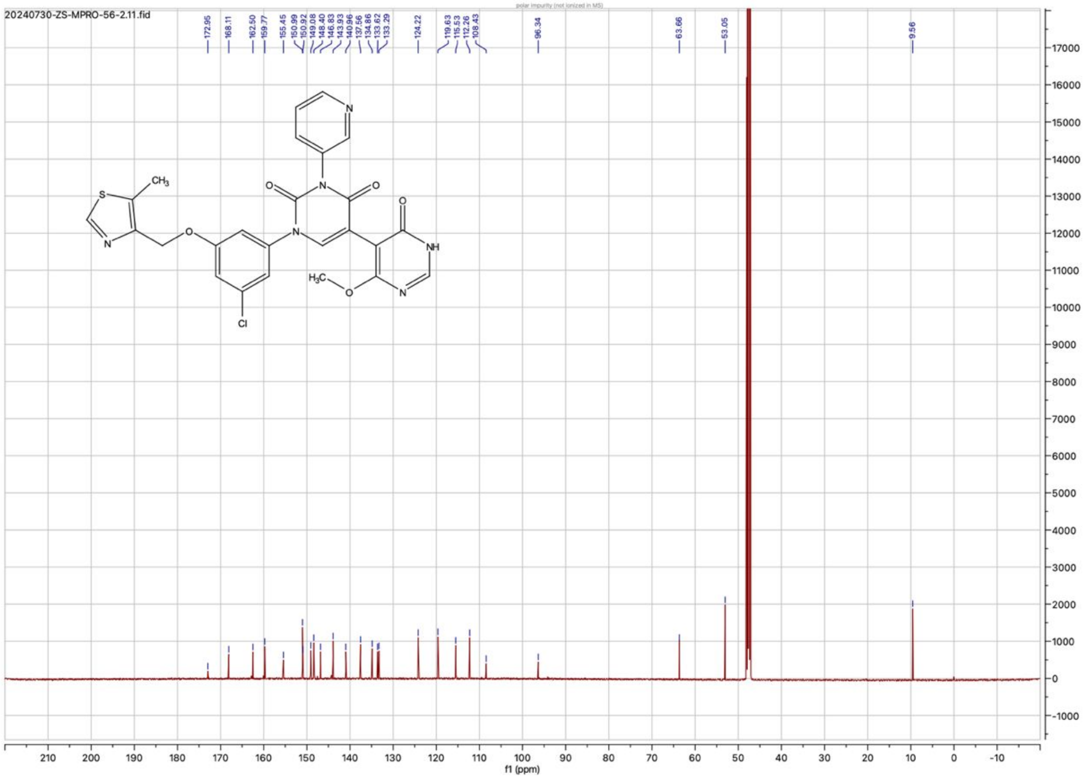

^{1}H and ^{13}C NMR spectrum of **49**

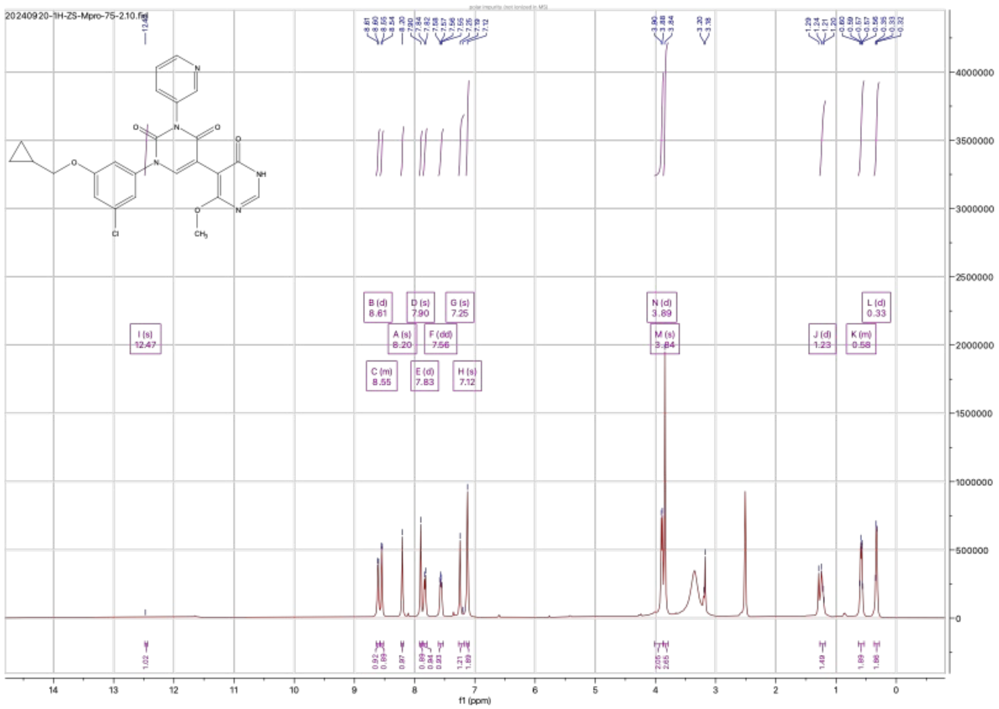

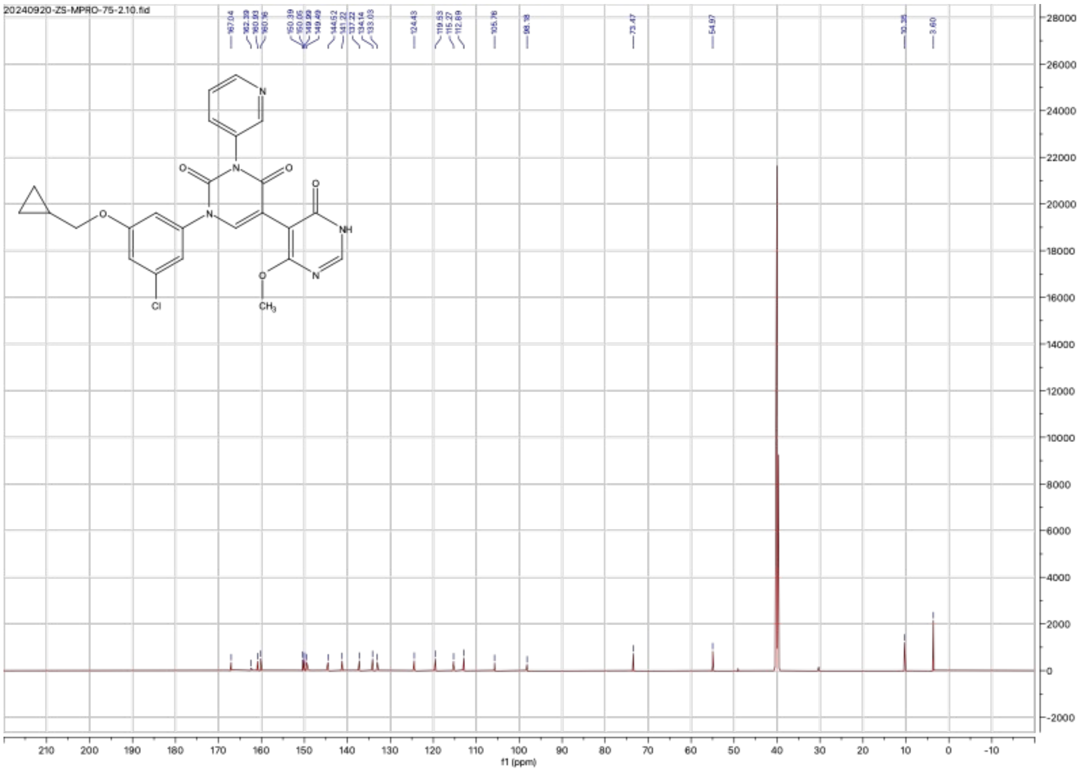

HRMS Spectra

HRMS spectra of compound **4**

APCI Positive

Peak Mass	Display Formula	RDB	Delta [ppm]	Delta [mmu]	Theo. mass	Combined Score	MS Cov. [%]
534.09886	$C_{25}H_{25}O_8N^{35}Cl^{32}S$	13.5	0.87	0.47	534.09839	95.02	98.09
534.09886	$C_{26}H_{21}O_4N_5{}^{35}Cl^{32}S$	18.5	-1.63	-0.87	534.09973	94.97	98.09
534.09886	$C_{27}H_{17}N_9{}^{35}Cl^{32}S$	23.5	-4.14	-2.21	534.10107	94.25	98.09
534.09886	$C_{13}H_{29}O_{15}N_3{}^{35}Cl^{32}S$	0.5	-2.59	-1.38	534.10024	74.36	77.2
534.09886	$C_9H_{25}O_{13}N_9{}^{35}Cl^{32}S$	1.5	2.44	1.3	534.09756	73.66	76.62

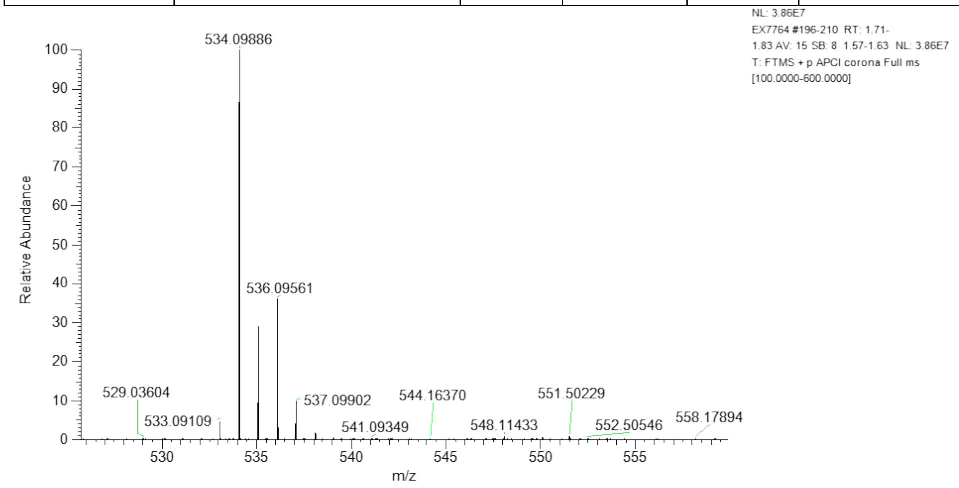

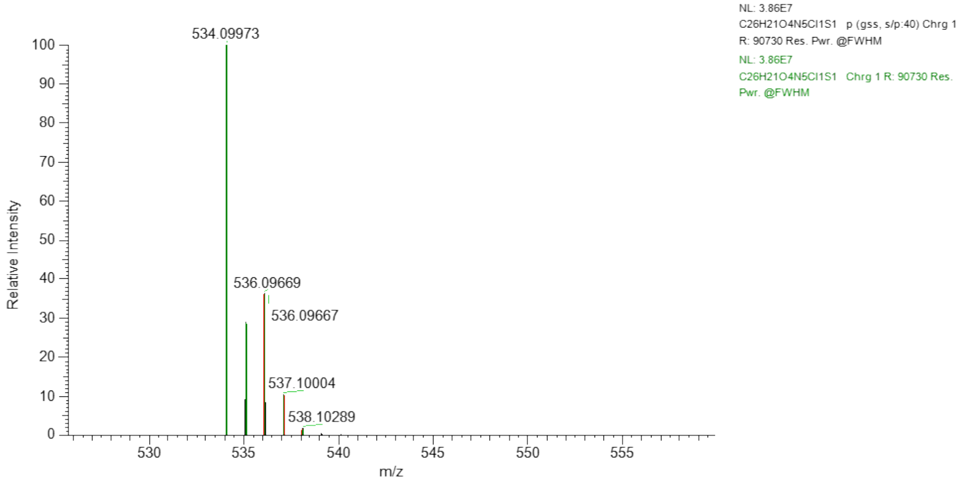

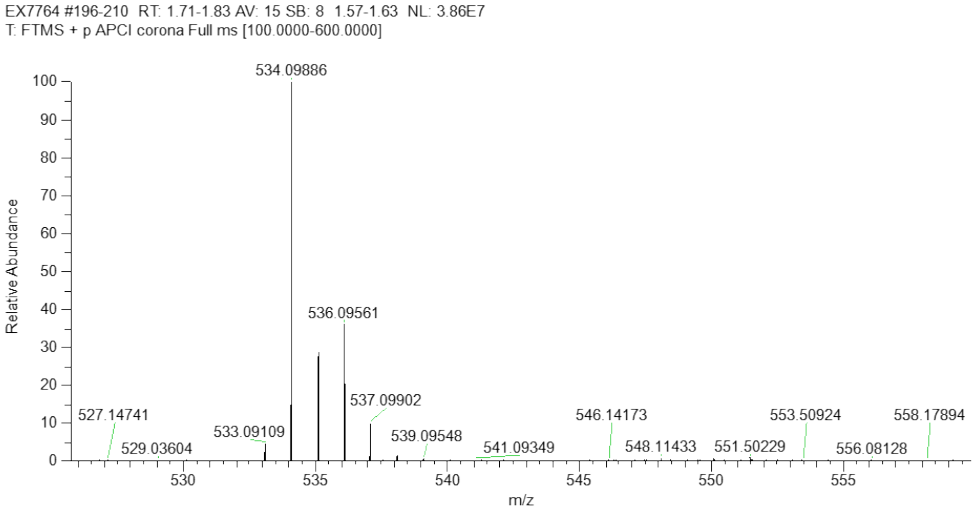

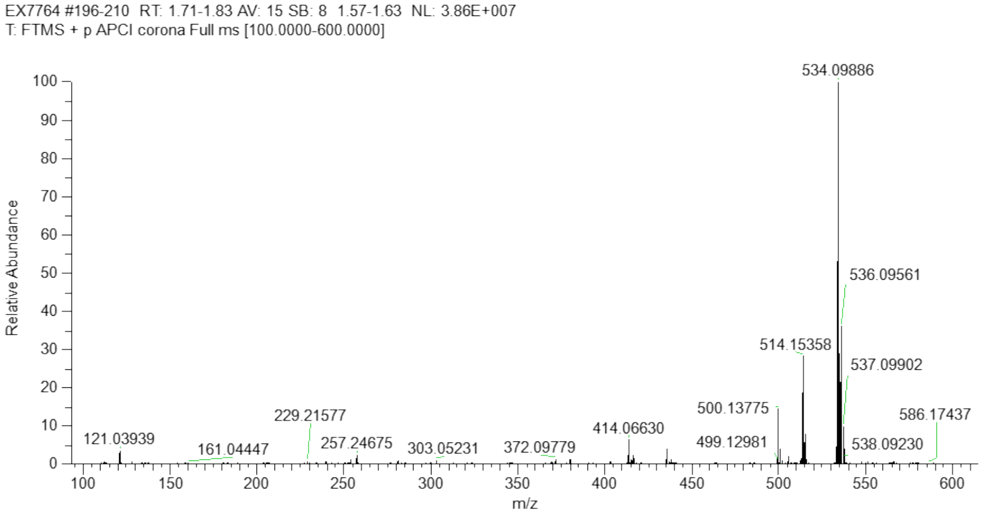

HRMS spectra of compound **18**

APCI Positive

Peak Mass	Display Formula	RDB	Delta [ppm]	Delta [mmu]	Theo. mass	Combined Score	MS Cov. [%]
548.11537	$C_{27}H_{23}O_{4}N_{5}{}^{35}Cl^{32}S$	18.5	-0.01	-0.01	548.11538	83.9	87.25
548.11537	$C_{26}H_{27}O_{8}N^{35}Cl^{32}S$	13.5	2.43	1.33	548.11404	83.76	87.25
548.11537	$C_{28}H_{19}N_{9}{}^{35}Cl^{32}S$	23.5	-2.45	-1.35	548.11672	83.69	87.25
548.11537	$C_{15}H_{27}O_{11}N_{7}{}^{35}Cl^{32}S$	5.5	-3.38	-1.86	548.11723	65.91	68.82
548.11537	$C_{14}H_{31}O_{15}N_{3}{}^{35}Cl^{32}S$	0.5	-0.94	-0.52	548.11589	65.39	68.34
548.11537	$C_{10}H_{27}O_{13}N_{9}{}^{35}Cl^{32}S$	1.5	3.96	2.16	548.11321	65.21	68.34

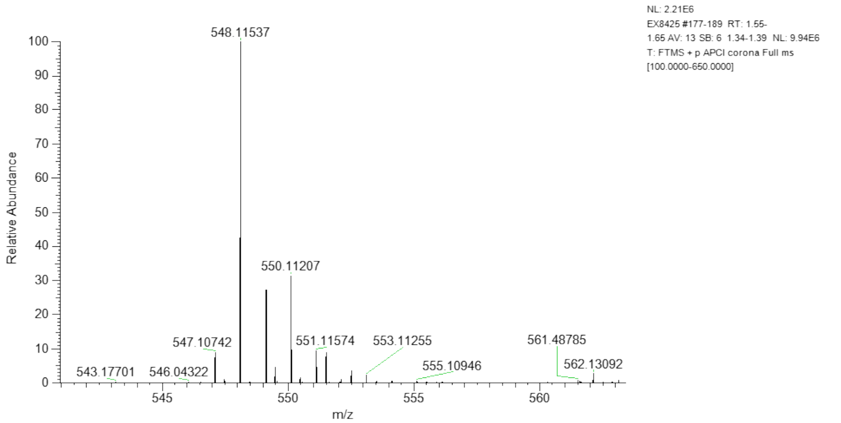

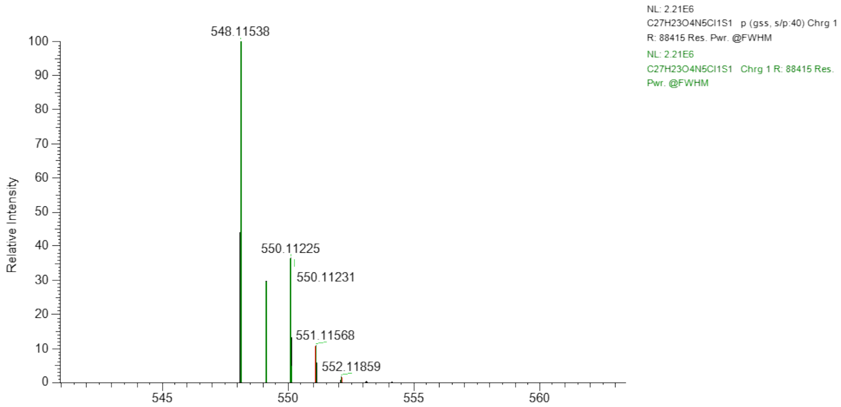

EX8425 #177-189 RT: 1.55-1.65 AV: 13 SB: 6 1.34-1.39 NL: 9.94E+006
T: FTMS + p APCI corona Full ms [100.0000-650.0000]

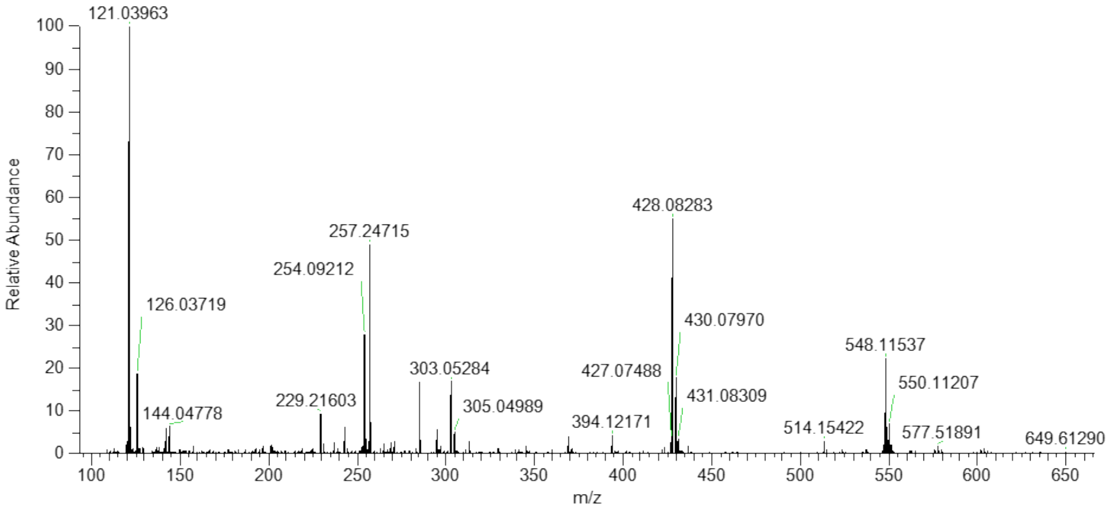

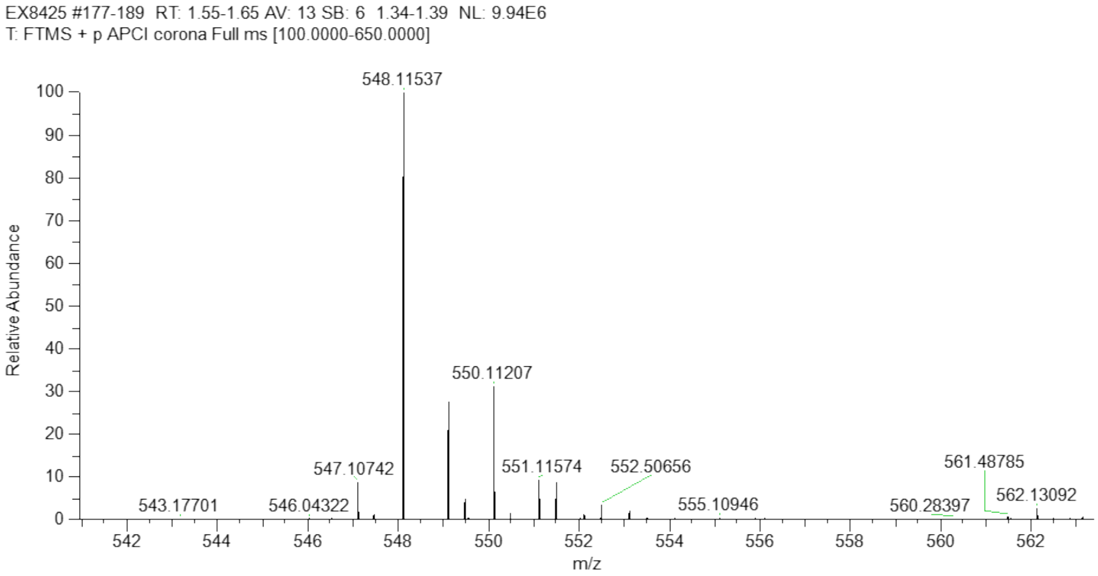

HRMS spectra of compound **19**

APCI Positive

Peak Mass	Display Formula	RDB	Delta [ppm]	Delta [mmu]	Theo. mass	Combined Score	MS Cov. [%]
553.11976	$C_{26}H_{20}O_4N_6{}^{35}ClF_2$	18.5	0.08	0.05	553.11971	91.02	94.81
553.11976	$C_{25}H_{24}O_8N_2{}^{35}ClF_2$	13.5	2.5	1.38	553.11838	90.87	94.81
553.11976	$C_{27}H_{16}N_{10}{}^{35}ClF_2$	23.5	-2.34	-1.29	553.12105	90.84	94.81
553.11976	$C_{14}H_{24}O_{11}N_8{}^{35}ClF_2$	5.5	-3.27	-1.8	553.12156	77.09	80.15
553.11976	$C_{13}H_{28}O_{15}N_4{}^{35}ClF_2$	0.5	-0.85	-0.47	553.12023	76.35	79.62
553.11976	$C_9H_{24}O_{13}N_{10}{}^{35}ClF_2$	1.5	4.01	2.22	553.11754	76.18	79.62
553.11976	$C_{30}H_{24}O_6{}^{35}ClF_2$	17.5	-4.78	-2.64	553.1224	58.71	61.75

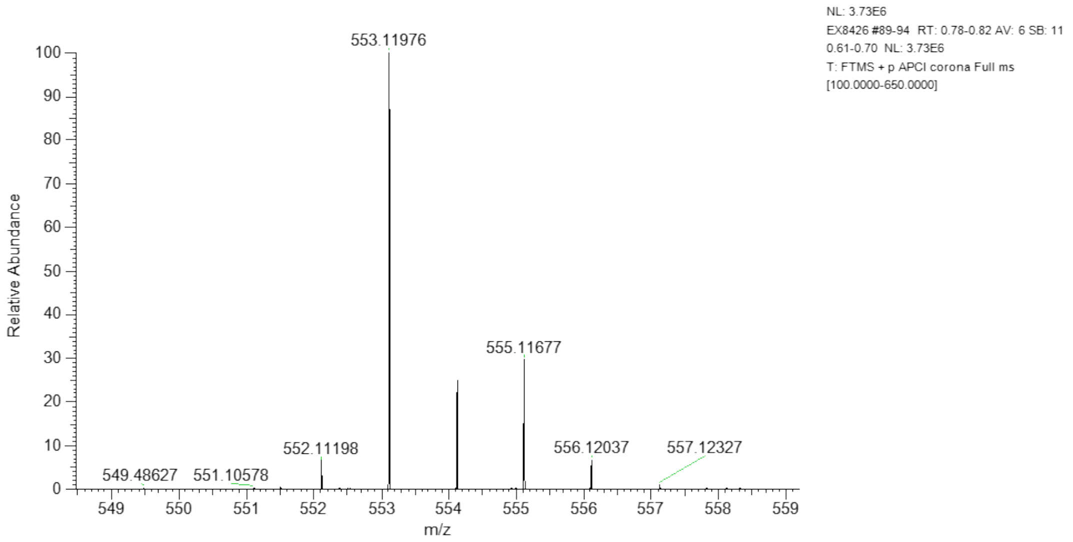
NL: 3.73E6
EX8426 #89-94 RT: 0.78-0.82 AV: 6 SB: 11
0.61-0.70 NL: 3.73E6
T: FTMS + p APCI corona Full ms
[100.0000-650.0000]
553.11976
555.11677
552.11198
556.12037
557.12327
549.48627
551.10578
Relative Abundance
m/z

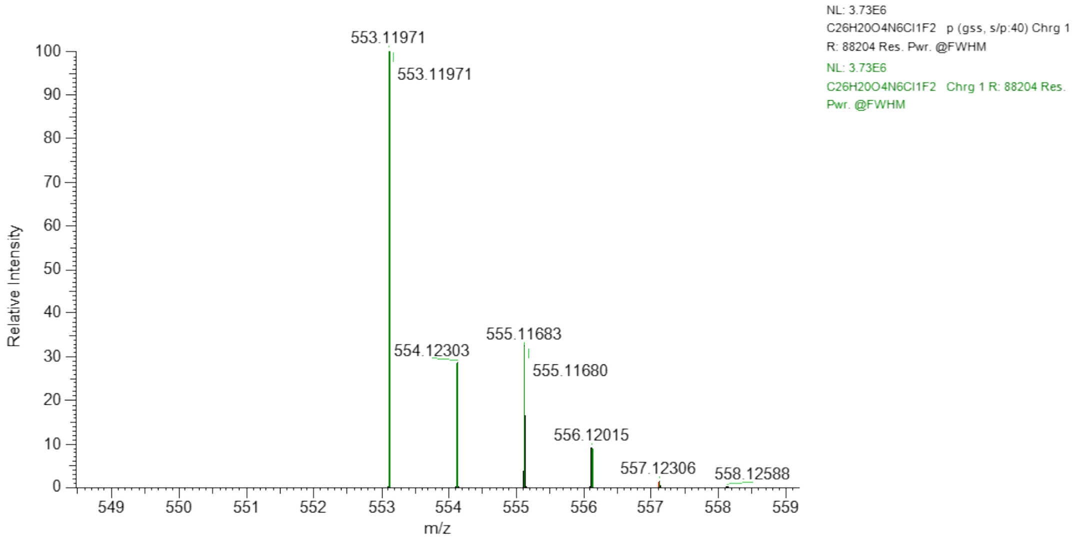
NL: 3.73E6
C26H20O4N6Cl1F2 p (gss, s/p:40) Chrg 1
R: 88204 Res. Pwr. @FWHM
NL: 3.73E6
C26H20O4N6Cl1F2 Chrg 1 R: 88204 Res. Pwr. @FWHM
553.11971
553.11971
554.12303
555.11683
555.11680
556.12015
557.12306
558.12588
Relative Intensity
m/z

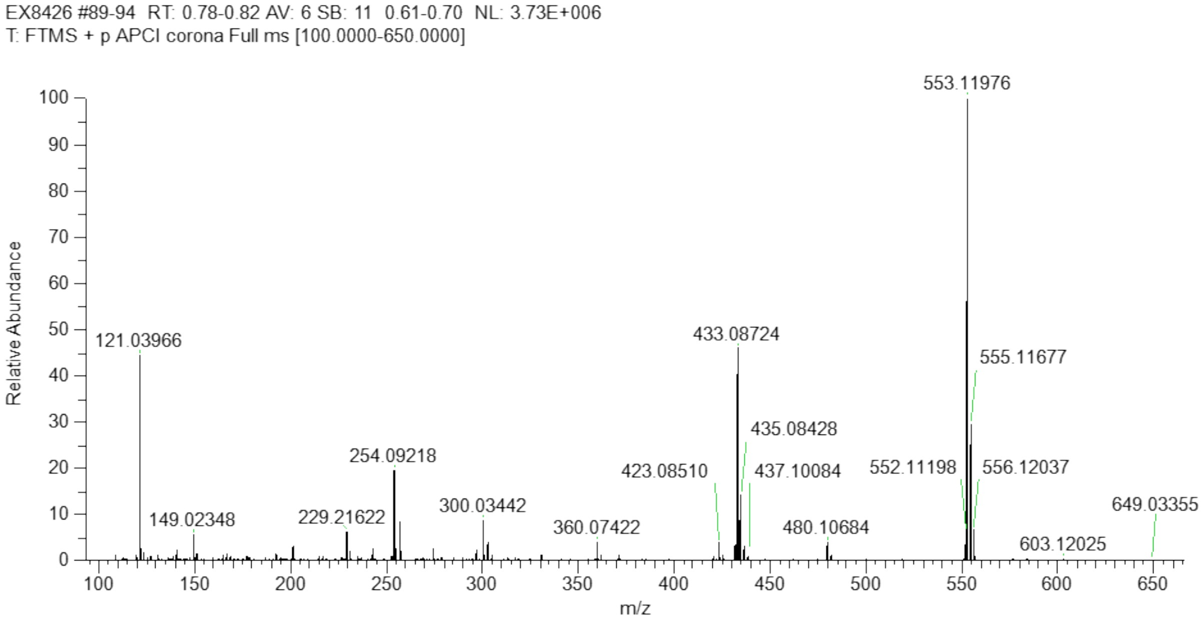
EX8426 #89-94 RT: 0.78-0.82 AV: 6 SB: 11 0.61-0.70 NL: 3.73E+006
T: FTMS + p APCI corona Full ms [100.0000-650.0000]
553.11976
121.03966
433.08724
555.11677
435.08428
254.09218
423.08510
437.10084
552.11198
556.12037
149.02348
229.21622
300.03442
360.07422
480.10684
649.03355
603.12025
Relative Abundance
m/z

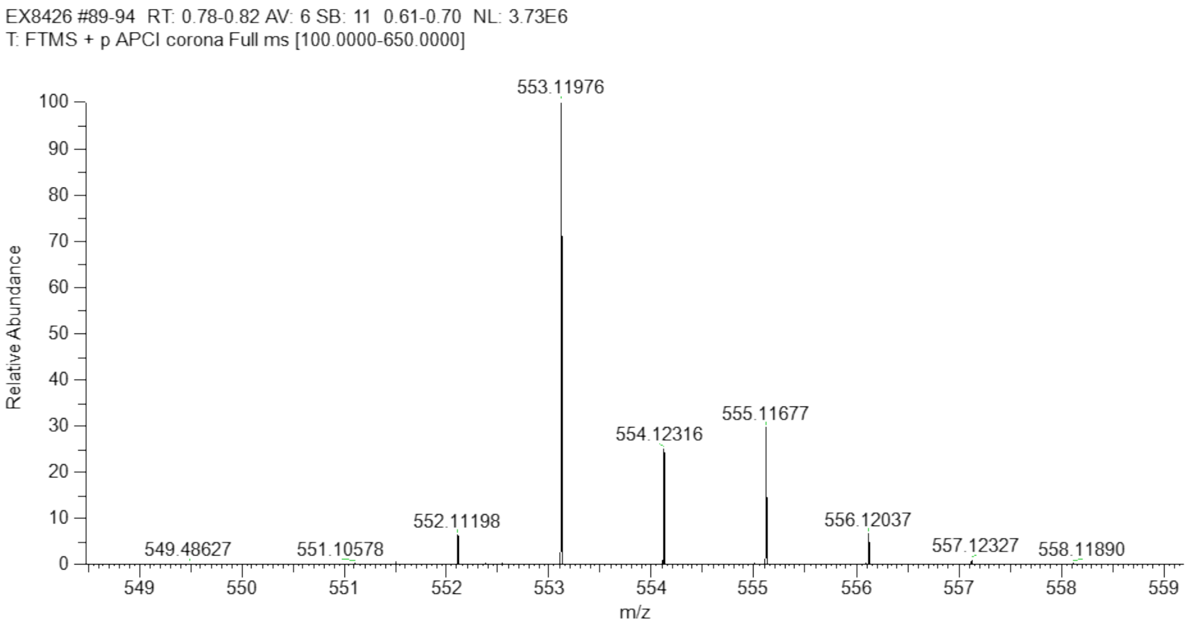

HRMS spectra of compound **20**

APCI Positive

Peak Mass	Display Formula	RDB	Delta [ppm]	Delta [mmu]	Theo. mass	Combined Score	MS Cov. [%]
477.13233	$C_{25}H_{22}O_4N_4{}^{35}Cl$	16.5	-0.18	-0.08	477.13241	93.84	97.19
477.13233	$C_{26}H_{18}N_8{}^{35}Cl$	21.5	-2.98	-1.42	477.13375	93.49	97.19
477.13233	$C_{24}H_{26}O_8{}^{35}Cl$	11.5	2.63	1.26	477.13107	93	96.63
477.13233	$C_{13}H_{26}O_{11}N_6{}^{35}Cl$	3.5	-4.05	-1.93	477.13426	76.77	79.24
477.13233	$C_8H_{26}O_{13}N_8{}^{35}Cl$	-0.5	4.38	2.09	477.13024	73.65	77.04

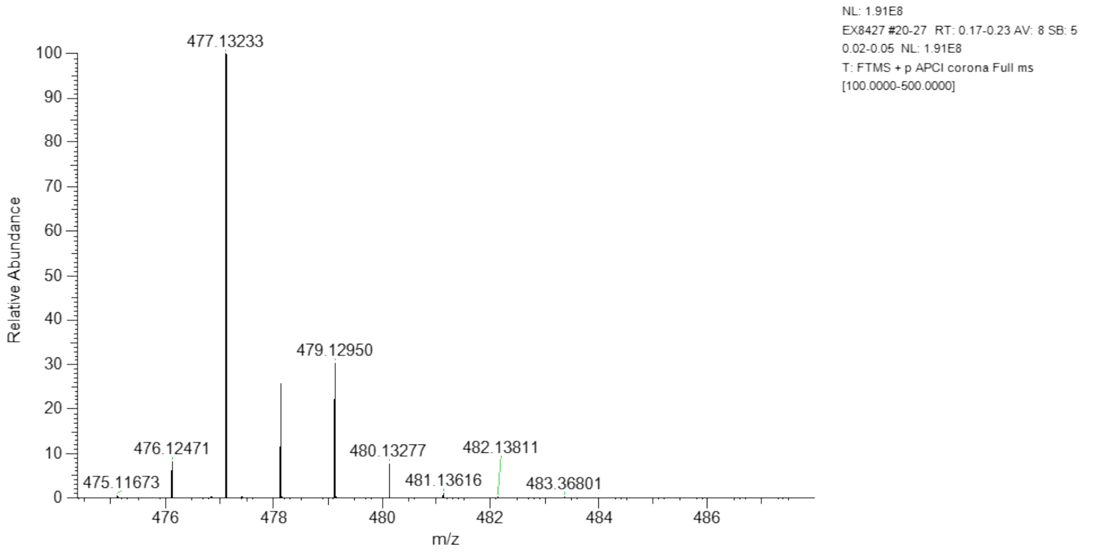

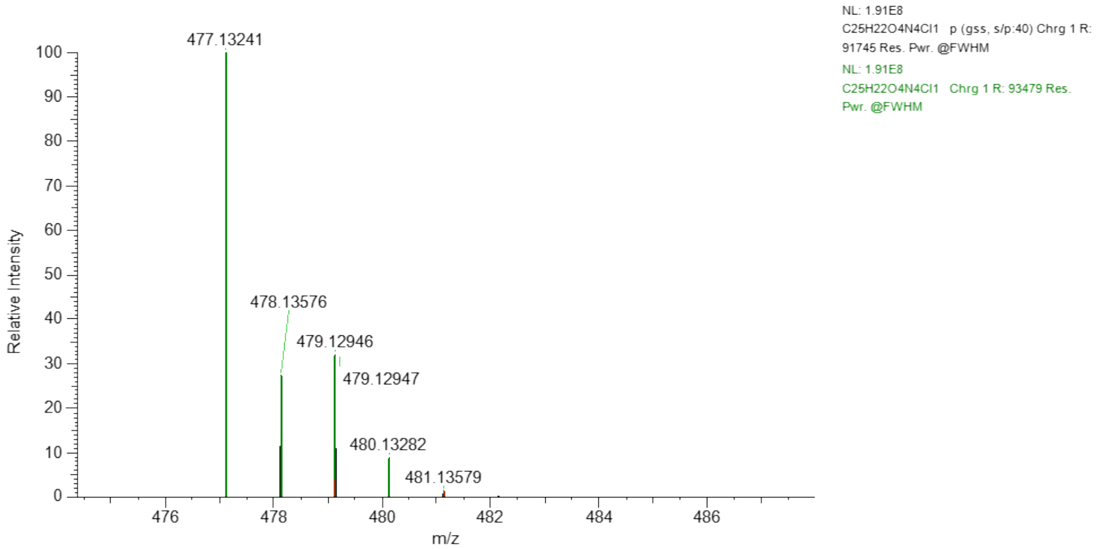

EX8427 #20-27 RT: 0.17-0.23 AV: 8 SB: 5 0.02-0.05 NL: 1.91E+008
T: FTMS + p APCI corona Full ms [100.0000-500.0000]

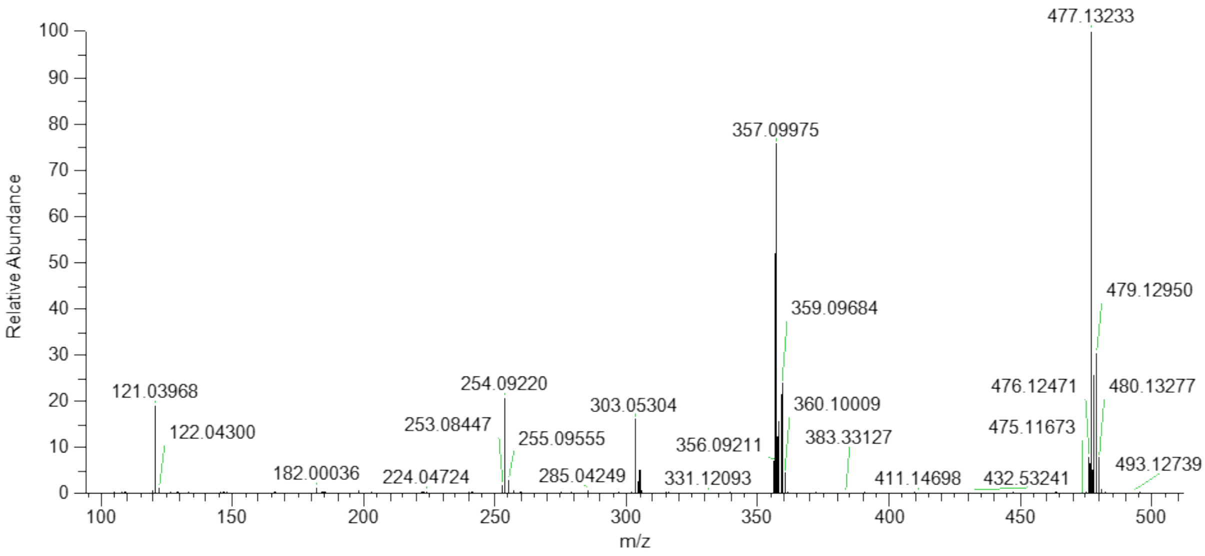

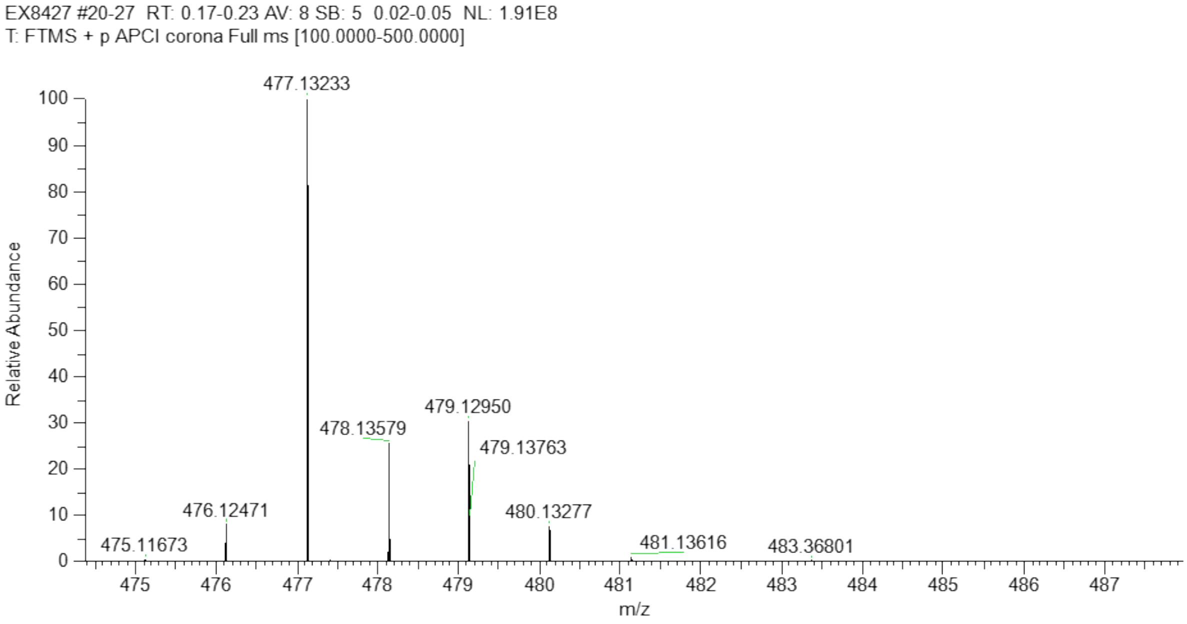

HRMS spectra of compound **21**

APCI Positive

Peak Mass	Display Formula	RDB	Delta [ppm]	Delta [mmu]	Theo. mass	Combined Score	MS Cov. [%]
514.15438	$C_{27}H_{24}O_4N_5{}^{32}S$	18.5	0.05	0.03	514.15435	95.1	98.99
514.15438	$C_{28}H_{20}N_9{}^{32}S$	23.5	-2.55	-1.31	514.15569	89.67	93.94
514.15438	$C_{26}H_{28}O_8N^{32}S$	13.5	2.65	1.37	514.15301	89.65	93.94
514.15438	$C_{14}H_{32}O_{15}N_3{}^{32}S$	0.5	-0.95	-0.48	514.15486	77.02	79.92
514.15438	$C_{15}H_{28}O_{11}N_7{}^{32}S$	5.5	-3.55	-1.82	514.1562	75.29	77.69
514.15438	$C_{10}H_{28}O_{13}N_9{}^{32}S$	1.5	4.27	2.2	514.15218	70.51	74.17

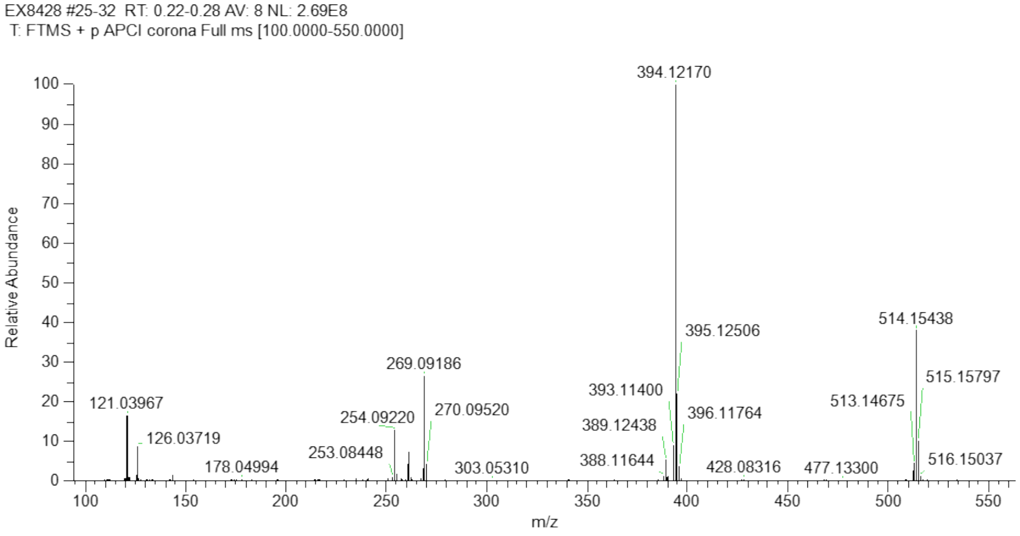

EX8428 #25-32 RT: 0.22-0.28 AV: 8 NL: 1.02E+008
T: FTMS + p APCI corona Full ms [100.0000-550.0000]

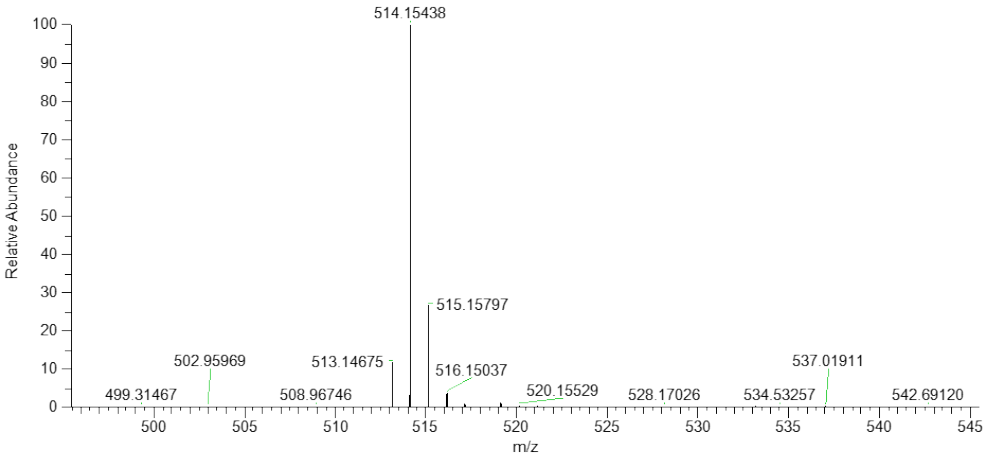

HRMS spectra of compound **22**

APCI Positive

Peak Mass	Display Formula	RDB	Delta [ppm]	Delta [mmu]	Theo. mass	Combined Score	MS Cov. [%]
519.15867	$C_{26}H_{21}O_4N_6F_2$	18.5	-0.04	-0.02	519.15869	96.38	99.34
519.15867	$C_{27}H_{17}N_{10}F_2$	23.5	-2.61	-1.35	519.16002	96.09	99.34
519.15867	$C_{25}H_{25}O_8N_2F_2$	13.5	2.54	1.32	519.15735	92.91	96.71
519.15867	$C_{14}H_{25}O_{11}N_8F_2$	5.5	-3.6	-1.87	519.16054	76.67	78.94
519.15867	$C_{13}H_{29}O_{15}N_4F_2$	0.5	-1.02	-0.53	519.1592	75.89	78.72
519.15867	$C_9H_{25}O_{13}N_{10}F_2$	1.5	4.15	2.16	519.15651	72.54	76.09

EX8429 #16-21 RT: 0.14-0.18 AV: 6 SB: 5 0.02-0.05 NL: 1.81E8
T: FTMS + p APCI corona Full ms [100.0000-550.0000]

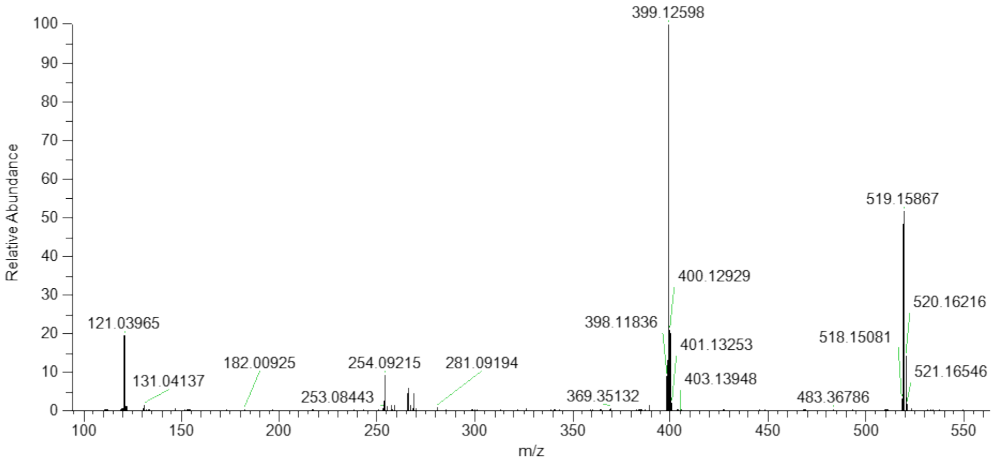

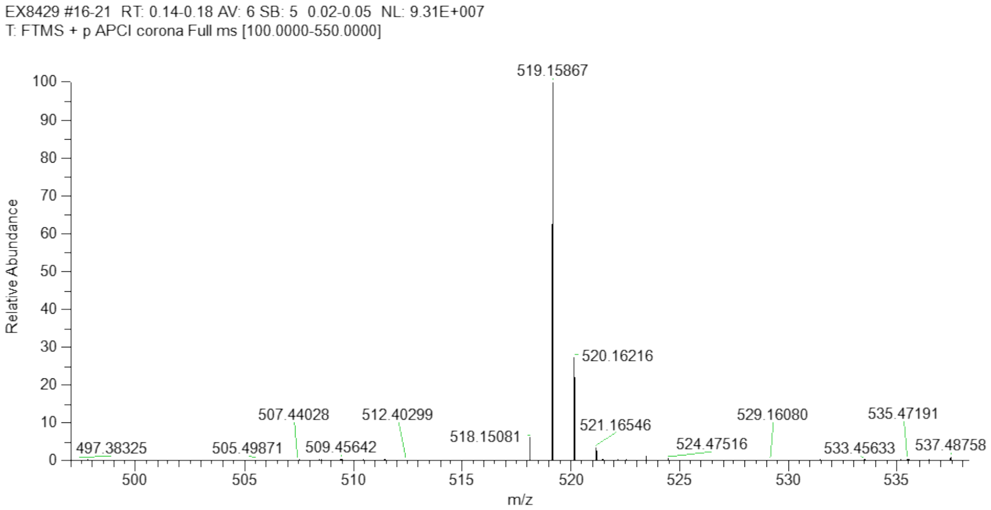

HRMS spectra of compound **23**

APCI Positive

Peak Mass	Display Formula	RDB	Delta [ppm]	Delta [mmu]	Theo. mass	Combined Score	MS Cov. [%]
443.17104	$C_{25}H_{23}O_4N_4$	16.5	-0.78	-0.34	443.17138	96.85	99.79
443.17104	$C_{26}H_{19}N_8$	21.5	-3.79	-1.68	443.17272	95.31	99.79
443.17104	$C_{24}H_{27}O_8$	11.5	2.24	1	443.17004	93.59	97.35
443.17104	$C_{13}H_{27}O_{11}N_6$	3.5	-4.95	-2.19	443.17323	76.86	79.85
443.17104	$C_8H_{27}O_{13}N_8$	-0.5	4.13	1.83	443.16921	73.52	77.22

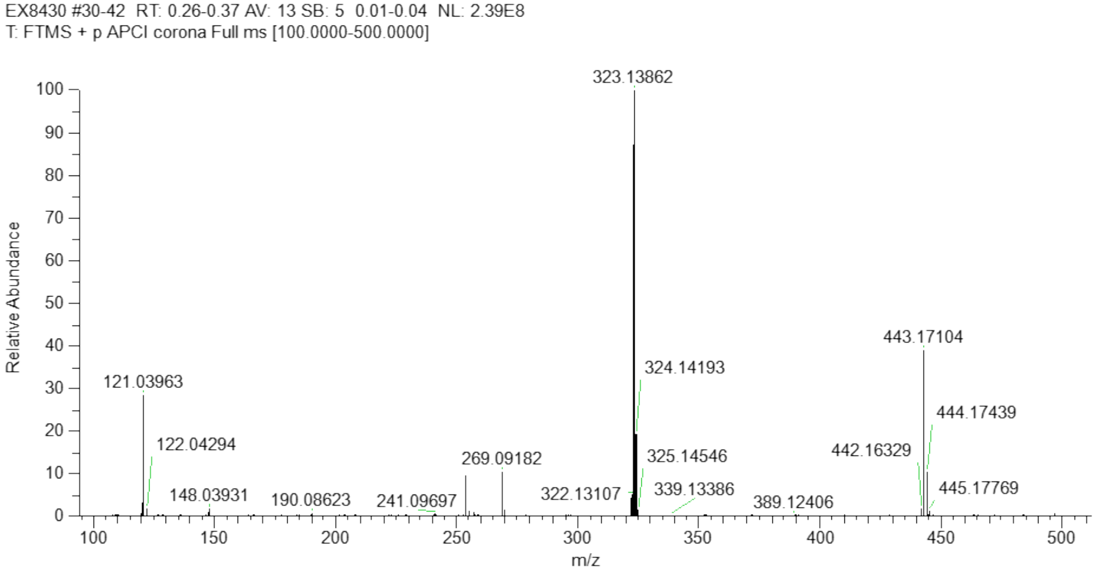

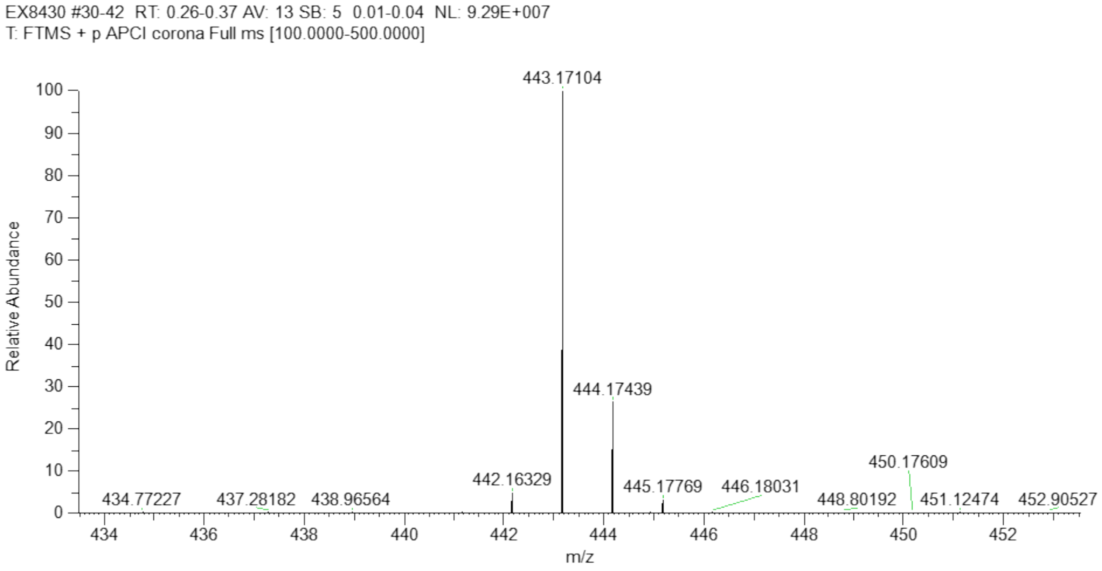

HRMS spectra of compound **36**

APCI Positive

Peak Mass	Display Formula	RDB	Delta [ppm]	Delta [mmu]	Theo. mass	Combined Score	MS Cov. [%]	Pattern Cov. [%]
447.14575	$C_{24}H_{20}O_4N_4F$	16.5	-1.25	-0.56	447.14631	96.63	99.59	99.71
447.14575	$C_{23}H_{24}O_8F$	11.5	1.74	0.78	447.14497	96.27	99.59	99.59
447.14575	$C_{25}H_{16}N_8F$	21.5	-4.24	-1.9	447.14765	92.63	97.26	94.21
447.14575	$C_7H_{24}O_{13}N_8F$	-0.5	3.61	1.61	447.14414	74.47	78.18	87.63
447.14575	$C_{20}H_{16}O_2N_{10}F$	17.5	4.76	2.13	447.14362	74.31	78.18	77.5

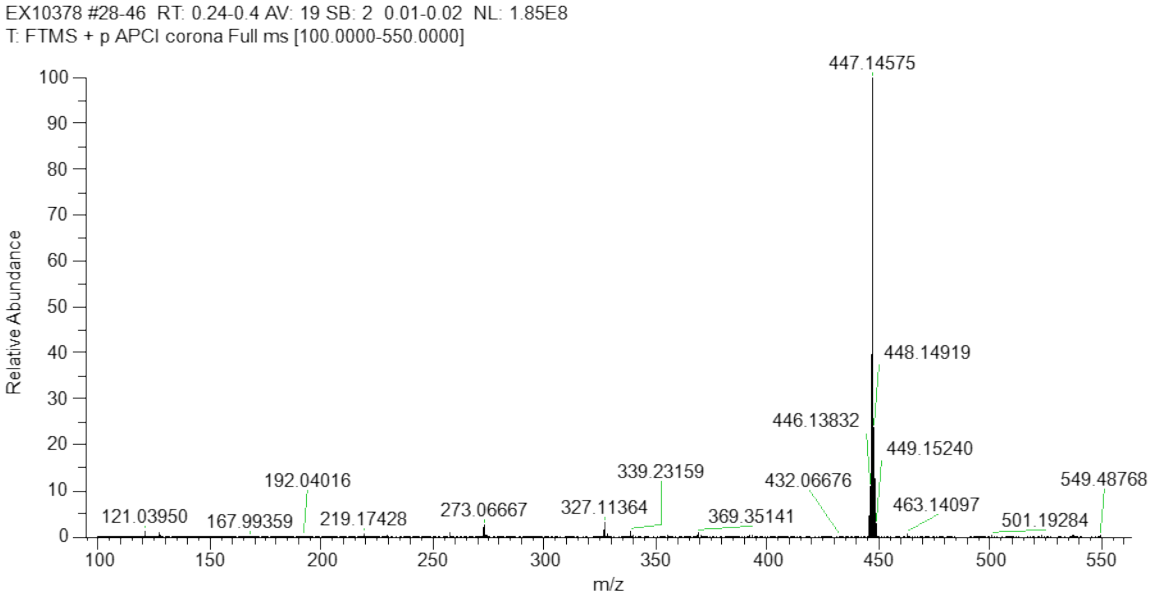

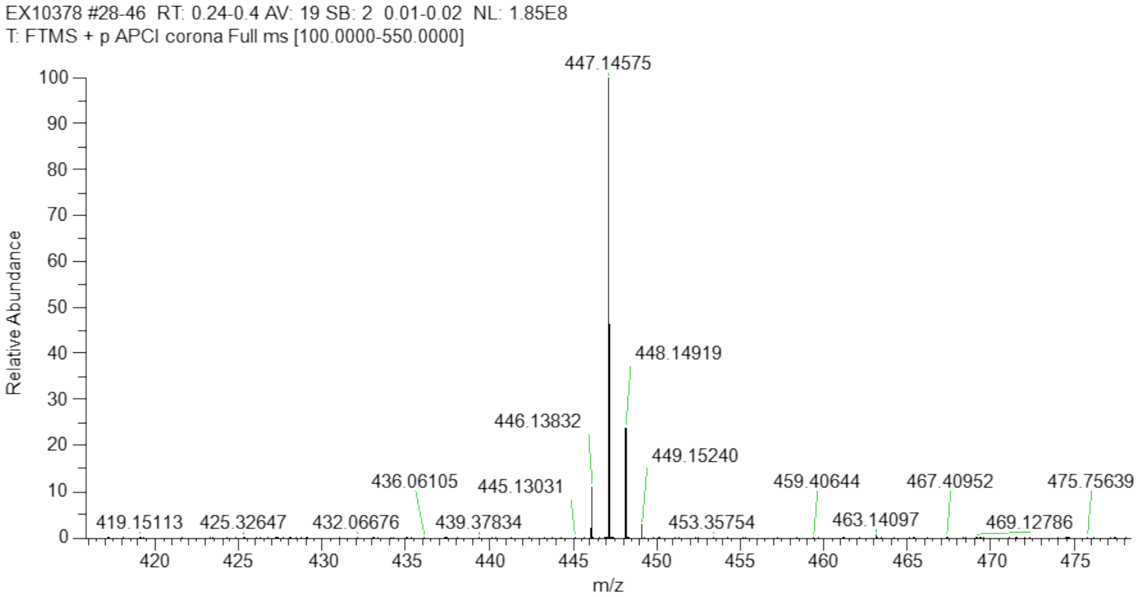

m/z	Relative
121.0395	1.314952
257.2473	1.063549
273.0667	2.467134
327.1136	3.301305
339.2316	1.132905
446.1383	10.9376
447.1458	100
448.1492	24.4035
449.1524	2.984615

HRMS spectra of compound **37**

APCI Positive

Peak Mass	Display Formula	RDB	Delta [ppm]	Delta [mmu]	Theo. mass	Combined Score	MS Cov. [%]	Pattern Cov. [%]
507.06607	$C_{24}H_{20}O_4N_4{}^{79}Br$	16.5	-0.34	-0.17	507.06624	92.18	94.45	99.84
507.06607	$C_{25}H_{16}N_8{}^{79}Br$	21.5	-2.97	-1.51	507.06758	91.48	94.45	99.89
507.06607	$C_{23}H_{24}O_8{}^{79}Br$	11.5	2.3	1.16	507.06491	90.25	93.3	98.17
507.06607	$C_{12}H_{24}O_{11}N_6{}^{79}Br$	3.5	-3.99	-2.02	507.06809	74.03	76.93	88.06
507.06607	$C_7H_{24}O_{13}N_8{}^{79}Br$	-0.5	3.95	2	507.06407	72.43	75.78	91.05
507.06607	$C_{20}H_{16}O_2N_{10}{}^{79}Br$	17.5	4.96	2.51	507.06356	72.26	75.78	81.14

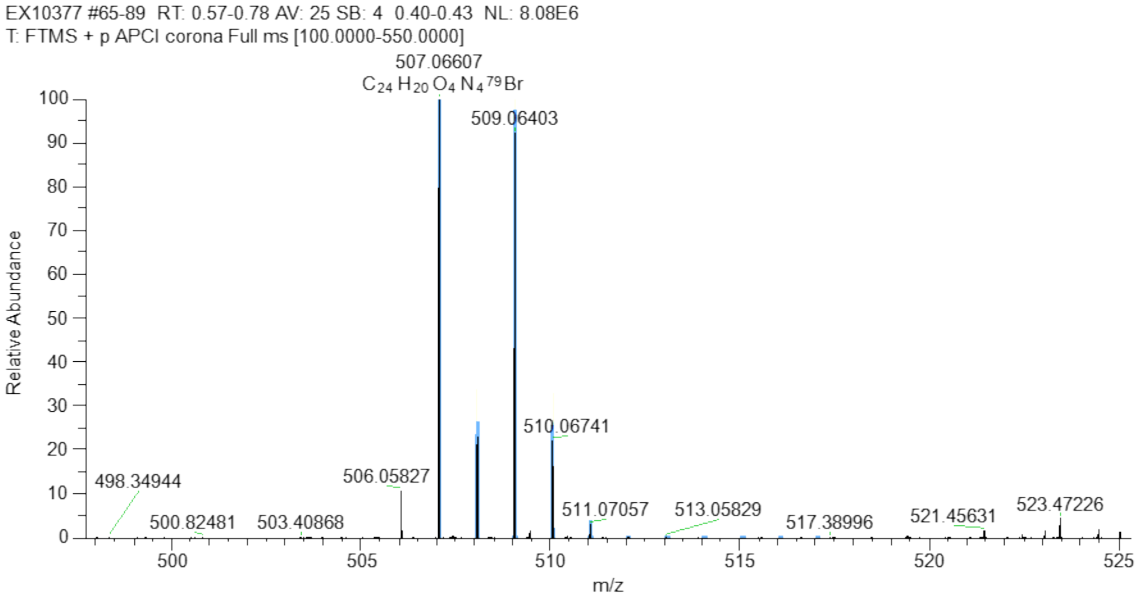

EX10377 #65-89 RT: 0.57-0.78 AV: 25 SB: 4 0.40-0.43 NL: 8.08E6
T: FTMS + p APCI corona Full ms [100.0000-550.0000]
507.06607
C24 H20 O4 N4 79Br
509.06403
510.06741
498.34944
500.82481
503.40868
506.05827
511.07057
513.05829
517.38996
521.45631
523.47226
Relative Abundance
m/z

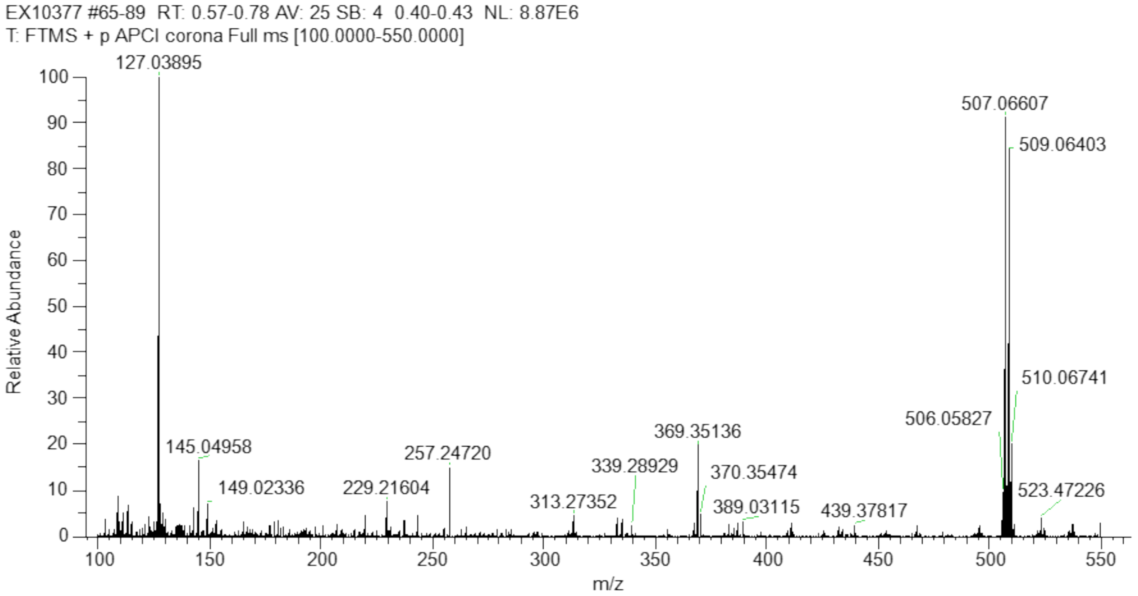

EX10377 #65-89 RT: 0.57-0.78 AV: 25 SB: 4 0.40-0.43 NL: 8.87E6
T: FTMS + p APCI corona Full ms [100.0000-550.0000]
127.03895
145.04958
149.02336
229.21604
257.24720
313.27352
339.28929
369.35136
370.35474
389.03115
439.37817
506.05827
507.06607
509.06403
510.06741
523.47226
Relative Abundance
m/z

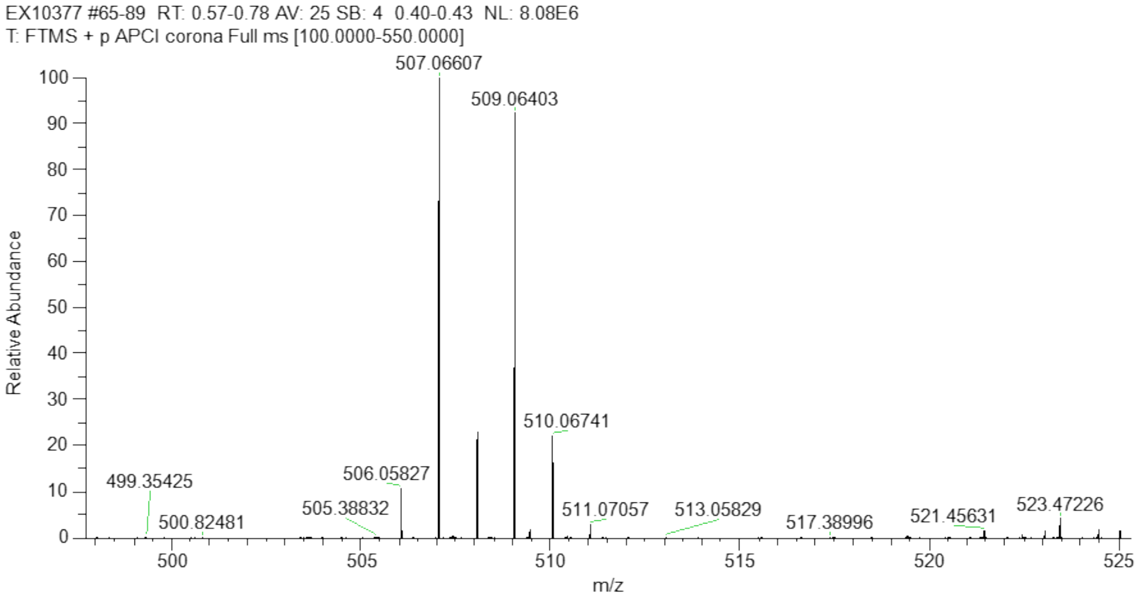

m/z	Relative
109.0285	8.593427
111.0441	4.972095
113.0233	6.777006
127.0389	100
128.0423	6.792383
129.0545	5.027119
143.034	6.25122
145.0496	16.03979
149.0234	6.827051
220.0604	4.681988
229.216	7.47214
257.2472	14.98403
369.3514	19.37284
370.3547	4.595676
506.0583	9.64127
507.0661	88.37251
508.0563	8.601179
508.0694	20.36468
509.064	84.41985
510.0674	19.59976

HRMS spectra of compound **38**

APCI Positive

Peak Mass	Display Formula	RDB	Delta [ppm]	Delta [mmu]	Theo. mass	Combined Score	MS Cov. [%]	Pattern Cov. [%]
481.10685	$C_{24}H_{19}O_{4}N_{4}{}^{35}ClF$	16.5	-1.02	-0.49	481.10734	93.13	96.19	97.72
481.10685	$C_{23}H_{23}O_{8}{}^{35}ClF$	11.5	1.76	0.85	481.106	92.25	95.68	96.71
481.10685	$C_{25}H_{15}N_{8}{}^{35}ClF$	21.5	-3.8	-1.82	481.10867	91.79	95.68	97.12
481.10685	$C_{20}H_{15}O_{2}N_{10}{}^{35}ClF$	17.5	4.56	2.2	481.10465	80.16	83.02	86.71
481.10685	$C_{12}H_{23}O_{11}N_{6}{}^{35}ClF$	3.5	-4.86	-2.34	481.10919	74.41	77.24	88.13
481.10685	$C_{7}H_{23}O_{13}N_{8}{}^{35}ClF$	-0.5	3.5	1.69	481.10516	73.52	76.73	91.78

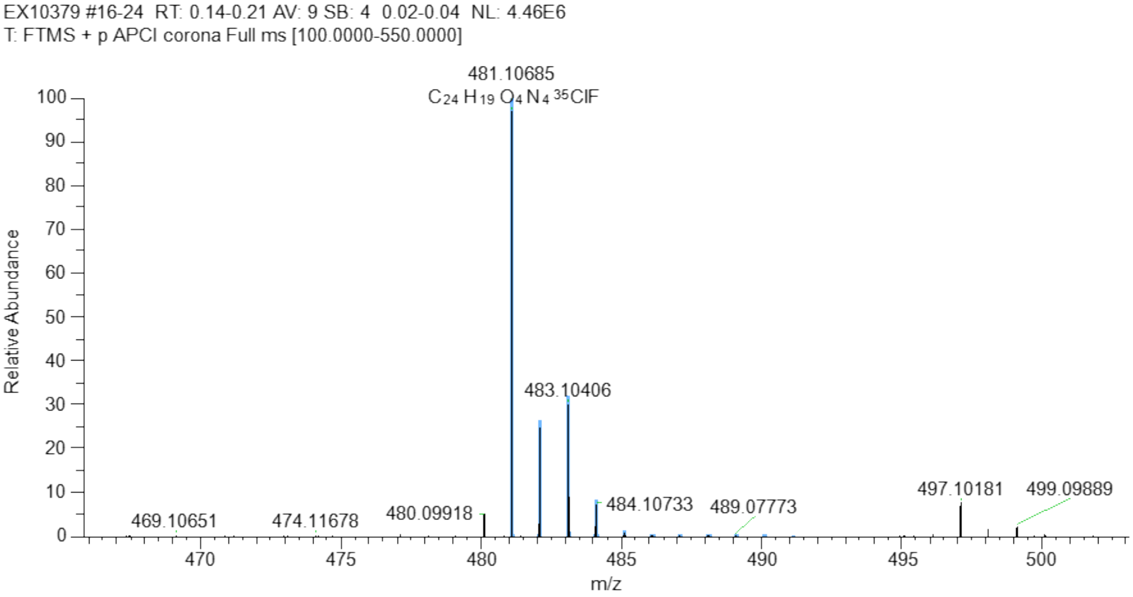

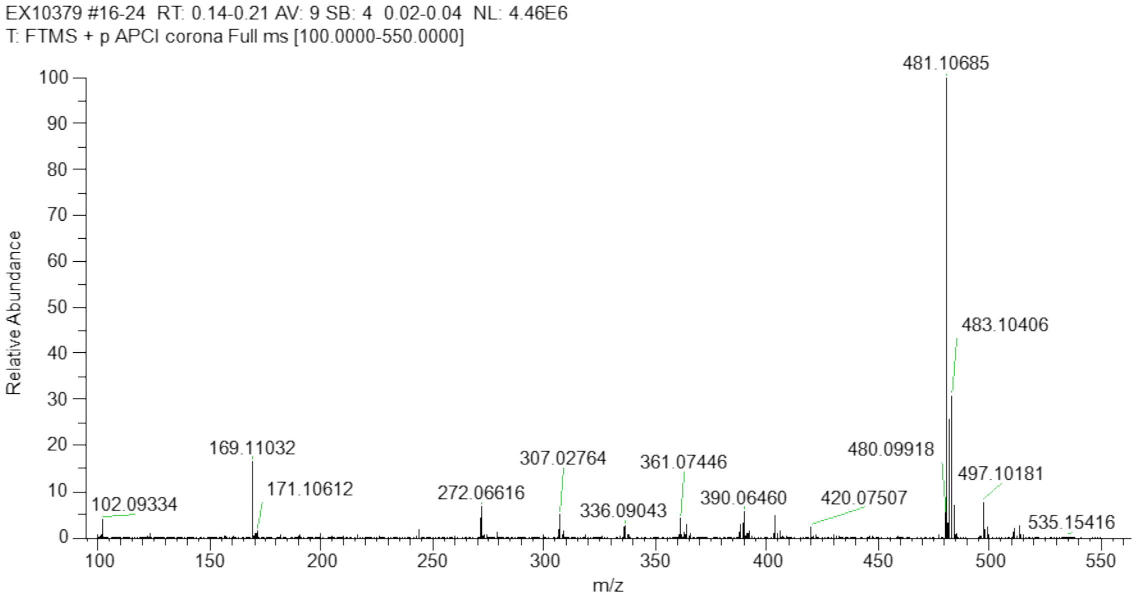

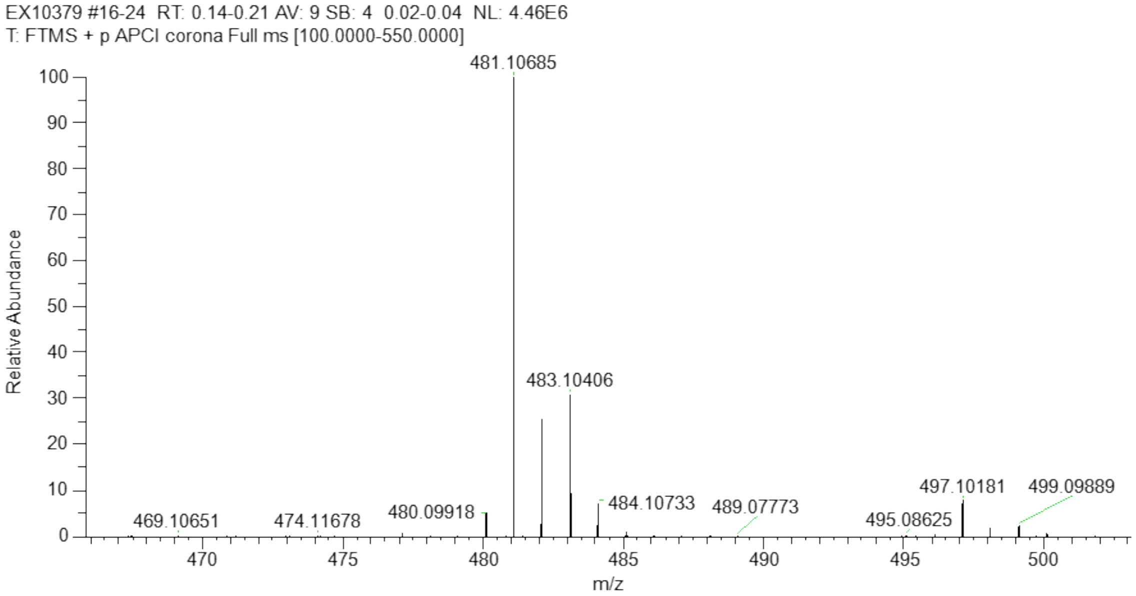

m/z	Relative
102.0933	3.873851
169.1103	15.88038
272.0662	6.635339
307.0276	4.974269
336.0904	2.588199
361.0745	4.143009
364.0853	2.811874
388.0853	2.713315
390.0646	5.494069
404.0803	4.594513

420.0751	2.325107
480.0992	4.888367
481.1068	100
482.1103	24.98581
483.1041	30.20013
483.1125	2.639679
484.1073	7.165355
497.1018	7.488023
499.0989	2.176368
513.097	2.463531

HRMS spectra of compound **41**

APCI Positive

Peak Mass	Display Formula	RDB	Delta [ppm]	Delta [mmu]	Theo. mass	Combined Score	MS Cov. [%]
521.07988	$C_{24}H_{18}O_4N_6{}^{35}Cl^{32}S$	18.5	1.06	0.55	521.07933	89.91	93.68
521.07988	$C_{25}H_{14}N_{10}{}^{35}Cl^{32}S$	23.5	-1.5	-0.79	521.08067	89.73	93.68
521.07988	$C_{23}H_{22}O_8N_2{}^{35}Cl^{32}S$	13.5	3.63	1.89	521.07799	89.59	93.68
521.07988	$C_{12}H_{22}O_{11}N_8{}^{35}Cl^{32}S$	5.5	-2.49	-1.3	521.08118	73.55	76.65
521.07988	$C_{11}H_{26}O_{15}N_4{}^{35}Cl^{32}S$	0.5	0.08	0.04	521.07984	72.82	76.13
521.07988	$C_{28}H_{22}O_6{}^{35}Cl^{32}S$	17.5	-4.09	-2.13	521.08201	72.53	76.13

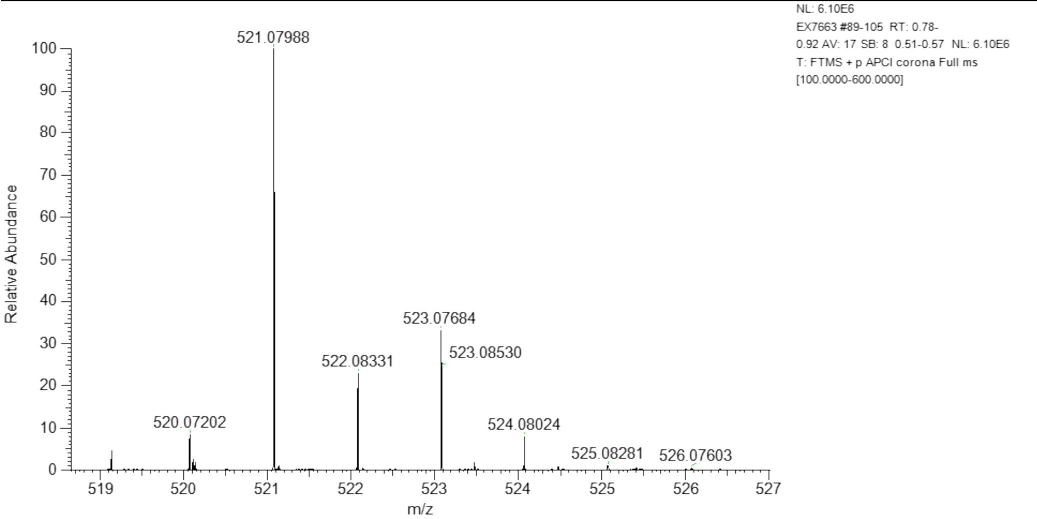

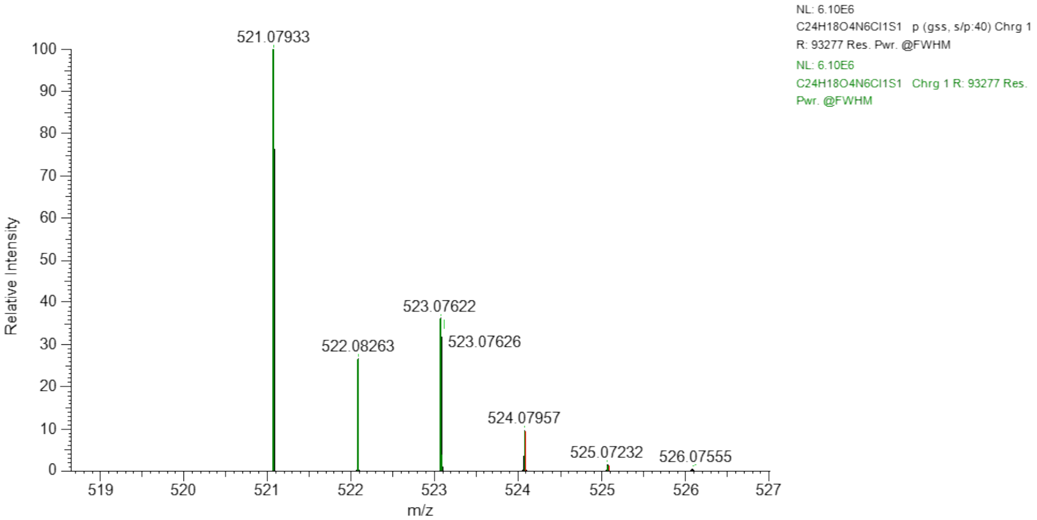

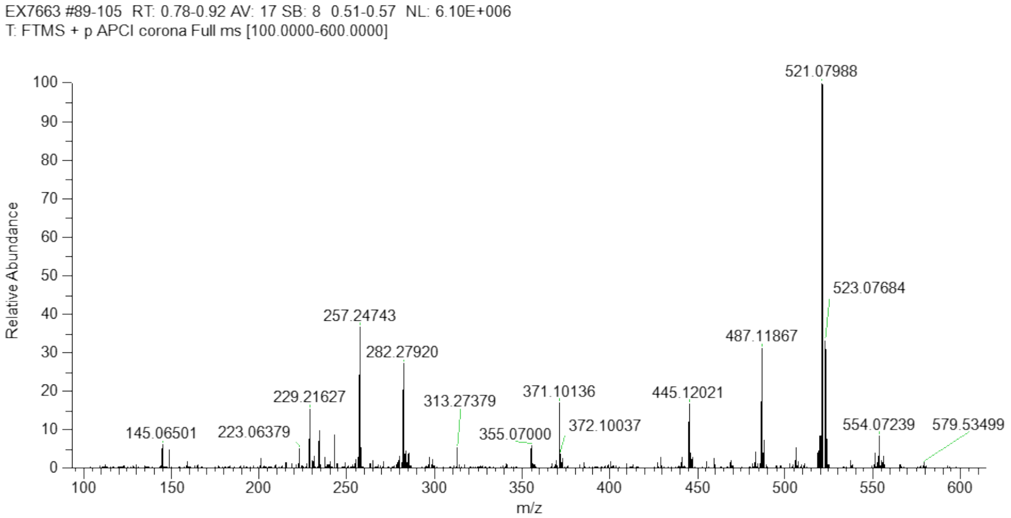

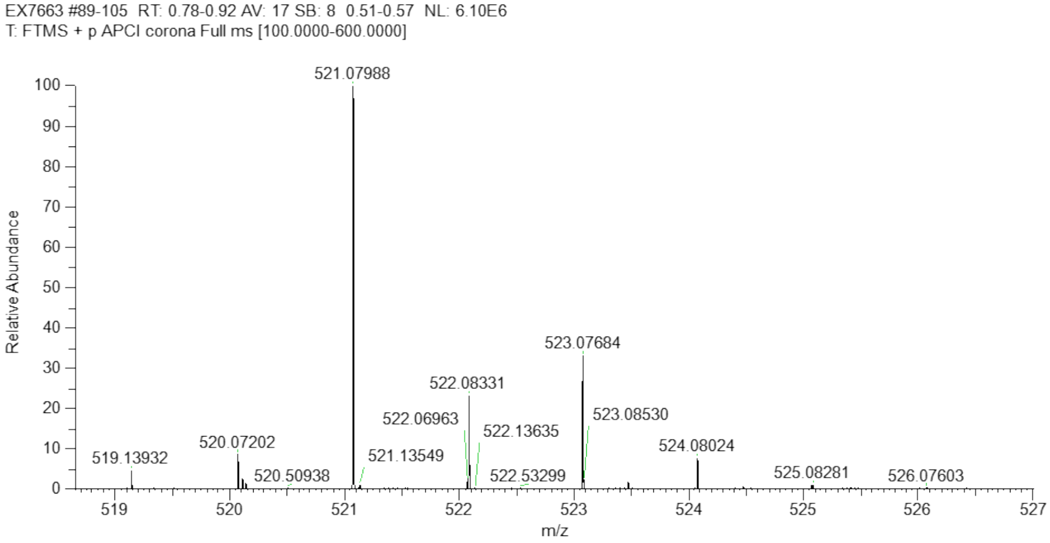

HRMS spectra of compound **42**

APCI Positive

Peak Mass	Display Formula	RDB	Delta [ppm]	Delta [mmu]	Theo. mass	Combined Score	MS Cov. [%]
521.07932	$C_{24}H_{18}O_4N_6{}^{35}Cl{}^{32}S$	18.5	-0.02	-0.01	521.07933	91.49	95.24
521.07932	$C_{23}H_{22}O_8N_2{}^{35}Cl{}^{32}S$	13.5	2.55	1.33	521.07799	91.3	95.24
521.07932	$C_{25}H_{14}N_{10}{}^{35}Cl{}^{32}S$	23.5	-2.59	-1.35	521.08067	91.28	95.24
521.07932	$C_{12}H_{22}O_{11}N_8{}^{35}Cl{}^{32}S$	5.5	-3.57	-1.86	521.08118	73.98	77.34
521.07932	$C_{11}H_{26}O_{15}N_4{}^{35}Cl{}^{32}S$	0.5	-1	-0.52	521.07984	73.52	76.89
521.07932	$C_7H_{22}O_{13}N_{10}{}^{35}Cl{}^{32}S$	1.5	4.15	2.16	521.07716	73.36	76.89

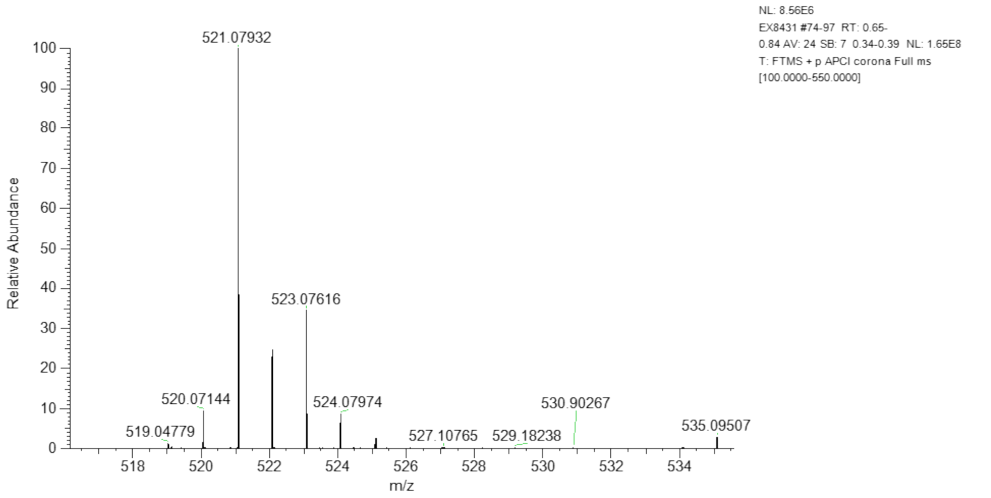

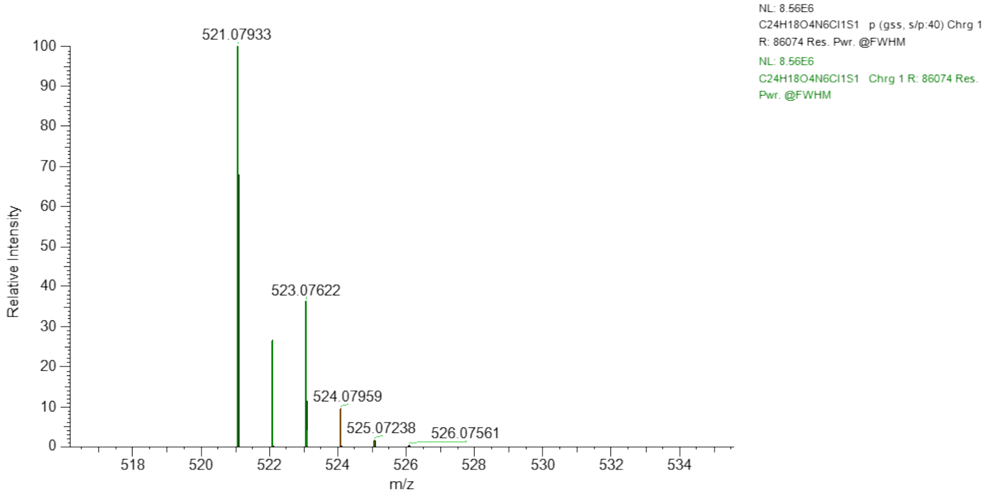

EX8431 #74-97 RT: 0.65-0.84 AV: 24 SB: 7 0.34-0.39 NL: 1.65E+008
T: FTMS + p APCI corona Full ms [100.0000-550.0000]

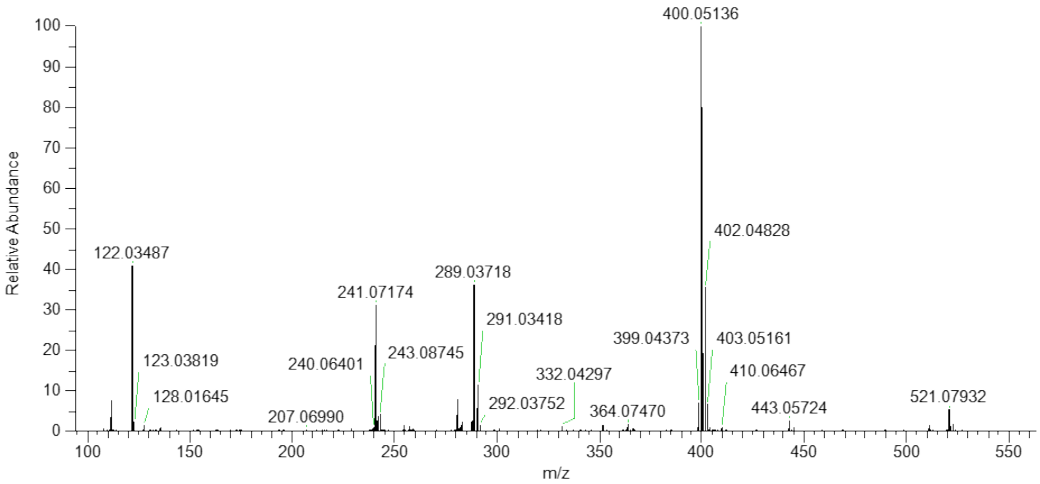

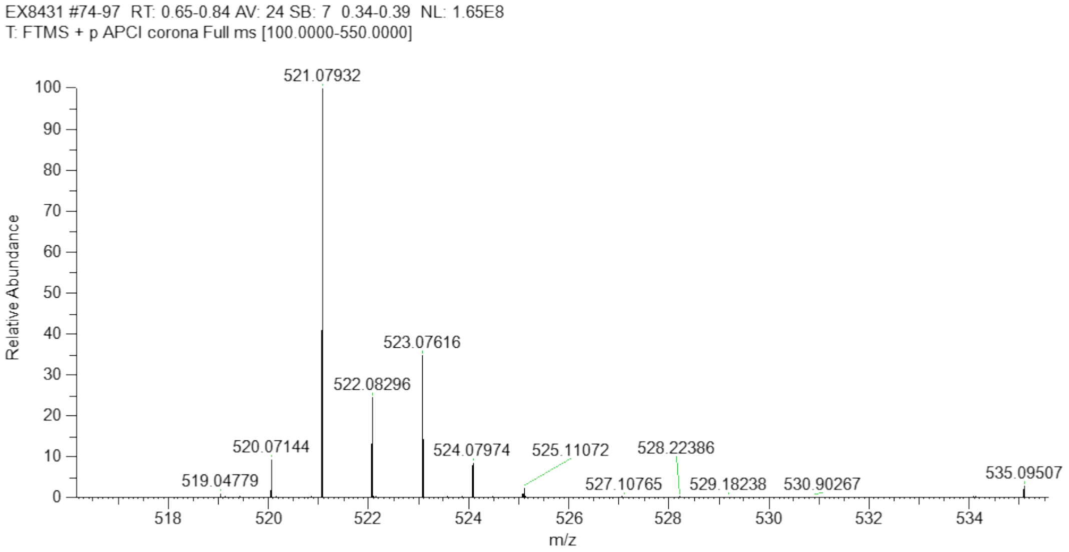

HRMS spectra of compound **43**

APCI Positive

Peak Mass	Display Formula	RDB	Delta [ppm]	Delta [mmu]	Theo. mass	Combined Score	MS Cov. [%]
540.09936	$C_{24}H_{17}O_4N_7{}^{35}ClF_2$	18.5	0.09	0.05	540.09931	87.52	90.6
540.09936	$C_{23}H_{21}O_8N_3{}^{35}ClF_2$	13.5	2.57	1.38	540.09798	87.29	90.6
540.09936	$C_{12}H_{21}O_{11}N_9{}^{35}ClF_2$	5.5	-3.33	-1.8	540.10116	71.45	73.84
540.09936	$C_{11}H_{25}O_{15}N_5{}^{35}ClF_2$	0.5	-0.86	-0.47	540.09983	70.41	73.35
540.09936	$C_{28}H_{21}O_6N^{35}ClF_2$	17.5	-4.88	-2.64	540.102	53.77	56.55

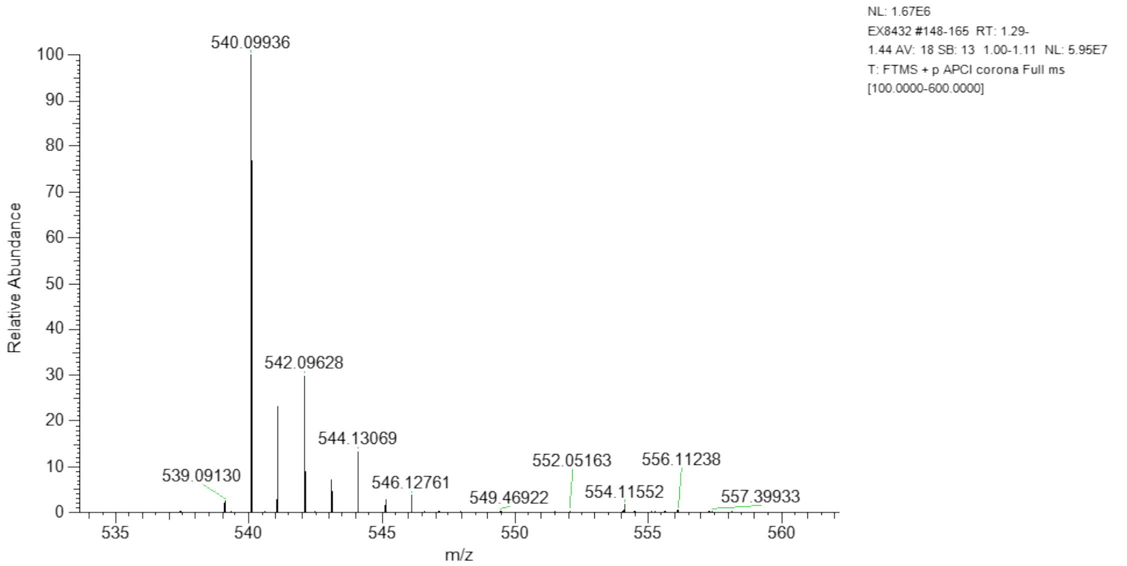
NL: 1.67E6
EX8432 #148-165 RT: 1.29-
1.44 AV: 18 SB: 13 1.00-1.11 NL: 5.95E7
T: FTMS + p APCI corona Full ms
[100.0000-600.0000]
540.09936
542.09628
544.13069
539.09130
546.12761
549.46922
552.05163
554.11552
556.11238
557.39933
Relative Abundance
m/z

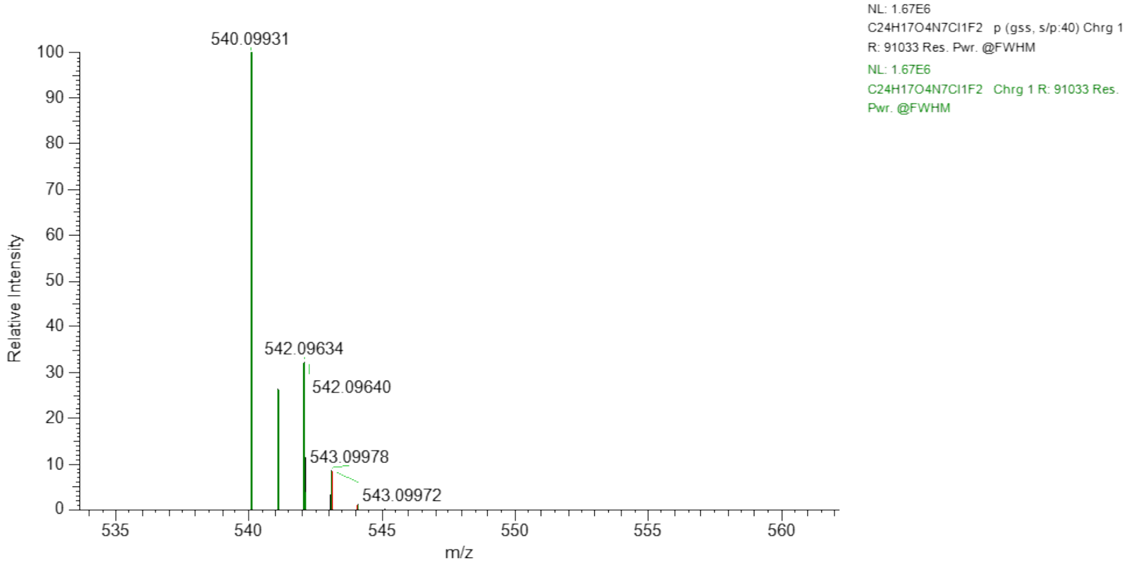
NL: 1.67E6
C24H17O4N7Cl1F2 p (gss, s/p:40) Chrg 1
R: 91033 Res. Pwr. @FWHM
NL: 1.67E6
C24H17O4N7Cl1F2 Chrg 1 R: 91033 Res. Pwr. @FWHM
540.09931
542.09634
542.09640
543.09978
543.09972
Relative Intensity
m/z

EX8432 #148-165 RT: 1.29-1.44 AV: 18 SB: 13 1.00-1.11 NL: 5.95E+007
T: FTMS + p APCI corona Full ms [100.0000-600.0000]

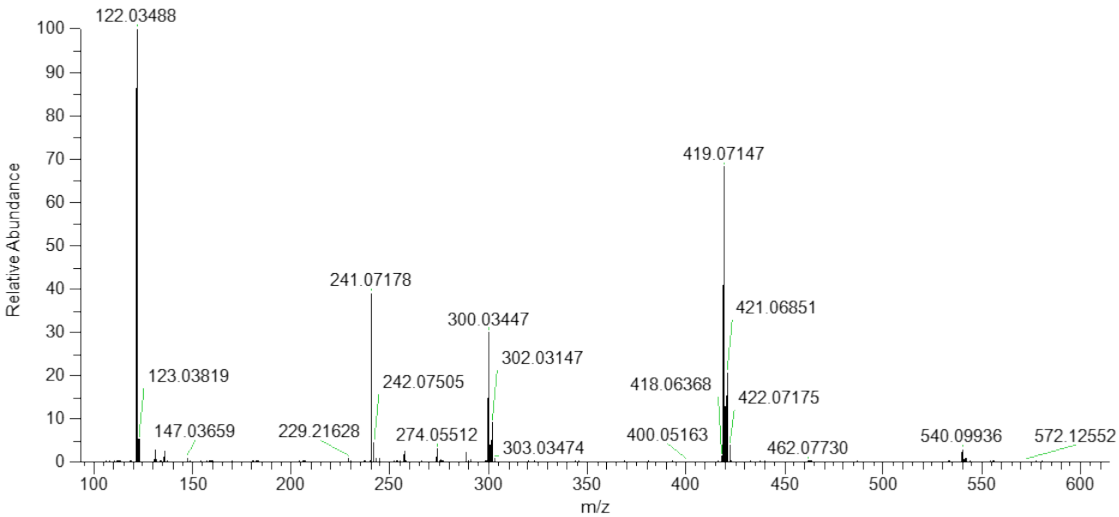

EX8432 #148-165 RT: 1.29-1.44 AV: 18 SB: 13 1.00-1.11 NL: 5.95E7
T: FTMS + p APCI corona Full ms [100.0000-600.0000]

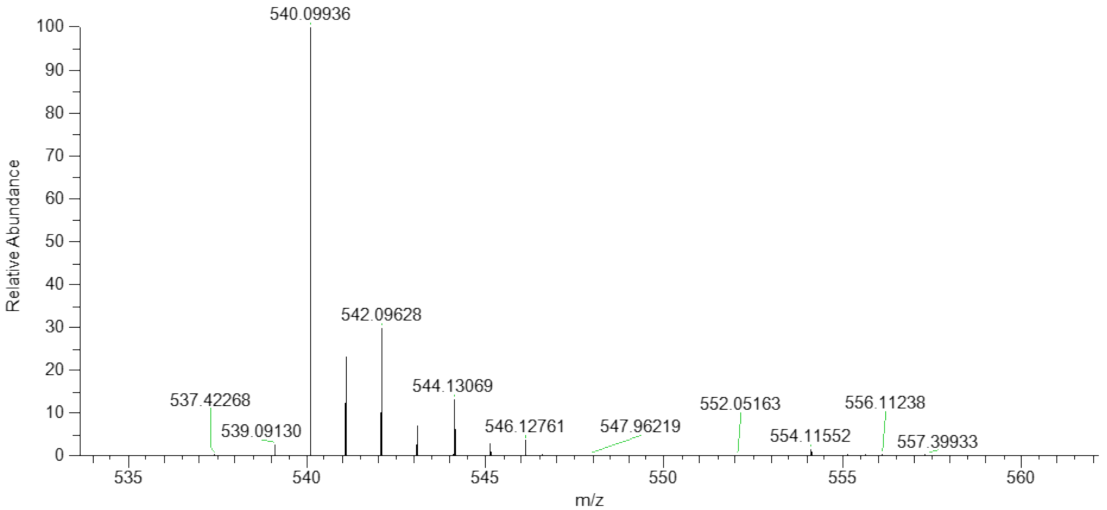

HRMS spectrum of compound **44**

APCI Positive

Peak Mass	Display Formula	RDB	Delta [ppm]	Delta [mmu]	Theo. mass	Combined Score	MS Cov. [%]
464.11174	$C_{21}H_{17}O_3N_8{}^{35}Cl$	17	2.31	1.07	464.11067	95.22	97.95
464.11174	$C_{23}H_{19}O_4N_5{}^{35}Cl$	16.5	-0.58	-0.27	464.11201	93.34	96.66
464.11174	$C_{24}H_{15}N_9{}^{35}Cl$	21.5	-3.46	-1.61	464.11335	92.82	96.66
464.11174	$C_{22}H_{23}O_8N^{35}Cl$	11.5	2.3	1.07	464.11067	92.38	96.16
464.11174	$C_{25}H_{21}O_5N_2{}^{35}Cl$	16	-3.48	-1.61	464.11335	88.04	92.11
464.11174	$C_9H_{21}O_{10}N_{10}{}^{35}Cl$	4	-1.68	-0.78	464.11252	77	79.89
464.11174	$C_{11}H_{23}O_{11}N_7{}^{35}Cl$	3.5	-4.57	-2.12	464.11386	76.92	79.89
464.11174	$C_8H_{25}O_{14}N_6{}^{35}Cl$	-1	1.21	0.56	464.11118	74.66	78.1
464.11174	$C_6H_{23}O_{13}N_9{}^{35}Cl$	-0.5	4.1	1.9	464.10984	74.43	78.1

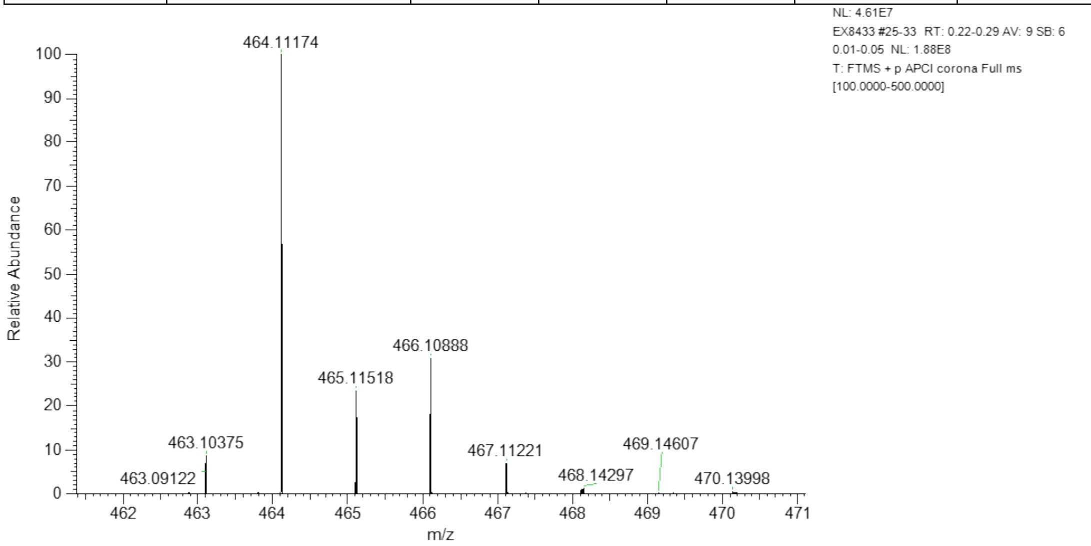

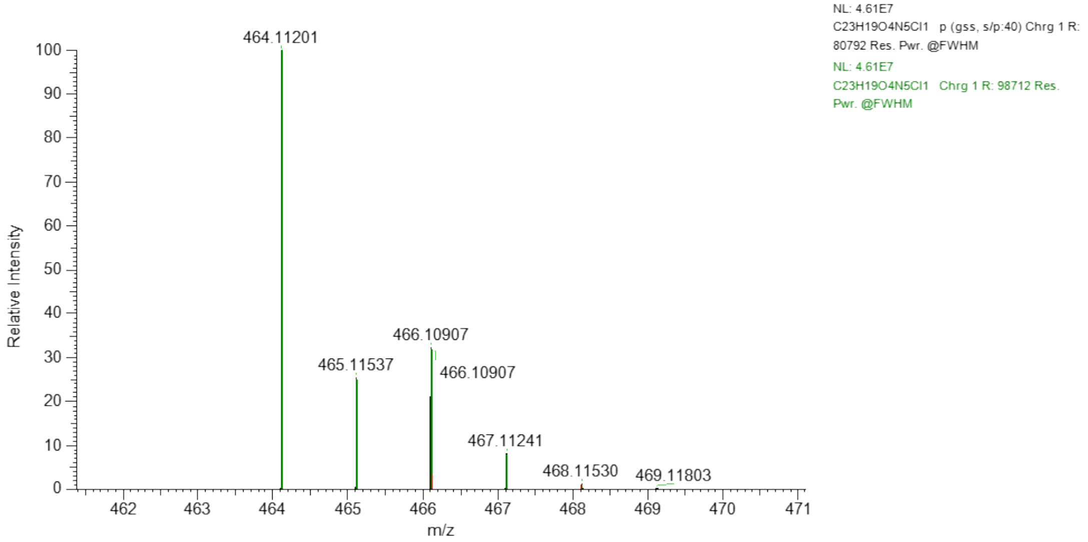

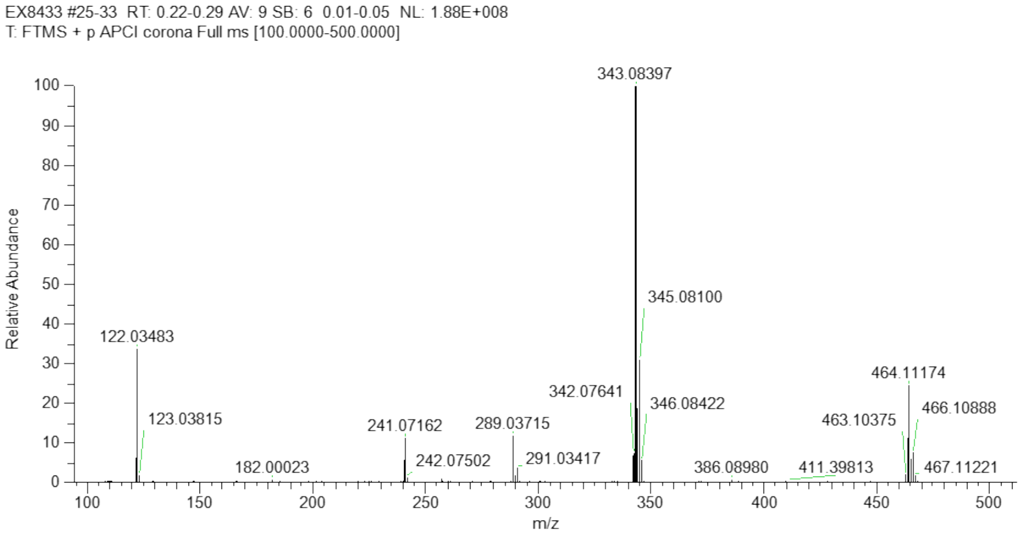

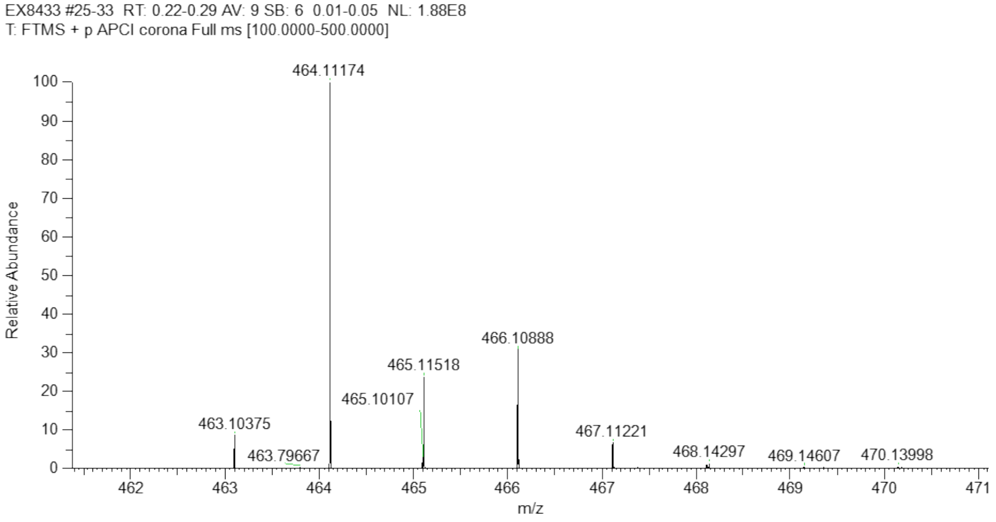

HRMS spectrum of compound **45**

APCI Positive

Peak Mass	Display Formula	RDB	Delta [ppm]	Delta [mmu]	Theo. mass	Combined Score	MS Cov. [%]
537.07396	$C_{23}H_{22}O_9N_2{}^{35}Cl{}^{32}S$	13.5	1.97	1.05	537.0729	86.34	89.82
537.07396	$C_{24}H_{18}O_5N_6{}^{35}Cl{}^{32}S$	18.5	-0.52	-0.28	537.0742	86.22	89.82
537.07396	$C_{25}H_{14}ON_{10}{}^{35}Cl{}^{32}S$	23.5	-3.01	-1.62	537.0756	85.95	89.82
537.07396	$C_{12}H_{22}O_{12}N_8{}^{35}Cl{}^{32}S$	5.5	-3.97	-2.13	537.0761	76.3	79.63
537.07396	$C_7H_{22}O_{14}N_{10}{}^{35}Cl{}^{32}S$	1.5	3.52	1.89	537.0721	73.91	77.16

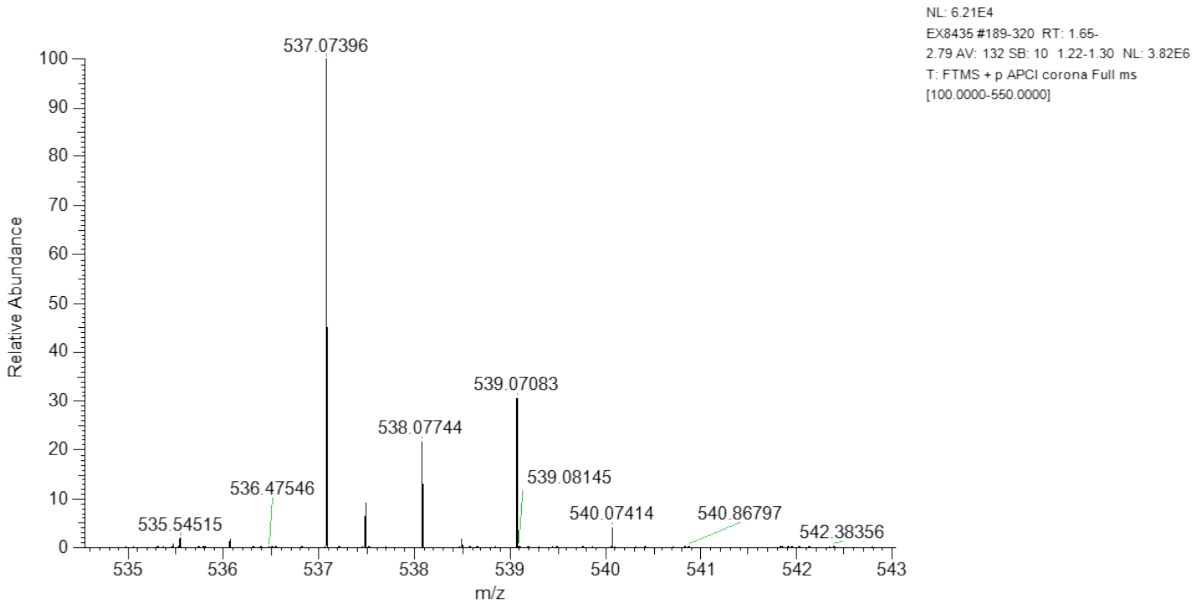
NL: 6.21E4
EX8435 #189-320 RT: 1.65-
2.79 AV: 132 SB: 10 1.22-1.30 NL: 3.82E6
T: FTMS + p APCI corona Full ms
[100.0000-550.0000]
537.07396
539.07083
538.07744
539.08145
536.47546
540.07414
540.86797
535.54515
542.38356
Relative Abundance
m/z

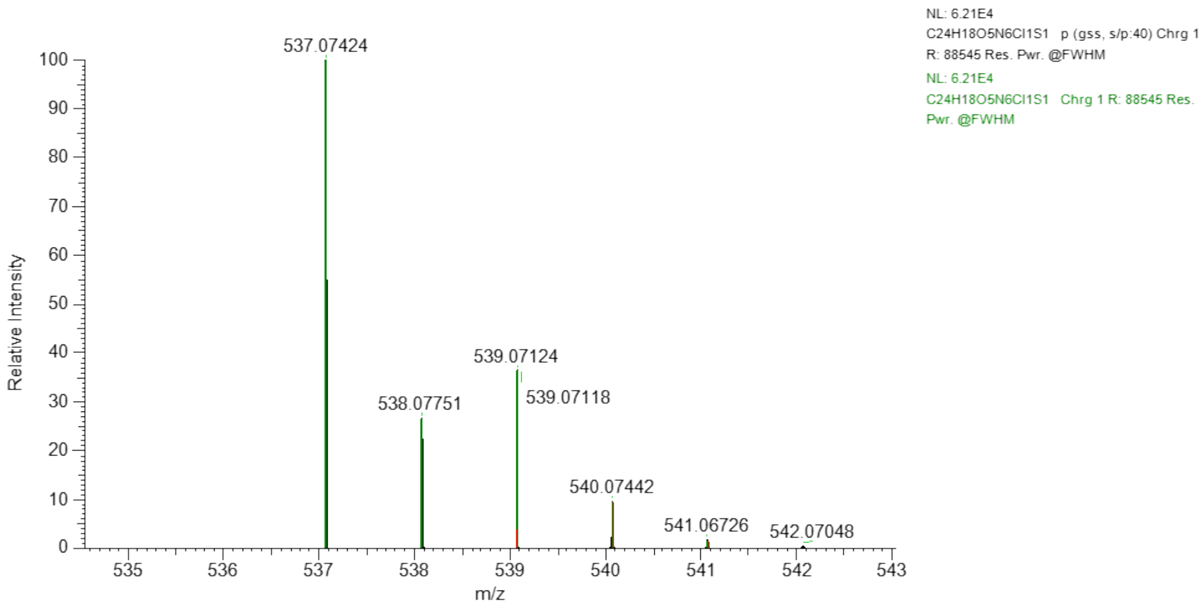
NL: 6.21E4
C24H18O5N6Cl1S1 p (gss, s/p:40) Chrg 1
R: 88545 Res. Pwr. @FWHM
NL: 6.21E4
C24H18O5N6Cl1S1 Chrg 1 R: 88545 Res.
Pwr. @FWHM
537.07424
539.07124
539.07118
538.07751
540.07442
541.06726
542.07048
Relative Intensity
m/z

HRMS spectrum of compound **46**

APCI Positive

Peak Mass	Display Formula	RDB	Delta [ppm]	Delta [mmu]	Theo. mass	Combined Score	MS Cov. [%]
556.09414	$C_{23}H_{21}O_9N_3{}^{35}ClF_2$	13.5	2.24	1.25	556.09289	95.16	98.58
556.09414	$C_{24}H_{17}O_5N_7{}^{35}ClF_2$	18.5	-0.17	-0.09	556.09423	95.02	98.58
556.09414	$C_{12}H_{21}O_{12}N_9{}^{35}ClF_2$	5.5	-3.49	-1.94	556.09608	76.7	80.26
556.09414	$C_{28}H_{21}O_7N^{35}ClF_2$	17.5	-4.99	-2.77	556.09691	59.03	62.11

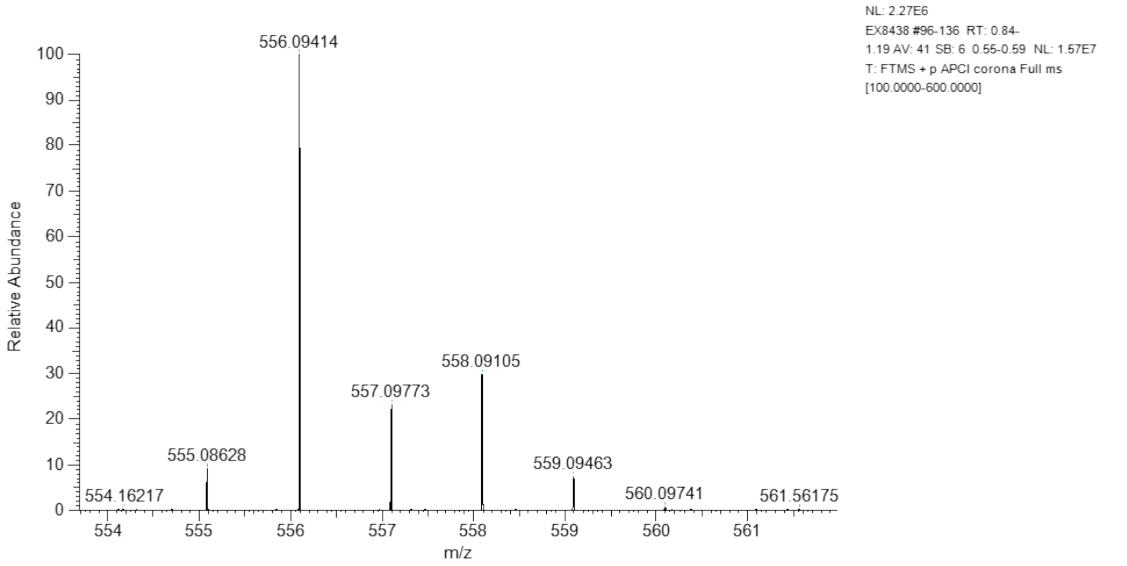

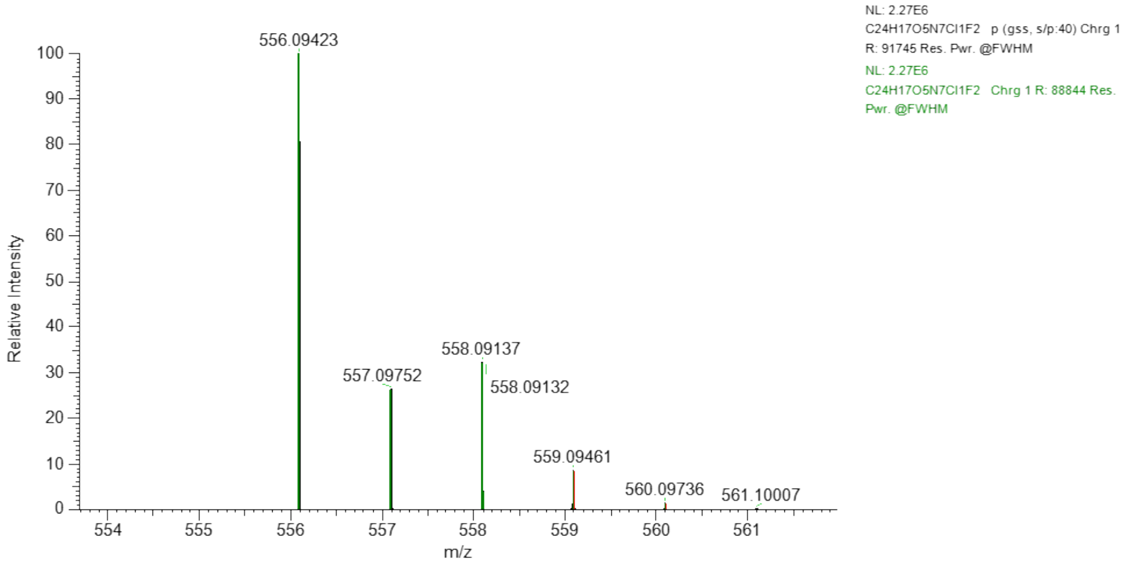

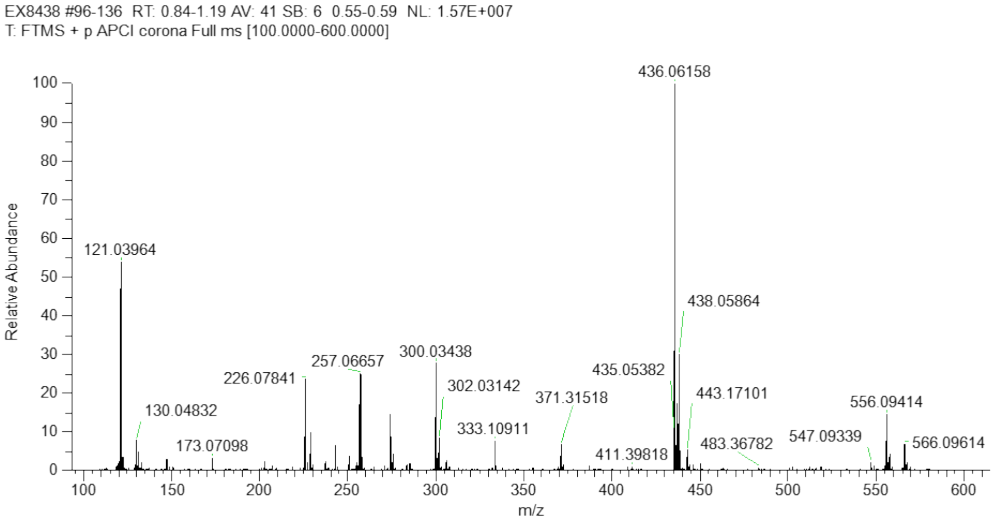

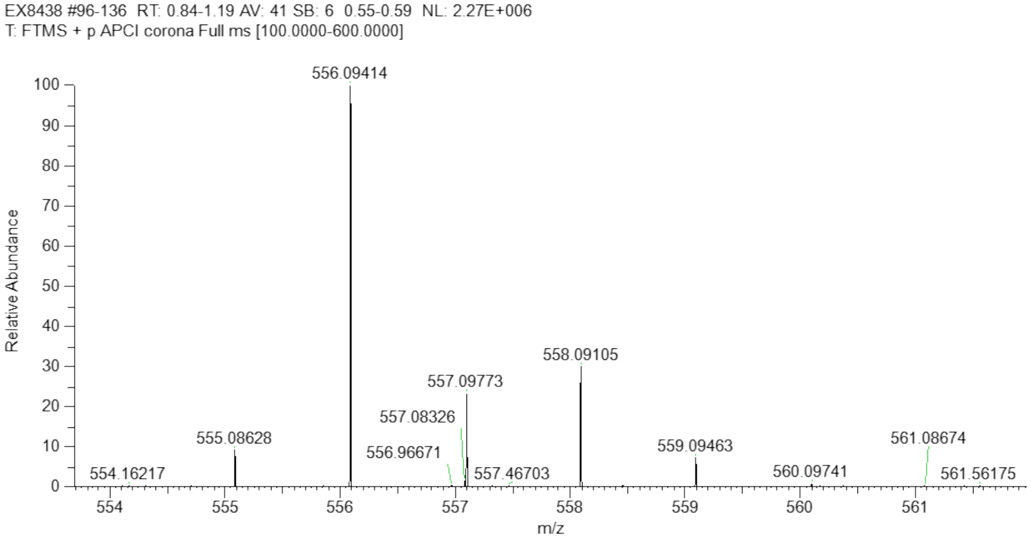

HRMS spectrum of compound **47**

APCI Positive

Peak Mass	Display Formula	RDB	Delta [ppm]	Delta [mmu]	Theo. mass	Combined Score	MS Cov. [%]
480.10647	$C_{23}H_{19}O_5N_5{}^{35}Cl$	16.5	-0.95	-0.45	480.10692	90.36	93.58
480.10647	$C_{22}H_{23}O_9N^{35}Cl$	11.5	1.84	0.88	480.10559	90.29	93.58
480.10647	$C_{24}H_{15}ON_9{}^{35}Cl$	21.5	-3.74	-1.79	480.10826	89.78	93.58
480.10647	$C_{11}H_{23}O_{12}N_7{}^{35}Cl$	3.5	-4.8	-2.3	480.10877	72.42	75.82
480.10647	$C_6H_{23}O_{14}N_9{}^{35}Cl$	-0.5	3.57	1.72	480.10475	72.42	75.82

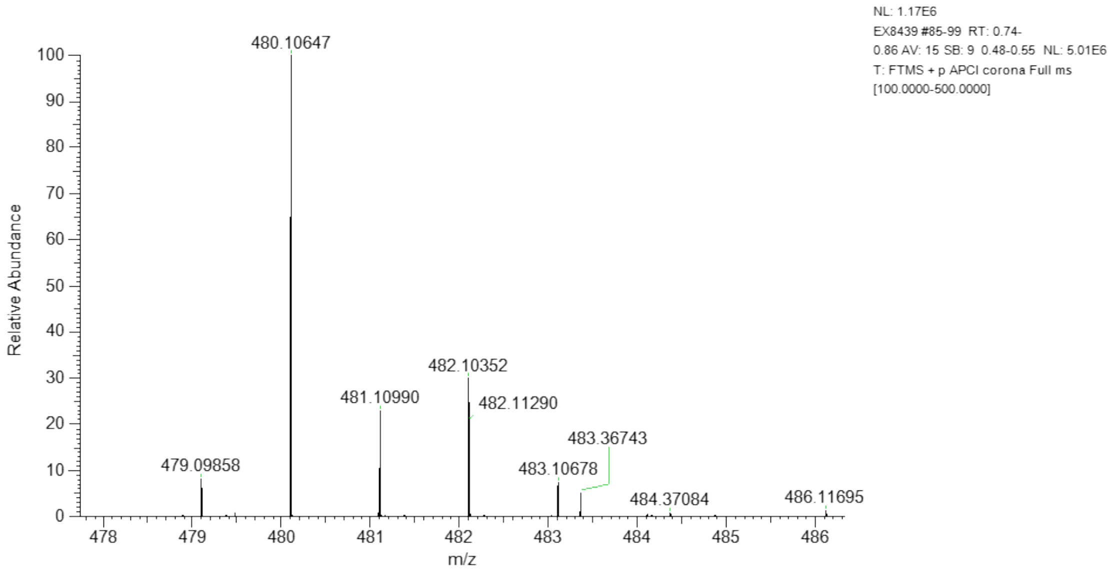
NL: 1.17E6
EX8439 #85-99 RT: 0.74-
0.86 AV: 15 SB: 9 0.48-0.55 NL: 5.01E6
T: FTMS + p APCI corona Full ms
[100.0000-500.0000]
Relative Abundance
m/z
480.10647
481.10990
482.10352
482.11290
483.36743
483.10678
479.09858
484.37084
486.11695

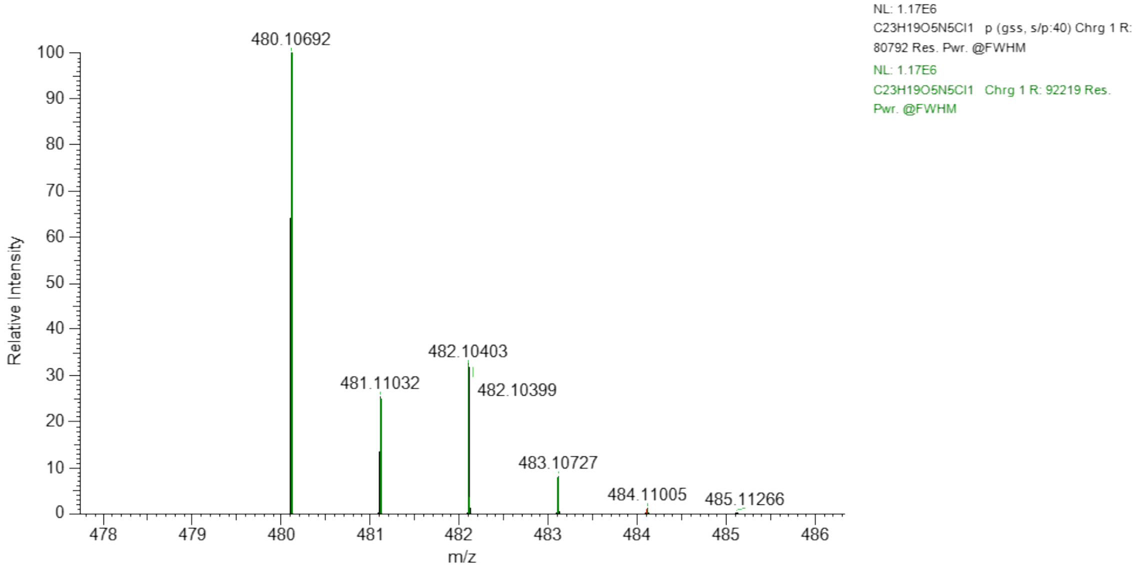
NL: 1.17E6
C23H19O5N5Cl1 p (gss, s/p:40) Chrg 1 R:
80792 Res. Pwr. @FWHM
NL: 1.17E6
C23H19O5N5Cl1 Chrg 1 R: 92219 Res.
Pwr. @FWHM
Relative Intensity
m/z
480.10692
481.11032
482.10403
482.10399
483.10727
484.11005
485.11266

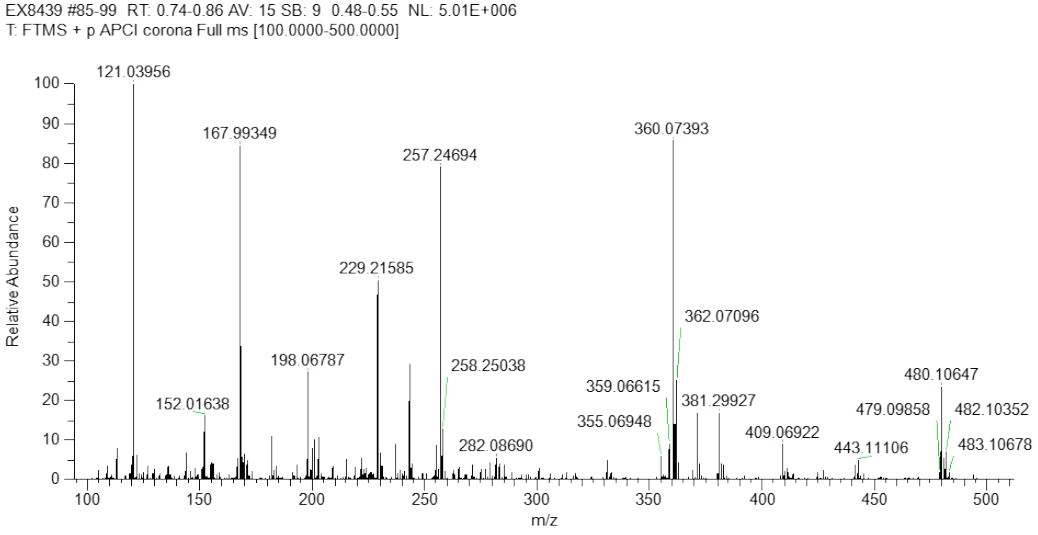
EX8439 #85-99 RT: 0.74-0.86 AV: 15 SB: 9 0.48-0.55 NL: 5.01E+006
T: FTMS + p APCI corona Full ms [100.0000-500.0000]
Relative Abundance
m/z
121.03956
167.99349
257.24694
360.07393
229.21585
362.07096
198.06787
258.25038
359.06615
152.01638
381.29927
355.06948
409.06922
282.08690
480.10647
479.09858
482.10352
443.11106
483.10678

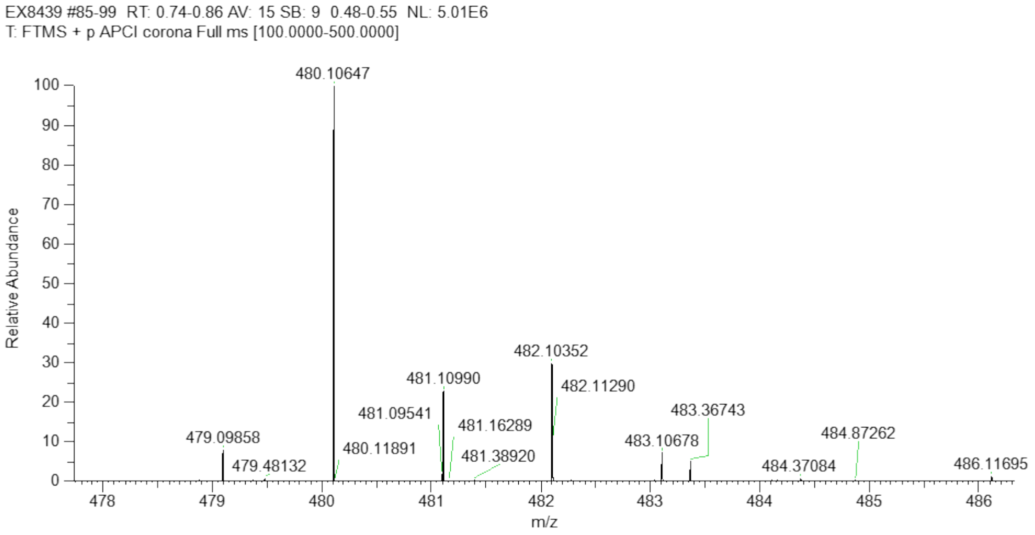

HRMS spectrum of compound **48**

APCI Positive

Peak Mass	Display Formula	RDB	Delta [ppm]	Delta [mmu]	Theo. mass	Combined Score	MS Cov. [%]
551.08948	$C_{24}H_{24}O_9N_2{}^{35}Cl^{32}S$	13.5	1.67	0.92	551.08856	64.67	67.04
551.08948	$C_{25}H_{20}O_5N_6{}^{35}Cl^{32}S$	18.5	-0.76	-0.41	551.08989	64.57	67.04
551.08948	$C_{26}H_{16}ON_{10}{}^{35}Cl^{32}S$	23.5	-3.18	-1.75	551.09123	64.34	67.04
551.08948	$C_{13}H_{24}O_{12}N_8{}^{35}Cl^{32}S$	5.5	-4.11	-2.26	551.09174	56.75	58.92
551.08948	$C_8H_{24}O_{14}N_{10}{}^{35}Cl^{32}S$	1.5	3.19	1.76	551.08772	54.18	56.42

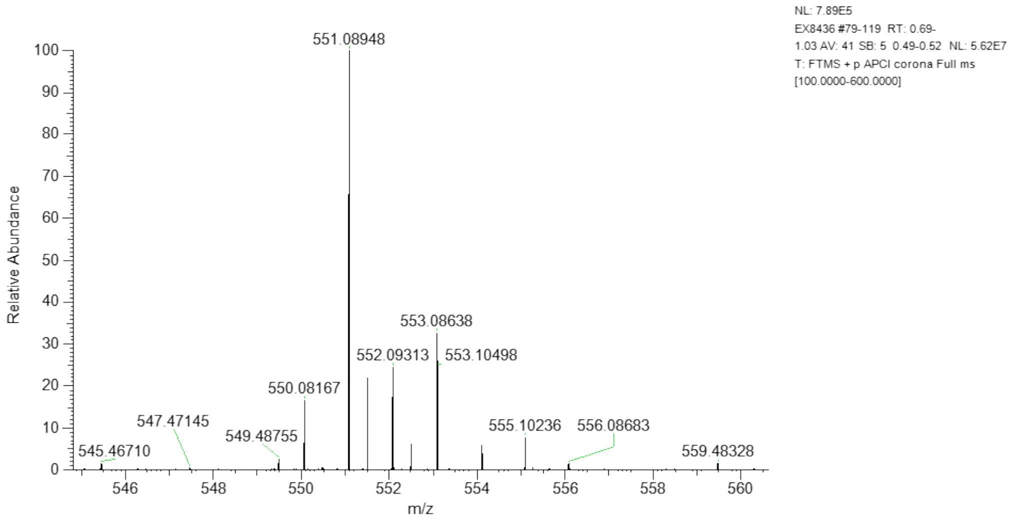

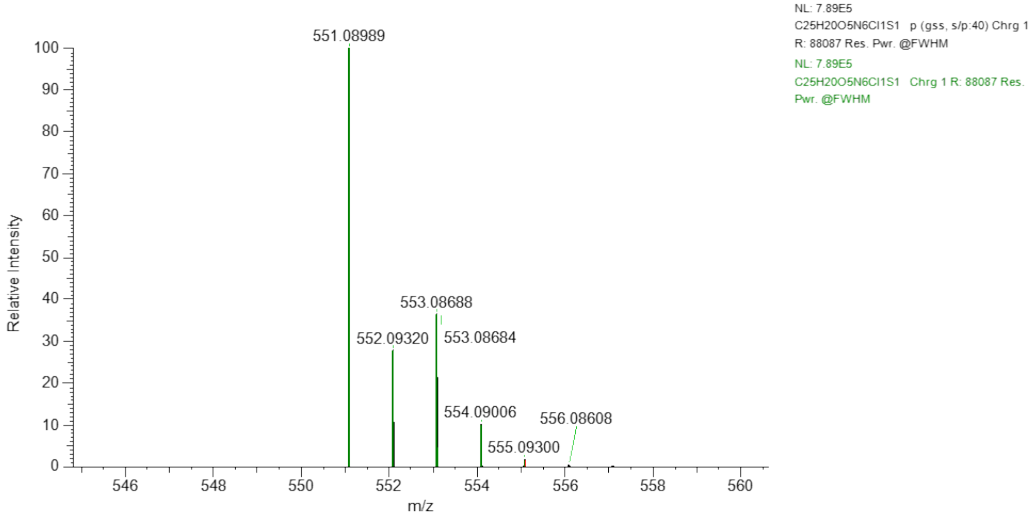

HRMS spectrum of compound **49**

APCI Positive

Peak Mass	Display Formula	RDB	Delta [ppm]	Delta [mmu]	Theo. mass	Combined Score	MS Cov. [%]	Pattern Cov. [%]
494.12258	$C_{25}H_{17}ON_{9}{}^{35}Cl$	21.5	-2.69	-1.33	494.12391	93.71	95.52	100
494.12258	$C_{24}H_{21}O_{5}N_{5}{}^{35}Cl$	16.5	0.02	0.01	494.12257	93.07	95.46	99.9
494.12258	$C_{23}H_{25}O_{9}N^{35}Cl$	11.5	2.72	1.34	494.12124	85.37	88.88	94.08
494.12258	$C_{12}H_{25}O_{12}N_{7}{}^{35}Cl$	3.5	-3.73	-1.84	494.12442	72.66	75.03	87.68
494.12258	$C_{7}H_{25}O_{14}N_{9}{}^{35}Cl$	-0.5	4.41	2.18	494.1204	51.85	54.38	68.7

FT8695_240923093825 #172-177 RT: 2.35-2.43 AV: 6 SB: 4 2.21-2.25 NL: 8.60E7
T: FTMS + p APCI corona Full ms [100.00-500.00]

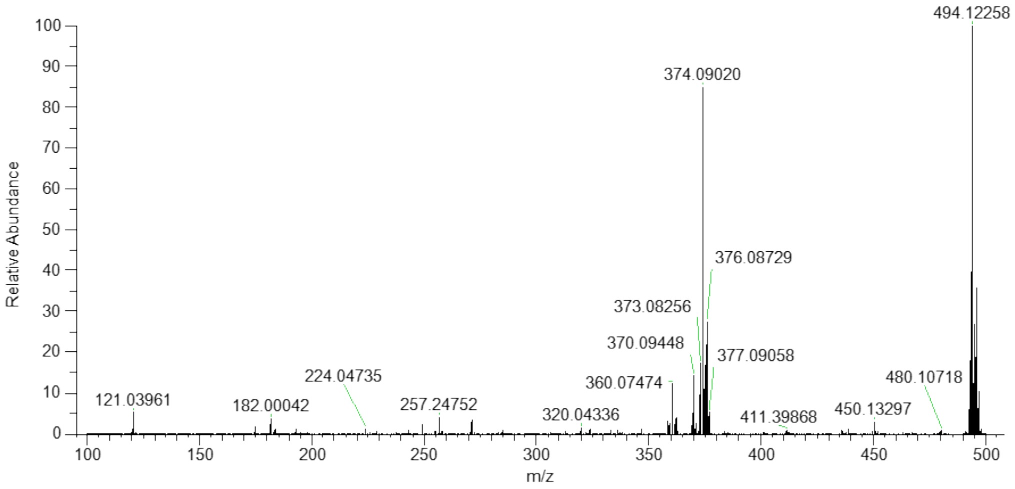

FT8695_240923093825 #172-177 RT: 2.35-2.43 AV: 6 SB: 4 2.21-2.25 NL: 8.60E7
T: FTMS + p APCI corona Full ms [100.00-500.00]

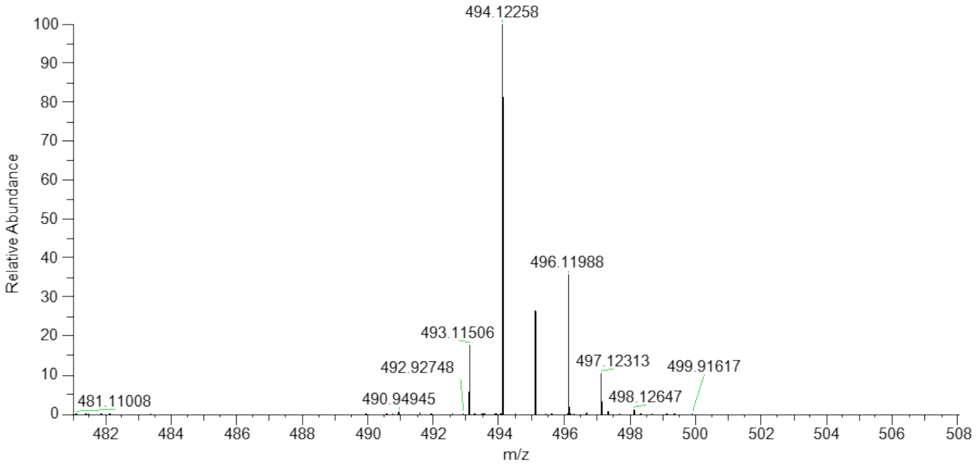

X-ray crystallography

Compound 10

R_1=4.96

Submitted by: **Zafer Sahin, Liotta Lab**

Solved by: **John Bacsa**

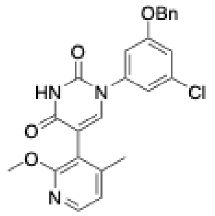

Crystal Data and Experimental

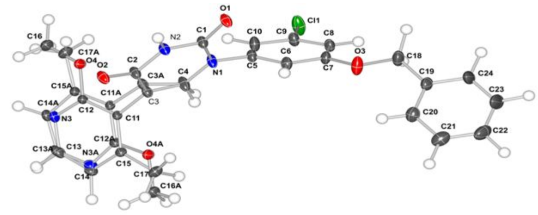

Experimental. Single colorless block-shaped crystals of ZS-Mpro-18 were chosen from the sample as supplied. A suitable crystal with dimensions 0.51 × 0.41 × 0.29 mm^3 was selected and mounted on a loop with paratone on a XtaLAB Synergy, Dualflex, HyPix diffractometer. The crystal was kept at a steady T = 100.03(10) K during data collection. The structure was solved with the ShelXT 2018/2 (Sheldrick, 2018) solution program using iterative methods and by using Olex2 1.5-alpha (Dolomanov et al., 2009) as the graphical interface. The model was refined with olex2.refine 1.5-alpha (Bourhis et al., 2015) using full matrix least squares minimisation on $\boldsymbol{F}^2$.

Crystal Data. $C_{24.5}H_{23}ClN_3O_5$, M_r = 474.925, triclinic, P-1 (No. 2), a = 10.1257(3) Å, b = 10.9097(3) Å, c = 11.25(3) Å, α = 82.518(2)°, β = 79.468(2)°, γ = 66.901(3)°, V = 1118.68(6) Å^3, T = 100.03(10) K, Z = 2, Z' = 1, μ(Mo K_α) = 0.214, 30008 reflections measured, 7795 unique (R_{int} = 0.0353) which were used in all calculations. The final wR_2 was 0.1112 (all data) and R_1 was 0.0496 (I≥2 σ(I)).

**Compound**	ZS-Mpro-18
Formula	$C_{24.5}H_{23}ClN_3O_5$
$D_{calc.}$/ g cm^{-3}	1.410
μ/mm^{-1}	0.214
Formula Weight	474.925
Color	colorless
Shape	block-shaped
Size/mm^3	0.51×0.41×0.29
T/K	100.03(10)
Crystal System	triclinic
Space Group	P-1
a/Å	10.1257(3)
b/Å	10.9097(3)
c/Å	11.2215(3)
α/°	82.518(2)
β/°	79.468(2)
γ/°	66.901(3)
V/Å^3	1118.68(6)
Z	2
Z'	1
Wavelength/Å	0.71073
Radiation type	Mo K_α
Θ_{min}/°	3.70
Θ_{max}/°	33.69
Measured Refl's.	30008
Indep't Refl's	7795
Refl's I≥2 σ(I)	6192
R_{int}	0.0353
Parameters	488
Restraints	489
Largest Peak	0.8384
Deepest Hole	-0.5664
GooF	1.0427
wR_2 (all data)	0.1112
wR_2	0.1051
R_1 (all data)	0.0644
R_1	0.0496

Structure Quality Indicators

**Reflections:**	d min (MoKα) 2Θ=67.4° 0.64	I/σ(I) 26.9	Rint m=3.80 3.53%	Full 50.5° 88% to 67.4° 98.3
**Refinement:**	Shift -0.001	Max Peak 0.8	Min Peak -0.6	GooF 1.043

A colorless block-shaped-shaped crystal with dimensions 0.51 × 0.41 × 0.29 mm^3 was mounted on a loop with paratone. Data were collected using a XtaLAB Synergy, Dualflex, HyPix diffractometer equipped with an Oxford Cryosystems low-temperature device operating at T = 100.03(10) K.

Data were measured using ω scans with Mo K_α radiation. The diffraction pattern was indexed and the total number of runs and images was based on the strategy calculation from the program CrysAlisPro system (CCD 43.92a 64-bit (release 05-10-2023)). The maximum resolution that was achieved was Θ = 33.69° (0.64 Å).

The unit cell was refined using CrysAlisPro 1.171.43.103a (Rigaku OD, 2023) on 13494 reflections, 45% of the observed reflections.

Data reduction, scaling and absorption corrections were performed using CrysAlisPro 1.171.43.103a (Rigaku OD, 2023). The final completeness is 98.27 % out to 33.69° in Θ. A numerical absorption correction based on gaussian integration over a multifaceted crystal model was performed using CrysAlisPro 1.171.42.74a (Rigaku Oxford Diffraction, 2022). An empirical absorption correction using spherical harmonics, implemented in SCALE3 ABSPACK scaling algorithm was also applied. The absorption coefficient μ of this material is 0.214 mm^{-1} at this wavelength (λ = 0.71073Å) and the minimum and maximum transmissions are 0.933 and 0.960.

The structure was solved and the space group *P*-1 (# 2) determined by the ShelXT 2018/2 (Sheldrick, 2018) structure solution program using using iterative methods and refined by full matrix least squares minimisation on $\boldsymbol{F}^2$ using version of olex2.refine 1.5-alpha (Bourhis et al., 2015). All non-hydrogen atoms and most hydrogen atoms were refined anisotropically. Most hydrogen atom positions were refined using the Hirshfeld model. Refinement was by using NoSpherA2, an implementation of non-spherical atom-form-factors (F. Kleemiss, H. Puschmann, O. Dolomanov, S.Grabowsky - https://doi.org/10.1039/D0SC05526C – 2020). NoSpherA2 implementation of HAR makes use of tailor-made aspherical atomic form factors calculated from a Hirshfeld-partitioned electron density (ED) not from spherical-atom form factors. The ED was calculated from a Gaussian basis set single determinant SCF wavefunction from DFT using selected functionals for a fragment of this crystal. This fragment was embedded in an electrostatic crystal field by employing cluster charges. The following options were used: SOFTWARE: ORCA PARTITIONING: NoSpherA2 INT ACCURACY: Normal METHOD: PBE BASIS SET: def2-SVP CHARGE: 0 MULTIPLICITY: 1 DATE: 2024-02-23_16-10-31.

There is a single formula unit in the asymmetric unit, which is represented by the reported sum formula. In other words: Z is 2 and Z' is 1. The moiety formula is C24 H20 Cl N3 O4, 1[H3], 1[O1], 0.5[C1]. The molecular structure is, on the whole, ordered; however, the methoxy-methyl-pyridine ring exists in two different rotational forms in the crystal structure, with one being the major form (~80% of the total structure).

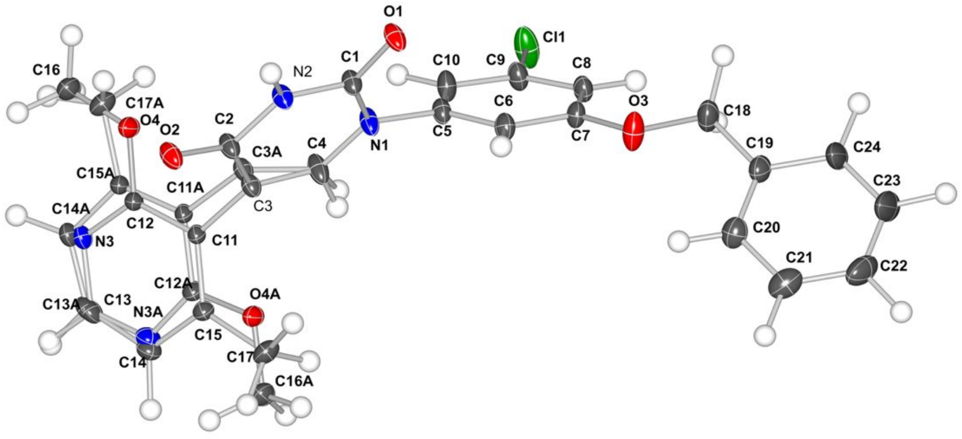

Figure S1 The asymmetric unit showing both components of the disorder. There is also a disordered water/methanol molecule in the crystal structure.

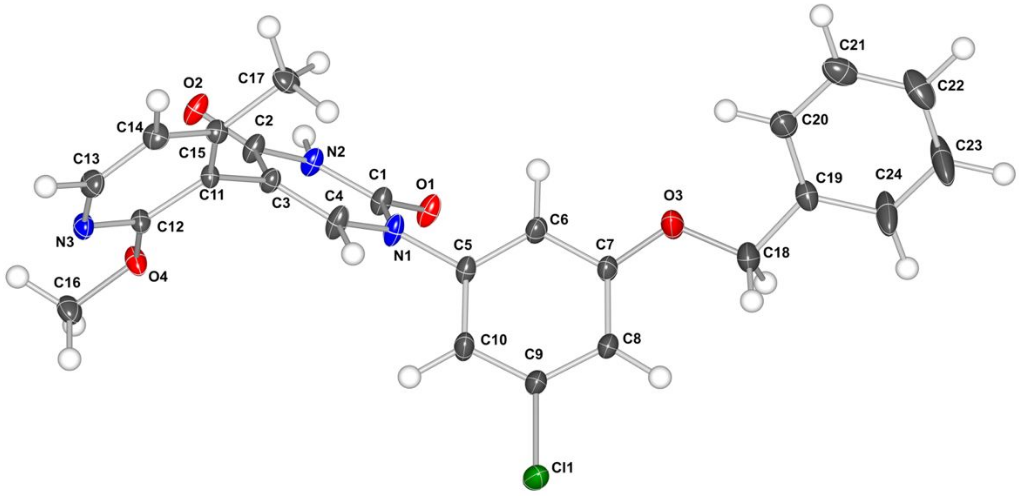

Figure S2 The asymmetric unit with the major component (0.80) of the disordered group.

Data Plots: Diffraction Data

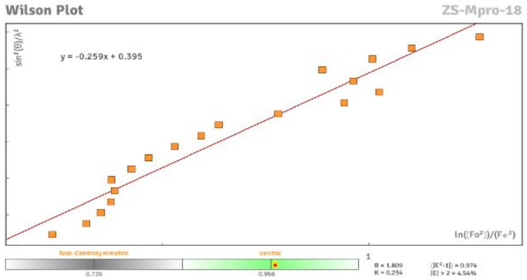

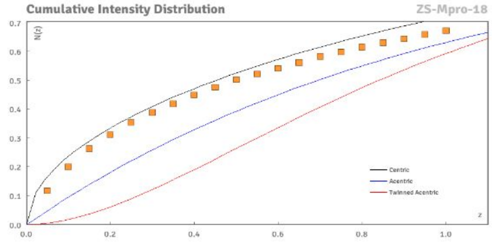

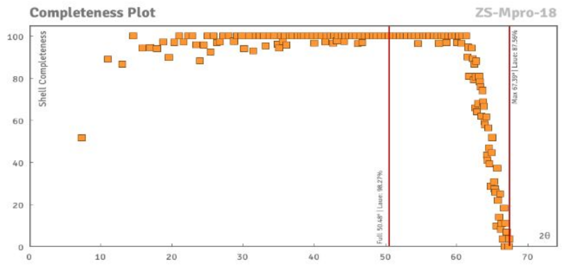

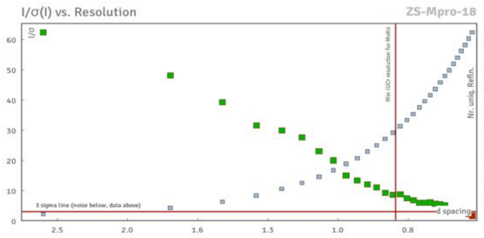

Data Plots: Refinement and Data

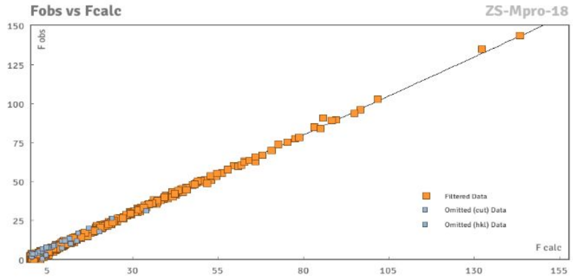

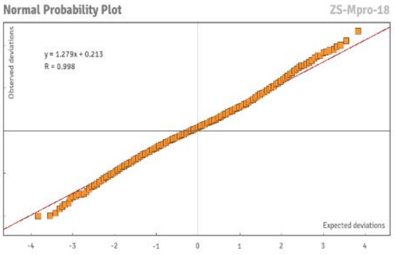

Reflection Statistics

Total reflections (after filtering)	29642	Unique reflections	7795
Completeness	0.876	Mean I/σ	15.75
hkl_{max} collected	(15, 16, 17)	hkl_{min} collected	(-15, -16, -16)
hkl_{max} used	(15, 16, 17)	hkl_{min} used	(-14, -16, 0)
Lim d_{max} collected	100.0	Lim d_{min} collected	0.36
d_{max} used	5.5	d_{min} used	0.64
Friedel pairs	5798	Friedel pairs merged	1
Inconsistent equivalents	0	R_{int}	0.0352
R_{sigma}	0.0372	Intensity transformed	0
Omitted reflections	366	Omitted by user (OMIT hkl)	2
Multiplicity	(4999, 4112, 2642, 1101, 482, 238, 67, 14, 4)	Maximum multiplicity	12
Removed systematic absences	0	Filtered off (Shel/OMIT)	0

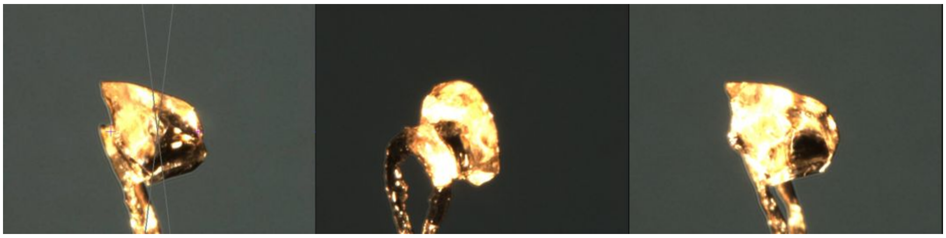

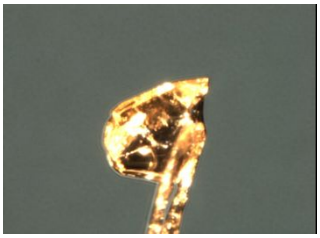

Table 1: Fractional Atomic Coordinates (×10[4]) and Equivalent Isotropic Displacement Parameters (Å[2]×10[3]) for ZS-Mpro-18. U_{eq} is defined as 1/3 of the trace of the orthogonalised U_{ij}.

Atom	x	y	z	U_{eq}
Cl1	7219.9(4)	2267.9(4)	816.3(4)	36.84(10)
O1	5957.4(9)	8311.1(8)	234.8(8)	22.15(18)
O2	1377.0(9)	10684.0(8)	1951.6(8)	22.78(18)
O3	9807.6(10)	5005.9(10)	2059.1(11)	32.1(2)
N1	4829.2(10)	7223.5(10)	1672.5(9)	20.7(2)
N2	3652.5(10)	9465.7(9)	1104.4(9)	18.37(19)
C1	4884.2(12)	8338.7(11)	959.4(11)	18.2(2)
C2	2372.6(12)	9597.1(9)	1875.0(9)	19.2(2)
C4	3585.5(10)	7258.5(9)	2439.9(11)	23.1(2)
C5	6099.6(12)	6018.4(11)	1603.4(11)	18.1(2)
C6	7371.4(12)	6029.2(12)	1881.9(11)	20.0(2)
C7	8629.1(12)	4877.4(12)	1775.5(11)	19.2(2)
C8	8596.6(12)	3704.4(12)	1430.7(11)	20.3(2)
C9	7283.7(13)	3731.2(12)	1199.0(12)	22.1(2)
C10	6023.4(13)	4871.4(12)	1260.8(12)	23.2(2)
C18	11183.0(14)	3959.3(15)	1896.9(14)	29.5(3)
C19	12087.7(13)	4038.0(13)	2782.8(12)	23.8(2)
C20	11500.5(15)	4827.5(13)	3771.2(13)	26.5(3)
C21	12366.0(17)	4785.7(14)	4622.9(14)	31.1(3)
C22	13819.2(18)	3965.6(19)	4488.2(14)	38.5(3)
C23	14413.5(18)	3184(3)	3493.0(16)	55.8(6)
C24	13554.2(15)	3219(2)	2645.6(14)	45.1(4)
O4	101.1(11)	8615.5(12)	1602.9(9)	21.1(2)
N3	-1362.1(18)	8382(3)	3404.5(14)	19.1(4)
C3	2364.8(9)	8374.6(8)	2563.5(10)	23.6(2)
C11	1046.7(11)	8370.2(17)	3399.4(10)	15.0(3)
C12	-126.2(16)	8454(2)	2826.8(11)	16.7(3)
C13	-1495(4)	8265(7)	4632.8(15)	20.4(4)
C14	-439(3)	8210(5)	5271.0(14)	20.2(4)
C15	878.4(16)	8263(2)	4652.4(12)	16.9(3)
C16	-998.8(17)	8669.8(19)	948.3(14)	24.3(3)
C17	2016.8(17)	8199.0(17)	5369.0(15)	22.8(3)
O4A	1901(4)	8219(5)	4800(3)	21.1(2)
N3A	-399(10)	8120(20)	5098(4)	20.2(4)
C3A	2364.8(9)	8374.6(8)	2563.5(10)	23.6(2)
C11A	921(4)	8329(8)	3069(3)	15.0(3)

Atom	x	y	z	U_{eq}
C12A	724(5)	8242(10)	4376(3)	16.9(3)
C13A	-1518(14)	8200(30)	4538(5)	20.4(4)
C14A	-1540(7)	8526(18)	3322(5)	23(2)
C15A	-250(5)	8478(11)	2541(4)	16.7(3)
C16A	1813(7)	8223(8)	6080(4)	24.3(3)
C17A	-288(6)	8551(7)	1207(4)	22.8(3)

Table 2: Anisotropic Displacement Parameters (×10[4]) for ZS-Mpro-18. The anisotropic displacement factor exponent takes the form: $-2\pi^2[h^2a^{*2} \times U_{11} + ... + 2hka^* \times b^* \times U_{12}]$

Atom	U_{11}	U_{22}	U_{33}	U_{23}	U_{13}	U_{12}
Cl1	20.32(15)	27.65(17)	64.5(3)	-5.12(12)	-5.92(15)	-22.74(16)
O1	15.6(4)	16.3(4)	26.7(4)	-3.2(3)	5.3(3)	5.0(3)
O2	18.4(4)	15.0(4)	25.8(4)	-0.9(3)	4.0(3)	2.5(3)
O3	19.7(4)	22.0(5)	55.8(7)	-3.3(3)	-15.1(4)	-7.3(4)
N1	12.2(4)	15.5(4)	27.9(5)	-2.9(3)	2.2(3)	5.9(3)
N2	14.9(4)	13.6(4)	22.1(5)	-3.7(3)	1.5(3)	3.4(3)
C1	13.7(5)	14.3(5)	22.5(5)	-4.0(3)	1.3(4)	3.7(4)
C2	15.0(5)	13.9(5)	22.6(5)	-2.6(3)	2.5(4)	2.9(4)
C4	14.4(5)	15.0(5)	32.2(6)	-3.0(4)	4.1(4)	5.9(4)
H4A	15(8)	15(3)	49(12)	2(2)	16(5)	10(2)
C5	12.4(4)	14.9(5)	23.3(5)	-3.0(3)	-1.1(4)	2.6(4)
C6	15.3(5)	15.4(5)	26.9(6)	-3.2(4)	-3.7(4)	-0.0(4)
H6	25(7)	19(4)	64(12)	-4(2)	-15(5)	-9(3)
C7	14.1(5)	16.2(5)	25.2(6)	-2.5(3)	-5.4(4)	-1.1(4)
C8	14.5(5)	18.5(5)	25.0(6)	-2.1(4)	-3.0(4)	-4.9(4)
H8	18(4)	23(3)	60(12)	1.6(17)	-10(3)	-13(3)
C9	15.0(5)	19.3(5)	30.3(6)	-3.1(4)	-3.0(4)	-6.8(4)
C10	13.0(5)	19.5(5)	34.5(6)	-3.1(4)	-3.8(4)	-2.6(4)
H10	18(4)	34(8)	76(13)	-5(2)	-14(3)	-16(5)
C18	18.0(5)	31.6(7)	36.6(7)	-2.9(5)	-9.1(5)	-7.4(5)
H18A	38(7)	65(9)	39(3)	-18(4)	-5.3(16)	-8.5(18)
H18B	31(7)	35(3)	70(8)	-9.4(19)	-20(4)	-2(2)
C19	16.3(5)	29.7(6)	25.9(6)	-8.9(4)	-4.9(4)	0.4(4)
C20	25.8(6)	20.4(6)	34.2(7)	-8.0(5)	-8.7(5)	-1.4(5)
H20	31(3)	55(9)	53(9)	4(2)	-16(2)	-23(5)
C21	41.7(8)	26.8(6)	32.4(7)	-19.0(6)	-13.5(5)	3.4(5)
H21	57(8)	60(10)	48(5)	-24(4)	-10(3)	-18(3)
C22	32.6(7)	60.3(10)	30.5(7)	-24.9(6)	-13.9(5)	6.9(6)
H22	41(7)	80(12)	36(6)	-28(5)	-19(3)	3(5)
C23	17.5(7)	110.5(17)	33.7(8)	-14.3(8)	-7.9(5)	-11.1(8)
H23	25(3)	230(30)	102(13)	15(3)	-29(3)	-97(10)
C24	13.4(6)	83.5(13)	27.7(7)	-4.2(6)	-3.4(5)	-11.5(7)
H24	42(8)	118(15)	42(7)	13(5)	-13(4)	-33(5)

Atom	U_{11}	U_{22}	U_{33}	U_{23}	U_{13}	U_{12}
H2	30(7)	16(3)	39(10)	-4(2)	11(4)	7(2)
O4	14.6(5)	35.2(6)	14.3(5)	-9.7(4)	-3.1(3)	-1.7(4)
N3	14.9(6)	21.0(10)	19.0(7)	-5.0(6)	-0.3(4)	-1.5(5)
C3	15.5(5)	15.2(5)	31.7(6)	-3.0(3)	6.4(4)	4.5(4)
C11	13.6(5)	16.0(5)	12.6(6)	-4.1(4)	0.9(4)	0.4(6)
C12	16.9(5)	19.7(5)	9.9(6)	-4.7(4)	1.1(4)	0.0(7)
C13	16.0(5)	24.9(10)	19.4(6)	-8.5(4)	3.3(4)	-3.8(7)
C14	19.5(5)	22.4(9)	17.1(6)	-7.8(5)	1.5(5)	-2.2(9)
C15	15.8(6)	16.8(5)	17.4(7)	-5.7(4)	-3.3(4)	1.2(6)
C16	22.2(7)	32.3(8)	18.9(6)	-9.6(6)	-4.3(5)	-3.4(5)
C17	26.5(8)	26.0(7)	18.9(7)	-10.6(6)	-11.1(6)	1.4(6)
H13	24(4)	74(15)	31(7)	-26(3)	7(2)	-2(4)
H14	37(10)	79(16)	18(2)	-33(7)	1.5(12)	-1.9(14)
H17A	38(7)	30(3)	43(10)	-8.5(18)	-13(4)	5.4(19)
H17B	39(6)	42(7)	41(9)	-22(3)	-5(3)	3(4)
H17C	45(11)	42(8)	28(5)	-12(4)	-9(3)	-8(3)
O4A	14.6(5)	35.2(6)	14.3(5)	-9.7(4)	-3.1(3)	-1.7(4)
N3A	19.5(5)	22.4(9)	17.1(6)	-7.8(5)	1.5(5)	-2.2(9)
C3A	15.5(5)	15.2(5)	31.7(6)	-3.0(3)	6.4(4)	4.5(4)
C11A	13.6(5)	16.0(5)	12.6(6)	-4.1(4)	0.9(4)	0.4(6)
C12A	15.8(6)	16.8(5)	17.4(7)	-5.7(4)	-3.3(4)	1.2(6)
C13A	16.0(5)	24.9(10)	19.4(6)	-8.5(4)	3.3(4)	-3.8(7)
C14A	23(3)	23(3)	23(3)	-7.8(10)	-3.8(9)	-1.3(9)
C15A	16.9(5)	19.7(5)	9.9(6)	-4.7(4)	1.1(4)	0.0(7)
C16A	22.2(7)	32.3(8)	18.9(6)	-9.6(6)	-4.3(5)	-3.4(5)
C17A	26.5(8)	26.0(7)	18.9(7)	-10.6(6)	-11.1(6)	1.4(6)

Table 3: Bond Lengths in Å for ZS-Mpro-18.

Atom	Atom	Length/Å
Cl1	C9	1.7341(13)
O1	C1	1.2250(13)
O2	C2	1.2194(12)
O3	C7	1.3515(15)
O3	C18	1.4107(16)
N1	C1	1.3809(14)
N1	C4	1.3796(14)
N1	C5	1.4326(14)
N2	C1	1.3669(14)
N2	C2	1.3873(14)
N2	H2	1.009(17)
C2	C3	1.4537(3)
C2	C3A	1.4537(3)
C4	H4B	1.0730

Atom	Atom	Length/Å
C4	H4A	1.076(18)
C4	C3	1.3528(3)
C4	C3A	1.3528(3)
C5	C6	1.3843(16)
C5	C10	1.3898(17)
C6	H6	1.083(16)
C6	C7	1.3939(15)
C7	C8	1.3982(17)
C8	H8	1.058(16)
C8	C9	1.3891(17)
C9	C10	1.3876(16)
C10	H10	1.067(17)
C18	H18A	1.142(19)
C18	H18B	1.037(18)

Atom	Atom	Length/Å
C18	C19	1.5006(19)
C19	C20	1.3898(19)
C19	C24	1.3922(19)
C20	H20	1.016(17)
C20	C21	1.395(2)
C21	H21	1.106(19)
C21	C22	1.382(2)
C22	H22	1.090(18)
C22	C23	1.391(3)
C23	H23	1.07(2)
C23	C24	1.389(2)
C24	H24	1.12(2)
O4	C12	1.3504(14)
O4	C16	1.4210(16)
N3	C12	1.3268(16)
N3	C13	1.3543(18)
C3	C11	1.4858(4)
C11	C12	1.4177(16)
C11	C15	1.3801(15)
C13	C14	1.3710(18)
C13	H13	1.057(19)
C14	C15	1.4052(17)
C14	H14	1.10(2)
C15	C17	1.4973(17)
C16	H16A	1.03(2)

Atom	Atom	Length/Å
C16	H16B	1.08(2)
C16	H16C	1.03(2)
C17	H17A	1.06(2)
C17	H17B	1.07(2)
C17	H17C	1.07(2)
O4A	C12A	1.353(3)
O4A	C16A	1.423(3)
N3A	C12A	1.310(3)
N3A	C13A	1.362(3)
C3A	C11A	1.4862(4)
C11A	C12A	1.438(3)
C11A	C15A	1.365(2)
C13A	H13A	1.0780
C13A	C14A	1.366(3)
C14A	H14A	1.0780
C14A	C15A	1.421(3)
C15A	C17A	1.495(3)
C16A	H16D	1.0780
C16A	H16E	1.0780
C16A	H16F	1.0780
C17A	H17D	1.0780
C17A	H17E	1.0780
C17A	H17F	1.0780

Table 4: Bond Angles in ° for ZS-Mpro-18.

Atom	Atom	Atom	Angle/°
C18	O3	C7	120.81(10)
C4	N1	C1	121.22(9)
C5	N1	C1	118.53(9)
C5	N1	C4	120.25(9)
C2	N2	C1	127.09(9)
H2	N2	C1	117.3(10)
H2	N2	C2	115.6(10)
N1	C1	O1	122.13(10)
N2	C1	O1	122.47(10)
N2	C1	N1	115.40(9)
N2	C2	O2	120.03(8)
C3	C2	O2	125.19(9)
C3	C2	N2	114.76(8)
C3A	C2	O2	125.19(9)
C3A	C2	N2	114.76(8)
C3A	C2	C3	0.0

Atom	Atom	Atom	Angle/°
H4B	C4	N1	118.56(5)
H4A	C4	N1	112.7(9)
H4A	C4	H4B	5.8(9)
C3	C4	N1	122.88(9)
C3	C4	H4B	118.56(6)
C3	C4	H4A	124.4(9)
C3A	C4	N1	122.88(9)
C3A	C4	H4B	118.56(6)
C3A	C4	H4A	124.4(9)
C3A	C4	C3	0.0
C6	C5	N1	118.96(10)
C10	C5	N1	119.26(10)
C10	C5	C6	121.78(10)
H6	C6	C5	122.3(9)
C7	C6	C5	119.57(11)
C7	C6	H6	118.0(9)

Atom	Atom	Atom	Angle/°
C6	C7	O3	114.49(11)
C8	C7	O3	125.19(10)
C8	C7	C6	120.29(11)
H8	C8	C7	124.0(9)
C9	C8	C7	117.98(10)
C9	C8	H8	118.0(9)
C8	C9	Cl1	118.32(9)
C10	C9	Cl1	118.55(9)
C10	C9	C8	123.13(11)
C9	C10	C5	117.17(11)
H10	C10	C5	120.4(10)
H10	C10	C9	122.5(10)
H18A	C18	O3	109.0(10)
H18B	C18	O3	111.1(10)
H18B	C18	H18A	108.8(15)
C19	C18	O3	109.48(11)
C19	C18	H18A	111.7(10)
C19	C18	H18B	106.8(10)
C20	C19	C18	122.58(12)
C24	C19	C18	118.17(12)
C24	C19	C20	119.11(13)
H20	C20	C19	120.1(10)
C21	C20	C19	120.38(13)
C21	C20	H20	119.5(10)
H21	C21	C20	119.9(11)
C22	C21	C20	120.36(14)
C22	C21	H21	119.6(11)
H22	C22	C21	118.3(11)
C23	C22	C21	119.39(14)
C23	C22	H22	122.3(11)
H23	C23	C22	119.8(13)
C24	C23	C22	120.42(16)
C24	C23	H23	119.7(13)
C23	C24	C19	120.33(15)
H24	C24	C19	120.7(11)
H24	C24	C23	118.9(11)
C16	O4	C12	118.40(11)
C13	N3	C12	115.82(14)
C4	C3	C2	118.57(8)
C11	C3	C2	120.21(9)
C11	C3	C4	121.21(10)
C12	C11	C3	115.32(10)
C15	C11	C3	126.55(11)
C15	C11	C12	118.11(9)
N3	C12	O4	120.72(12)
C11	C12	O4	114.43(10)

Atom	Atom	Atom	Angle/°
C11	C12	N3	124.85(11)
C14	C13	N3	123.68(13)
H13	C13	N3	118.6(12)
H13	C13	C14	117.7(12)
C15	C14	C13	120.18(13)
H14	C14	C13	118.4(11)
H14	C14	C15	121.4(11)
C14	C15	C11	117.31(12)
C17	C15	C11	123.49(11)
C17	C15	C14	119.20(12)
H16A	C16	O4	104.1(12)
H16B	C16	O4	110.6(12)
H16B	C16	H16A	113.9(17)
H16C	C16	O4	109.9(12)
H16C	C16	H16A	107.4(17)
H16C	C16	H16B	110.7(17)
H17A	C17	C15	111.4(12)
H17B	C17	C15	112.1(12)
H17B	C17	H17A	103.3(17)
H17C	C17	C15	110.6(13)
H17C	C17	H17A	113.7(18)
H17C	C17	H17B	105.4(18)
C16A	O4A	C12A	117.3(3)
C13A	N3A	C12A	115.5(3)
C4	C3A	C2	118.57(8)
C11A	C3A	C2	116.7(3)
C11A	C3A	C4	122.3(3)
C12A	C11A	C3A	110.78(19)
C15A	C11A	C3A	132.5(2)
C15A	C11A	C12A	116.43(19)
N3A	C12A	O4A	122.3(2)
C11A	C12A	O4A	111.5(2)
C11A	C12A	N3A	126.2(2)
H13A	C13A	N3A	118.8(2)
C14A	C13A	N3A	122.4(3)
C14A	C13A	H13A	118.8(2)
H14A	C14A	C13A	119.9(3)
C15A	C14A	C13A	120.2(3)
C15A	C14A	H14A	119.9(2)
C14A	C15A	C11A	117.5(2)
C17A	C15A	C11A	125.9(3)
C17A	C15A	C14A	116.5(3)
H16D	C16A	O4A	109.5
H16E	C16A	O4A	109.5
H16E	C16A	H16D	109.5
H16F	C16A	O4A	109.5

Atom	Atom	Atom	Angle/°
H16F	C16A	H16D	109.5
H16F	C16A	H16E	109.5
H17D	C17A	C15A	109.5
H17E	C17A	C15A	109.5
H17E	C17A	H17D	109.5
H17F	C17A	C15A	109.5
H17F	C17A	H17D	109.5
H17F	C17A	H17E	109.5

Table 5: Torsion Angles in ° for ZS-Mpro-18.

Atom	Atom	Atom	Atom	Angle/°
Cl1	C9	C8	C7	-178.05(10)
Cl1	C9	C10	C5	178.22(10)
O1	C1	N1	C4	-176.79(11)
O1	C1	N1	C5	3.37(15)
O1	C1	N2	C2	178.46(11)
O2	C2	N2	C1	176.68(11)
O2	C2	C3	C4	-175.82(12)
O2	C2	C3	C11	3.05(15)
O2	C2	C3A	C4	-175.82(12)
O2	C2	C3A	C11A	21.4(2)
O3	C7	C6	C5	179.39(11)
O3	C7	C8	C9	178.36(13)
O3	C18	C19	C20	13.90(16)
O3	C18	C19	C24	-170.32(13)
N1	C1	N2	C2	-0.50(14)
N1	C4	C3	C2	-1.20(13)
N1	C4	C3	C11	179.94(10)
N1	C4	C3A	C2	-1.20(13)
N1	C4	C3A	C11A	160.57(18)
N1	C5	C6	C7	-177.66(10)
N1	C5	C10	C9	179.58(11)
N2	C2	C3	C4	2.65(12)
N2	C2	C3	C11	-178.47(10)
N2	C2	C3A	C4	2.65(12)
N2	C2	C3A	C11A	-160.1(2)
C2	C3	C11	C12	72.73(15)
C2	C3	C11	C15	-108.87(15)
C2	C3A	C11A	C12A	-115.0(5)
C2	C3A	C11A	C15A	57.9(6)
C4	C3	C11	C12	-108.42(15)
C4	C3	C11	C15	69.97(16)
C4	C3A	C11A	C12A	82.9(5)
C4	C3A	C11A	C15A	-104.2(6)
C5	C6	C7	C8	-2.22(14)
C5	C10	C9	C8	-1.70(15)
C6	C7	C8	C9	0.15(14)

Atom	Atom	Atom	Atom	Angle/°
C7	C8	C9	C10	1.86(14)
C18	C19	C20	C21	174.88(13)
C18	C19	C24	C23	-175.30(17)
C19	C20	C21	C22	0.46(16)
C19	C24	C23	C22	0.0(2)
C20	C21	C22	C23	0.18(18)
C21	C22	C23	C24	-0.4(2)
O4	C12	N3	C13	-177.9(4)
O4	C12	C11	C3	-4.2(2)
O4	C12	C11	C15	177.27(16)
N3	C12	C11	C3	175.6(2)
N3	C12	C11	C15	-2.9(3)
N3	C13	C14	C15	-0.7(7)
C3	C11	C15	C14	-176.8(3)
C3	C11	C15	C17	3.2(2)
C11	C15	C14	C13	0.1(4)
C13	C14	C15	C17	-179.9(4)
O4A	C12A	N3A	C13A	178.2(17)
O4A	C12A	C11A	C3A	-0.2(8)
O4A	C12A	C11A	C15A	-174.4(7)
N3A	C12A	C11A	C3A	-176.7(14)
N3A	C12A	C11A	C15A	9.1(16)
N3A	C13A	C14A	C15A	15(3)
C3A	C11A	C15A	C14A	-172.9(11)
C3A	C11A	C15A	C17A	9.2(10)
C11A	C15A	C14A	C13A	-11.0(18)
C13A	C14A	C15A	C17A	167.0(16)

Table 6: Hydrogen Fractional Atomic Coordinates (×10[4]) and Equivalent Isotropic Displacement Parameters (Å[2]×10[3]) for ZS-Mpro-18. U_{eq} is defined as 1/3 of the trace of the orthogonalised U_{ij}.

Atom	x	y	z	U_{eq}
H4B	3586.4(10)	6361.1(9)	2961.5(11)	27.7(3)
H4A	3700(20)	6308(19)	2914(19)	32(5)
H6	7441(18)	6917(16)	2151(17)	36(4)
H8	9509(17)	2795(16)	1342(17)	35(4)
H10	5027(18)	4886(18)	1060(19)	42(5)
H18A	11720(20)	4040(20)	916(18)	47(4)
H18B	11094(19)	3035(18)	2073(18)	45(4)
H20	10440(20)	5460(20)	3866(17)	49(5)
H21	11880(20)	5370(20)	5434(18)	53(5)
H22	14450(20)	3920(20)	5194(17)	50(5)
H23	15550(20)	2580(40)	3360(30)	128(13)
H24	14070(20)	2630(30)	1830(19)	77(8)

Atom	x	y	z	U_{eq}
H2	3680(20)	10312(17)	636(17)	33(5)
H16A	-670(20)	8960(20)	70(20)	36.5(5)
H16B	-1110(20)	7720(20)	1040(19)	36.5(5)
H16C	-1970(20)	9420(20)	1253(19)	36.5(5)
H13	-2480(20)	8250(30)	5134(19)	42(6)
H14	-660(20)	8140(30)	6271(18)	42(6)
H17A	2700(20)	7200(20)	5580(20)	38(4)
H17B	2760(20)	8620(20)	4860(20)	40(5)
H17C	1530(30)	8780(20)	6150(20)	38(5)
H13A	-2419(14)	8010(30)	5074(5)	24.5(5)
H14A	-2540(7)	8818(18)	2954(5)	28(3)
H16D	2600(40)	8580(50)	6275(4)	36.5(5)
H16E	739(19)	8870(40)	6451(8)	36.5(5)
H16F	2030(60)	7223(12)	6483(7)	36.5(5)
H17D	-70(60)	9410(30)	777(9)	34.2(5)
H17E	520(40)	7660(30)	825(10)	34.2(5)
H17F	-1350(20)	8640(60)	1059(4)	34.2(5)

Table 7: Atomic Occupancies for all atoms that are not fully occupied in ZS-Mpro-18.

Atom	Occupancy
H4B	0.1971(17)
H4A	0.8029(17)
O4	0.8029(17)
N3	0.8029(17)
C3	0.8029(17)
C11	0.8029(17)
C12	0.8029(17)
C13	0.8029(17)
C14	0.8029(17)
C15	0.8029(17)
C16	0.8029(17)
H16A	0.8029(17)
H16B	0.8029(17)

Atom	Occupancy
H16C	0.8029(17)
C17	0.8029(17)
H13	0.8029(17)
H14	0.8029(17)
H17A	0.8029(17)
H17B	0.8029(17)
H17C	0.8029(17)
O4A	0.1971(17)
N3A	0.1971(17)
C3A	0.1971(17)
C11A	0.1971(17)
C12A	0.1971(17)
C13A	0.1971(17)
H13A	0.1971(17)

Atom	Occupancy
C14A	0.1971(17)
H14A	0.1971(17)
C15A	0.1971(17)
C16A	0.1971(17)
H16D	0.1971(17)
H16E	0.1971(17)
H16F	0.1971(17)
C17A	0.1971(17)
H17D	0.1971(17)
H17E	0.1971(17)
H17F	0.1971(17)

Table 8: Solvent masking (PLATON/SQUEEZE) information for Compound **10**.

No	x	y	z	V	e	Content
1	0.500	0.000	0.500	76.2	23.4	2H3,2O1,1C1
